# Synergistic effects of cerebral small vessel disease burden and plasma phosphorylated tau 181 on white matter microstructure and cognition in a Chinese cohort

**DOI:** 10.1093/braincomms/fcag080

**Published:** 2026-03-12

**Authors:** Jingxian Xu, Hao-Jie Chen, Yichen Wang, Zheqi Hu, Zhihong Ke, Lili Huang, Yuting Mo, Dan Yang, Chenglu Mao, Ying Chen, Xiaolei Zhu, Haifeng Chen, Ni Shu, Yun Xu

**Affiliations:** Department of Neurology, Nanjing Drum Tower Hospital, Affiliated Hospital of Medical School, Nanjing University, Nanjing 210008, China; State Key Laboratory of Cognitive Neuroscience and Learning & IDG/McGovern Institute for Brain Research, Beijing Normal University, Beijing 100875, China; Division of Life Science and State Key Laboratory of Nervous System Disorders, The Hong Kong University of Science and Technology, Clear Water Bay, Kowloon, Hong Kong 999077, China; State Key Laboratory of Cognitive Neuroscience and Learning & IDG/McGovern Institute for Brain Research, Beijing Normal University, Beijing 100875, China; BABRI Center, Beijing Normal University, Beijing 100875, China; Beijing Key Laboratory of Brain Imaging and Connectomics, Beijing Normal University, Beijing 100875, China; Department of Neurology, Nanjing Drum Tower Hospital, Affiliated Hospital of Medical School, Nanjing University, Nanjing 210008, China; Department of Neurology, Nanjing Drum Tower Hospital, Affiliated Hospital of Medical School, Nanjing University, Nanjing 210008, China; Department of Neurology, Nanjing Drum Tower Hospital, Affiliated Hospital of Medical School, Nanjing University, Nanjing 210008, China; Jiangsu Key Laboratory of Molecular Medicine, Medical School of Nanjing University, Nanjing 210008, China; Jiangsu Province Stroke Center for Diagnosis and Therapy, Nanjing Drum Tower Hospital, Nanjing 210008, China; Nanjing Neuropsychiatry Clinic Medical Center, Nanjing Drum Tower Hospital, Nanjing 210008, China; Department of Neurology, Nanjing Drum Tower Hospital, Affiliated Hospital of Medical School, Nanjing University, Nanjing 210008, China; Jiangsu Key Laboratory of Molecular Medicine, Medical School of Nanjing University, Nanjing 210008, China; Jiangsu Province Stroke Center for Diagnosis and Therapy, Nanjing Drum Tower Hospital, Nanjing 210008, China; Nanjing Neuropsychiatry Clinic Medical Center, Nanjing Drum Tower Hospital, Nanjing 210008, China; Department of Neurology, Nanjing Drum Tower Hospital, Affiliated Hospital of Medical School, Nanjing University, Nanjing 210008, China; Department of Neurology, Nanjing Drum Tower Hospital, Affiliated Hospital of Medical School, Nanjing University, Nanjing 210008, China; Department of Neurology, Nanjing Drum Tower Hospital, Affiliated Hospital of Medical School, Nanjing University, Nanjing 210008, China; Department of Neurology, Nanjing Drum Tower Hospital, Affiliated Hospital of Medical School, Nanjing University, Nanjing 210008, China; Jiangsu Key Laboratory of Molecular Medicine, Medical School of Nanjing University, Nanjing 210008, China; Jiangsu Province Stroke Center for Diagnosis and Therapy, Nanjing Drum Tower Hospital, Nanjing 210008, China; Nanjing Neuropsychiatry Clinic Medical Center, Nanjing Drum Tower Hospital, Nanjing 210008, China; Department of Neurology, Nanjing Drum Tower Hospital, Affiliated Hospital of Medical School, Nanjing University, Nanjing 210008, China; Jiangsu Key Laboratory of Molecular Medicine, Medical School of Nanjing University, Nanjing 210008, China; Jiangsu Province Stroke Center for Diagnosis and Therapy, Nanjing Drum Tower Hospital, Nanjing 210008, China; Nanjing Neuropsychiatry Clinic Medical Center, Nanjing Drum Tower Hospital, Nanjing 210008, China; Department of Neurology, Nanjing Drum Tower Hospital, Nanjing Drum Tower Hospital Clinical College, Nanjing University of Chinese Medicine, Nanjing 210008, China; State Key Laboratory of Cognitive Neuroscience and Learning & IDG/McGovern Institute for Brain Research, Beijing Normal University, Beijing 100875, China; BABRI Center, Beijing Normal University, Beijing 100875, China; Beijing Key Laboratory of Brain Imaging and Connectomics, Beijing Normal University, Beijing 100875, China; Department of Neurology, Nanjing Drum Tower Hospital, Affiliated Hospital of Medical School, Nanjing University, Nanjing 210008, China; Jiangsu Key Laboratory of Molecular Medicine, Medical School of Nanjing University, Nanjing 210008, China; Jiangsu Province Stroke Center for Diagnosis and Therapy, Nanjing Drum Tower Hospital, Nanjing 210008, China; Nanjing Neuropsychiatry Clinic Medical Center, Nanjing Drum Tower Hospital, Nanjing 210008, China; Department of Neurology, Nanjing Drum Tower Hospital, Nanjing Drum Tower Hospital Clinical College, Nanjing University of Chinese Medicine, Nanjing 210008, China

**Keywords:** CSVD, cognitive impairment, plasma biomarker, white matter, synergistic effects

## Abstract

Cerebral small vessel disease (CSVD) burden and plasma biomarkers are both critically associated with white matter (WM) microstructural damage and cognitive decline. However, whether these factors interact synergistically to exacerbate brain degeneration and cognitive decline remains unclear. We included 375 Chinese participants from the Aging cohort at Nanjing Drum Tower Hospital: 144 with no CSVD, 103 with mild CSVD (CSVD-I) and 128 with moderate to severe CSVD (CSVD-II). All participants underwent comprehensive cognitive assessment, plasma biomarker quantification and MRI scanning. Diffusion tensor imaging was used to evaluate WM microstructure. Interaction effects between CSVD burden and plasma biomarkers were analysed, and path analyses were performed to explore how two factors synergistically influence WM tracts and cognitive impairment. We found significant interactions between phosphorylated tau 181 (p-tau_181_) and CSVD burden on the integrity of multiple WM tracts (*P*_FDR_ < 0.05). The CSVD-II showed the strongest effect of p-tau_181_ on neurofilament light, as well as an indirect effect of the cingulum mediating the relationship between p-tau_181_ and cognition [Δ$\bar{IE}$ = −0.108, 90% CI = (−0.25, −0.0302)]. Our findings suggest that vascular and molecular pathologies synergistically contribute to WM integrity damage and cognitive impairment. Targeting both vascular and molecular factors may be crucial for developing effective interventions to mitigate cognitive decline in the elderly population.

## Teaser

Xu *et al*. report that cerebral small vessel disease burden and plasma p-tau_181_ synergistically affect white matter microstructure and cognition in a Chinese cohort. The findings highlight a synergistic interaction between vascular and tau pathologies, underscoring the importance of integrated strategies for mitigating cognitive decline.

## Graphical Abstract

**Graphical Abstract:**
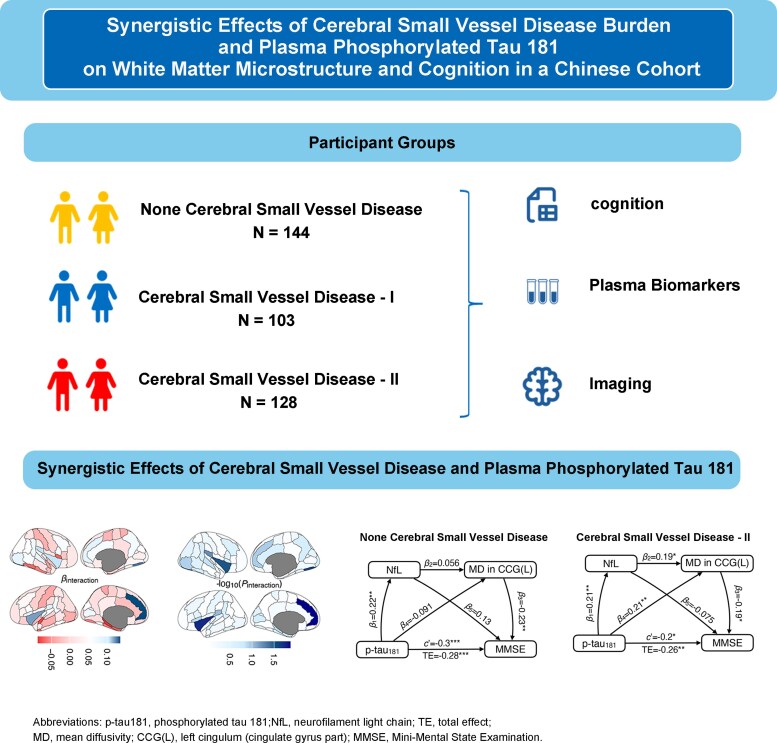

## Introduction

Cognitive impairment is a widespread and growing public health challenge in the elderly population.^1,2^ While cognitive decline is commonly associated with ageing, its underlying mechanisms involve complex interactions among vascular pathology and neurodegenerative processes.^3,4^ White matter (WM) tract disruptions have emerged as a key contributor to cognitive deterioration,^5,6^ given WM’s essential role in mediating communication between cortical and subcortical regions.^7^ The structural integrity of WM can be assessed at the microstructural level using diffusion tensor imaging (DTI) techniques.^8^ Increasing evidence indicates that abnormalities in WM microstructure are closely associated with cognitive impairment, including deficits in memory, attention and executive function (EF).^9,10^ Given its central role in cognitive function, it is important to investigate potential mechanisms, particularly those involving vascular damage and neurodegenerative pathology, that may disrupt WM integrity and cause cognitive decline.

Cerebral small vessel disease (CSVD) burden reflects the severity of vascular damage in the brain.^11^ It is common among the elderly and is a major factor in cognitive impairment and dementia.^12^ Neuroimaging markers of CSVD, including white matter hyperintensities (WMH), lacunes, enlarged perivascular spaces and microbleeds,^13^ reflect microstructure pathology such as demyelination, axonal loss, gliosis and microvascular ischaemia.^14^ DTI studies show reduced fractional anisotropy (FA) and increased mean diffusivity (MD) in individuals with CSVD, indicating a decline in the integrity of WM.^15,16^ These microstructural alterations disrupt large-scale communication between brain regions and are strongly associated with impairments in memory, EF and information processing speed (IPS).^16,17^ Together, these findings highlight the significant impact of CSVD burden on WM microstructure and cognition.

In addition to CSVD burden, fluid-based biomarkers for neurodegenerative diseases such as phosphorylated tau (p-tau) and beta-amyloid (Aβ) are closely associated with WM microstructural alterations.^18^ Plasma biomarkers offer an accessible alternative to CSF measurements and show strong correspondence with CSF levels.^19^ These biomarkers may reflect reduced axonal density and regional myelin content.^20^ Plasma Aβ_42_ was associated with white matter hyperintensity volume (WMHV), and CSF Aβ_42_ have been positively correlated with FA in the left fornix.^21,22^ Plasma p-tau_181_ was associated with WM microstructural alterations.^23^ These microstructural alterations may serve as a potential link between fluid biomarker abnormalities and impaired cognitive function. The findings suggest that abnormal plasma biomarker levels are associated with greater WMHV, lower WM microstructural integrity and lower global cognition.^24^ Taken together, these fluid biomarkers may serve as indicators of WM pathology and contribute to cognitive decline through their effects on WM microstructure.

Recent evidence suggests that CSVD and AD-related pathologies, particularly Aβ and tau accumulation, do not act independently but interact synergistically through shared biological mechanisms. Lower Aβ_42_/Aβ_40_ and elevated p-tau_181_ have a dependent neurotoxic effect and are associated with disruption of mitochondrial function, oxidative defence and calcium homeostasis.^25-28^ This process further compromises neurovascular coupling and WM integrity through progressive axonal degeneration. Conversely, chronic hypoperfusion and endothelial dysfunction in CSVD can induce increased blood–brain barrier leakage, facilitating the entry of plasma-derived neurotoxic molecules and inflammatory mediators into the brain.^29^ Concurrently, persistent blood–brain barrier dysfunction may exacerbate vascular damage and trigger secondary inflammation, thereby enhancing tau phosphorylation and aggregation.^30^ This bidirectional interaction between CSVD and neurodegenerative pathology may amplify WM injury and cognitive deterioration.

However, most previous studies examining vascular-neurodegenerative interactions have relied on isolated vascular markers such as WMH volume or CSF biomarkers,^26,31^ limiting their ability to characterize combined effects of comprehensive CSVD burden and plasma biomarkers on WM microstructure and cognitive performance. Therefore, our analysis focused on an elderly population from the Aging cohort at Nanjing Drum Tower Hospital, integrating CSVD imaging features, plasma biomarkers, cognitive assessments and advanced image processing techniques to quantify WM microstructural integrity. We used the path analysis to explore the potential mechanisms by which CSVD burden and plasma biomarkers may impair WM integrity and lead to cognitive impairment.

## Materials and methods

### Participants

**Figure 1:**
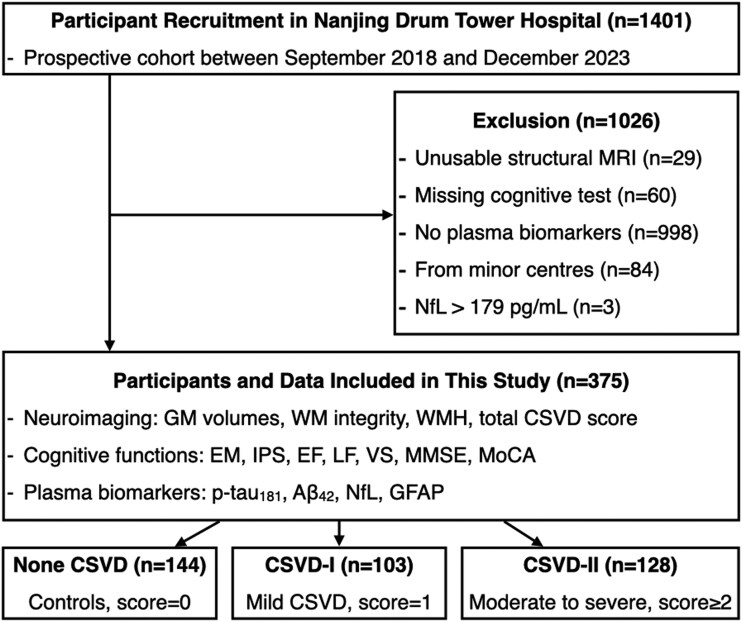
**Flow chart for participant selection and included data.** MRI, magnetic resonance imaging; CSVD, cerebral small vessel disease; CSVD-I, mild CSVD pathology; CSVD-II, moderate to severe CSVD pathology; GM, grey matter; WM, white matter; WMH, white matter hyperintensity; p-tau_181_, phosphorylated tau 181; Aβ_42_, amyloid beta 42; GFAP, glial fibrillary acidic protein; NfL, neurofilament light; EM, episodic memory; IPS, information processing speed; EF, executive function; LF, language function; VS, visuospatial function; MMSE, Mini-Mental State Examination; MoCA, Montreal Cognitive Assessment.

Participants were recruited from the Aging cohort at Nanjing Drum Tower Hospital (ChiCTR-EOC-17011961) (Fig. 1). The study was approved by the local institutional review board and all subjects provided written informed consent. We used the total CSVD score, which ranges from 0 to 4, to evaluate the CSVD burden of all the subjects. This score was derived from four visually assessed MRI features, each contributing one point if present: (i) lacunes, (ii) microbleeds, (iii) moderate to severe basal ganglia enlarged perivascular spaces and (iv) moderate to severe deep WMH or severe periventricular WMH (assessed using the Fazekas scale).^32^ The assessment was cross-evaluated by two trained physicians to ensure accuracy and reliability. Based on established criteria tailored for East-Asian populations,^32^ participants were further categorized into three CSVD burden grades according to their total CSVD score: no CSVD (score = 0, CSVD-0), mild CSVD (score = 1, CSVD-I) and moderate to severe CSVD (score ≥ 2, CSVD-II). Participants with unusable MRI data, missing cognitive test data, missing plasma biomarker data, heart disease or the potential presence of severe and unrelated CNS disease [neurofilament light chain (NfL) levels > 179 pg/ml],^33^ as well as those from minor centres, were excluded from the study.

### Plasma biomarkers

Plasma biomarkers were tested for all participants’ plasma samples from the Nanjing Drum Tower Hospital. Baseline measurements of Aβ_42_, p-tau_181_, NfL and glial fibrillary acidic protein (GFAP) concentrations were obtained using the Simoa HD-X analyser. P-tau_181_ concentration was measured using the Simoa® pTau-181 Advantage V2.1 Kit, while Aβ_42_, NfL and GFAP concentrations were measured using the Simoa® N4PE Advantage Kit. We employed a Gaussian mixture model using plasma Aβ_42_ levels from 375 participants to define Aβ positivity (cut-off value = 5.13).^34^

### Neuropsychological assessments

All participants underwent a standardized battery of neuropsychological assessments specifically tailored for Chinese population.^35^ Comprehensive cognitive functions were assessed with the Mini-Mental State Examination (MMSE) and the Montreal Cognitive Assessment (MoCA). Cognitive subdomains were assessed and calculated based on previously published methods for the same cohort,^36^ including Episodic Memory (EM) assessed using the Auditory Verbal Learning Test-Delayed Recall and the Auditory Verbal Learning Test-Recognition; IPS assessed using the Trail Making Test A, Stroop-A and Stroop-B; EF assessed using the Trail Making Test B and Stroop-C; Visuospatial Function (VS) assessed using the Clock Drawing Test and the Visual Reproduction Test; and Language Function (LF) assessed using the Category Verbal Fluency test and the Boston Naming Test. Our study included participants spanning the full cognitive spectrum, from normal cognition to cognitive impairment.

### Brain MRI acquisition and processing

Structural and diffusion MRI scans were acquired at Drum Tower Hospital using a 3T Philips Ingenia scanner. The parameters for T_1_-weighted imaging (T1WI) were as follows: gradient-echo sequence, field of view (FOV) = 256 mm^2^, slice thickness = 1 mm, matrix size = 256 × 256, number of slices = 192, gap = 0 mm, repetition time (TR) = 9.8 ms, inversion time (TI) = 0 ms, echo time (TE) = 4.6 ms, flip angle = 8° and voxel size = 1 × 1 × 1 mm^3^. The parameters for T2-FLAIR imaging were as follows: inversion recovery sequence, FOV = 288 mm^2^, slice thickness = 0.95 mm, matrix size = 288 × 288, number of slices = 200, gap = 0 mm, TR = 4500 ms, TI = 1600 ms, TE = 339 ms, flip angle = 90° and voxel size = 0.95 × 0.9 × 0.9 mm^3^. The parameters for diffusion MRI were as follows: single-shot echo-planar imaging sequence, FOV = 224 mm^2^, slice thickness = 2.5 mm, matrix size = 112 × 112, number of slices = 55, gap = 0 mm, TR = 8655 ms, TE = 70.9 ms, flip angle = 90°, voxel size = 2 × 2 × 2.5 mm^3^, 32 gradient directions (b = 1000 s/mm^2^) and one b = 0 s/mm^2^ image.

We employed the standard imaging processing pipeline from the UK Biobank to compute structural brain imaging markers, including regional GM volumes, WMHV and the microstructural integrity of WM tracts.^37,38^ Pre-processing for T_1_-weighted images included skull stripping^39^ and bias-field intensity correction.^40^ Pre-processing for diffusion MRI was conducted using the eddy command,^41^ incorporating susceptibility distortion correction, eddy current correction and head motion correction, with the field map estimated via synb0 software.^42^ Following pre-processing, regional GM volumes were estimated within GM segmentation^40^ under the Harvard–Oxford cortical and subcortical structural atlases of 110 parcellations.^43^ WMHV was calculated using the BIANCA programme, with segmentation performed based on both T_1_ and T_2_-FLAIR images.^44^ Volumetric measures were adjusted for head size by applying the scaling factor from native T_1_ space to MNI space, estimated through the SIENAX procedure.^45^ To evaluate the microstructural integrity of WM tracts, diffusion tensors were first fitted using the DTIFIT programme to compute the voxel-wise FA, MD and mode of anisotropy (MO). These measures were subsequently processed using TBSS^46^ to generate tract-wise metrics of 48 WM tracts.^47^

### Statistical analysis

All the statistical analyses were conducted using R version 4.5.2. To compare demographics among different CSVD burden grades, one-way ANOVA was employed for continuous variables, while *χ*² tests were used for categorical variables. We used linear regression models to explore the relationship between p-tau_181_ levels and their interaction with CSVD burden grade on NfL levels, adjusting for age, sex, education, diabetes, hypertension, intracranial volume and Aβ positivity. The significance of the p-tau_181_ effect within each CSVD burden grade, as well as the differences of the effects among grades, was assessed using bootstrapping with 5000 resamples. Effects were considered significant if the 90% BCa CI (bias-corrected and accelerated bootstrap interval) did not include zero.^48^

Next, linear regressions were employed to examine the relationships between p-tau_181_ levels and regional brain imaging markers, adjusting for the same covariates. Nominal 95% confidence intervals were used to determine the significance of the effects of p-tau_181_ and its interactions with the CSVD burden grade. False discovery rate (FDR) adjustment was applied across 124 brain GM regions (110 FAST segmentations and 14 FIRST segmentations) or 48 WM tracts to control for false positives.

Finally, path analyses were conducted using the lavaan package^49^ to investigate the relationships between p-tau_181_ and cognitive functions, potentially mediated by NfL and brain imaging markers.^50^ The main path model was specified as follows: NfL = *β*_1_ × p-tau_181_, imaging marker = *β*_2_ × NfL + *β*_4_ × p-tau_181_ and cognitive function = *β*_3_ × imaging marker + *β*_5_ × NfL + *c*′ × p-tau_181_. All paths were adjusted for age, sex and education. Three indirect effects were considered: *IE*_1_ = *β*_1_ × *β*_2_ × *β*_3_, *IE*_2_ = *β*_1_ × *β*_5_ and *IE*_3_ = *β*_3_ × *β*_4_. The total indirect effect (*IE*) was calculated as *IE* = *IE*_1_ + *IE*_2_ + *IE*_3_ and the total effect as *TE* = *c*′ + *IE*. Differences in path coefficients and indirect effects between CSVD burden grades were assessed with 90% BCa CIs through 5000 bootstrapped resamples.

### Supplementary analyses

To enhance clinical relevance, we explored the interactive effect between p-tau_181_ levels and WMHV tertiles on NfL and regional brain imaging markers. To address potential biases from the segmentation method, we replicated our analyses using regional subcortical grey matter volumes derived from FIRST segmentation.^51^ While the primary analyses utilized a 90% CI due to the limited sample size, we also calculated 95% CIs for a more stringent significance threshold. Additionally, we replaced p-tau_181_ with plasma Aβ_42_ and NfL with GFAP to assess the sensitivity and specificity of the synergistic effects between different plasma biomarkers and CSVD burden.

## Results

### Participant characteristics

Table 1 summarizes the characteristics of the participants across different grades of CSVD burden. This study included 375 participants: 144 with no CSVD, 103 with mild CSVD (CSVD-I) and 128 with moderate to severe CSVD (CSVD-II). Within the CSVD-II group, 70 participants had a score of 2, 41 participants had a score of 3 and 17 participants had a score of 4. The mean age of participants increased with the severity of CSVD burden (*P* < 0.001), from 65.73 ± 8.74 years in the no CSVD to 73.06 ± 7.92 years in moderate to severe CSVD. While GM volume did not differ significantly across CSVD burden groups (*P* = 0.2), WM volume was lower in moderate to severe CSVD compared to mild CSVD and no CSVD (*P* < 0.001). The WMH volume was higher in moderate to severe CSVD compared to the other groups (*P* < 0.001). A significant between-group difference was observed for plasma NfL levels (*P* = 0.002). We also noted increasing trends of plasma p-tau_181_ and NfL levels towards increase with higher CSVD burden. Significant differences were also observed in MoCA, IPS, LF, EF and VS, with performance generally declining as the CSVD burden increased (*P* < 0.05).

**Table 1:** Participant characteristics of different CSVD burden grades

Characteristic	No CSVD *N* = 144^a^	CSVD-I *N* = 103^a^	CSVD-II *N* = 128^a^	*P*-value^b^	Overall *N* = 375^a^
Demographic					
Age (years)	65.73 (8.74)	67.06 (9.24)	73.06 (7.92)	<0.001	68.59 (9.19)
Sex (%)				0.025	
Female	95 (66%)	66 (64%)	65 (51%)		226 (60%)
Male	49 (34%)	37 (36%)	63 (49%)		149 (40%)
Education (years)	11.88 (4.74)	11.35 (4.40)	10.66 (4.64)	0.10	11.32 (4.63)
Hypertension	41 (28%)	29 (28%)	69 (54%)	<0.001	139 (37%)
Diabetes	15 (10%)	10 (9.7%)	33 (26%)	<0.001	58 (15%)
Brain imaging					
GMV (ml)	997.74 (127.21)	997.67 (128.73)	970.01 (116.46)	0.12	988.26 (124.44)
WMV (ml)	896.36 (102.28)	891.67 (91.40)	848.21 (92.12)	<0.001	878.64 (98.21)
WMHV (ml)	6.04 (6.96)	8.41 (9.43)	19.20 (19.03)	<0.001	11.18 (14.14)
CSFV (ml)	770.63 (99.93)	751.43 (104.11)	776.55 (111.71)	0.2	767.37 (105.43)
ITV (ml)	2664.73 (268.09)	2640.76 (262.91)	2594.77 (267.74)	0.094	2634.27 (267.54)
Plasma markers					
Aβ_42_ (pg/ml)	6.61 (1.95)	6.48 (1.78)	6.33 (2.13)	0.5	6.48 (1.97)
Aβ_status (%)	119 (83%)	88 (85%)	99 (77%)	0.3	306 (82%)
Aβ_40_ (pg/ml)	70.20 (44.30)	77.02 (40.59)	84.50 (44.28)	0.026	76.96 (43.62)
Aβ_42_/Aβ_40_ (pg/ml)	0.29 (0.64)	0.18 (0.21)	0.21 (0.51)	0.2	0.23 (0.51)
p-tau_181_ (pg/ml)	2.47 (1.76)	2.41 (1.57)	2.86 (1.79)	0.090	2.59 (1.73)
p-tau_181_/Aβ_42_ (pg/ml)	0.46 (0.52)	0.41 (0.32)	0.60 (0.89)	0.068	0.50 (0.64)
NFL (pg/ml)	17.72 (16.24)	16.58 (9.92)	23.17 (18.16)	0.002	19.27 (15.74)
GFAP (pg/ml)	104.09 (67.80)	112.51 (93.11)	118.32 (91.02)	0.4	111.26 (83.50)
Cognitive functions					
MMSE	26.72 (4.47)	26.01 (5.54)	25.42 (4.89)	0.094	26.08 (4.94)
MoCA	22.43 (5.46)	21.87 (6.33)	20.35 (5.66)	0.011	21.57 (5.83)
Episodic memory	0.07 (0.79)	0.04 (0.79)	−0.11 (0.72)	0.13	0.00 (0.77)
Processing speed	0.11 (0.88)	0.05 (0.91)	−0.17 (0.79)	0.022	0.00 (0.87)
Language function	0.08 (0.85)	0.13 (0.82)	−0.20 (0.84)	0.005	0.00 (0.85)
Executive function	0.13 (0.89)	0.03 (0.71)	−0.16 (0.60)	0.006	0.00 (0.76)
Visuospatial function	0.11 (0.86)	0.07 (0.70)	−0.18 (0.94)	0.011	0.00 (0.86)

CSVD, cerebral small vessel disease; No, CSVD score = 0; CSVD-I, mild CSVD (score = 1); CSVD-II, moderate to severe CSVD (score ≥ 2); GMV, whole-brain grey matter volume; WMV, whole-brain white matter volume; WMHV, white matter hyperintensity volume; CSFV, grey matter volume; ITV, intracranial volume; A*β*, amyloid beta; GFAP, glial fibrillary acidic protein; NfL, neurofilament light; MMSE, Mini-Mental State Examination; MoCA, Montreal Cognitive Assessment; SD, standard deviation.

^a^Mean (SD); *n* (%).

^b^One-way analysis of means; Pearson’s *χ*² test.

### Association between p-tau_181_ and NfL levels moderated by CSVD burden

**Figure 2:**
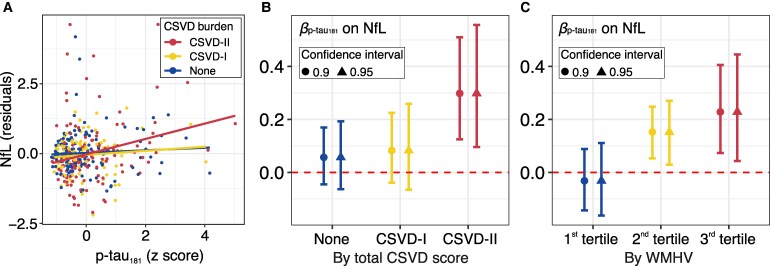
**Association between p-tau_181_ and NfL levels moderated by CSVD burden.** Multivariable linear regression models were used to assess the association between standardized plasma p-tau_181_ levels and NfL concentrations, including a p-tau_181_ × CSVD burden interaction, adjusting for age, sex, education, hypertension, diabetes, intracranial volume and Aβ positivity. (**A**) Covariate-adjusted NfL residuals plotted against p-tau_181_, stratified by CSVD burden grade, with fitted regression lines from the multivariable model; the interaction term was significant (est = 0.096, *P* = 0.018). *N* = 375 participants (no CSVD: *N* = 144; CSVD-I: *N* = 103; CSVD-II: *N* = 128), with each data point representing one participant. Panels **B** and **C** display standardized regression coefficients (*β*) for the effect of p-tau_181_ on NfL estimated from the same model, stratified by total CSVD score and white matter hyperintensity volume (WMHV) tertiles, respectively; error bars represent bootstrapped confidence intervals based on 5000 resamples (circles: 90% bias-corrected and accelerated confidence intervals; triangles: 95% confidence intervals). CSVD, cerebral small vessel disease; CSVD-I, mild CSVD pathology; CSVD-II, moderate to severe CSVD pathology; WMHV, white matter hyperintensity volume; NfL, neurofilament light; p-tau_181_, phosphorylated tau 181.

In the total sample, linear regression model suggested both significant relationship between p-tau_181_ and NfL levels (*β* = 0.197, *P* = 0.0051), as well as a significant interaction between p-tau_181_ and CSVD burden grade (est = 0.096, *P* = 0.018; Fig. 2A). This prompted a further assessment of the relationship between p-tau_181_ and NfL within each CSVD burden grade.

Stratified linear regressions suggested that in participants with moderate to severe CSVD burden, p-tau_181_ had a significant effect on NfL levels [*β* = 0.30, 90% CI = (0.13, 0.51); Fig. 2B], with this effect being significantly stronger compared to no CSVD [Δ*β* = 0.24, 90% CI = (0.052, 0.48)] and mild CSVD [Δ*β* = 0.22, 90% CI = (2.05e-3, 0.47)]. Similar findings were observed among participants in the high WMHV tertile (Fig. 2C) and were consistent with a more stringent 95% confidence interval (Supplementary Table 1). These results suggest a CSVD-moderated relationship between p-tau_181_ and NfL.

### Association between p-tau_181_ and WM integrity moderated by CSVD burden

**Figure 3:**
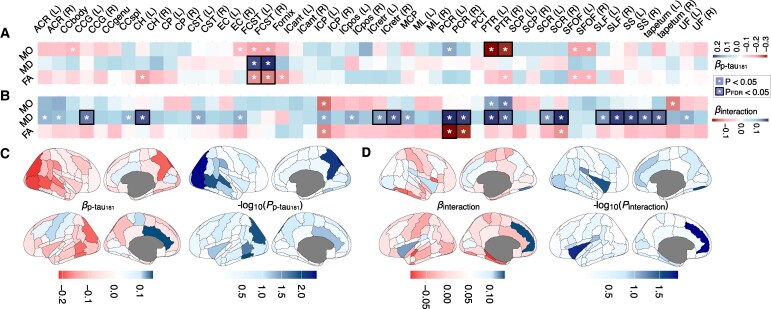
**Effects of plasma p-tau_181_ and its interaction with CSVD burden on white matter microstructure and regional grey matter volume.** Tract-wise white matter microstructural measures (**A** and **B**) and regional grey matter volumes (**C** and **D**) were analysed using multivariable linear regression models including plasma p-tau_181_, CSVD burden (no CSVD: *N* = 144; CSVD-I: *N* = 103; CSVD-II: *N* = 128) and their interaction, adjusting for age, sex, education, hypertension, diabetes, intracranial volume (for volumetric outcomes) and plasma Aβ status, with each participant constituting the experimental unit. Panel **A** shows standardized regression coefficients (*β*) for the main effect of p-tau_181_ on FA, MD and MO, while panel **B** shows *β* coefficients for the p-tau_181_ × CSVD burden interaction, with asterisks indicating *P* < 0.05 and black boxes indicating *P*_FDR_ < 0.05 across white matter tracts. Panels **C** and **D** display cortical maps of standardized *β* coefficients and corresponding −log_10_(*P*) values for the main and interaction effects, respectively. Heatmap colour scales represent standardized *β* coefficients and −log_10_ (*P*) values. p-tau_181_, phosphorylated tau 181; FA, fractional anisotropy; MD, mean diffusivity; MO, mode of anisotropy. Full abbreviations of white matter tracts are provided in Supplementary Table 10.

Our analyses revealed that p-tau_181_ exhibited significant associations with the microstructural integrity of multiple WM tracts. Notably, the MD and FA of the fornix cres and stria terminalis and posterior thalamic radiation were significantly related to p-tau_181_ levels (Fig. 3A, *P*_FDR_ < 0.05). Additionally, we found significant interactions between p-tau_181_ and CSVD burden on multiple WM tracts, particularly evident in the MD of the left cingulum (hippocampal part: est = 0.15, *P* = 0.00179, *P*_FDR_ = 0.027; cingulate gyrus part: est = 0.16, *P* = 0.0015, *P*_FDR_ = 0.015), bilateral retro-lenticular part of the internal capsule, posterior thalamic radiation, posterior corona radiata, splenium of the corpus callosum, superior longitudinal fasciculus, sagittal stratum and tapetum (Fig. 3B; Supplementary Table 2, *P*_FDR_ < 0.05).

In comparison to WM integrity, our analyses revealed much less significant effects of p-tau_181_ as well as its interaction of CSVD burden on regional GM volumes (Supplementary Table 3). We observed relationships between p-tau_181_ levels and the volumes of the parieto-occipital regions (Fig. 3C, *P* < 0.05), bilateral hippocampi (left: *β* = −0.21, *P* = 0.0037, *P*_FDR_ = 0.094; right: *β* = −0.21, *P* = 0.00379, *P*_FDR_ = 0.094) and amygdala (left: *β* = −0.16, *P* = 0.025, *P*_FDR_ = 0.19; right: *β* = −0.17, *P* = 0.015, *P*_FDR_ = 0.14). Significant interactions were found in the volumes of the insular cortex, left paracingulate gyrus and right frontal operculum cortex (Fig. 3D, *P* < 0.05; Supplementary Table 3). However, none of these effects in GM passed FDR correction. Overall, our analyses suggest substantial relationships between p-tau_181_ and WM tracts that might be moderated by CSVD burden.

### Association between p-tau_181_, WM tracts and cognitive function moderated by CSVD burden

**Figure 4:**
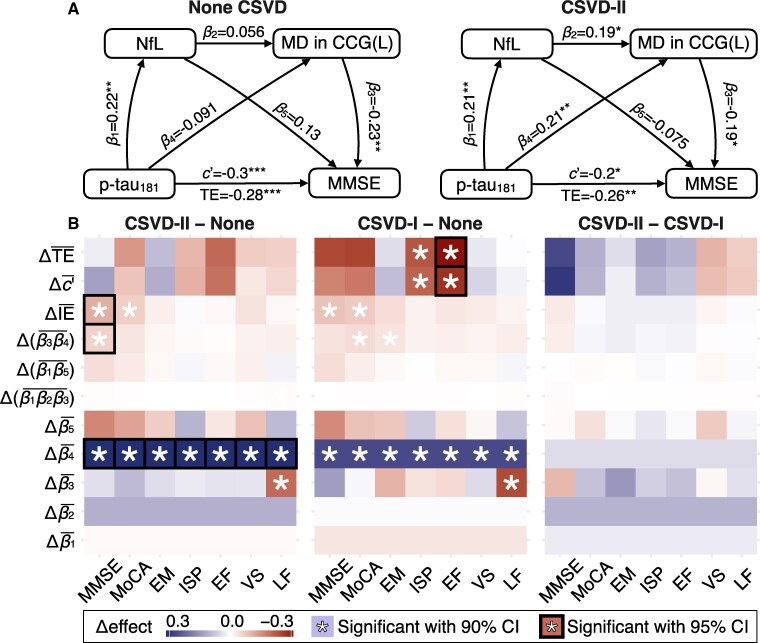
**Path analyses of the associations among plasma p-tau_181_, neurofilament light, left cingulum microstructure and cognitive performance across CSVD burden groups.** Path models specified that p-tau_181_ predicts NfL (*β*_1_), NfL and p-tau_181_ together predict MD in the left cingulum (cingulate gyrus part; *β*_2_ and *β*_4_) and cognitive outcomes are predicted by MD, NfL and p-tau_181_ (*β*_3_, *β*_5_, and *c*′), with TE representing the sum of direct and indirect effects. All coefficients are standardized and adjusted for age, sex, education, hypertension, diabetes, intracranial volume and A*β* status. (**A**) Estimated path coefficients within each CSVD burden group (no CSVD: *N* = 144; CSVD-I: *N* = 103; CSVD-II: *N* = 128), with each participant constituting the experimental unit. (**B**) Between-group differences in path coefficients and indirect effects (Δ), evaluated using 5000 bias-corrected and accelerated bootstrap resamples; colour scale indicates effect size differences, with asterisks denoting significance at 90% confidence intervals and black-boxed asterisks denoting significance at 95% confidence intervals. CSVD, cerebral small vessel disease; CSVD-I, mild CSVD pathology; CSVD-II, moderate to severe CSVD pathology; NfL, neurofilament light; p-tau_181_, phosphorylated tau 181; MD, mean diffusivity; CCG(L), left cingulum (cingulate gyrus part); MMSE, Mini-Mental State Examination; MoCA, Montreal Cognitive Assessment; EM, episodic memory; ISP, information processing speed; EF, executive function; VS, visuospatial function; LF, language function; IE, indirect effect; TE, total effect.

Through stratified path analyses, we identified CSVD-moderated associations between the MD of multiple WM tracts and p-tau_181_ (Supplementary Table 4), particularly in the left cingulum (cingulate gyrus part) (Fig. 4A) and the left tapetum (Supplementary Fig. 1). In the moderate to severe CSVD, the model showed a higher standardized coefficient between p-tau_181_ and MD in the left cingulum compared to the no CSVD [Δ$\bar{\beta_{4}}$ = 0.269, 90% CI = (0.0357, 0.495)]. Significant differences in the direct relationships between p-tau_181_ and both IPS [Δ$\bar{c\prime }$ = −0.212, 90% CI = (−0.413, −0.0194)] and EF [Δ$\bar{c\prime }$ = −0.276, 90% CI = (−0.583, −0.0524)] were also observed in mild CSVD compared to no CSVD (Fig. 4B).

Our analyses further revealed significant mediation of WM tract microstructural integrity on the effect of p-tau_181_ on cognitive functions in individuals with moderate to severe CSVD (Fig. 4B; Supplementary Table 5). Notably, the association between p-tau_181_ and MMSE was mediated by the MD in the left cingulum [CSVD-advanced: $\bar{\beta_{3}\beta_{4}}$ = −0.0394, 90% CI = (−0.11, −0.0101)] and was chain-mediated by NfL and left cingulum [$\bar{\beta_{1}\beta_{2}\beta_{3}}$ = −0.00645, 90% CI (−0.0304, −1.454e-03)]. These mediation effects were significantly stronger in participants with moderate to severe CSVD compared to no CSVD, as indicated by the difference in the total indirect effect of the association between p-tau_181_ and MMSE [Δ$\bar{IE}$ = −0.108, 90% CI = (−0.25, −0.0302)].

### Supplementary analyses

We replicated the analyses using WMHV tertiles as an indicator of CSVD burden. Consistent with our initial findings, we observed a stronger correlation between p-tau_181_ and NfL in participants with high WMHV (Fig. 2C; Supplementary Table 1). However, no significant interactions between p-tau_181_ and WMHV tertiles were observed in regional GM volumes (Supplementary Table 6) or WM tract-wise measures (Supplementary Fig. 2, *P*_FDR_ > 0.05).

To control for methodological bias that is sensitive to subcortical structures, we replicated the analyses using FIRST subcortical segmentation. The effect of p-tau_181_ on the volume of the right hippocampus remained significant (*β* = −0.23, *P* = 4.23e-04, *P*_FDR_ = 0.053), but no interactions were nominally significant (*P* > 0.05; Supplementary Table 7).

When using 95% CI, the difference in the relationship between p-tau_181_ and NfL among CSVD burden levels remained similar to the results obtained with 90% CI (Supplementary Table 1), except for the significance between moderate to severe and mild CSVD burden grades (Supplementary Fig. 3). The difference in the total indirect effect on the association between p-tau_181_ and cognition between moderate to severe CSVD and no CSVD remained significant with 95% CI (Fig. 4B; Supplementary Table 8).

We replaced p-tau_181_ with Aβ_42_ and found no significant effects of Aβ_42_ on NfL at any CSVD burden grade and no significant interactions with CSVD burden on NfL (Supplementary Fig. 4). While significant effects of Aβ_42_ on regional GM volumes (Supplementary Table 9) and tract-wise measures (Supplementary Fig. 5) were detected at a nominal 95% level, none of these results passed FDR correction. Only one significant interaction between Aβ_42_ and CSVD burden on WM tract was observed in the MO of the right cerebral peduncle (Supplementary Fig. 6).

When NfL was replaced with GFAP, a significant relationship between p-tau_181_ and GFAP was found only in the no CSVD group (Supplementary Fig. 7). These findings suggest that p-tau_181_ is more sensitive than Aβ_42_, and NfL is more sensitive than GFAP in detecting CSVD-related microstructural changes in WM. Overall, the results indicate that the relationship between p-tau_181_ and WM axonal injuries was robustly moderated by the CSVD burden.

## Discussion

This study demonstrated that CSVD burden and plasma biomarkers synergistically affect WM microstructure and cognitive function. Specifically, in individuals with a higher CSVD burden, plasma p-tau_181_ level was associated with NfL concentrations and WM microstructural integrity. Moreover, we identified a pathway whereby CSVD burden and p-tau_181_ influence cognition through NfL and WM damage within the cingulum. These findings suggest the synergistic interaction between vascular and neurodegenerative molecular pathologies in disrupting WM microstructure structure and accelerating cognitive impairment.

In individuals with a moderate to severe CSVD burden, we observed a stronger association between p-tau_181_ and NfL, suggesting that vascular pathology may exacerbate tau-related axonal degeneration. NfL is a key marker of axonal injury and has been associated with WM microstructural abnormalities and cognitive decline.^52,53^ The Vanderbilt Memory & Aging Project found that NfL was negatively associated with FA and positively associated with MD, axial diffusivity and radial diffusivity.^52^ Increased NfL levels reflect compromised axonal integrity.^54^ Our findings suggest that tau pathology may have a greater detrimental impact on axonal structures with vascular injury. This is consistent with previous findings that vascular dysfunction, including blood–brain barrier disruption and chronic microvascular ischaemia, enhances brain susceptibility to neurodegenerative processes by accumulating neurotoxic products, impairing metabolic support and promoting neuroinflammation.^55,56^ In an AAV-mediated tau overexpression mouse model, tau accumulation was shown to be accompanied by vascular structural alterations and inflammatory responses, ultimately resulting in axonal degeneration.^57^ Therefore, vascular impairment may lower the threshold for molecular injury, amplifying the detrimental effects of tau pathology on WM integrity.

We found that p-tau_181_ levels were significantly associated with the microstructural integrity of multiple WM tracts, including the fornix and stria terminalis and posterior thalamic radiation. These findings suggest that tau-related neurodegeneration affects WM pathways critical for cognitive function. In AD patients, increased p-tau specifically was related to reduced WM fibre cross-section.^58^ Tau phosphorylation may impair axonal transport and induce microglial activation, leading to WM disintegration.^59,60^ Furthermore, the WM tracts affected in our analysis are key components of memory and executive control networks. The fornix and stria terminalis serves as components of the limbic system, while the posterior thalamic radiation mediates information transfer between thalamic and cortical regions.^61-63^ Therefore, these WM tracts related to p-tau_181_ may constitute an important structural basis for cognitive impairment.

We found a significant interaction between CSVD burden and plasma p-tau_181_ levels on WM microstructure, specifically in the left cingulum. Although some researchers reported that AD pathology and CSVD exert independent and spatially different effects on WM microstructure, our findings suggest a synergistic interaction between two processes.^64-66^ A cross-sectional analysis found increased plasma and CSF tau levels and myelin loss in the WMH individuals and mouse ischaemia–reperfusion model.^67^ This suggests that beyond WMH, other cerebrovascular imaging markers may promote tau accumulation and its pathological effects, exacerbating WM microstructural damage.^56^ The cingulum bundle is an important region where the synergistic effects of vascular and neurodegenerative pathologies converge.^68^ Due to its complex vascular supply and anatomical proximity to regions vulnerable to early tau pathology, the cingulum may be susceptible to the combined vascular and neurodegenerative insults.^68,69^ The findings pay our attention to the synergistic effects of vascular and neurodegenerative molecular factors on WM microstructure integrity.

Our path analyses demonstrated that the CSVD burden and plasma p-tau_181_ levels jointly affected global cognition through elevated NfL concentrations and increased MD within the cingulum. This suggests a pathological cascade in which vascular and tau-related insults exacerbate axonal injury and further compromise cingulum structure and cognition. The cingulum is a key WM tract. Given its central role in interconnecting medial temporal, parietal and frontal areas involved in higher-order cognitive processing, the cingulum damage may particularly disrupt cognitive functions such as orientation, memory and verbal working memory.^68^ Similar studies have found microstructure alteration in the left cingulum cingulate gyrus was a direct determinant of the WMH-related Modified Boston Naming Test performance.^70^ In our study, microstructural disruptions in this region could mediate the adverse effects of upstream CSVD burden and p-tau_181_ accumulation on global cognition, especially LF. Notably, both NfL and cingulum microstructural changes served as mediators, providing pathways for vascular and molecular injuries to cognitive function.

To further clarify our findings, we additionally explored other plasma biomarkers, including Aβ_42_, and GFAP. The results revealed that Aβ_42_ and GFAP showed limited correlations with DTI metrics, while increased p-tau_181_ and NfL were strongly linked to increased MD and decreased FA in key WM tracts. In a community-dwelling cohort, plasma p-tau_181_ was significantly associated with WMH and FA in both unadjusted models and models adjusted for body mass index and estimated glomerular filtration rate.^71,72^ NfL was related to vascular WM scores in both low- and high-age groups.^73^ These findings suggest that p-tau_181_ and NfL may be more sensitive indicators of WM degeneration in the context of vascular burden.

This study has several limitations. First, its cross-sectional design precludes causal inference regarding the relationships among CSVD burden, plasma biomarkers and cognitive impairment. Second, we did not assess plasma p-tau_217_, which has shown superior performance than plasma p-tau_181_ in recent studies; future work incorporating p-tau_217_ is warranted.^19,74,75^ Third, the single-centre, relatively homogeneous Chinese elderly sample may limit generalizability, and replication in larger, multi-ethnic multi-centre cohorts is needed. Fourth, WMH and enlarged perivascular spaces are not entirely specific to CSVD and may partially reflect AD-related pathology.^26,76^ Although CSVD burden was rated by two trained physicians, automated quantitative approaches may improve precision and reproducibility. Finally, genetic factors such as the apolipoprotein E gene and polygenic AD risk were unavailable, limiting our ability to assess potential genetic modifiers of the observed associations.^77-79^

In conclusion, our study suggested that CSVD burden and plasma p-tau_181_ synergistically contribute to WM microstructural damage within the cingulum, which subsequently impairs cognitive performance. These findings emphasize the importance of integrating cerebrovascular neuroimaging and neurodegenerative molecular biomarkers to better understand the multifactorial mechanisms underlying cognitive impairment. Clinically, we should focus on the cerebrovascular health and plasma neurodegenerative biomarkers changes in patients with cognitive concerns. Future research should focus on exploring potential treatment strategies targeting both vascular and molecular pathways to delay cognitive decline in elderly individuals.

## Supplementary Material

fcag080_Supplementary_Data

## Data Availability

Data will be made available on request from the authors. The processing pipeline scripts are openly available in GitHub (https://github.com/xjx98/SynergisticEffectsofCSVDandAD).
