# Supplementary material for: Synergistic effects of cerebral small vessel disease burden and plasma phosphorylated tau 181 on white matter microstructure and cognition in a Chinese cohort
Source: Brain Commun. 2026 Mar 12;8(2):fcag080. doi: 10.1093/braincomms/fcag080 (PMC13022830; doi:10.1093/braincomms/fcag080)
Supplement: fcag080_Supplementary_Data [file fcag080_supplementary_data.pdf]

Synergistic Effects of Cerebral Small Vessel Disease Burden and Plasma Phosphorylated Tau 181 on White Matter Microstructure and Cognition in a Chinese Cohort

Supplementary Figures

FIGURE S1 2

FIGURE S2 3

FIGURE S3 4

FIGURE S4 5

FIGURE S5 6

FIGURE S6 7

FIGURE S7 8

Figure S1

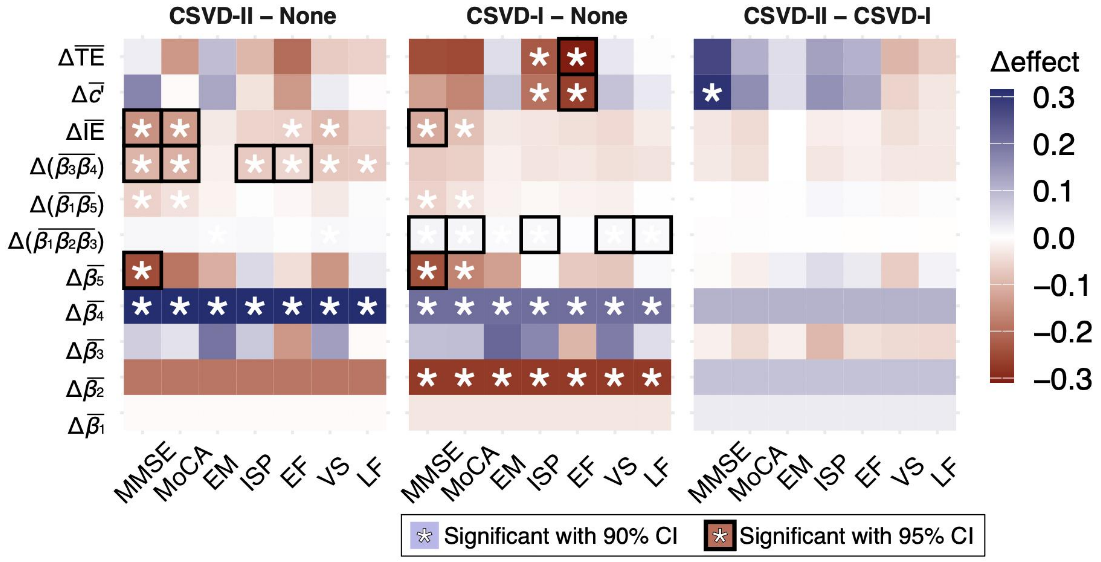

Figure S1. Path analyses of the associations among plasma p-tau$_{181}$, neurofilament light (NfL), left tapetum microstructure, and cognitive functions across CSVD burden grades. Path models were stratified by CSVD burden and specified that p-tau$_{181}$ predicts NfL, NfL and p-tau$_{181}$ together predict mean diffusivity in the left tapetum, and cognitive outcomes are predicted by imaging markers, NfL, and p-tau$_{181}$. Between-group differences in path coefficients and indirect effects were evaluated using 5,000 bias-corrected and accelerated bootstrap resamples with 90% confidence intervals. **N = 375 participants** (None CSVD: **N = 144**; CSVD-I: **N = 103**; CSVD-II: **N = 128**), with each participant constituting the experimental unit. Abbreviations: CSVD, cerebral small vessel disease; NfL, neurofilament light; MMSE, Mini-Mental State Examination; MoCA, Montreal Cognitive Assessment; EM, episodic memory; ISP, information processing speed; EF, executive function; VS, visuospatial function; LF, language function.

Figure S2

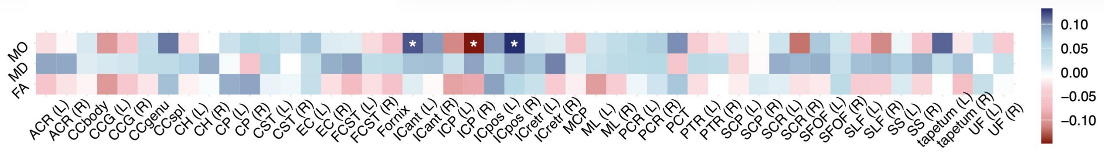

Figure S2. Interaction between plasma p-tau181 and white matter hyperintensity volume (WMHV) tertiles on white-matter tract microstructure. Tract-wise white-matter measures were analysed using multivariable linear regression models including plasma p-tau181, WMHV tertile, and their interaction, adjusting for age, sex, education, hypertension, diabetes, intracranial volume, and plasma Aβ status. Statistical significance of interaction terms was assessed using *t* tests from the regression models; asterisks indicate nominal significance ($P < 0.05$), and no effects survived false discovery rate (FDR) correction across tracts. Heatmap colour scale represents standardized regression coefficients (β, unitless). **N = 375 participants**, with each participant constituting the experimental unit. Abbreviations: WMHV, white matter hyperintensity volume; WM, white matter; FDR, false discovery rate. Full abbreviations are provided in Table S10.

Figure S3

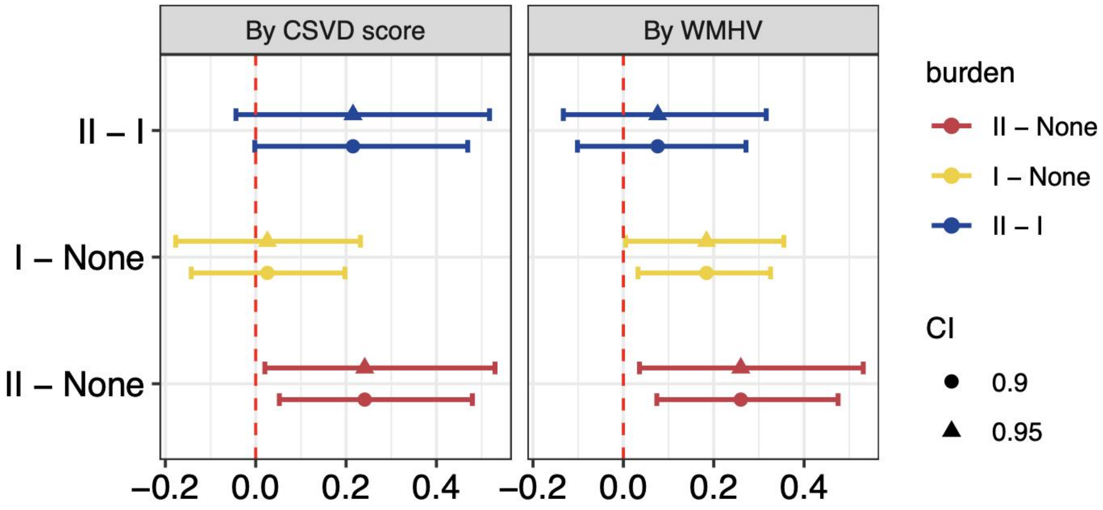

Figure S3. Supplementary analysis of differences in the association between plasma p-tau$_{181}$ and NfL across CSVD burden levels. Differences in standardized regression coefficients were evaluated using 5,000 bootstrap resamples with 90% and 95% confidence intervals. The x-axis indicates CSVD burden categories or WMHV tertiles, and the y-axis represents differences in standardized regression coefficients (Δβ, unitless). **N = 375 participants**, with each participant constituting the experimental unit. Abbreviations: CSVD, cerebral small vessel disease; NfL, neurofilament light; CI, confidence interval; WMHV, white matter hyperintensity volume.

Figure S4

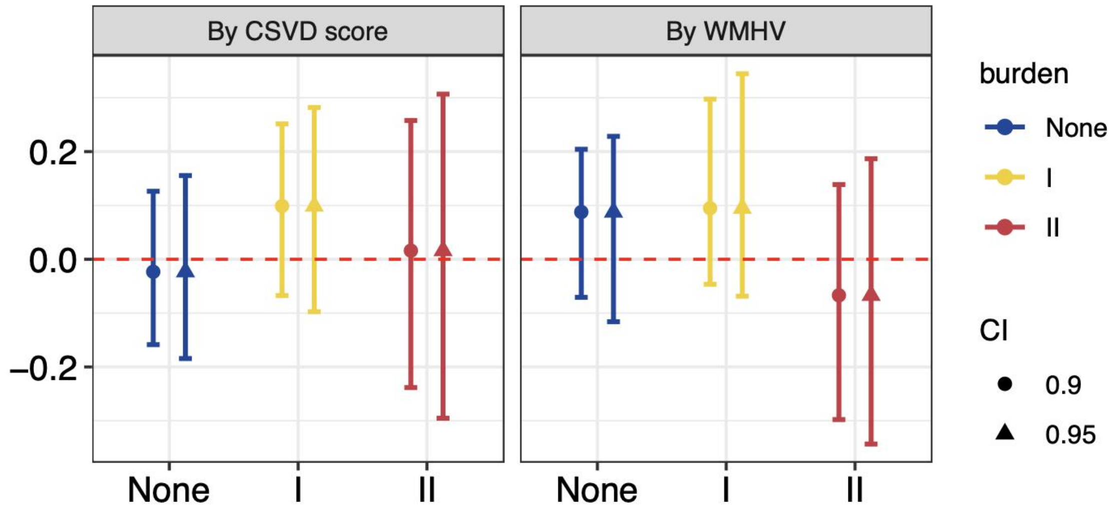

Figure S4. Association between plasma Aβ$_{42}$ and NfL levels moderated by CSVD burden. Multivariable linear regression models were used to assess the association between standardized plasma Aβ$_{42}$ levels and NfL concentrations, including an Aβ$_{42}$ × CSVD burden interaction, adjusting for age, sex, education, hypertension, diabetes, intracranial volume, and plasma Aβ status. The x-axis represents standardized Aβ$_{42}$ levels and the y-axis represents standardized NfL levels. **N = 375 participants**, with each participant constituting the experimental unit. Abbreviations: CSVD, cerebral small vessel disease; NfL, neurofilament light; WMHV, white matter hyperintensity volume; CI, confidence interval.

Figure S5

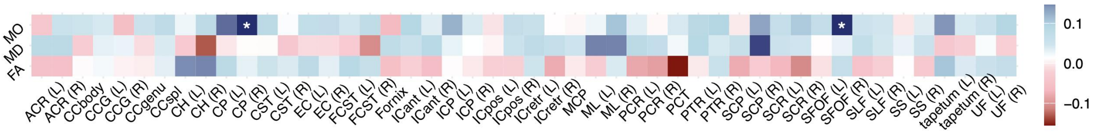

Figure S5. Effects of plasma Aβ_{42} on white-matter tract microstructure. Tract-wise white-matter measures were analysed using multivariable linear regression models adjusted for age, sex, education, hypertension, diabetes, intracranial volume, and plasma Aβ status. Statistical significance of regression coefficients was assessed using *t* tests; asterisks indicate nominal significance ($P < 0.05$), and no effects survived false discovery rate (FDR) correction across tracts. Heatmap colour scale represents standardized regression coefficients (β, unitless). **N = 375 participants**, with each participant constituting the experimental unit. Abbreviations: WM, white matter; FDR, false discovery rate. Full abbreviations are provided in **Table S10**.

Figure S6

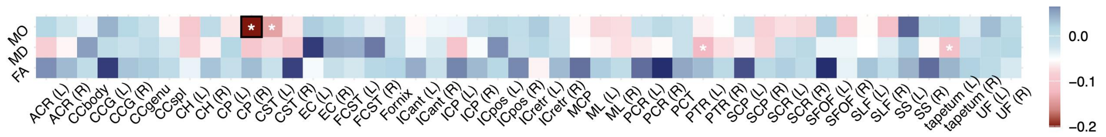

Figure S6. Interaction between plasma Aβ42 and CSVD burden on white-matter tract microstructure. Interaction effects were examined using multivariable linear regression models including plasma Aβ42, CSVD burden, and their interaction, adjusting for age, sex, education, hypertension, diabetes, intracranial volume, and plasma Aβ status. Statistical significance of interaction terms was assessed using *t* tests; asterisks indicate nominal significance ($P < 0.05$), and black-boxed cells denote effects surviving false discovery rate (FDR) correction. Heatmap colour scale represents standardized regression coefficients (β, unitless). **N = 375 participants**, with each participant constituting the experimental unit. Abbreviations: WM, white matter; FDR, false discovery rate. Full abbreviations are provided in Table S10.

Figure S7

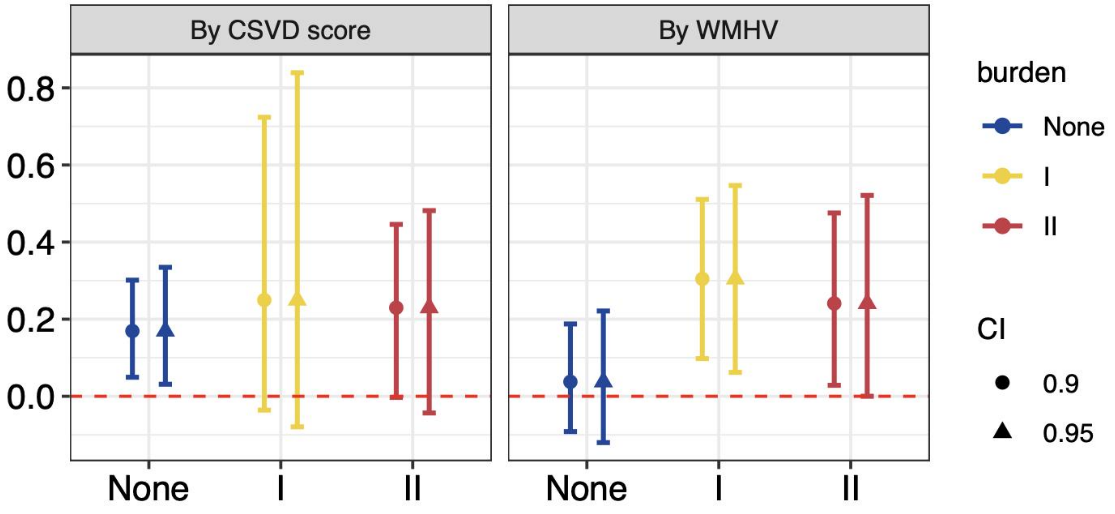

Figure S7. Association between plasma p-tau$_{181}$ and glial fibrillary acidic protein (GFAP) levels moderated by CSVD burden. Multivariable linear regression models were used to assess the association between standardized plasma p-tau$_{181}$ levels and GFAP concentrations, including a p-tau$_{181}$ × CSVD burden interaction, adjusting for age, sex, education, hypertension, diabetes, intracranial volume, and plasma Aβ status. The x-axis represents standardized p-tau$_{181}$ levels and the y-axis represents standardized GFAP levels. **N = 375 participants**, with each participant constituting the experimental unit. Abbreviations: CSVD, cerebral small vessel disease; GFAP, glial fibrillary acidic protein; WMHV, white matter hyperintensity volume; CI, confidence interval.

Synergistic Effects of Cerebral Small Vessel Disease Burden and Plasma Phosphorylated Tau 181 on White Matter Microstructure and Cognition in a Chinese Cohort

Supplementary Tables

Table S1 2
Table S2 3
Table S3 4
Table S4 6
Table S5 24
Table S6 26
Table S7 28
Table S8 29
Table S9 49
Table S10 52

Table S1

burden_type	burden	conf	effect
CSVD level	burden0	0.9	0.057 [-0.045, 0.17]
CSVD level	burden1	0.9	0.083 [-0.039, 0.23]
CSVD level	burden2	0.9	0.3 [0.13, 0.51]
CSVD level	diff_1_0	0.9	0.026 [-0.14, 0.2]
CSVD level	diff_2_0	0.9	0.24 [0.052, 0.48]
CSVD level	diff_2_1	0.9	0.22 [-2.05e-03, 0.47]
CSVD level	burden0	0.95	0.057 [-0.063, 0.19]
CSVD level	burden1	0.95	0.083 [-0.065, 0.26]
CSVD level	burden2	0.95	0.3 [0.096, 0.56]
CSVD level	diff_1_0	0.95	0.026 [-0.18, 0.23]
CSVD level	diff_2_0	0.95	0.24 [0.02, 0.53]
CSVD level	diff_2_1	0.95	0.22 [-0.044, 0.52]
WMHV level	burden0	0.9	-0.032 [-0.14, 0.088]
WMHV level	burden1	0.9	0.15 [0.053, 0.25]
WMHV level	burden2	0.9	0.23 [0.073, 0.41]
WMHV level	diff_1_0	0.9	0.18 [0.032, 0.33]
WMHV level	diff_2_0	0.9	0.26 [0.074, 0.48]
WMHV level	diff_2_1	0.9	0.076 [-0.1, 0.27]
WMHV level	burden0	0.95	-0.032 [-0.16, 0.11]
WMHV level	burden1	0.95	0.15 [0.029, 0.27]
WMHV level	burden2	0.95	0.23 [0.043, 0.44]
WMHV level	diff_1_0	0.95	0.18 [5.08e-03, 0.36]
WMHV level	diff_2_0	0.95	0.26 [0.036, 0.53]
WMHV level	diff_2_1	0.95	0.076 [-0.13, 0.32]

Table S2

description	effect of ptau181	effect of ptau181*CSVD burden
Mean FA in fornix on FA skeleton	β = -0.19, p = 6.19e-03, pFDR = 0.1	B = 0.073, p = 0.17, pFDR = 0.56
Mean FA in inferior cerebellar peduncle on FA skeleton (left)	β = -9.63e-03, p = 0.9, pFDR = 0.98	B = -0.13, p = 0.019, pFDR = 0.32
Mean FA in superior corona radiata on FA skeleton (right)	β = 0.12, p = 0.1, pFDR = 0.57	B = -0.12, p = 0.027, pFDR = 0.34
Mean FA in posterior corona radiata on FA skeleton (right)	β = 0.08, p = 0.27, pFDR = 0.77	B = -0.17, p = 2.02e-03, pFDR = 0.05
Mean FA in posterior corona radiata on FA skeleton (left)	β = 0.1, p = 0.15, pFDR = 0.65	B = -0.18, p = 8.49e-04, pFDR = 0.042
Mean FA in posterior thalamic radiation on FA skeleton (right)	β = -0.15, p = 0.028, pFDR = 0.2	B = -0.026, p = 0.62, pFDR = 0.89
Mean FA in cingulum hippocampus on FA skeleton (left)	β = -0.19, p = 8.84e-03, pFDR = 0.11	B = 5.78e-03, p = 0.92, pFDR = 0.98
Mean FA in fornix cres+stria terminalis on FA skeleton (right)	β = -0.24, p = 7.31e-04, pFDR = 0.037	B = 3.46e-03, p = 0.95, pFDR = 0.98
Mean FA in fornix cres+stria terminalis on FA skeleton (left)	β = -0.21, p = 1.58e-03, pFDR = 0.039	B = -0.011, p = 0.83, pFDR = 0.96
Mean FA in superior fronto-occipital fasciculus on FA skeleton (right)	β = -0.17, p = 0.019, pFDR = 0.16	B = 0.014, p = 0.8, pFDR = 0.96
Mean FA in superior fronto-occipital fasciculus on FA skeleton (left)	β = -0.17, p = 0.019, pFDR = 0.16	B = 0.031, p = 0.56, pFDR = 0.85
Mean MD in middle cerebellar peduncle on FA skeleton	β = -0.018, p = 0.8, pFDR = 0.97	B = 0.13, p = 0.018, pFDR = 0.057
Mean MD in splenium of corpus callosum on FA skeleton	β = 0.056, p = 0.42, pFDR = 0.97	B = 0.11, p = 0.037, pFDR = 0.077
Mean MD in corticospinal tract on FA skeleton (left)	β = -0.059, p = 0.42, pFDR = 0.97	B = 0.12, p = 0.029, pFDR = 0.07
Mean MD in inferior cerebellar peduncle on FA skeleton (left)	β = 0.027, p = 0.71, pFDR = 0.97	B = 0.12, p = 0.027, pFDR = 0.07
Mean MD in posterior limb of internal capsule on FA skeleton (left)	β = -0.13, p = 0.071, pFDR = 0.71	B = 0.12, p = 0.029, pFDR = 0.07
Mean MD in retrolenticular part of internal capsule on FA skeleton (right)	β = 6.03e-03, p = 0.93, pFDR = 0.98	B = 0.16, p = 3.08e-03, pFDR = 0.019
Mean MD in retrolenticular part of internal capsule on FA skeleton (left)	β = -0.033, p = 0.63, pFDR = 0.97	B = 0.14, p = 0.01, pFDR = 0.037
Mean MD in anterior corona radiata on FA skeleton (right)	β = 0.018, p = 0.79, pFDR = 0.97	B = 0.1, p = 0.036, pFDR = 0.077
Mean MD in anterior corona radiata on FA skeleton (left)	β = -3.15e-03, p = 0.96, pFDR = 0.98	B = 0.11, p = 0.026, pFDR = 0.07
Mean MD in superior corona radiata on FA skeleton (right)	β = -0.071, p = 0.29, pFDR = 0.97	B = 0.18, p = 4.69e-04, pFDR = 0.015
Mean MD in superior corona radiata on FA skeleton (left)	β = -0.061, p = 0.37, pFDR = 0.97	B = 0.14, p = 8.26e-03, pFDR = 0.032
Mean MD in posterior corona radiata on FA skeleton (right)	β = -0.036, p = 0.6, pFDR = 0.97	B = 0.17, p = 1.43e-03, pFDR = 0.015
Mean MD in posterior corona radiata on FA skeleton (left)	β = -0.033, p = 0.63, pFDR = 0.97	B = 0.18, p = 7.87e-04, pFDR = 0.015
Mean MD in posterior thalamic radiation on FA skeleton (right)	β = -1.42e-03, p = 0.98, pFDR = 0.98	B = 0.15, p = 3.71e-03, pFDR = 0.021
Mean MD in posterior thalamic radiation on FA skeleton (left)	β = -0.051, p = 0.47, pFDR = 0.97	B = 0.17, p = 1.37e-03, pFDR = 0.015
Mean MD in sagittal stratum on FA skeleton (right)	β = -0.015, p = 0.83, pFDR = 0.97	B = 0.15, p = 5.17e-03, pFDR = 0.026
Mean MD in sagittal stratum on FA skeleton (left)	β = -0.053, p = 0.46, pFDR = 0.97	B = 0.17, p = 2.10e-03, pFDR = 0.015
Mean MD in external capsule on FA skeleton (right)	β = -0.043, p = 0.52, pFDR = 0.97	B = 0.12, p = 0.023, pFDR = 0.066
Mean MD in cingulum cingulate gyrus on FA skeleton (left)	β = -0.034, p = 0.63, pFDR = 0.97	B = 0.15, p = 6.40e-03, pFDR = 0.027
Mean MD in cingulum hippocampus on FA skeleton (left)	β = 0.03, p = 0.65, pFDR = 0.97	B = 0.16, p = 1.53e-03, pFDR = 0.015
Mean MD in fornix cres+stria terminalis on FA skeleton (right)	β = 0.25, p = 2.36e-04, pFDR = 0.012	B = -0.012, p = 0.82, pFDR = 0.82
Mean MD in fornix cres+stria terminalis on FA skeleton (left)	β = 0.23, p = 7.95e-04, pFDR = 0.02	B = 0.022, p = 0.67, pFDR = 0.69
Mean MD in superior longitudinal fasciculus on FA skeleton (right)	β = -0.038, p = 0.6, pFDR = 0.97	B = 0.17, p = 1.79e-03, pFDR = 0.015
Mean MD in superior longitudinal fasciculus on FA skeleton (left)	β = -0.035, p = 0.63, pFDR = 0.97	B = 0.14, p = 0.013, pFDR = 0.043
Mean MD in uncinate fasciculus on FA skeleton (left)	β = -0.04, p = 0.58, pFDR = 0.97	B = 0.12, p = 0.037, pFDR = 0.077
Mean MD in tapetum on FA skeleton (left)	β = -8.65e-03, p = 0.91, pFDR = 0.98	B = 0.15, p = 6.00e-03, pFDR = 0.027
Mean MO in body of corpus callosum on FA skeleton	β = -0.15, p = 0.036, pFDR = 0.23	B = 0.043, p = 0.44, pFDR = 0.73
Mean MO in inferior cerebellar peduncle on FA skeleton (left)	β = 0.077, p = 0.3, pFDR = 0.75	B = -0.15, p = 0.011, pFDR = 0.3
Mean MO in posterior corona radiata on FA skeleton (left)	β = 0.16, p = 0.026, pFDR = 0.19	B = -6.34e-03, p = 0.91, pFDR = 0.95
Mean MO in posterior thalamic radiation on FA skeleton (right)	β = -0.29, p = 1.55e-04, pFDR = 3.87e-03	B = 0.15, p = 0.012, pFDR = 0.3
Mean MO in posterior thalamic radiation on FA skeleton (left)	β = -0.31, p = 5.90e-05, pFDR = 2.95e-03	B = 0.13, p = 0.03, pFDR = 0.37
Mean MO in external capsule on FA skeleton (right)	β = -0.15, p = 0.046, pFDR = 0.26	B = 0.051, p = 0.36, pFDR = 0.65
Mean MO in fornix cres+stria terminalis on FA skeleton (right)	β = -0.19, p = 0.013, pFDR = 0.14	B = 0.059, p = 0.3, pFDR = 0.65
Mean MO in fornix cres+stria terminalis on FA skeleton (left)	β = -0.19, p = 0.016, pFDR = 0.14	B = 0.072, p = 0.22, pFDR = 0.65
Mean MO in superior fronto-occipital fasciculus on FA skeleton (right)	β = -0.19, p = 0.017, pFDR = 0.14	B = 0.095, p = 0.11, pFDR = 0.65
Mean MO in superior fronto-occipital fasciculus on FA skeleton (left)	β = -0.19, p = 0.015, pFDR = 0.14	B = 0.055, p = 0.34, pFDR = 0.65
Mean MO in tapetum on FA skeleton (right)	β = -6.34e-03, p = 0.93, pFDR = 0.97	B = -0.14, p = 0.021, pFDR = 0.35

Table S3

description	effect of ptau181	effect of ptau181*CSVD burden
Volume of grey matter in Frontal Pole (left)	β = -0.075, p = 0.34, pFDR = 0.73	B = -8.81e-04, p = 0.99, pFDR = 1
Volume of grey matter in Frontal Pole (right)	β = -0.033, p = 0.67, pFDR = 0.88	B = 8.87e-03, p = 0.88, pFDR = 0.98
Volume of grey matter in Insular Cortex (left)	β = -0.15, p = 0.052, pFDR = 0.31	B = 0.12, p = 0.036, pFDR = 0.95
Volume of grey matter in Insular Cortex (right)	β = -0.12, p = 0.11, pFDR = 0.5	B = 0.11, p = 0.051, pFDR = 0.95
Volume of grey matter in Superior Frontal Gyrus (left)	β = -0.092, p = 0.25, pFDR = 0.71	B = -0.016, p = 0.79, pFDR = 0.98
Volume of grey matter in Superior Frontal Gyrus (right)	β = -0.04, p = 0.62, pFDR = 0.88	B = -6.52e-03, p = 0.91, pFDR = 0.98
Volume of grey matter in Middle Frontal Gyrus (left)	β = 0.073, p = 0.38, pFDR = 0.73	B = 0.032, p = 0.6, pFDR = 0.98
Volume of grey matter in Middle Frontal Gyrus (right)	β = -0.12, p = 0.15, pFDR = 0.59	B = 0.057, p = 0.34, pFDR = 0.95
Volume of grey matter in Inferior Frontal Gyrus, pars triangularis (left)	β = -0.11, p = 0.19, pFDR = 0.66	B = 0.025, p = 0.68, pFDR = 0.98
Volume of grey matter in Inferior Frontal Gyrus, pars triangularis (right)	β = -0.039, p = 0.63, pFDR = 0.88	B = -0.029, p = 0.63, pFDR = 0.98
Volume of grey matter in Inferior Frontal Gyrus, pars opercularis (left)	β = -0.034, p = 0.66, pFDR = 0.88	B = 0.063, p = 0.28, pFDR = 0.95
Volume of grey matter in Inferior Frontal Gyrus, pars opercularis (right)	β = -0.033, p = 0.68, pFDR = 0.88	B = 0.057, p = 0.33, pFDR = 0.95
Volume of grey matter in Precentral Gyrus (left)	β = 0.11, p = 0.13, pFDR = 0.54	B = -0.028, p = 0.6, pFDR = 0.98
Volume of grey matter in Precentral Gyrus (right)	β = -0.035, p = 0.64, pFDR = 0.88	B = -0.027, p = 0.64, pFDR = 0.98
Volume of grey matter in Temporal Pole (left)	β = -0.091, p = 0.25, pFDR = 0.71	B = 0.01, p = 0.86, pFDR = 0.98
Volume of grey matter in Temporal Pole (right)	β = -0.079, p = 0.32, pFDR = 0.73	B = 6.87e-03, p = 0.91, pFDR = 0.98
Volume of grey matter in Superior Temporal Gyrus, anterior division (left)	β = 5.78e-03, p = 0.94, pFDR = 0.97	B = -0.092, p = 0.13, pFDR = 0.95
Volume of grey matter in Superior Temporal Gyrus, anterior division (right)	β = -0.033, p = 0.69, pFDR = 0.88	B = -0.055, p = 0.36, pFDR = 0.95
Volume of grey matter in Superior Temporal Gyrus, posterior division (left)	β = -0.081, p = 0.32, pFDR = 0.73	B = -0.013, p = 0.82, pFDR = 0.98
Volume of grey matter in Superior Temporal Gyrus, posterior division (right)	β = -0.21, p = 9.18e-03, pFDR = 0.12	B = 0.084, p = 0.17, pFDR = 0.95
Volume of grey matter in Middle Temporal Gyrus, anterior division (left)	β = -0.096, p = 0.25, pFDR = 0.71	B = 0.02, p = 0.75, pFDR = 0.98
Volume of grey matter in Middle Temporal Gyrus, anterior division (right)	β = -0.039, p = 0.63, pFDR = 0.88	B = -0.04, p = 0.52, pFDR = 0.98
Volume of grey matter in Middle Temporal Gyrus, posterior division (left)	β = -0.11, p = 0.2, pFDR = 0.66	B = -0.05, p = 0.41, pFDR = 0.95
Volume of grey matter in Middle Temporal Gyrus, posterior division (right)	β = -0.16, p = 0.055, pFDR = 0.31	B = 7.35e-03, p = 0.9, pFDR = 0.98
Volume of grey matter in Middle Temporal Gyrus, temporooccipital part (left)	β = -0.19, p = 0.02, pFDR = 0.17	B = 8.63e-03, p = 0.89, pFDR = 0.98
Volume of grey matter in Middle Temporal Gyrus, temporooccipital part (right)	β = -0.2, p = 0.017, pFDR = 0.16	B = 0.059, p = 0.33, pFDR = 0.95
Volume of grey matter in Inferior Temporal Gyrus, anterior division (left)	β = 0.023, p = 0.78, pFDR = 0.93	B = -0.072, p = 0.23, pFDR = 0.95
Volume of grey matter in Inferior Temporal Gyrus, anterior division (right)	β = 0.036, p = 0.65, pFDR = 0.88	B = -0.081, p = 0.18, pFDR = 0.95
Volume of grey matter in Inferior Temporal Gyrus, posterior division (left)	β = -0.06, p = 0.47, pFDR = 0.83	B = 2.40e-03, p = 0.97, pFDR = 1
Volume of grey matter in Inferior Temporal Gyrus, posterior division (right)	β = -0.14, p = 0.078, pFDR = 0.39	B = 0.017, p = 0.78, pFDR = 0.98
Volume of grey matter in Inferior Temporal Gyrus, temporooccipital part (left)	β = -0.21, p = 9.42e-03, pFDR = 0.12	B = 0.061, p = 0.31, pFDR = 0.95
Volume of grey matter in Inferior Temporal Gyrus, temporooccipital part (right)	β = -0.09, p = 0.27, pFDR = 0.71	B = -0.075, p = 0.21, pFDR = 0.95
Volume of grey matter in Postcentral Gyrus (left)	β = 0.076, p = 0.28, pFDR = 0.71	B = -0.058, p = 0.28, pFDR = 0.95
Volume of grey matter in Postcentral Gyrus (right)	β = -0.019, p = 0.8, pFDR = 0.93	B = -0.051, p = 0.36, pFDR = 0.95
Volume of grey matter in Superior Parietal Lobule (left)	β = -0.041, p = 0.62, pFDR = 0.88	B = 9.39e-03, p = 0.88, pFDR = 0.98
Volume of grey matter in Superior Parietal Lobule (right)	β = -0.097, p = 0.23, pFDR = 0.7	B = 7.30e-03, p = 0.9, pFDR = 0.98
Volume of grey matter in Supramarginal Gyrus, anterior division (left)	β = -7.58e-03, p = 0.93, pFDR = 0.97	B = -9.42e-03, p = 0.88, pFDR = 0.98
Volume of grey matter in Supramarginal Gyrus, anterior division (right)	β = -0.17, p = 0.04, pFDR = 0.27	B = 0.02, p = 0.74, pFDR = 0.98
Volume of grey matter in Supramarginal Gyrus, posterior division (left)	β = -0.078, p = 0.34, pFDR = 0.73	B = -0.011, p = 0.86, pFDR = 0.98
Volume of grey matter in Supramarginal Gyrus, posterior division (right)	β = -0.16, p = 0.05, pFDR = 0.31	B = 0.071, p = 0.24, pFDR = 0.95
Volume of grey matter in Angular Gyrus (left)	β = -0.1, p = 0.23, pFDR = 0.7	B = 0.062, p = 0.31, pFDR = 0.95
Volume of grey matter in Angular Gyrus (right)	β = -0.12, p = 0.14, pFDR = 0.55	B = 0.044, p = 0.46, pFDR = 0.95
Volume of grey matter in Lateral Occipital Cortex, superior division (left)	β = -0.21, p = 6.19e-03, pFDR = 0.097	B = -4.00e-04, p = 0.99, pFDR = 1
Volume of grey matter in Lateral Occipital Cortex, superior division (right)	β = -0.24, p = 3.37e-03, pFDR = 0.07	B = 0.048, p = 0.42, pFDR = 0.95
Volume of grey matter in Lateral Occipital Cortex, inferior division (left)	β = -0.11, p = 0.16, pFDR = 0.59	B = -0.052, p = 0.39, pFDR = 0.95
Volume of grey matter in Lateral Occipital Cortex, inferior division (right)	β = -0.23, p = 3.23e-03, pFDR = 0.07	B = 0.086, p = 0.14, pFDR = 0.95
Volume of grey matter in Intracalcarine Cortex (left)	β = 0.027, p = 0.75, pFDR = 0.92	B = 0.068, p = 0.26, pFDR = 0.95
Volume of grey matter in Intracalcarine Cortex (right)	β = 0.079, p = 0.32, pFDR = 0.73	B = 0.024, p = 0.69, pFDR = 0.98
Volume of grey matter in Frontal Medial Cortex (left)	β = -7.74e-03, p = 0.92, pFDR = 0.97	B = -0.022, p = 0.72, pFDR = 0.98
Volume of grey matter in Frontal Medial Cortex (right)	β = -0.073, p = 0.36, pFDR = 0.73	B = 0.023, p = 0.69, pFDR = 0.98
Volume of grey matter in Juxtapositional Lobule Cortex (formerly Supplementary Motor Cortex) (left)	β = 0.019, p = 0.81, pFDR = 0.93	B = 6.05e-03, p = 0.92, pFDR = 0.98
Volume of grey matter in Juxtapositional Lobule Cortex (formerly Supplementary Motor Cortex) (right)	β = 0.048, p = 0.55, pFDR = 0.88	B = -0.046, p = 0.44, pFDR = 0.95
Volume of grey matter in Subcallosal Cortex (left)	β = -0.087, p = 0.29, pFDR = 0.71	B = 2.13e-04, p = 1, pFDR = 1
Volume of grey matter in Subcallosal Cortex (right)	β = -0.055, p = 0.5, pFDR = 0.84	B = 0.016, p = 0.79, pFDR = 0.98
Volume of grey matter in Paracingulate Gyrus (left)	β = -0.078, p = 0.34, pFDR = 0.73	B = 0.15, p = 0.011, pFDR = 0.95
Volume of grey matter in Paracingulate Gyrus (right)	β = -0.038, p = 0.64, pFDR = 0.88	B = 0.094, p = 0.12, pFDR = 0.95
Volume of grey matter in Cingulate Gyrus, anterior division (left)	β = 0.15, p = 0.076, pFDR = 0.39	B = -0.023, p = 0.7, pFDR = 0.98
Volume of grey matter in Cingulate Gyrus, anterior division (right)	β = 0.057, p = 0.48, pFDR = 0.83	B = 0.081, p = 0.18, pFDR = 0.95
Volume of grey matter in Cingulate Gyrus, posterior division (left)	β = -0.056, p = 0.5, pFDR = 0.84	B = 0.023, p = 0.7, pFDR = 0.98
Volume of grey matter in Cingulate Gyrus, posterior division (right)	β = -0.072, p = 0.37, pFDR = 0.73	B = -5.71e-03, p = 0.92, pFDR = 0.98
Volume of grey matter in Precuneous Cortex (left)	β = -0.13, p = 0.1, pFDR = 0.47	B = -9.32e-03, p = 0.88, pFDR = 0.98
Volume of grey matter in Precuneous Cortex (right)	β = -0.22, p = 5.20e-03, pFDR = 0.093	B = 0.066, p = 0.27, pFDR = 0.95
Volume of grey matter in Cuneal Cortex (left)	β = -0.064, p = 0.43, pFDR = 0.8	B = 0.05, p = 0.41, pFDR = 0.95
Volume of grey matter in Cuneal Cortex (right)	β = -0.062, p = 0.45, pFDR = 0.81	B = 0.055, p = 0.36, pFDR = 0.95
Volume of grey matter in Frontal Orbital Cortex (left)	β = -0.013, p = 0.87, pFDR = 0.94	B = -0.035, p = 0.56, pFDR = 0.98
Volume of grey matter in Frontal Orbital Cortex (right)	β = -0.018, p = 0.82, pFDR = 0.93	B = 0.016, p = 0.79, pFDR = 0.98
Volume of grey matter in Parahippocampal Gyrus, anterior division (left)	β = -0.036, p = 0.65, pFDR = 0.88	B = -0.094, p = 0.11, pFDR = 0.95
Volume of grey matter in Parahippocampal Gyrus, anterior division (right)	β = -0.033, p = 0.67, pFDR = 0.88	B = -0.015, p = 0.79, pFDR = 0.98
Volume of grey matter in Parahippocampal Gyrus, posterior division (left)	β = 0.018, p = 0.82, pFDR = 0.93	B = -0.1, p = 0.09, pFDR = 0.95
Volume of grey matter in Parahippocampal Gyrus, posterior division (right)	β = -0.047, p = 0.53, pFDR = 0.86	B = 0.02, p = 0.71, pFDR = 0.98
Volume of grey matter in Lingual Gyrus (left)	β = 0.025, p = 0.75, pFDR = 0.92	B = -3.58e-03, p = 0.95, pFDR = 0.99
Volume of grey matter in Lingual Gyrus (right)	β = 0.015, p = 0.85, pFDR = 0.94	B = 0.014, p = 0.82, pFDR = 0.98
Volume of grey matter in Temporal Fusiform Cortex, anterior division (left)	β = 0.054, p = 0.5, pFDR = 0.84	B = -0.062, p = 0.3, pFDR = 0.95
Volume of grey matter in Temporal Fusiform Cortex, anterior division (right)	β = 0.065, p = 0.42, pFDR = 0.79	B = -0.081, p = 0.18, pFDR = 0.95
Volume of grey matter in Temporal Fusiform Cortex, posterior division (left)	β = 0.029, p = 0.72, pFDR = 0.91	B = -0.077, p = 0.2, pFDR = 0.95
Volume of grey matter in Temporal Fusiform Cortex, posterior division (right)	β = -4.48e-03, p = 0.96, pFDR = 0.97	B = -0.027, p = 0.65, pFDR = 0.98
Volume of grey matter in Temporal Occipital Fusiform Cortex (left)	β = -0.075, p = 0.36, pFDR = 0.73	B = 0.03, p = 0.62, pFDR = 0.98
Volume of grey matter in Temporal Occipital Fusiform Cortex (right)	β = -0.17, p = 0.041, pFDR = 0.27	B = 0.061, p = 0.31, pFDR = 0.95
Volume of grey matter in Occipital Fusiform Gyrus (left)	β = 0.043, p = 0.58, pFDR = 0.88	B = -0.07, p = 0.23, pFDR = 0.95
Volume of grey matter in Occipital Fusiform Gyrus (right)	β = -0.011, p = 0.88, pFDR = 0.94	B = -0.081, p = 0.16, pFDR = 0.95
Volume of grey matter in Frontal Operculum Cortex (left)	β = -0.033, p = 0.68, pFDR = 0.88	B = 0.075, p = 0.21, pFDR = 0.95
Volume of grey matter in Frontal Operculum Cortex (right)	β = -0.13, p = 0.1, pFDR = 0.47	B = 0.12, p = 0.043, pFDR = 0.95
Volume of grey matter in Central Opercular Cortex (left)	β = -0.017, p = 0.83, pFDR = 0.93	B = 0.044, p = 0.47, pFDR = 0.95
Volume of grey matter in Central Opercular Cortex (right)	β = -0.031, p = 0.71, pFDR = 0.9	B = 7.14e-03, p = 0.91, pFDR = 0.98
Volume of grey matter in Parietal Operculum Cortex (left)	β = -0.022, p = 0.79, pFDR = 0.93	B = 0.096, p = 0.11, pFDR = 0.95
Volume of grey matter in Parietal Operculum Cortex (right)	β = -0.11, p = 0.2, pFDR = 0.66	B = 0.11, p = 0.08, pFDR = 0.95
Volume of grey matter in Planum Polare (left)	β = 0.074, p = 0.36, pFDR = 0.73	B = -0.049, p = 0.41, pFDR = 0.95
Volume of grey matter in Planum Polare (right)	β = 0.041, p = 0.6, pFDR = 0.88	B = -0.043, p = 0.46, pFDR = 0.95
Volume of grey matter in Heschl's Gyrus (includes H1 and H2) (left)	β = 0.093, p = 0.23, pFDR = 0.7	B = -0.029, p = 0.62, pFDR = 0.98
Volume of grey matter in Heschl's Gyrus (includes H1 and H2) (right)	β = 4.55e-03, p = 0.95, pFDR = 0.97	B = -0.014, p = 0.81, pFDR = 0.98
Volume of grey matter in Planum Temporale (left)	β = -0.026, p = 0.75, pFDR = 0.92	B = 0.058, p = 0.34, pFDR = 0.95
Volume of grey matter in Planum Temporale (right)	β = -0.066, p = 0.42, pFDR = 0.79	B = 0.042, p = 0.49, pFDR = 0.97
Volume of grey matter in Supracalcarine Cortex (left)	β = -0.041, p = 0.62, pFDR = 0.88	B = 0.058, p = 0.34, pFDR = 0.95
Volume of grey matter in Supracalcarine Cortex (right)	β = 0.028, p = 0.73, pFDR = 0.91	B = 0.041, p = 0.5, pFDR = 0.97
Volume of grey matter in Occipital Pole (left)	β = -0.086, p = 0.27, pFDR = 0.71	B = -0.031, p = 0.6, pFDR = 0.98
Volume of grey matter in Occipital Pole (right)	β = -0.14, p = 0.065, pFDR = 0.36	B = 0.053, p = 0.35, pFDR = 0.95
Volume of grey matter in Thalamus (left)	β = 0.056, p = 0.47, pFDR = 0.83	B = 0.03, p = 0.6, pFDR = 0.98
Volume of grey matter in Thalamus (right)	β = 0.082, p = 0.28, pFDR = 0.71	B = -0.034, p = 0.55, pFDR = 0.98
Volume of grey matter in Caudate (left)	β = 0.042, p = 0.57, pFDR = 0.88	B = 0.026, p = 0.63, pFDR = 0.98
Volume of grey matter in Caudate (right)	β = 0.015, p = 0.84, pFDR = 0.93	B = 0.044, p = 0.43, pFDR = 0.95
Volume of grey matter in Putamen (left)	β = -0.021, p = 0.78, pFDR = 0.93	B = -0.053, p = 0.34, pFDR = 0.95
Volume of grey matter in Putamen (right)	β = -0.069, p = 0.36, pFDR = 0.73	B = 0.018, p = 0.75, pFDR = 0.98
Volume of grey matter in Pallidum (left)	β = 0.074, p = 0.35, pFDR = 0.73	B = 0.056, p = 0.34, pFDR = 0.95
Volume of grey matter in Pallidum (right)	β = 0.12, p = 0.12, pFDR = 0.51	B = 0.044, p = 0.45, pFDR = 0.95
Volume of grey matter in Hippocampus (left)	β = -0.21, p = 3.05e-03, pFDR = 0.07	B = -0.039, p = 0.46, pFDR = 0.95
Volume of grey matter in Hippocampus (right)	β = -0.22, p = 2.43e-03, pFDR = 0.07	B = 1.24e-03, p = 0.98, pFDR = 1
Volume of grey matter in Amygdala (left)	β = -0.17, p = 0.021, pFDR = 0.17	B = 0.013, p = 0.81, pFDR = 0.98
Volume of grey matter in Amygdala (right)	β = -0.18, p = 0.012, pFDR = 0.12	B = 0.029, p = 0.59, pFDR = 0.98
Volume of grey matter in Ventral Striatum (left)	β = -1.76e-03, p = 0.98, pFDR = 0.98	B = -0.02, p = 0.72, pFDR = 0.98
Volume of grey matter in Ventral Striatum (right)	β = -0.013, p = 0.87, pFDR = 0.94	B = 0.033, p = 0.58, pFDR = 0.98
Volume of grey matter in Frontal Pole (left)	β = -0.062, p = 0.4, pFDR = 0.8	B = 0.012, p = 0.83, pFDR = 0.97
Volume of grey matter in Frontal Pole (right)	β = -0.021, p = 0.77, pFDR = 0.91	B = 0.014, p = 0.8, pFDR = 0.97
Volume of grey matter in Insular Cortex (left)	β = -0.13, p = 0.058, pFDR = 0.34	B = 0.12, p = 0.018, pFDR = 0.96

**Volume of grey matter in Insular Cortex (right)**	β = -0.098, p = 0.14, pFDR = 0.6	B = 0.11, p = 0.031, pFDR = 0.96
**Volume of grey matter in Superior Frontal Gyrus (left)**	β = -0.092, p = 0.22, pFDR = 0.69	B = -5.58e-04, p = 0.99, pFDR = 0.99
**Volume of grey matter in Superior Frontal Gyrus (right)**	β = -0.053, p = 0.48, pFDR = 0.85	B = 0.014, p = 0.81, pFDR = 0.97
**Volume of grey matter in Middle Frontal Gyrus (left)**	β = 0.065, p = 0.4, pFDR = 0.8	B = 0.04, p = 0.49, pFDR = 0.97
**Volume of grey matter in Middle Frontal Gyrus (right)**	β = -0.1, p = 0.16, pFDR = 0.63	B = 0.062, p = 0.26, pFDR = 0.97
**Volume of grey matter in Inferior Frontal Gyrus, pars triangularis (left)**	β = -0.098, p = 0.22, pFDR = 0.69	B = 0.028, p = 0.64, pFDR = 0.97
**Volume of grey matter in Inferior Frontal Gyrus, pars triangularis (right)**	β = -0.035, p = 0.67, pFDR = 0.89	B = -0.02, p = 0.73, pFDR = 0.97
**Volume of grey matter in Inferior Frontal Gyrus, pars opercularis (left)**	β = -0.018, p = 0.82, pFDR = 0.93	B = 0.058, p = 0.34, pFDR = 0.97
**Volume of grey matter in Inferior Frontal Gyrus, pars opercularis (right)**	β = -0.036, p = 0.65, pFDR = 0.89	B = 0.064, p = 0.27, pFDR = 0.97
**Volume of grey matter in Precentral Gyrus (left)**	β = 0.11, p = 0.15, pFDR = 0.61	B = -0.013, p = 0.81, pFDR = 0.97
**Volume of grey matter in Precentral Gyrus (right)**	β = -0.036, p = 0.62, pFDR = 0.89	B = -0.011, p = 0.84, pFDR = 0.97
**Volume of grey matter in Temporal Pole (left)**	β = -0.091, p = 0.24, pFDR = 0.7	B = 0.021, p = 0.71, pFDR = 0.97
**Volume of grey matter in Temporal Pole (right)**	β = -0.071, p = 0.37, pFDR = 0.8	B = 0.014, p = 0.81, pFDR = 0.97
**Volume of grey matter in Superior Temporal Gyrus, anterior division (left)**	β = 1.06e-03, p = 0.99, pFDR = 0.99	B = -0.081, p = 0.16, pFDR = 0.97
**Volume of grey matter in Superior Temporal Gyrus, anterior division (right)**	β = -0.031, p = 0.69, pFDR = 0.89	B = -0.047, p = 0.4, pFDR = 0.97
**Volume of grey matter in Superior Temporal Gyrus, posterior division (left)**	β = -0.073, p = 0.34, pFDR = 0.78	B = -0.012, p = 0.83, pFDR = 0.97
**Volume of grey matter in Superior Temporal Gyrus, posterior division (right)**	β = -0.18, p = 0.013, pFDR = 0.14	B = 0.079, p = 0.14, pFDR = 0.97
**Volume of grey matter in Middle Temporal Gyrus, anterior division (left)**	β = -0.082, p = 0.29, pFDR = 0.71	B = 0.022, p = 0.7, pFDR = 0.97
**Volume of grey matter in Middle Temporal Gyrus, anterior division (right)**	β = -0.038, p = 0.63, pFDR = 0.89	B = -0.03, p = 0.62, pFDR = 0.97
**Volume of grey matter in Middle Temporal Gyrus, posterior division (left)**	β = -0.1, p = 0.19, pFDR = 0.67	B = -0.037, p = 0.52, pFDR = 0.97
**Volume of grey matter in Middle Temporal Gyrus, posterior division (right)**	β = -0.14, p = 0.069, pFDR = 0.36	B = 9.04e-03, p = 0.87, pFDR = 0.97
**Volume of grey matter in Middle Temporal Gyrus, temporooccipital part (left)**	β = -0.18, p = 0.021, pFDR = 0.18	B = 0.014, p = 0.81, pFDR = 0.97
**Volume of grey matter in Middle Temporal Gyrus, temporooccipital part (right)**	β = -0.2, p = 0.012, pFDR = 0.14	B = 0.063, p = 0.28, pFDR = 0.97
**Volume of grey matter in Inferior Temporal Gyrus, anterior division (left)**	β = 0.034, p = 0.66, pFDR = 0.89	B = -0.065, p = 0.26, pFDR = 0.97
**Volume of grey matter in Inferior Temporal Gyrus, anterior division (right)**	β = 0.029, p = 0.72, pFDR = 0.89	B = -0.062, p = 0.29, pFDR = 0.97
**Volume of grey matter in Inferior Temporal Gyrus, posterior division (left)**	β = -0.045, p = 0.55, pFDR = 0.89	B = 6.71e-03, p = 0.91, pFDR = 0.97
**Volume of grey matter in Inferior Temporal Gyrus, posterior division (right)**	β = -0.14, p = 0.076, pFDR = 0.38	B = 0.022, p = 0.7, pFDR = 0.97
**Volume of grey matter in Inferior Temporal Gyrus, temporooccipital part (left)**	β = -0.19, p = 0.01, pFDR = 0.14	B = 0.059, p = 0.27, pFDR = 0.97
**Volume of grey matter in Inferior Temporal Gyrus, temporooccipital part (right)**	β = -0.078, p = 0.3, pFDR = 0.71	B = -0.07, p = 0.21, pFDR = 0.97
**Volume of grey matter in Postcentral Gyrus (left)**	β = 0.071, p = 0.33, pFDR = 0.77	B = -0.043, p = 0.43, pFDR = 0.97
**Volume of grey matter in Postcentral Gyrus (right)**	β = -0.019, p = 0.79, pFDR = 0.92	B = -0.037, p = 0.5, pFDR = 0.97
**Volume of grey matter in Superior Parietal Lobule (left)**	β = -0.04, p = 0.61, pFDR = 0.89	B = 0.017, p = 0.78, pFDR = 0.97
**Volume of grey matter in Superior Parietal Lobule (right)**	β = -0.097, p = 0.23, pFDR = 0.69	B = 0.014, p = 0.81, pFDR = 0.97
**Volume of grey matter in Supramarginal Gyrus, anterior division (left)**	β = -2.64e-03, p = 0.97, pFDR = 0.98	B = -3.18e-03, p = 0.96, pFDR = 0.98
**Volume of grey matter in Supramarginal Gyrus, anterior division (right)**	β = -0.16, p = 0.04, pFDR = 0.3	B = 0.025, p = 0.68, pFDR = 0.97
**Volume of grey matter in Supramarginal Gyrus, posterior division (left)**	β = -0.081, p = 0.29, pFDR = 0.71	B = 4.78e-03, p = 0.93, pFDR = 0.97
**Volume of grey matter in Supramarginal Gyrus, posterior division (right)**	β = -0.15, p = 0.063, pFDR = 0.34	B = 0.072, p = 0.23, pFDR = 0.97
**Volume of grey matter in Angular Gyrus (left)**	β = -0.1, p = 0.2, pFDR = 0.67	B = 0.068, p = 0.25, pFDR = 0.97
**Volume of grey matter in Angular Gyrus (right)**	β = -0.11, p = 0.17, pFDR = 0.63	B = 0.05, p = 0.4, pFDR = 0.97
**Volume of grey matter in Lateral Occipital Cortex, superior division (left)**	β = -0.19, p = 0.012, pFDR = 0.14	B = 4.02e-03, p = 0.94, pFDR = 0.97
**Volume of grey matter in Lateral Occipital Cortex, superior division (right)**	β = -0.21, p = 4.23e-03, pFDR = 0.094	B = 0.052, p = 0.35, pFDR = 0.97
**Volume of grey matter in Lateral Occipital Cortex, inferior division (left)**	β = -0.1, p = 0.16, pFDR = 0.63	B = -0.038, p = 0.49, pFDR = 0.97
**Volume of grey matter in Lateral Occipital Cortex, inferior division (right)**	β = -0.21, p = 4.52e-03, pFDR = 0.094	B = 0.085, p = 0.12, pFDR = 0.97
**Volume of grey matter in Intracalcarine Cortex (left)**	β = 0.026, p = 0.74, pFDR = 0.89	B = 0.073, p = 0.2, pFDR = 0.97
**Volume of grey matter in Intracalcarine Cortex (right)**	β = 0.083, p = 0.28, pFDR = 0.71	B = 0.024, p = 0.67, pFDR = 0.97
**Volume of grey matter in Frontal Medial Cortex (left)**	β = -0.013, p = 0.87, pFDR = 0.94	B = -6.55e-03, p = 0.91, pFDR = 0.97
**Volume of grey matter in Frontal Medial Cortex (right)**	β = -0.069, p = 0.37, pFDR = 0.8	B = 0.033, p = 0.57, pFDR = 0.97
**Volume of grey matter in Juxtapositional Lobule Cortex (formerly Supplementary Motor Cortex) (left)**	β = 5.27e-03, p = 0.95, pFDR = 0.97	B = 0.018, p = 0.75, pFDR = 0.97
**Volume of grey matter in Juxtapositional Lobule Cortex (formerly Supplementary Motor Cortex) (right)**	β = 0.035, p = 0.65, pFDR = 0.89	B = -0.032, p = 0.57, pFDR = 0.97
**Volume of grey matter in Subcallosal Cortex (left)**	β = -0.06, p = 0.36, pFDR = 0.8	B = 7.53e-03, p = 0.88, pFDR = 0.97
**Volume of grey matter in Subcallosal Cortex (right)**	β = -0.028, p = 0.67, pFDR = 0.89	B = 0.018, p = 0.71, pFDR = 0.97
**Volume of grey matter in Paracingulate Gyrus (left)**	β = -0.056, p = 0.44, pFDR = 0.83	B = 0.13, p = 0.014, pFDR = 0.96
**Volume of grey matter in Paracingulate Gyrus (right)**	β = -0.023, p = 0.76, pFDR = 0.9	B = 0.087, p = 0.11, pFDR = 0.97
**Volume of grey matter in Cingulate Gyrus, anterior division (left)**	β = 0.15, p = 0.054, pFDR = 0.34	B = -0.025, p = 0.66, pFDR = 0.97
**Volume of grey matter in Cingulate Gyrus, anterior division (right)**	β = 0.072, p = 0.34, pFDR = 0.78	B = 0.065, p = 0.25, pFDR = 0.97
**Volume of grey matter in Cingulate Gyrus, posterior division (left)**	β = -0.033, p = 0.62, pFDR = 0.89	B = 0.022, p = 0.66, pFDR = 0.97
**Volume of grey matter in Cingulate Gyrus, posterior division (right)**	β = -0.053, p = 0.44, pFDR = 0.83	B = 4.19e-03, p = 0.93, pFDR = 0.97
**Volume of grey matter in Precuneous Cortex (left)**	β = -0.11, p = 0.12, pFDR = 0.53	B = 3.95e-03, p = 0.94, pFDR = 0.97
**Volume of grey matter in Precuneous Cortex (right)**	β = -0.19, p = 7.77e-03, pFDR = 0.14	B = 0.066, p = 0.21, pFDR = 0.97
**Volume of grey matter in Cuneal Cortex (left)**	β = -0.055, p = 0.46, pFDR = 0.83	B = 0.054, p = 0.33, pFDR = 0.97
**Volume of grey matter in Cuneal Cortex (right)**	β = -0.043, p = 0.57, pFDR = 0.89	B = 0.051, p = 0.36, pFDR = 0.97
**Volume of grey matter in Frontal Orbital Cortex (left)**	β = -0.013, p = 0.86, pFDR = 0.94	B = -0.015, p = 0.78, pFDR = 0.97
**Volume of grey matter in Frontal Orbital Cortex (right)**	β = -7.34e-03, p = 0.92, pFDR = 0.97	B = 0.024, p = 0.66, pFDR = 0.97
**Volume of grey matter in Parahippocampal Gyrus, anterior division (left)**	β = -0.047, p = 0.53, pFDR = 0.89	B = -0.071, p = 0.19, pFDR = 0.97
**Volume of grey matter in Parahippocampal Gyrus, anterior division (right)**	β = -0.028, p = 0.7, pFDR = 0.89	B = -7.41e-03, p = 0.89, pFDR = 0.97
**Volume of grey matter in Parahippocampal Gyrus, posterior division (left)**	β = 0.014, p = 0.86, pFDR = 0.94	B = -0.087, p = 0.13, pFDR = 0.97
**Volume of grey matter in Parahippocampal Gyrus, posterior division (right)**	β = -0.049, p = 0.53, pFDR = 0.89	B = 0.034, p = 0.56, pFDR = 0.97
**Volume of grey matter in Lingual Gyrus (left)**	β = 0.037, p = 0.61, pFDR = 0.89	B = -8.72e-04, p = 0.99, pFDR = 0.99
**Volume of grey matter in Lingual Gyrus (right)**	β = 0.024, p = 0.74, pFDR = 0.89	B = 0.012, p = 0.82, pFDR = 0.97
**Volume of grey matter in Temporal Fusiform Cortex, anterior division (left)**	β = 0.044, p = 0.56, pFDR = 0.89	B = -0.044, p = 0.44, pFDR = 0.97
**Volume of grey matter in Temporal Fusiform Cortex, anterior division (right)**	β = 0.058, p = 0.46, pFDR = 0.83	B = -0.065, p = 0.26, pFDR = 0.97
**Volume of grey matter in Temporal Fusiform Cortex, posterior division (left)**	β = 0.033, p = 0.66, pFDR = 0.89	B = -0.063, p = 0.25, pFDR = 0.97
**Volume of grey matter in Temporal Fusiform Cortex, posterior division (right)**	β = 5.47e-03, p = 0.94, pFDR = 0.97	B = -0.019, p = 0.73, pFDR = 0.97
**Volume of grey matter in Temporal Occipital Fusiform Cortex (left)**	β = -0.068, p = 0.39, pFDR = 0.8	B = 0.033, p = 0.57, pFDR = 0.97
**Volume of grey matter in Temporal Occipital Fusiform Cortex (right)**	β = -0.14, p = 0.058, pFDR = 0.34	B = 0.057, p = 0.3, pFDR = 0.97
**Volume of grey matter in Occipital Fusiform Gyrus (left)**	β = 0.042, p = 0.59, pFDR = 0.89	B = -0.06, p = 0.31, pFDR = 0.97
**Volume of grey matter in Occipital Fusiform Gyrus (right)**	β = -0.014, p = 0.86, pFDR = 0.94	B = -0.071, p = 0.24, pFDR = 0.97
**Volume of grey matter in Frontal Operculum Cortex (left)**	β = -0.037, p = 0.62, pFDR = 0.89	B = 0.083, p = 0.13, pFDR = 0.97
**Volume of grey matter in Frontal Operculum Cortex (right)**	β = -0.13, p = 0.098, pFDR = 0.47	B = 0.13, p = 0.025, pFDR = 0.96
**Volume of grey matter in Central Opercular Cortex (left)**	β = -9.29e-03, p = 0.9, pFDR = 0.95	B = 0.041, p = 0.44, pFDR = 0.97
**Volume of grey matter in Central Opercular Cortex (right)**	β = -0.018, p = 0.8, pFDR = 0.92	B = 0.01, p = 0.85, pFDR = 0.97
**Volume of grey matter in Parietal Operculum Cortex (left)**	β = -0.026, p = 0.74, pFDR = 0.89	B = 0.096, p = 0.095, pFDR = 0.97
**Volume of grey matter in Parietal Operculum Cortex (right)**	β = -0.1, p = 0.19, pFDR = 0.66	B = 0.11, p = 0.058, pFDR = 0.97
**Volume of grey matter in Planum Polare (left)**	β = 0.065, p = 0.39, pFDR = 0.8	B = -0.031, p = 0.58, pFDR = 0.97
**Volume of grey matter in Planum Polare (right)**	β = 0.033, p = 0.65, pFDR = 0.89	B = -0.024, p = 0.64, pFDR = 0.97
**Volume of grey matter in Heschl's Gyrus (includes H1 and H2) (left)**	β = 0.083, p = 0.26, pFDR = 0.71	B = -0.014, p = 0.79, pFDR = 0.97
**Volume of grey matter in Heschl's Gyrus (includes H1 and H2) (right)**	β = 0.015, p = 0.85, pFDR = 0.94	B = -9.74e-03, p = 0.86, pFDR = 0.97
**Volume of grey matter in Planum Temporale (left)**	β = -0.027, p = 0.72, pFDR = 0.89	B = 0.057, p = 0.29, pFDR = 0.97
**Volume of grey matter in Planum Temporale (right)**	β = -0.054, p = 0.46, pFDR = 0.83	B = 0.041, p = 0.45, pFDR = 0.97
**Volume of grey matter in Supracalcarine Cortex (left)**	β = -0.041, p = 0.58, pFDR = 0.89	B = 0.062, p = 0.25, pFDR = 0.97
**Volume of grey matter in Supracalcarine Cortex (right)**	β = 0.031, p = 0.68, pFDR = 0.89	B = 0.039, p = 0.49, pFDR = 0.97
**Volume of grey matter in Occipital Pole (left)**	β = -0.091, p = 0.24, pFDR = 0.7	B = -0.012, p = 0.84, pFDR = 0.97
**Volume of grey matter in Occipital Pole (right)**	β = -0.13, p = 0.063, pFDR = 0.34	B = 0.068, p = 0.21, pFDR = 0.97
**Volume of grey matter in Thalamus (left)**	β = 0.062, p = 0.42, pFDR = 0.82	B = 0.038, p = 0.5, pFDR = 0.97
**Volume of grey matter in Thalamus (right)**	β = 0.083, p = 0.28, pFDR = 0.71	B = -0.021, p = 0.71, pFDR = 0.97
**Volume of grey matter in Caudate (left)**	β = 0.038, p = 0.61, pFDR = 0.89	B = 0.033, p = 0.56, pFDR = 0.97
**Volume of grey matter in Caudate (right)**	β = 0.011, p = 0.88, pFDR = 0.94	B = 0.051, p = 0.35, pFDR = 0.97
**Volume of grey matter in Putamen (left)**	β = -0.028, p = 0.72, pFDR = 0.89	B = -0.041, p = 0.47, pFDR = 0.97
**Volume of grey matter in Putamen (right)**	β = -0.081, p = 0.29, pFDR = 0.71	B = 0.036, p = 0.53, pFDR = 0.97
**Volume of grey matter in Pallidum (left)**	β = 0.065, p = 0.4, pFDR = 0.8	B = 0.06, p = 0.3, pFDR = 0.97
**Volume of grey matter in Pallidum (right)**	β = 0.12, p = 0.12, pFDR = 0.53	B = 0.044, p = 0.45, pFDR = 0.97
**Volume of grey matter in Hippocampus (left)**	β = -0.2, p = 3.70e-03, pFDR = 0.094	B = -0.029, p = 0.56, pFDR = 0.97
**Volume of grey matter in Hippocampus (right)**	β = -0.21, p = 3.79e-03, pFDR = 0.094	B = 4.95e-03, p = 0.93, pFDR = 0.97
**Volume of grey matter in Amygdala (left)**	β = -0.16, p = 0.025, pFDR = 0.19	B = 0.024, p = 0.65, pFDR = 0.97
**Volume of grey matter in Amygdala (right)**	β = -0.17, p = 0.015, pFDR = 0.14	B = 0.037, p = 0.46, pFDR = 0.97
**Volume of grey matter in Ventral Striatum (left)**	β = 2.79e-03, p = 0.97, pFDR = 0.98	B = -6.46e-03, p = 0.9, pFDR = 0.97
**Volume of grey matter in Ventral Striatum (right)**	β = -0.012, p = 0.87, pFDR = 0.94	B = 0.044, p = 0.4, pFDR = 0.97

Table S4

cognition	term	effect	description
MMSE	beta1_0	0.213 [0.0157, 0.61]*	Mean FA in posterior corona radiata on FA skeleton (left)
MMSE	c_prime_0	-0.32 [-0.518, -0.127]*	Mean FA in posterior corona radiata on FA skeleton (left)
MMSE	total_0	-0.287 [-0.498, -0.1]*	Mean FA in posterior corona radiata on FA skeleton (left)
MMSE	beta1_1	0.179 [0.0191, 0.388]*	Mean FA in posterior corona radiata on FA skeleton (left)
MMSE	c_prime_1	-0.52 [-0.7, -0.245]*	Mean FA in posterior corona radiata on FA skeleton (left)
MMSE	total_1	-0.533 [-0.705, -0.268]*	Mean FA in posterior corona radiata on FA skeleton (left)
MMSE	beta1_2	0.206 [0.0214, 0.399]*	Mean FA in posterior corona radiata on FA skeleton (left)
MMSE	beta2_2	-0.197 [-0.441, -0.0271]*	Mean FA in posterior corona radiata on FA skeleton (left)
MMSE	total_2	-0.262 [-0.469, -0.0361]*	Mean FA in posterior corona radiata on FA skeleton (left)
MMSE	diff_beta2_2_0	-0.272 [-0.586, -0.0803]*	Mean FA in posterior corona radiata on FA skeleton (left)
MoCA	beta1_0	0.213 [0.0157, 0.61]*	Mean FA in posterior corona radiata on FA skeleton (left)
MoCA	beta3_0	0.126 [0.0171, 0.25]*	Mean FA in posterior corona radiata on FA skeleton (left)
MoCA	c_prime_0	-0.241 [-0.386, -0.0812]*	Mean FA in posterior corona radiata on FA skeleton (left)
MoCA	total_0	-0.22 [-0.363, -0.0657]*	Mean FA in posterior corona radiata on FA skeleton (left)
MoCA	beta1_1	0.179 [0.0191, 0.388]*	Mean FA in posterior corona radiata on FA skeleton (left)
MoCA	c_prime_1	-0.464 [-0.614, -0.213]*	Mean FA in posterior corona radiata on FA skeleton (left)
MoCA	total_1	-0.472 [-0.612, -0.227]*	Mean FA in posterior corona radiata on FA skeleton (left)
MoCA	beta1_2	0.206 [0.0214, 0.399]*	Mean FA in posterior corona radiata on FA skeleton (left)
MoCA	beta2_2	-0.197 [-0.441, -0.0271]*	Mean FA in posterior corona radiata on FA skeleton (left)
MoCA	c_prime_2	-0.331 [-0.479, -0.13]*	Mean FA in posterior corona radiata on FA skeleton (left)
MoCA	total_2	-0.361 [-0.526, -0.184]*	Mean FA in posterior corona radiata on FA skeleton (left)
MoCA	diff_beta2_2_0	-0.272 [-0.586, -0.0803]*	Mean FA in posterior corona radiata on FA skeleton (left)
MoCA	diff_ind1_2_0	-7.304e-03 [-0.0349, -3.797e-04]*	Mean FA in posterior corona radiata on FA skeleton (left)
EM	beta1_0	0.213 [0.0157, 0.61]*	Mean FA in posterior corona radiata on FA skeleton (left)
EM	c_prime_0	-0.241 [-0.36, -0.143]*	Mean FA in posterior corona radiata on FA skeleton (left)
EM	total_0	-0.25 [-0.352, -0.146]*	Mean FA in posterior corona radiata on FA skeleton (left)
EM	beta1_1	0.179 [0.0191, 0.388]*	Mean FA in posterior corona radiata on FA skeleton (left)
EM	c_prime_1	-0.183 [-0.326, -0.0334]*	Mean FA in posterior corona radiata on FA skeleton (left)
EM	total_1	-0.204 [-0.332, -0.0676]*	Mean FA in posterior corona radiata on FA skeleton (left)
EM	beta1_2	0.206 [0.0214, 0.399]*	Mean FA in posterior corona radiata on FA skeleton (left)
EM	beta2_2	-0.197 [-0.441, -0.0271]*	Mean FA in posterior corona radiata on FA skeleton (left)
EM	diff_beta2_2_0	-0.272 [-0.586, -0.0803]*	Mean FA in posterior corona radiata on FA skeleton (left)
ISP	beta1_0	0.213 [0.0157, 0.61]*	Mean FA in posterior corona radiata on FA skeleton (left)
ISP	c_prime_0	-0.185 [-0.318, -0.0108]*	Mean FA in posterior corona radiata on FA skeleton (left)
ISP	total_0	-0.169 [-0.307, -7.943e-03]*	Mean FA in posterior corona radiata on FA skeleton (left)
ISP	beta1_1	0.179 [0.0191, 0.388]*	Mean FA in posterior corona radiata on FA skeleton (left)
ISP	c_prime_1	-0.411 [-0.524, -0.259]*	Mean FA in posterior corona radiata on FA skeleton (left)
ISP	total_1	-0.399 [-0.514, -0.25]*	Mean FA in posterior corona radiata on FA skeleton (left)
ISP	beta1_2	0.206 [0.0214, 0.399]*	Mean FA in posterior corona radiata on FA skeleton (left)
ISP	beta2_2	-0.197 [-0.441, -0.0271]*	Mean FA in posterior corona radiata on FA skeleton (left)
ISP	c_prime_2	-0.285 [-0.418, -0.119]*	Mean FA in posterior corona radiata on FA skeleton (left)
ISP	total_2	-0.267 [-0.389, -0.118]*	Mean FA in posterior corona radiata on FA skeleton (left)
ISP	diff_beta2_2_0	-0.272 [-0.586, -0.0803]*	Mean FA in posterior corona radiata on FA skeleton (left)
ISP	diff_c_prime_1_0	-0.226 [-0.431, -0.0257]*	Mean FA in posterior corona radiata on FA skeleton (left)
ISP	diff_total_1_0	-0.23 [-0.426, -0.0261]*	Mean FA in posterior corona radiata on FA skeleton (left)
LF	beta1_0	0.213 [0.0157, 0.61]*	Mean FA in posterior corona radiata on FA skeleton (left)
LF	beta3_0	0.115 [0.0117, 0.227]*	Mean FA in posterior corona radiata on FA skeleton (left)
LF	c_prime_0	-0.238 [-0.376, -0.0849]*	Mean FA in posterior corona radiata on FA skeleton (left)
LF	total_0	-0.233 [-0.359, -0.0909]*	Mean FA in posterior corona radiata on FA skeleton (left)
LF	beta1_1	0.179 [0.0191, 0.388]*	Mean FA in posterior corona radiata on FA skeleton (left)
LF	c_prime_1	-0.242 [-0.376, -0.105]*	Mean FA in posterior corona radiata on FA skeleton (left)
LF	total_1	-0.23 [-0.369, -0.1]*	Mean FA in posterior corona radiata on FA skeleton (left)
LF	beta1_2	0.206 [0.0214, 0.399]*	Mean FA in posterior corona radiata on FA skeleton (left)
LF	beta2_2	-0.197 [-0.441, -0.0271]*	Mean FA in posterior corona radiata on FA skeleton (left)
LF	c_prime_2	-0.306 [-0.451, -0.127]*	Mean FA in posterior corona radiata on FA skeleton (left)
LF	total_2	-0.294 [-0.436, -0.133]*	Mean FA in posterior corona radiata on FA skeleton (left)
LF	diff_beta2_2_0	-0.272 [-0.586, -0.0803]*	Mean FA in posterior corona radiata on FA skeleton (left)
EF	beta1_0	0.213 [0.0157, 0.61]*	Mean FA in posterior corona radiata on FA skeleton (left)
EF	beta3_0	0.161 [0.0342, 0.418]*	Mean FA in posterior corona radiata on FA skeleton (left)
EF	beta1_1	0.179 [0.0191, 0.388]*	Mean FA in posterior corona radiata on FA skeleton (left)
EF	c_prime_1	-0.354 [-0.486, -0.196]*	Mean FA in posterior corona radiata on FA skeleton (left)
EF	total_1	-0.36 [-0.494, -0.207]*	Mean FA in posterior corona radiata on FA skeleton (left)
EF	beta1_2	0.206 [0.0214, 0.399]*	Mean FA in posterior corona radiata on FA skeleton (left)
EF	beta2_2	-0.197 [-0.441, -0.0271]*	Mean FA in posterior corona radiata on FA skeleton (left)
EF	c_prime_2	-0.241 [-0.391, -0.0141]*	Mean FA in posterior corona radiata on FA skeleton (left)
EF	total_2	-0.252 [-0.395, -0.0436]*	Mean FA in posterior corona radiata on FA skeleton (left)
EF	diff_beta2_2_0	-0.272 [-0.586, -0.0803]*	Mean FA in posterior corona radiata on FA skeleton (left)
EF	diff_ind1_2_0	-6.967e-03 [-0.032, -3.392e-05]*	Mean FA in posterior corona radiata on FA skeleton (left)
EF	diff_c_prime_1_0	-0.29 [-0.592, -0.0657]*	Mean FA in posterior corona radiata on FA skeleton (left)
EF	diff_total_1_0	-0.31 [-0.606, -0.0808]*	Mean FA in posterior corona radiata on FA skeleton (left)
VS	beta1_0	0.213 [0.0157, 0.61]*	Mean FA in posterior corona radiata on FA skeleton (left)
VS	beta3_0	0.107 [1.859e-03, 0.239]*	Mean FA in posterior corona radiata on FA skeleton (left)
VS	c_prime_0	-0.249 [-0.429, -0.0711]*	Mean FA in posterior corona radiata on FA skeleton (left)
VS	total_0	-0.223 [-0.396, -0.0501]*	Mean FA in posterior corona radiata on FA skeleton (left)
VS	beta1_1	0.179 [0.0191, 0.388]*	Mean FA in posterior corona radiata on FA skeleton (left)
VS	c_prime_1	-0.202 [-0.381, -0.0122]*	Mean FA in posterior corona radiata on FA skeleton (left)
VS	beta1_2	0.206 [0.0214, 0.399]*	Mean FA in posterior corona radiata on FA skeleton (left)
VS	beta2_2	-0.197 [-0.441, -0.0271]*	Mean FA in posterior corona radiata on FA skeleton (left)
VS	c_prime_2	-0.299 [-0.475, -0.0958]*	Mean FA in posterior corona radiata on FA skeleton (left)
VS	total_2	-0.292 [-0.475, -0.112]*	Mean FA in posterior corona radiata on FA skeleton (left)
VS	diff_beta2_2_0	-0.272 [-0.586, -0.0803]*	Mean FA in posterior corona radiata on FA skeleton (left)
MMSE	beta1_0	0.213 [0.0157, 0.61]*	Mean MD in retrolenticular part of internal capsule on FA skeleton (right)
MMSE	beta3_0	-0.258 [-0.493, -0.0673]*	Mean MD in retrolenticular part of internal capsule on FA skeleton (right)
MMSE	c_prime_0	-0.316 [-0.505, -0.138]*	Mean MD in retrolenticular part of internal capsule on FA skeleton (right)
MMSE	total_0	-0.287 [-0.498, -0.1]*	Mean MD in retrolenticular part of internal capsule on FA skeleton (right)
MMSE	beta1_1	0.179 [0.0191, 0.388]*	Mean MD in retrolenticular part of internal capsule on FA skeleton (right)
MMSE	beta4_1	0.208 [0.0472, 0.373]*	Mean MD in retrolenticular part of internal capsule on FA skeleton (right)
MMSE	c_prime_1	-0.505 [-0.685, -0.249]*	Mean MD in retrolenticular part of internal capsule on FA skeleton (right)
MMSE	total_1	-0.533 [-0.705, -0.268]*	Mean MD in retrolenticular part of internal capsule on FA skeleton (right)
MMSE	beta1_2	0.206 [0.0214, 0.399]*	Mean MD in retrolenticular part of internal capsule on FA skeleton (right)
MMSE	beta2_2	0.336 [0.181, 0.532]*	Mean MD in retrolenticular part of internal capsule on FA skeleton (right)
MMSE	beta4_2	0.196 [0.0109, 0.325]*	Mean MD in retrolenticular part of internal capsule on FA skeleton (right)
MMSE	total_2	-0.262 [-0.469, -0.0361]*	Mean MD in retrolenticular part of internal capsule on FA skeleton (right)
MMSE	diff_beta2_2_0	0.275 [0.0721, 0.649]*	Mean MD in retrolenticular part of internal capsule on FA skeleton (right)

MMSE	diff_beta2_2_1	0.27 [0.0101, 0.524]*	Mean MD in retrolenticular part of internal capsule on FA skeleton (right)
MoCA	beta1_0	0.213 [0.0157, 0.61]*	Mean MD in retrolenticular part of internal capsule on FA skeleton (right)
MoCA	beta3_0	-0.211 [-0.348, -0.0563]*	Mean MD in retrolenticular part of internal capsule on FA skeleton (right)
MoCA	c_prime_0	-0.236 [-0.376, -0.0753]*	Mean MD in retrolenticular part of internal capsule on FA skeleton (right)
MoCA	total_0	-0.22 [-0.363, -0.0657]*	Mean MD in retrolenticular part of internal capsule on FA skeleton (right)
MoCA	beta1_1	0.179 [0.0191, 0.388]*	Mean MD in retrolenticular part of internal capsule on FA skeleton (right)
MoCA	beta4_1	0.208 [0.0472, 0.373]*	Mean MD in retrolenticular part of internal capsule on FA skeleton (right)
MoCA	c_prime_1	-0.432 [-0.578, -0.194]*	Mean MD in retrolenticular part of internal capsule on FA skeleton (right)
MoCA	ind_1	-0.0399 [-0.11, -8.852e-04]*	Mean MD in retrolenticular part of internal capsule on FA skeleton (right)
MoCA	total_1	-0.472 [-0.612, -0.227]*	Mean MD in retrolenticular part of internal capsule on FA skeleton (right)
MoCA	beta1_2	0.206 [0.0214, 0.399]*	Mean MD in retrolenticular part of internal capsule on FA skeleton (right)
MoCA	beta2_2	0.336 [0.181, 0.532]*	Mean MD in retrolenticular part of internal capsule on FA skeleton (right)
MoCA	beta4_2	0.196 [0.0109, 0.325]*	Mean MD in retrolenticular part of internal capsule on FA skeleton (right)
MoCA	c_prime_2	-0.346 [-0.506, -0.146]*	Mean MD in retrolenticular part of internal capsule on FA skeleton (right)
MoCA	total_2	-0.361 [-0.526, -0.184]*	Mean MD in retrolenticular part of internal capsule on FA skeleton (right)
MoCA	diff_beta2_2_0	0.275 [0.0721, 0.649]*	Mean MD in retrolenticular part of internal capsule on FA skeleton (right)
MoCA	diff_beta2_2_1	0.27 [0.0101, 0.524]*	Mean MD in retrolenticular part of internal capsule on FA skeleton (right)
EM	beta1_0	0.213 [0.0157, 0.61]*	Mean MD in retrolenticular part of internal capsule on FA skeleton (right)
EM	c_prime_0	-0.238 [-0.367, -0.145]*	Mean MD in retrolenticular part of internal capsule on FA skeleton (right)
EM	total_0	-0.25 [-0.352, -0.146]*	Mean MD in retrolenticular part of internal capsule on FA skeleton (right)
EM	beta1_1	0.179 [0.0191, 0.388]*	Mean MD in retrolenticular part of internal capsule on FA skeleton (right)
EM	beta4_1	0.208 [0.0472, 0.373]*	Mean MD in retrolenticular part of internal capsule on FA skeleton (right)
EM	beta3_1	-0.151 [-0.289, -0.0102]*	Mean MD in retrolenticular part of internal capsule on FA skeleton (right)
EM	ind3_1	-0.0304 [-0.0824, -3.106e-03]*	Mean MD in retrolenticular part of internal capsule on FA skeleton (right)
EM	ind_1	-0.0499 [-0.135, -5.809e-03]*	Mean MD in retrolenticular part of internal capsule on FA skeleton (right)
EM	total_1	-0.204 [-0.332, -0.0676]*	Mean MD in retrolenticular part of internal capsule on FA skeleton (right)
EM	beta1_2	0.206 [0.0214, 0.399]*	Mean MD in retrolenticular part of internal capsule on FA skeleton (right)
EM	beta2_2	0.336 [0.181, 0.532]*	Mean MD in retrolenticular part of internal capsule on FA skeleton (right)
EM	beta4_2	0.196 [0.0109, 0.325]*	Mean MD in retrolenticular part of internal capsule on FA skeleton (right)
EM	diff_beta2_2_0	0.275 [0.0721, 0.649]*	Mean MD in retrolenticular part of internal capsule on FA skeleton (right)
EM	diff_beta2_2_1	0.27 [0.0101, 0.524]*	Mean MD in retrolenticular part of internal capsule on FA skeleton (right)
EM	diff_beta3_2_1	0.236 [7.189e-03, 0.466]*	Mean MD in retrolenticular part of internal capsule on FA skeleton (right)
ISP	beta1_0	0.213 [0.0157, 0.61]*	Mean MD in retrolenticular part of internal capsule on FA skeleton (right)
ISP	c_prime_0	-0.182 [-0.306, -6.469e-03]*	Mean MD in retrolenticular part of internal capsule on FA skeleton (right)
ISP	total_0	-0.169 [-0.307, -7.943e-03]*	Mean MD in retrolenticular part of internal capsule on FA skeleton (right)
ISP	beta1_1	0.179 [0.0191, 0.388]*	Mean MD in retrolenticular part of internal capsule on FA skeleton (right)
ISP	beta4_1	0.208 [0.0472, 0.373]*	Mean MD in retrolenticular part of internal capsule on FA skeleton (right)
ISP	c_prime_1	-0.4 [-0.519, -0.254]*	Mean MD in retrolenticular part of internal capsule on FA skeleton (right)
ISP	total_1	-0.399 [-0.514, -0.25]*	Mean MD in retrolenticular part of internal capsule on FA skeleton (right)
ISP	beta1_2	0.206 [0.0214, 0.399]*	Mean MD in retrolenticular part of internal capsule on FA skeleton (right)
ISP	beta2_2	0.336 [0.181, 0.532]*	Mean MD in retrolenticular part of internal capsule on FA skeleton (right)
ISP	beta4_2	0.196 [0.0109, 0.325]*	Mean MD in retrolenticular part of internal capsule on FA skeleton (right)
ISP	c_prime_2	-0.295 [-0.434, -0.12]*	Mean MD in retrolenticular part of internal capsule on FA skeleton (right)
ISP	total_2	-0.267 [-0.389, -0.118]*	Mean MD in retrolenticular part of internal capsule on FA skeleton (right)
ISP	diff_beta2_2_0	0.275 [0.0721, 0.649]*	Mean MD in retrolenticular part of internal capsule on FA skeleton (right)
ISP	diff_beta2_2_1	0.27 [0.0101, 0.524]*	Mean MD in retrolenticular part of internal capsule on FA skeleton (right)
ISP	diff_c_prime_1_0	-0.217 [-0.43, -0.0314]*	Mean MD in retrolenticular part of internal capsule on FA skeleton (right)
ISP	diff_total_1_0	-0.23 [-0.426, -0.0261]*	Mean MD in retrolenticular part of internal capsule on FA skeleton (right)
LF	beta1_0	0.213 [0.0157, 0.61]*	Mean MD in retrolenticular part of internal capsule on FA skeleton (right)
LF	c_prime_0	-0.236 [-0.369, -0.0802]*	Mean MD in retrolenticular part of internal capsule on FA skeleton (right)
LF	total_0	-0.233 [-0.359, -0.0909]*	Mean MD in retrolenticular part of internal capsule on FA skeleton (right)
LF	beta1_1	0.179 [0.0191, 0.388]*	Mean MD in retrolenticular part of internal capsule on FA skeleton (right)
LF	beta4_1	0.208 [0.0472, 0.373]*	Mean MD in retrolenticular part of internal capsule on FA skeleton (right)
LF	c_prime_1	-0.202 [-0.34, -0.0767]*	Mean MD in retrolenticular part of internal capsule on FA skeleton (right)
LF	ind3_1	-0.04 [-0.089, -5.488e-04]*	Mean MD in retrolenticular part of internal capsule on FA skeleton (right)
LF	total_1	-0.23 [-0.369, -0.1]*	Mean MD in retrolenticular part of internal capsule on FA skeleton (right)
LF	beta1_2	0.206 [0.0214, 0.399]*	Mean MD in retrolenticular part of internal capsule on FA skeleton (right)
LF	beta2_2	0.336 [0.181, 0.532]*	Mean MD in retrolenticular part of internal capsule on FA skeleton (right)
LF	beta4_2	0.196 [0.0109, 0.325]*	Mean MD in retrolenticular part of internal capsule on FA skeleton (right)
LF	c_prime_2	-0.295 [-0.441, -0.108]*	Mean MD in retrolenticular part of internal capsule on FA skeleton (right)
LF	total_2	-0.294 [-0.436, -0.133]*	Mean MD in retrolenticular part of internal capsule on FA skeleton (right)
LF	diff_beta2_2_0	0.275 [0.0721, 0.649]*	Mean MD in retrolenticular part of internal capsule on FA skeleton (right)
LF	diff_beta2_2_1	0.27 [0.0101, 0.524]*	Mean MD in retrolenticular part of internal capsule on FA skeleton (right)
EF	beta1_0	0.213 [0.0157, 0.61]*	Mean MD in retrolenticular part of internal capsule on FA skeleton (right)
EF	beta1_1	0.179 [0.0191, 0.388]*	Mean MD in retrolenticular part of internal capsule on FA skeleton (right)
EF	beta4_1	0.208 [0.0472, 0.373]*	Mean MD in retrolenticular part of internal capsule on FA skeleton (right)
EF	c_prime_1	-0.346 [-0.477, -0.194]*	Mean MD in retrolenticular part of internal capsule on FA skeleton (right)
EF	total_1	-0.36 [-0.494, -0.207]*	Mean MD in retrolenticular part of internal capsule on FA skeleton (right)
EF	beta1_2	0.206 [0.0214, 0.399]*	Mean MD in retrolenticular part of internal capsule on FA skeleton (right)
EF	beta2_2	0.336 [0.181, 0.532]*	Mean MD in retrolenticular part of internal capsule on FA skeleton (right)
EF	beta4_2	0.196 [0.0109, 0.325]*	Mean MD in retrolenticular part of internal capsule on FA skeleton (right)
EF	c_prime_2	-0.259 [-0.414, -0.0211]*	Mean MD in retrolenticular part of internal capsule on FA skeleton (right)
EF	total_2	-0.252 [-0.395, -0.0436]*	Mean MD in retrolenticular part of internal capsule on FA skeleton (right)
EF	diff_beta2_2_0	0.275 [0.0721, 0.649]*	Mean MD in retrolenticular part of internal capsule on FA skeleton (right)
EF	diff_beta2_2_1	0.27 [0.0101, 0.524]*	Mean MD in retrolenticular part of internal capsule on FA skeleton (right)
EF	diff_c_prime_1_0	-0.288 [-0.608, -0.0711]*	Mean MD in retrolenticular part of internal capsule on FA skeleton (right)
EF	diff_total_1_0	-0.31 [-0.606, -0.0808]*	Mean MD in retrolenticular part of internal capsule on FA skeleton (right)
VS	beta1_0	0.213 [0.0157, 0.61]*	Mean MD in retrolenticular part of internal capsule on FA skeleton (right)
VS	c_prime_0	-0.245 [-0.423, -0.063]*	Mean MD in retrolenticular part of internal capsule on FA skeleton (right)
VS	total_0	-0.223 [-0.396, -0.0501]*	Mean MD in retrolenticular part of internal capsule on FA skeleton (right)
VS	beta1_1	0.179 [0.0191, 0.388]*	Mean MD in retrolenticular part of internal capsule on FA skeleton (right)
VS	beta4_1	0.208 [0.0472, 0.373]*	Mean MD in retrolenticular part of internal capsule on FA skeleton (right)
VS	beta3_1	-0.181 [-0.346, -0.0126]*	Mean MD in retrolenticular part of internal capsule on FA skeleton (right)
VS	ind3_1	-0.0379 [-0.106, -3.567e-03]*	Mean MD in retrolenticular part of internal capsule on FA skeleton (right)
VS	beta1_2	0.206 [0.0214, 0.399]*	Mean MD in retrolenticular part of internal capsule on FA skeleton (right)
VS	beta2_2	0.336 [0.181, 0.532]*	Mean MD in retrolenticular part of internal capsule on FA skeleton (right)
VS	beta4_2	0.196 [0.0109, 0.325]*	Mean MD in retrolenticular part of internal capsule on FA skeleton (right)
VS	c_prime_2	-0.302 [-0.484, -0.106]*	Mean MD in retrolenticular part of internal capsule on FA skeleton (right)
VS	total_2	-0.292 [-0.475, -0.112]*	Mean MD in retrolenticular part of internal capsule on FA skeleton (right)
VS	diff_beta2_2_0	0.275 [0.0721, 0.649]*	Mean MD in retrolenticular part of internal capsule on FA skeleton (right)
VS	diff_beta2_2_1	0.27 [0.0101, 0.524]*	Mean MD in retrolenticular part of internal capsule on FA skeleton (right)
VS	diff_beta3_2_1	0.218 [3.366e-03, 0.454]*	Mean MD in retrolenticular part of internal capsule on FA skeleton (right)
MMSE	beta1_0	0.213 [0.0157, 0.61]*	Mean MD in retrolenticular part of internal capsule on FA skeleton (left)
MMSE	beta3_0	-0.172 [-0.369, -8.501e-03]*	Mean MD in retrolenticular part of internal capsule on FA skeleton (left)
MMSE	c_prime_0	-0.321 [-0.524, -0.139]*	Mean MD in retrolenticular part of internal capsule on FA skeleton (left)
MMSE	total_0	-0.287 [-0.498, -0.1]*	Mean MD in retrolenticular part of internal capsule on FA skeleton (left)

MMSE	beta1_1	0.179 [0.0191, 0.388]*	Mean MD in retrolenticular part of internal capsule on FA skeleton (left)
MMSE	c_prime_1	-0.502 [-0.679, -0.238]*	Mean MD in retrolenticular part of internal capsule on FA skeleton (left)
MMSE	ind_1	-0.0311 [-0.121, -4.083e-04]*	Mean MD in retrolenticular part of internal capsule on FA skeleton (left)
MMSE	total_1	-0.533 [-0.705, -0.268]*	Mean MD in retrolenticular part of internal capsule on FA skeleton (left)
MMSE	beta1_2	0.206 [0.0214, 0.399]*	Mean MD in retrolenticular part of internal capsule on FA skeleton (left)
MMSE	beta3_2	-0.191 [-0.434, -0.0337]*	Mean MD in retrolenticular part of internal capsule on FA skeleton (left)
MMSE	ind1_2	-8.389e-03 [-0.0486, -5.600e-04]*	Mean MD in retrolenticular part of internal capsule on FA skeleton (left)
MMSE	ind3_2	-0.0216 [-0.0741, -1.432e-03]*	Mean MD in retrolenticular part of internal capsule on FA skeleton (left)
MMSE	ind_2	-0.0387 [-0.138, -1.731e-03]*	Mean MD in retrolenticular part of internal capsule on FA skeleton (left)
MMSE	total_2	-0.262 [-0.469, -0.0361]*	Mean MD in retrolenticular part of internal capsule on FA skeleton (left)
MMSE	diff_ind_1_0	-0.0649 [-0.19, -1.219e-03]*	Mean MD in retrolenticular part of internal capsule on FA skeleton (left)
MMSE	diff_ind_2_0	-0.0725 [-0.227, -8.518e-03]*	Mean MD in retrolenticular part of internal capsule on FA skeleton (left)
MoCA	beta1_0	0.213 [0.0157, 0.61]*	Mean MD in retrolenticular part of internal capsule on FA skeleton (left)
MoCA	beta3_0	-0.223 [-0.351, -0.0926]*	Mean MD in retrolenticular part of internal capsule on FA skeleton (left)
MoCA	c_prime_0	-0.243 [-0.387, -0.0874]*	Mean MD in retrolenticular part of internal capsule on FA skeleton (left)
MoCA	total_0	-0.22 [-0.363, -0.0657]*	Mean MD in retrolenticular part of internal capsule on FA skeleton (left)
MoCA	beta1_1	0.179 [0.0191, 0.388]*	Mean MD in retrolenticular part of internal capsule on FA skeleton (left)
MoCA	c_prime_1	-0.449 [-0.596, -0.208]*	Mean MD in retrolenticular part of internal capsule on FA skeleton (left)
MoCA	total_1	-0.472 [-0.612, -0.227]*	Mean MD in retrolenticular part of internal capsule on FA skeleton (left)
MoCA	beta1_2	0.206 [0.0214, 0.399]*	Mean MD in retrolenticular part of internal capsule on FA skeleton (left)
MoCA	beta3_2	-0.156 [-0.334, -7.051e-03]*	Mean MD in retrolenticular part of internal capsule on FA skeleton (left)
MoCA	c_prime_2	-0.33 [-0.489, -0.137]*	Mean MD in retrolenticular part of internal capsule on FA skeleton (left)
MoCA	ind1_2	-6.949e-03 [-0.0345, -1.326e-04]*	Mean MD in retrolenticular part of internal capsule on FA skeleton (left)
MoCA	ind3_2	-0.0169 [-0.0525, -5.930e-04]*	Mean MD in retrolenticular part of internal capsule on FA skeleton (left)
MoCA	ind_2	-0.0304 [-0.112, -1.811e-04]*	Mean MD in retrolenticular part of internal capsule on FA skeleton (left)
MoCA	total_2	-0.361 [-0.526, -0.184]*	Mean MD in retrolenticular part of internal capsule on FA skeleton (left)
MoCA	diff_ind_2_0	-0.0535 [-0.158, -3.070e-03]*	Mean MD in retrolenticular part of internal capsule on FA skeleton (left)
EM	beta1_0	0.213 [0.0157, 0.61]*	Mean MD in retrolenticular part of internal capsule on FA skeleton (left)
EM	c_prime_0	-0.241 [-0.371, -0.152]*	Mean MD in retrolenticular part of internal capsule on FA skeleton (left)
EM	total_0	-0.25 [-0.352, -0.146]*	Mean MD in retrolenticular part of internal capsule on FA skeleton (left)
EM	beta1_1	0.179 [0.0191, 0.388]*	Mean MD in retrolenticular part of internal capsule on FA skeleton (left)
EM	beta3_1	-0.181 [-0.332, -0.0249]*	Mean MD in retrolenticular part of internal capsule on FA skeleton (left)
EM	ind_1	-0.0481 [-0.136, -4.012e-03]*	Mean MD in retrolenticular part of internal capsule on FA skeleton (left)
EM	total_1	-0.204 [-0.332, -0.0676]*	Mean MD in retrolenticular part of internal capsule on FA skeleton (left)
EM	beta1_2	0.206 [0.0214, 0.399]*	Mean MD in retrolenticular part of internal capsule on FA skeleton (left)
ISP	beta1_0	0.213 [0.0157, 0.61]*	Mean MD in retrolenticular part of internal capsule on FA skeleton (left)
ISP	c_prime_0	-0.185 [-0.312, -9.413e-03]*	Mean MD in retrolenticular part of internal capsule on FA skeleton (left)
ISP	total_0	-0.169 [-0.307, -7.943e-03]*	Mean MD in retrolenticular part of internal capsule on FA skeleton (left)
ISP	beta1_1	0.179 [0.0191, 0.388]*	Mean MD in retrolenticular part of internal capsule on FA skeleton (left)
ISP	c_prime_1	-0.406 [-0.519, -0.254]*	Mean MD in retrolenticular part of internal capsule on FA skeleton (left)
ISP	total_1	-0.399 [-0.514, -0.25]*	Mean MD in retrolenticular part of internal capsule on FA skeleton (left)
ISP	beta1_2	0.206 [0.0214, 0.399]*	Mean MD in retrolenticular part of internal capsule on FA skeleton (left)
ISP	c_prime_2	-0.273 [-0.4, -0.113]*	Mean MD in retrolenticular part of internal capsule on FA skeleton (left)
ISP	ind1_2	-4.980e-03 [-0.0393, -1.205e-03]*	Mean MD in retrolenticular part of internal capsule on FA skeleton (left)
ISP	total_2	-0.267 [-0.389, -0.118]*	Mean MD in retrolenticular part of internal capsule on FA skeleton (left)
ISP	diff_ind1_2_0	-4.674e-03 [-0.0356, -6.851e-05]*	Mean MD in retrolenticular part of internal capsule on FA skeleton (left)
ISP	diff_c_prime_1_0	-0.221 [-0.428, -0.0295]*	Mean MD in retrolenticular part of internal capsule on FA skeleton (left)
ISP	diff_total_1_0	-0.23 [-0.426, -0.0261]*	Mean MD in retrolenticular part of internal capsule on FA skeleton (left)
LF	beta1_0	0.213 [0.0157, 0.61]*	Mean MD in retrolenticular part of internal capsule on FA skeleton (left)
LF	c_prime_0	-0.238 [-0.373, -0.0836]*	Mean MD in retrolenticular part of internal capsule on FA skeleton (left)
LF	total_0	-0.233 [-0.359, -0.0909]*	Mean MD in retrolenticular part of internal capsule on FA skeleton (left)
LF	beta1_1	0.179 [0.0191, 0.388]*	Mean MD in retrolenticular part of internal capsule on FA skeleton (left)
LF	c_prime_1	-0.242 [-0.382, -0.109]*	Mean MD in retrolenticular part of internal capsule on FA skeleton (left)
LF	total_1	-0.23 [-0.369, -0.1]*	Mean MD in retrolenticular part of internal capsule on FA skeleton (left)
LF	beta1_2	0.206 [0.0214, 0.399]*	Mean MD in retrolenticular part of internal capsule on FA skeleton (left)
LF	c_prime_2	-0.301 [-0.448, -0.124]*	Mean MD in retrolenticular part of internal capsule on FA skeleton (left)
LF	total_2	-0.294 [-0.436, -0.133]*	Mean MD in retrolenticular part of internal capsule on FA skeleton (left)
EF	beta1_0	0.213 [0.0157, 0.61]*	Mean MD in retrolenticular part of internal capsule on FA skeleton (left)
EF	beta3_0	-0.139 [-0.324, -3.288e-03]*	Mean MD in retrolenticular part of internal capsule on FA skeleton (left)
EF	beta1_1	0.179 [0.0191, 0.388]*	Mean MD in retrolenticular part of internal capsule on FA skeleton (left)
EF	c_prime_1	-0.35 [-0.472, -0.196]*	Mean MD in retrolenticular part of internal capsule on FA skeleton (left)
EF	total_1	-0.36 [-0.494, -0.207]*	Mean MD in retrolenticular part of internal capsule on FA skeleton (left)
EF	beta1_2	0.206 [0.0214, 0.399]*	Mean MD in retrolenticular part of internal capsule on FA skeleton (left)
EF	beta3_2	-0.182 [-0.348, -0.0352]*	Mean MD in retrolenticular part of internal capsule on FA skeleton (left)
EF	c_prime_2	-0.231 [-0.382, -0.0113]*	Mean MD in retrolenticular part of internal capsule on FA skeleton (left)
EF	ind1_2	-6.768e-03 [-0.0411, -1.941e-03]*	Mean MD in retrolenticular part of internal capsule on FA skeleton (left)
EF	ind3_2	-0.0223 [-0.0722, -5.806e-04]*	Mean MD in retrolenticular part of internal capsule on FA skeleton (left)
EF	total_2	-0.252 [-0.395, -0.0436]*	Mean MD in retrolenticular part of internal capsule on FA skeleton (left)
EF	diff_ind1_2_0	-5.977e-03 [-0.0371, -2.230e-04]*	Mean MD in retrolenticular part of internal capsule on FA skeleton (left)
EF	diff_c_prime_1_0	-0.288 [-0.588, -0.0635]*	Mean MD in retrolenticular part of internal capsule on FA skeleton (left)
EF	diff_total_1_0	-0.31 [-0.606, -0.0808]*	Mean MD in retrolenticular part of internal capsule on FA skeleton (left)
VS	beta1_0	0.213 [0.0157, 0.61]*	Mean MD in retrolenticular part of internal capsule on FA skeleton (left)
VS	c_prime_0	-0.248 [-0.429, -0.0698]*	Mean MD in retrolenticular part of internal capsule on FA skeleton (left)
VS	total_0	-0.223 [-0.396, -0.0501]*	Mean MD in retrolenticular part of internal capsule on FA skeleton (left)
VS	beta1_1	0.179 [0.0191, 0.388]*	Mean MD in retrolenticular part of internal capsule on FA skeleton (left)
VS	c_prime_1	-0.188 [-0.371, -8.930e-03]*	Mean MD in retrolenticular part of internal capsule on FA skeleton (left)
VS	beta1_2	0.206 [0.0214, 0.399]*	Mean MD in retrolenticular part of internal capsule on FA skeleton (left)
VS	c_prime_2	-0.292 [-0.471, -0.0878]*	Mean MD in retrolenticular part of internal capsule on FA skeleton (left)
VS	total_2	-0.292 [-0.475, -0.112]*	Mean MD in retrolenticular part of internal capsule on FA skeleton (left)
MMSE	beta1_0	0.213 [0.0157, 0.61]*	Mean MD in superior corona radiata on FA skeleton (right)
MMSE	c_prime_0	-0.328 [-0.527, -0.133]*	Mean MD in superior corona radiata on FA skeleton (right)
MMSE	total_0	-0.287 [-0.498, -0.1]*	Mean MD in superior corona radiata on FA skeleton (right)
MMSE	beta1_1	0.179 [0.0191, 0.388]*	Mean MD in superior corona radiata on FA skeleton (right)
MMSE	c_prime_1	-0.505 [-0.687, -0.243]*	Mean MD in superior corona radiata on FA skeleton (right)
MMSE	total_1	-0.533 [-0.705, -0.268]*	Mean MD in superior corona radiata on FA skeleton (right)
MMSE	beta1_2	0.206 [0.0214, 0.399]*	Mean MD in superior corona radiata on FA skeleton (right)
MMSE	beta2_2	0.251 [0.0853, 0.412]*	Mean MD in superior corona radiata on FA skeleton (right)
MMSE	beta4_2	0.169 [0.0349, 0.291]*	Mean MD in superior corona radiata on FA skeleton (right)
MMSE	total_2	-0.262 [-0.469, -0.0361]*	Mean MD in superior corona radiata on FA skeleton (right)
MMSE	diff_beta2_2_0	0.21 [0.0481, 0.473]*	Mean MD in superior corona radiata on FA skeleton (right)
MMSE	diff_beta4_2_0	0.255 [0.0217, 0.466]*	Mean MD in superior corona radiata on FA skeleton (right)
MMSE	diff_ind_2_0	-0.0787 [-0.216, -1.456e-03]*	Mean MD in superior corona radiata on FA skeleton (right)
MoCA	beta1_0	0.213 [0.0157, 0.61]*	Mean MD in superior corona radiata on FA skeleton (right)
MoCA	c_prime_0	-0.246 [-0.39, -0.0782]*	Mean MD in superior corona radiata on FA skeleton (right)
MoCA	total_0	-0.22 [-0.363, -0.0657]*	Mean MD in superior corona radiata on FA skeleton (right)
MoCA	beta1_1	0.179 [0.0191, 0.388]*	Mean MD in superior corona radiata on FA skeleton (right)

MoCA	beta3_1	-0.16 [-0.268, -4.841e-03]*	Mean MD in superior corona radiata on FA skeleton (right)
MoCA	c_prime_1	-0.444 [-0.593, -0.206]*	Mean MD in superior corona radiata on FA skeleton (right)
MoCA	total_1	-0.472 [-0.612, -0.227]*	Mean MD in superior corona radiata on FA skeleton (right)
MoCA	beta1_2	0.206 [0.0214, 0.399]*	Mean MD in superior corona radiata on FA skeleton (right)
MoCA	beta2_2	0.251 [0.0853, 0.412]*	Mean MD in superior corona radiata on FA skeleton (right)
MoCA	beta4_2	0.169 [0.0349, 0.291]*	Mean MD in superior corona radiata on FA skeleton (right)
MoCA	c_prime_2	-0.335 [-0.493, -0.138]*	Mean MD in superior corona radiata on FA skeleton (right)
MoCA	total_2	-0.361 [-0.526, -0.184]*	Mean MD in superior corona radiata on FA skeleton (right)
MoCA	diff_beta2_2_0	0.21 [0.0481, 0.473]*	Mean MD in superior corona radiata on FA skeleton (right)
MoCA	diff_beta4_2_0	0.255 [0.0217, 0.466]*	Mean MD in superior corona radiata on FA skeleton (right)
MoCA	diff_ind_2_0	-0.0518 [-0.152, -1.518e-04]*	Mean MD in superior corona radiata on FA skeleton (right)
EM	beta1_0	0.213 [0.0157, 0.61]*	Mean MD in superior corona radiata on FA skeleton (right)
EM	c_prime_0	-0.25 [-0.372, -0.153]*	Mean MD in superior corona radiata on FA skeleton (right)
EM	total_0	-0.25 [-0.352, -0.146]*	Mean MD in superior corona radiata on FA skeleton (right)
EM	beta1_1	0.179 [0.0191, 0.388]*	Mean MD in superior corona radiata on FA skeleton (right)
EM	beta3_1	-0.207 [-0.335, -0.0712]*	Mean MD in superior corona radiata on FA skeleton (right)
EM	ind_1	-0.0427 [-0.15, -6.541e-04]*	Mean MD in superior corona radiata on FA skeleton (right)
EM	total_1	-0.204 [-0.332, -0.0676]*	Mean MD in superior corona radiata on FA skeleton (right)
EM	beta1_2	0.206 [0.0214, 0.399]*	Mean MD in superior corona radiata on FA skeleton (right)
EM	beta2_2	0.251 [0.0853, 0.412]*	Mean MD in superior corona radiata on FA skeleton (right)
EM	beta4_2	0.169 [0.0349, 0.291]*	Mean MD in superior corona radiata on FA skeleton (right)
EM	diff_beta2_2_0	0.21 [0.0481, 0.473]*	Mean MD in superior corona radiata on FA skeleton (right)
EM	diff_beta3_2_1	0.257 [0.0462, 0.453]*	Mean MD in superior corona radiata on FA skeleton (right)
EM	diff_beta4_2_0	0.255 [0.0217, 0.466]*	Mean MD in superior corona radiata on FA skeleton (right)
ISP	beta1_0	0.213 [0.0157, 0.61]*	Mean MD in superior corona radiata on FA skeleton (right)
ISP	c_prime_0	-0.187 [-0.317, -2.219e-03]*	Mean MD in superior corona radiata on FA skeleton (right)
ISP	total_0	-0.169 [-0.307, -7.943e-03]*	Mean MD in superior corona radiata on FA skeleton (right)
ISP	beta1_1	0.179 [0.0191, 0.388]*	Mean MD in superior corona radiata on FA skeleton (right)
ISP	c_prime_1	-0.404 [-0.524, -0.258]*	Mean MD in superior corona radiata on FA skeleton (right)
ISP	total_1	-0.399 [-0.514, -0.25]*	Mean MD in superior corona radiata on FA skeleton (right)
ISP	beta1_2	0.206 [0.0214, 0.399]*	Mean MD in superior corona radiata on FA skeleton (right)
ISP	beta2_2	0.251 [0.0853, 0.412]*	Mean MD in superior corona radiata on FA skeleton (right)
ISP	beta4_2	0.169 [0.0349, 0.291]*	Mean MD in superior corona radiata on FA skeleton (right)
ISP	c_prime_2	-0.295 [-0.433, -0.129]*	Mean MD in superior corona radiata on FA skeleton (right)
ISP	total_2	-0.267 [-0.389, -0.118]*	Mean MD in superior corona radiata on FA skeleton (right)
ISP	diff_beta2_2_0	0.21 [0.0481, 0.473]*	Mean MD in superior corona radiata on FA skeleton (right)
ISP	diff_beta4_2_0	0.255 [0.0217, 0.466]*	Mean MD in superior corona radiata on FA skeleton (right)
ISP	diff_c_prime_1_0	-0.217 [-0.432, -0.0258]*	Mean MD in superior corona radiata on FA skeleton (right)
ISP	diff_total_1_0	-0.23 [-0.426, -0.0261]*	Mean MD in superior corona radiata on FA skeleton (right)
LF	beta1_0	0.213 [0.0157, 0.61]*	Mean MD in superior corona radiata on FA skeleton (right)
LF	c_prime_0	-0.239 [-0.375, -0.0748]*	Mean MD in superior corona radiata on FA skeleton (right)
LF	total_0	-0.233 [-0.359, -0.0909]*	Mean MD in superior corona radiata on FA skeleton (right)
LF	beta1_1	0.179 [0.0191, 0.388]*	Mean MD in superior corona radiata on FA skeleton (right)
LF	c_prime_1	-0.237 [-0.374, -0.104]*	Mean MD in superior corona radiata on FA skeleton (right)
LF	total_1	-0.23 [-0.369, -0.1]*	Mean MD in superior corona radiata on FA skeleton (right)
LF	beta1_2	0.206 [0.0214, 0.399]*	Mean MD in superior corona radiata on FA skeleton (right)
LF	beta2_2	0.251 [0.0853, 0.412]*	Mean MD in superior corona radiata on FA skeleton (right)
LF	beta4_2	0.169 [0.0349, 0.291]*	Mean MD in superior corona radiata on FA skeleton (right)
LF	c_prime_2	-0.314 [-0.463, -0.137]*	Mean MD in superior corona radiata on FA skeleton (right)
LF	total_2	-0.294 [-0.436, -0.133]*	Mean MD in superior corona radiata on FA skeleton (right)
LF	diff_beta2_2_0	0.21 [0.0481, 0.473]*	Mean MD in superior corona radiata on FA skeleton (right)
LF	diff_beta4_2_0	0.255 [0.0217, 0.466]*	Mean MD in superior corona radiata on FA skeleton (right)
EF	beta1_0	0.213 [0.0157, 0.61]*	Mean MD in superior corona radiata on FA skeleton (right)
EF	beta1_1	0.179 [0.0191, 0.388]*	Mean MD in superior corona radiata on FA skeleton (right)
EF	c_prime_1	-0.347 [-0.479, -0.197]*	Mean MD in superior corona radiata on FA skeleton (right)
EF	total_1	-0.36 [-0.494, -0.207]*	Mean MD in superior corona radiata on FA skeleton (right)
EF	beta1_2	0.206 [0.0214, 0.399]*	Mean MD in superior corona radiata on FA skeleton (right)
EF	beta2_2	0.251 [0.0853, 0.412]*	Mean MD in superior corona radiata on FA skeleton (right)
EF	beta4_2	0.169 [0.0349, 0.291]*	Mean MD in superior corona radiata on FA skeleton (right)
EF	c_prime_2	-0.249 [-0.406, -0.0227]*	Mean MD in superior corona radiata on FA skeleton (right)
EF	total_2	-0.252 [-0.395, -0.0436]*	Mean MD in superior corona radiata on FA skeleton (right)
EF	diff_beta2_2_0	0.21 [0.0481, 0.473]*	Mean MD in superior corona radiata on FA skeleton (right)
EF	diff_beta4_2_0	0.255 [0.0217, 0.466]*	Mean MD in superior corona radiata on FA skeleton (right)
EF	diff_c_prime_1_0	-0.282 [-0.592, -0.0646]*	Mean MD in superior corona radiata on FA skeleton (right)
EF	diff_total_1_0	-0.31 [-0.606, -0.0808]*	Mean MD in superior corona radiata on FA skeleton (right)
VS	beta1_0	0.213 [0.0157, 0.61]*	Mean MD in superior corona radiata on FA skeleton (right)
VS	c_prime_0	-0.25 [-0.427, -0.0602]*	Mean MD in superior corona radiata on FA skeleton (right)
VS	total_0	-0.223 [-0.396, -0.0501]*	Mean MD in superior corona radiata on FA skeleton (right)
VS	beta1_1	0.179 [0.0191, 0.388]*	Mean MD in superior corona radiata on FA skeleton (right)
VS	c_prime_1	-0.194 [-0.379, -0.0118]*	Mean MD in superior corona radiata on FA skeleton (right)
VS	beta1_2	0.206 [0.0214, 0.399]*	Mean MD in superior corona radiata on FA skeleton (right)
VS	beta2_2	0.251 [0.0853, 0.412]*	Mean MD in superior corona radiata on FA skeleton (right)
VS	beta4_2	0.169 [0.0349, 0.291]*	Mean MD in superior corona radiata on FA skeleton (right)
VS	c_prime_2	-0.31 [-0.493, -0.11]*	Mean MD in superior corona radiata on FA skeleton (right)
VS	total_2	-0.292 [-0.475, -0.112]*	Mean MD in superior corona radiata on FA skeleton (right)
VS	diff_beta2_2_0	0.21 [0.0481, 0.473]*	Mean MD in superior corona radiata on FA skeleton (right)
VS	diff_beta4_2_0	0.255 [0.0217, 0.466]*	Mean MD in superior corona radiata on FA skeleton (right)
MMSE	beta1_0	0.213 [0.0157, 0.61]*	Mean MD in superior corona radiata on FA skeleton (left)
MMSE	c_prime_0	-0.327 [-0.528, -0.131]*	Mean MD in superior corona radiata on FA skeleton (left)
MMSE	total_0	-0.287 [-0.498, -0.1]*	Mean MD in superior corona radiata on FA skeleton (left)
MMSE	beta1_1	0.179 [0.0191, 0.388]*	Mean MD in superior corona radiata on FA skeleton (left)
MMSE	beta3_1	-0.166 [-0.318, -0.0588]*	Mean MD in superior corona radiata on FA skeleton (left)
MMSE	c_prime_1	-0.498 [-0.676, -0.238]*	Mean MD in superior corona radiata on FA skeleton (left)
MMSE	total_1	-0.533 [-0.705, -0.268]*	Mean MD in superior corona radiata on FA skeleton (left)
MMSE	beta1_2	0.206 [0.0214, 0.399]*	Mean MD in superior corona radiata on FA skeleton (left)
MMSE	beta2_2	0.283 [0.119, 0.469]*	Mean MD in superior corona radiata on FA skeleton (left)
MMSE	ind1_2	-8.322e-03 [-0.0467, -1.027e-03]*	Mean MD in superior corona radiata on FA skeleton (left)
MMSE	total_2	-0.262 [-0.469, -0.0361]*	Mean MD in superior corona radiata on FA skeleton (left)
MMSE	diff_beta2_2_0	0.182 [0.0118, 0.488]*	Mean MD in superior corona radiata on FA skeleton (left)
MoCA	beta1_0	0.213 [0.0157, 0.61]*	Mean MD in superior corona radiata on FA skeleton (left)
MoCA	c_prime_0	-0.244 [-0.387, -0.0742]*	Mean MD in superior corona radiata on FA skeleton (left)
MoCA	total_0	-0.22 [-0.363, -0.0657]*	Mean MD in superior corona radiata on FA skeleton (left)
MoCA	beta1_1	0.179 [0.0191, 0.388]*	Mean MD in superior corona radiata on FA skeleton (left)
MoCA	beta3_1	-0.187 [-0.304, -0.0772]*	Mean MD in superior corona radiata on FA skeleton (left)
MoCA	c_prime_1	-0.44 [-0.586, -0.203]*	Mean MD in superior corona radiata on FA skeleton (left)
MoCA	total_1	-0.472 [-0.612, -0.227]*	Mean MD in superior corona radiata on FA skeleton (left)

MoCA	beta1_2	0.206 [0.0214, 0.399]*	Mean MD in superior corona radiata on FA skeleton (left)
MoCA	beta2_2	0.283 [0.119, 0.469]*	Mean MD in superior corona radiata on FA skeleton (left)
MoCA	c_prime_2	-0.342 [-0.493, -0.147]*	Mean MD in superior corona radiata on FA skeleton (left)
MoCA	ind1_2	-5.597e-03 [-0.0326, -8.691e-04]*	Mean MD in superior corona radiata on FA skeleton (left)
MoCA	total_2	-0.361 [-0.526, -0.184]*	Mean MD in superior corona radiata on FA skeleton (left)
MoCA	diff_beta2_2_0	0.182 [0.0118, 0.488]*	Mean MD in superior corona radiata on FA skeleton (left)
EM	beta1_0	0.213 [0.0157, 0.61]*	Mean MD in superior corona radiata on FA skeleton (left)
EM	c_prime_0	-0.252 [-0.377, -0.154]*	Mean MD in superior corona radiata on FA skeleton (left)
EM	total_0	-0.25 [-0.352, -0.146]*	Mean MD in superior corona radiata on FA skeleton (left)
EM	beta1_1	0.179 [0.0191, 0.388]*	Mean MD in superior corona radiata on FA skeleton (left)
EM	beta3_1	-0.222 [-0.351, -0.0866]*	Mean MD in superior corona radiata on FA skeleton (left)
EM	c_prime_1	-0.159 [-0.295, -3.951e-04]*	Mean MD in superior corona radiata on FA skeleton (left)
EM	ind_1	-0.0449 [-0.154, -7.223e-04]*	Mean MD in superior corona radiata on FA skeleton (left)
EM	total_1	-0.204 [-0.332, -0.0676]*	Mean MD in superior corona radiata on FA skeleton (left)
EM	beta1_2	0.206 [0.0214, 0.399]*	Mean MD in superior corona radiata on FA skeleton (left)
EM	beta2_2	0.283 [0.119, 0.469]*	Mean MD in superior corona radiata on FA skeleton (left)
EM	diff_beta2_2_0	0.182 [0.0118, 0.488]*	Mean MD in superior corona radiata on FA skeleton (left)
ISP	beta1_0	0.213 [0.0157, 0.61]*	Mean MD in superior corona radiata on FA skeleton (left)
ISP	c_prime_0	-0.186 [-0.317, -1.961e-03]*	Mean MD in superior corona radiata on FA skeleton (left)
ISP	total_0	-0.169 [-0.307, -7.943e-03]*	Mean MD in superior corona radiata on FA skeleton (left)
ISP	beta1_1	0.179 [0.0191, 0.388]*	Mean MD in superior corona radiata on FA skeleton (left)
ISP	c_prime_1	-0.399 [-0.517, -0.251]*	Mean MD in superior corona radiata on FA skeleton (left)
ISP	total_1	-0.399 [-0.514, -0.25]*	Mean MD in superior corona radiata on FA skeleton (left)
ISP	beta1_2	0.206 [0.0214, 0.399]*	Mean MD in superior corona radiata on FA skeleton (left)
ISP	beta2_2	0.283 [0.119, 0.469]*	Mean MD in superior corona radiata on FA skeleton (left)
ISP	c_prime_2	-0.289 [-0.42, -0.129]*	Mean MD in superior corona radiata on FA skeleton (left)
ISP	total_2	-0.267 [-0.389, -0.118]*	Mean MD in superior corona radiata on FA skeleton (left)
ISP	diff_beta2_2_0	0.182 [0.0118, 0.488]*	Mean MD in superior corona radiata on FA skeleton (left)
ISP	diff_c_prime_1_0	-0.214 [-0.431, -0.0249]*	Mean MD in superior corona radiata on FA skeleton (left)
ISP	diff_total_1_0	-0.23 [-0.426, -0.0261]*	Mean MD in superior corona radiata on FA skeleton (left)
LF	beta1_0	0.213 [0.0157, 0.61]*	Mean MD in superior corona radiata on FA skeleton (left)
LF	c_prime_0	-0.237 [-0.376, -0.0768]*	Mean MD in superior corona radiata on FA skeleton (left)
LF	total_0	-0.233 [-0.359, -0.0909]*	Mean MD in superior corona radiata on FA skeleton (left)
LF	beta1_1	0.179 [0.0191, 0.388]*	Mean MD in superior corona radiata on FA skeleton (left)
LF	c_prime_1	-0.237 [-0.376, -0.102]*	Mean MD in superior corona radiata on FA skeleton (left)
LF	total_1	-0.23 [-0.369, -0.1]*	Mean MD in superior corona radiata on FA skeleton (left)
LF	beta1_2	0.206 [0.0214, 0.399]*	Mean MD in superior corona radiata on FA skeleton (left)
LF	beta2_2	0.283 [0.119, 0.469]*	Mean MD in superior corona radiata on FA skeleton (left)
LF	c_prime_2	-0.309 [-0.454, -0.132]*	Mean MD in superior corona radiata on FA skeleton (left)
LF	total_2	-0.294 [-0.436, -0.133]*	Mean MD in superior corona radiata on FA skeleton (left)
LF	diff_beta2_2_0	0.182 [0.0118, 0.488]*	Mean MD in superior corona radiata on FA skeleton (left)
EF	beta1_0	0.213 [0.0157, 0.61]*	Mean MD in superior corona radiata on FA skeleton (left)
EF	beta1_1	0.179 [0.0191, 0.388]*	Mean MD in superior corona radiata on FA skeleton (left)
EF	c_prime_1	-0.34 [-0.471, -0.189]*	Mean MD in superior corona radiata on FA skeleton (left)
EF	total_1	-0.36 [-0.494, -0.207]*	Mean MD in superior corona radiata on FA skeleton (left)
EF	beta1_2	0.206 [0.0214, 0.399]*	Mean MD in superior corona radiata on FA skeleton (left)
EF	beta2_2	0.283 [0.119, 0.469]*	Mean MD in superior corona radiata on FA skeleton (left)
EF	c_prime_2	-0.248 [-0.4, -0.0303]*	Mean MD in superior corona radiata on FA skeleton (left)
EF	ind1_2	-5.338e-03 [-0.0339, -5.307e-05]*	Mean MD in superior corona radiata on FA skeleton (left)
EF	total_2	-0.252 [-0.395, -0.0436]*	Mean MD in superior corona radiata on FA skeleton (left)
EF	diff_beta2_2_0	0.182 [0.0118, 0.488]*	Mean MD in superior corona radiata on FA skeleton (left)
EF	diff_c_prime_1_0	-0.277 [-0.59, -0.0596]*	Mean MD in superior corona radiata on FA skeleton (left)
EF	diff_total_1_0	-0.31 [-0.606, -0.0808]*	Mean MD in superior corona radiata on FA skeleton (left)
VS	beta1_0	0.213 [0.0157, 0.61]*	Mean MD in superior corona radiata on FA skeleton (left)
VS	c_prime_0	-0.25 [-0.428, -0.0596]*	Mean MD in superior corona radiata on FA skeleton (left)
VS	total_0	-0.223 [-0.396, -0.0501]*	Mean MD in superior corona radiata on FA skeleton (left)
VS	beta1_1	0.179 [0.0191, 0.388]*	Mean MD in superior corona radiata on FA skeleton (left)
VS	c_prime_1	-0.19 [-0.376, -0.0132]*	Mean MD in superior corona radiata on FA skeleton (left)
VS	beta1_2	0.206 [0.0214, 0.399]*	Mean MD in superior corona radiata on FA skeleton (left)
VS	beta2_2	0.283 [0.119, 0.469]*	Mean MD in superior corona radiata on FA skeleton (left)
VS	c_prime_2	-0.301 [-0.477, -0.0966]*	Mean MD in superior corona radiata on FA skeleton (left)
VS	total_2	-0.292 [-0.475, -0.112]*	Mean MD in superior corona radiata on FA skeleton (left)
VS	diff_beta2_2_0	0.182 [0.0118, 0.488]*	Mean MD in superior corona radiata on FA skeleton (left)
MMSE	beta1_0	0.213 [0.0157, 0.61]*	Mean MD in posterior corona radiata on FA skeleton (right)
MMSE	c_prime_0	-0.316 [-0.52, -0.136]*	Mean MD in posterior corona radiata on FA skeleton (right)
MMSE	total_0	-0.287 [-0.498, -0.1]*	Mean MD in posterior corona radiata on FA skeleton (right)
MMSE	beta1_1	0.179 [0.0191, 0.388]*	Mean MD in posterior corona radiata on FA skeleton (right)
MMSE	beta4_1	0.199 [0.0452, 0.398]*	Mean MD in posterior corona radiata on FA skeleton (right)
MMSE	c_prime_1	-0.495 [-0.677, -0.236]*	Mean MD in posterior corona radiata on FA skeleton (right)
MMSE	total_1	-0.533 [-0.705, -0.268]*	Mean MD in posterior corona radiata on FA skeleton (right)
MMSE	beta1_2	0.206 [0.0214, 0.399]*	Mean MD in posterior corona radiata on FA skeleton (right)
MMSE	beta4_2	0.191 [0.0103, 0.319]*	Mean MD in posterior corona radiata on FA skeleton (right)
MMSE	beta3_2	-0.218 [-0.426, -0.0605]*	Mean MD in posterior corona radiata on FA skeleton (right)
MMSE	ind1_2	-7.593e-03 [-0.0489, -6.137e-04]*	Mean MD in posterior corona radiata on FA skeleton (right)
MMSE	ind3_2	-0.0385 [-0.106, -8.799e-03]*	Mean MD in posterior corona radiata on FA skeleton (right)
MMSE	ind_2	-0.0557 [-0.154, -0.013]*	Mean MD in posterior corona radiata on FA skeleton (right)
MMSE	total_2	-0.262 [-0.469, -0.0361]*	Mean MD in posterior corona radiata on FA skeleton (right)
MMSE	diff_beta4_1_0	0.215 [0.0206, 0.491]*	Mean MD in posterior corona radiata on FA skeleton (right)
MMSE	diff_ind3_2_0	-0.0362 [-0.126, -5.344e-03]*	Mean MD in posterior corona radiata on FA skeleton (right)
MMSE	diff_ind_2_0	-0.0848 [-0.242, -0.0156]*	Mean MD in posterior corona radiata on FA skeleton (right)
MoCA	beta1_0	0.213 [0.0157, 0.61]*	Mean MD in posterior corona radiata on FA skeleton (right)
MoCA	beta3_0	-0.212 [-0.353, -0.0457]*	Mean MD in posterior corona radiata on FA skeleton (right)
MoCA	c_prime_0	-0.237 [-0.386, -0.0828]*	Mean MD in posterior corona radiata on FA skeleton (right)
MoCA	total_0	-0.22 [-0.363, -0.0657]*	Mean MD in posterior corona radiata on FA skeleton (right)
MoCA	beta1_1	0.179 [0.0191, 0.388]*	Mean MD in posterior corona radiata on FA skeleton (right)
MoCA	beta4_1	0.199 [0.0452, 0.398]*	Mean MD in posterior corona radiata on FA skeleton (right)
MoCA	c_prime_1	-0.429 [-0.579, -0.185]*	Mean MD in posterior corona radiata on FA skeleton (right)
MoCA	ind3_1	-0.0354 [-0.1, -2.183e-03]*	Mean MD in posterior corona radiata on FA skeleton (right)
MoCA	ind_1	-0.043 [-0.125, -2.504e-03]*	Mean MD in posterior corona radiata on FA skeleton (right)
MoCA	total_1	-0.472 [-0.612, -0.227]*	Mean MD in posterior corona radiata on FA skeleton (right)
MoCA	beta1_2	0.206 [0.0214, 0.399]*	Mean MD in posterior corona radiata on FA skeleton (right)
MoCA	beta4_2	0.191 [0.0103, 0.319]*	Mean MD in posterior corona radiata on FA skeleton (right)
MoCA	c_prime_2	-0.326 [-0.474, -0.113]*	Mean MD in posterior corona radiata on FA skeleton (right)
MoCA	ind1_2	-4.491e-03 [-0.0362, -8.968e-05]*	Mean MD in posterior corona radiata on FA skeleton (right)
MoCA	ind3_2	-0.021 [-0.0839, -7.882e-04]*	Mean MD in posterior corona radiata on FA skeleton (right)
MoCA	total_2	-0.361 [-0.526, -0.184]*	Mean MD in posterior corona radiata on FA skeleton (right)

MoCA	diff_beta4_1_0	0.215 [0.0206, 0.491]*	Mean MD in posterior corona radiata on FA skeleton (right)
EM	beta1_0	0.213 [0.0157, 0.61]*	Mean MD in posterior corona radiata on FA skeleton (right)
EM	c_prime_0	-0.236 [-0.377, -0.152]*	Mean MD in posterior corona radiata on FA skeleton (right)
EM	total_0	-0.25 [-0.352, -0.146]*	Mean MD in posterior corona radiata on FA skeleton (right)
EM	beta1_1	0.179 [0.0191, 0.388]*	Mean MD in posterior corona radiata on FA skeleton (right)
EM	beta4_1	0.199 [0.0452, 0.398]*	Mean MD in posterior corona radiata on FA skeleton (right)
EM	beta3_1	-0.173 [-0.313, -0.0246]*	Mean MD in posterior corona radiata on FA skeleton (right)
EM	ind3_1	-0.0332 [-0.0921, -4.567e-03]*	Mean MD in posterior corona radiata on FA skeleton (right)
EM	ind_1	-0.0527 [-0.15, -9.172e-03]*	Mean MD in posterior corona radiata on FA skeleton (right)
EM	total_1	-0.204 [-0.332, -0.0676]*	Mean MD in posterior corona radiata on FA skeleton (right)
EM	beta1_2	0.206 [0.0214, 0.399]*	Mean MD in posterior corona radiata on FA skeleton (right)
EM	beta4_2	0.191 [0.0103, 0.319]*	Mean MD in posterior corona radiata on FA skeleton (right)
EM	diff_beta4_1_0	0.215 [0.0206, 0.491]*	Mean MD in posterior corona radiata on FA skeleton (right)
ISP	beta1_0	0.213 [0.0157, 0.61]*	Mean MD in posterior corona radiata on FA skeleton (right)
ISP	c_prime_0	-0.183 [-0.311, -0.0118]*	Mean MD in posterior corona radiata on FA skeleton (right)
ISP	total_0	-0.169 [-0.307, -7.943e-03]*	Mean MD in posterior corona radiata on FA skeleton (right)
ISP	beta1_1	0.179 [0.0191, 0.388]*	Mean MD in posterior corona radiata on FA skeleton (right)
ISP	beta4_1	0.199 [0.0452, 0.398]*	Mean MD in posterior corona radiata on FA skeleton (right)
ISP	c_prime_1	-0.406 [-0.522, -0.256]*	Mean MD in posterior corona radiata on FA skeleton (right)
ISP	total_1	-0.399 [-0.514, -0.25]*	Mean MD in posterior corona radiata on FA skeleton (right)
ISP	beta1_2	0.206 [0.0214, 0.399]*	Mean MD in posterior corona radiata on FA skeleton (right)
ISP	beta4_2	0.191 [0.0103, 0.319]*	Mean MD in posterior corona radiata on FA skeleton (right)
ISP	c_prime_2	-0.282 [-0.409, -0.109]*	Mean MD in posterior corona radiata on FA skeleton (right)
ISP	total_2	-0.267 [-0.389, -0.118]*	Mean MD in posterior corona radiata on FA skeleton (right)
ISP	diff_beta4_1_0	0.215 [0.0206, 0.491]*	Mean MD in posterior corona radiata on FA skeleton (right)
ISP	diff_c_prime_1_0	-0.223 [-0.424, -0.03]*	Mean MD in posterior corona radiata on FA skeleton (right)
ISP	diff_total_1_0	-0.23 [-0.426, -0.0261]*	Mean MD in posterior corona radiata on FA skeleton (right)
LF	beta1_0	0.213 [0.0157, 0.61]*	Mean MD in posterior corona radiata on FA skeleton (right)
LF	c_prime_0	-0.236 [-0.371, -0.0851]*	Mean MD in posterior corona radiata on FA skeleton (right)
LF	total_0	-0.233 [-0.359, -0.0909]*	Mean MD in posterior corona radiata on FA skeleton (right)
LF	beta1_1	0.179 [0.0191, 0.388]*	Mean MD in posterior corona radiata on FA skeleton (right)
LF	beta4_1	0.199 [0.0452, 0.398]*	Mean MD in posterior corona radiata on FA skeleton (right)
LF	c_prime_1	-0.224 [-0.363, -0.0936]*	Mean MD in posterior corona radiata on FA skeleton (right)
LF	total_1	-0.23 [-0.369, -0.1]*	Mean MD in posterior corona radiata on FA skeleton (right)
LF	beta1_2	0.206 [0.0214, 0.399]*	Mean MD in posterior corona radiata on FA skeleton (right)
LF	beta4_2	0.191 [0.0103, 0.319]*	Mean MD in posterior corona radiata on FA skeleton (right)
LF	c_prime_2	-0.312 [-0.457, -0.127]*	Mean MD in posterior corona radiata on FA skeleton (right)
LF	total_2	-0.294 [-0.436, -0.133]*	Mean MD in posterior corona radiata on FA skeleton (right)
LF	diff_beta4_1_0	0.215 [0.0206, 0.491]*	Mean MD in posterior corona radiata on FA skeleton (right)
EF	beta1_0	0.213 [0.0157, 0.61]*	Mean MD in posterior corona radiata on FA skeleton (right)
EF	beta1_1	0.179 [0.0191, 0.388]*	Mean MD in posterior corona radiata on FA skeleton (right)
EF	beta4_1	0.199 [0.0452, 0.398]*	Mean MD in posterior corona radiata on FA skeleton (right)
EF	c_prime_1	-0.341 [-0.468, -0.187]*	Mean MD in posterior corona radiata on FA skeleton (right)
EF	total_1	-0.36 [-0.494, -0.207]*	Mean MD in posterior corona radiata on FA skeleton (right)
EF	beta1_2	0.206 [0.0214, 0.399]*	Mean MD in posterior corona radiata on FA skeleton (right)
EF	beta4_2	0.191 [0.0103, 0.319]*	Mean MD in posterior corona radiata on FA skeleton (right)
EF	c_prime_2	-0.24 [-0.389, -4.810e-03]*	Mean MD in posterior corona radiata on FA skeleton (right)
EF	total_2	-0.252 [-0.395, -0.0436]*	Mean MD in posterior corona radiata on FA skeleton (right)
EF	diff_beta4_1_0	0.215 [0.0206, 0.491]*	Mean MD in posterior corona radiata on FA skeleton (right)
EF	diff_c_prime_1_0	-0.283 [-0.595, -0.061]*	Mean MD in posterior corona radiata on FA skeleton (right)
EF	diff_total_1_0	-0.31 [-0.606, -0.0808]*	Mean MD in posterior corona radiata on FA skeleton (right)
VS	beta1_0	0.213 [0.0157, 0.61]*	Mean MD in posterior corona radiata on FA skeleton (right)
VS	c_prime_0	-0.245 [-0.429, -0.0667]*	Mean MD in posterior corona radiata on FA skeleton (right)
VS	total_0	-0.223 [-0.396, -0.0501]*	Mean MD in posterior corona radiata on FA skeleton (right)
VS	beta1_1	0.179 [0.0191, 0.388]*	Mean MD in posterior corona radiata on FA skeleton (right)
VS	beta4_1	0.199 [0.0452, 0.398]*	Mean MD in posterior corona radiata on FA skeleton (right)
VS	ind3_1	-0.0315 [-0.1, -8.149e-04]*	Mean MD in posterior corona radiata on FA skeleton (right)
VS	beta1_2	0.206 [0.0214, 0.399]*	Mean MD in posterior corona radiata on FA skeleton (right)
VS	beta4_2	0.191 [0.0103, 0.319]*	Mean MD in posterior corona radiata on FA skeleton (right)
VS	c_prime_2	-0.3 [-0.475, -0.0894]*	Mean MD in posterior corona radiata on FA skeleton (right)
VS	total_2	-0.292 [-0.475, -0.112]*	Mean MD in posterior corona radiata on FA skeleton (right)
VS	diff_beta4_1_0	0.215 [0.0206, 0.491]*	Mean MD in posterior corona radiata on FA skeleton (right)
MMSE	beta1_0	0.213 [0.0157, 0.61]*	Mean MD in posterior corona radiata on FA skeleton (left)
MMSE	beta3_0	-0.224 [-0.515, -6.815e-03]*	Mean MD in posterior corona radiata on FA skeleton (left)
MMSE	c_prime_0	-0.31 [-0.527, -0.139]*	Mean MD in posterior corona radiata on FA skeleton (left)
MMSE	total_0	-0.287 [-0.498, -0.1]*	Mean MD in posterior corona radiata on FA skeleton (left)
MMSE	beta1_1	0.179 [0.0191, 0.388]*	Mean MD in posterior corona radiata on FA skeleton (left)
MMSE	beta4_1	0.172 [0.0111, 0.388]*	Mean MD in posterior corona radiata on FA skeleton (left)
MMSE	c_prime_1	-0.489 [-0.665, -0.218]*	Mean MD in posterior corona radiata on FA skeleton (left)
MMSE	ind_1	-0.0443 [-0.146, -2.435e-03]*	Mean MD in posterior corona radiata on FA skeleton (left)
MMSE	total_1	-0.533 [-0.705, -0.268]*	Mean MD in posterior corona radiata on FA skeleton (left)
MMSE	beta1_2	0.206 [0.0214, 0.399]*	Mean MD in posterior corona radiata on FA skeleton (left)
MMSE	beta4_2	0.21 [0.052, 0.336]*	Mean MD in posterior corona radiata on FA skeleton (left)
MMSE	ind1_2	-5.276e-03 [-0.0383, -1.384e-04]*	Mean MD in posterior corona radiata on FA skeleton (left)
MMSE	ind3_2	-0.0289 [-0.0958, -3.138e-03]*	Mean MD in posterior corona radiata on FA skeleton (left)
MMSE	ind_2	-0.046 [-0.15, -1.621e-03]*	Mean MD in posterior corona radiata on FA skeleton (left)
MMSE	total_2	-0.262 [-0.469, -0.0361]*	Mean MD in posterior corona radiata on FA skeleton (left)
MMSE	diff_beta4_2_0	0.212 [0.0356, 0.455]*	Mean MD in posterior corona radiata on FA skeleton (left)
MMSE	diff_ind_2_0	-0.069 [-0.246, -4.690e-03]*	Mean MD in posterior corona radiata on FA skeleton (left)
MoCA	beta1_0	0.213 [0.0157, 0.61]*	Mean MD in posterior corona radiata on FA skeleton (left)
MoCA	beta3_0	-0.264 [-0.423, -0.0555]*	Mean MD in posterior corona radiata on FA skeleton (left)
MoCA	c_prime_0	-0.231 [-0.388, -0.0841]*	Mean MD in posterior corona radiata on FA skeleton (left)
MoCA	total_0	-0.22 [-0.363, -0.0657]*	Mean MD in posterior corona radiata on FA skeleton (left)
MoCA	beta1_1	0.179 [0.0191, 0.388]*	Mean MD in posterior corona radiata on FA skeleton (left)
MoCA	beta4_1	0.172 [0.0111, 0.388]*	Mean MD in posterior corona radiata on FA skeleton (left)
MoCA	beta3_1	-0.182 [-0.3, -0.0338]*	Mean MD in posterior corona radiata on FA skeleton (left)
MoCA	c_prime_1	-0.431 [-0.577, -0.178]*	Mean MD in posterior corona radiata on FA skeleton (left)
MoCA	ind3_1	-0.0336 [-0.099, -6.586e-04]*	Mean MD in posterior corona radiata on FA skeleton (left)
MoCA	total_1	-0.472 [-0.612, -0.227]*	Mean MD in posterior corona radiata on FA skeleton (left)
MoCA	beta1_2	0.206 [0.0214, 0.399]*	Mean MD in posterior corona radiata on FA skeleton (left)
MoCA	beta4_2	0.21 [0.052, 0.336]*	Mean MD in posterior corona radiata on FA skeleton (left)
MoCA	beta3_2	-0.144 [-0.326, -3.843e-03]*	Mean MD in posterior corona radiata on FA skeleton (left)
MoCA	c_prime_2	-0.32 [-0.47, -0.111]*	Mean MD in posterior corona radiata on FA skeleton (left)
MoCA	ind1_2	-5.057e-03 [-0.0348, -2.317e-04]*	Mean MD in posterior corona radiata on FA skeleton (left)
MoCA	ind3_2	-0.0276 [-0.0873, -5.294e-03]*	Mean MD in posterior corona radiata on FA skeleton (left)
MoCA	ind_2	-0.041 [-0.129, -4.711e-03]*	Mean MD in posterior corona radiata on FA skeleton (left)

MoCA	total_2	-0.361 [-0.526, -0.184]*	Mean MD in posterior corona radiata on FA skeleton (left)
MoCA	diff_beta4_2_0	0.212 [0.0356, 0.455]*	Mean MD in posterior corona radiata on FA skeleton (left)
MoCA	diff_ind_2_0	-0.0521 [-0.179, -3.498e-03]*	Mean MD in posterior corona radiata on FA skeleton (left)
EM	beta1_0	0.213 [0.0157, 0.61]*	Mean MD in posterior corona radiata on FA skeleton (left)
EM	c_prime_0	-0.231 [-0.378, -0.151]*	Mean MD in posterior corona radiata on FA skeleton (left)
EM	total_0	-0.25 [-0.352, -0.146]*	Mean MD in posterior corona radiata on FA skeleton (left)
EM	beta1_1	0.179 [0.0191, 0.388]*	Mean MD in posterior corona radiata on FA skeleton (left)
EM	beta4_1	0.172 [0.0111, 0.388]*	Mean MD in posterior corona radiata on FA skeleton (left)
EM	beta3_1	-0.249 [-0.377, -0.1]*	Mean MD in posterior corona radiata on FA skeleton (left)
EM	ind3_1	-0.0404 [-0.107, -5.828e-03]*	Mean MD in posterior corona radiata on FA skeleton (left)
EM	ind_1	-0.0599 [-0.165, -0.0122]*	Mean MD in posterior corona radiata on FA skeleton (left)
EM	total_1	-0.204 [-0.332, -0.0676]*	Mean MD in posterior corona radiata on FA skeleton (left)
EM	beta1_2	0.206 [0.0214, 0.399]*	Mean MD in posterior corona radiata on FA skeleton (left)
EM	beta4_2	0.21 [0.052, 0.336]*	Mean MD in posterior corona radiata on FA skeleton (left)
EM	diff_beta3_2_1	0.277 [0.0393, 0.46]*	Mean MD in posterior corona radiata on FA skeleton (left)
EM	diff_beta4_2_0	0.212 [0.0356, 0.455]*	Mean MD in posterior corona radiata on FA skeleton (left)
EM	diff_ind3_1_0	-0.0316 [-0.134, -1.738e-03]*	Mean MD in posterior corona radiata on FA skeleton (left)
EM	diff_ind_1_0	-0.0412 [-0.195, -6.274e-04]*	Mean MD in posterior corona radiata on FA skeleton (left)
ISP	beta1_0	0.213 [0.0157, 0.61]*	Mean MD in posterior corona radiata on FA skeleton (left)
ISP	beta3_0	-0.189 [-0.331, -1.062e-03]*	Mean MD in posterior corona radiata on FA skeleton (left)
ISP	c_prime_0	-0.177 [-0.313, -0.0158]*	Mean MD in posterior corona radiata on FA skeleton (left)
ISP	total_0	-0.169 [-0.307, -7.943e-03]*	Mean MD in posterior corona radiata on FA skeleton (left)
ISP	beta1_1	0.179 [0.0191, 0.388]*	Mean MD in posterior corona radiata on FA skeleton (left)
ISP	beta4_1	0.172 [0.0111, 0.388]*	Mean MD in posterior corona radiata on FA skeleton (left)
ISP	c_prime_1	-0.397 [-0.51, -0.247]*	Mean MD in posterior corona radiata on FA skeleton (left)
ISP	total_1	-0.399 [-0.514, -0.25]*	Mean MD in posterior corona radiata on FA skeleton (left)
ISP	beta1_2	0.206 [0.0214, 0.399]*	Mean MD in posterior corona radiata on FA skeleton (left)
ISP	beta4_2	0.21 [0.052, 0.336]*	Mean MD in posterior corona radiata on FA skeleton (left)
ISP	c_prime_2	-0.288 [-0.419, -0.114]*	Mean MD in posterior corona radiata on FA skeleton (left)
ISP	total_2	-0.267 [-0.389, -0.118]*	Mean MD in posterior corona radiata on FA skeleton (left)
ISP	diff_beta4_2_0	0.212 [0.0356, 0.455]*	Mean MD in posterior corona radiata on FA skeleton (left)
ISP	diff_c_prime_1_0	-0.22 [-0.414, -0.0209]*	Mean MD in posterior corona radiata on FA skeleton (left)
ISP	diff_total_1_0	-0.23 [-0.426, -0.0261]*	Mean MD in posterior corona radiata on FA skeleton (left)
LF	beta1_0	0.213 [0.0157, 0.61]*	Mean MD in posterior corona radiata on FA skeleton (left)
LF	c_prime_0	-0.231 [-0.375, -0.093]*	Mean MD in posterior corona radiata on FA skeleton (left)
LF	total_0	-0.233 [-0.359, -0.0909]*	Mean MD in posterior corona radiata on FA skeleton (left)
LF	beta1_1	0.179 [0.0191, 0.388]*	Mean MD in posterior corona radiata on FA skeleton (left)
LF	beta4_1	0.172 [0.0111, 0.388]*	Mean MD in posterior corona radiata on FA skeleton (left)
LF	c_prime_1	-0.232 [-0.376, -0.0963]*	Mean MD in posterior corona radiata on FA skeleton (left)
LF	total_1	-0.23 [-0.369, -0.1]*	Mean MD in posterior corona radiata on FA skeleton (left)
LF	beta1_2	0.206 [0.0214, 0.399]*	Mean MD in posterior corona radiata on FA skeleton (left)
LF	beta4_2	0.21 [0.052, 0.336]*	Mean MD in posterior corona radiata on FA skeleton (left)
LF	c_prime_2	-0.31 [-0.454, -0.121]*	Mean MD in posterior corona radiata on FA skeleton (left)
LF	total_2	-0.294 [-0.436, -0.133]*	Mean MD in posterior corona radiata on FA skeleton (left)
LF	diff_beta4_2_0	0.212 [0.0356, 0.455]*	Mean MD in posterior corona radiata on FA skeleton (left)
EF	beta1_0	0.213 [0.0157, 0.61]*	Mean MD in posterior corona radiata on FA skeleton (left)
EF	beta1_1	0.179 [0.0191, 0.388]*	Mean MD in posterior corona radiata on FA skeleton (left)
EF	beta4_1	0.172 [0.0111, 0.388]*	Mean MD in posterior corona radiata on FA skeleton (left)
EF	c_prime_1	-0.337 [-0.464, -0.183]*	Mean MD in posterior corona radiata on FA skeleton (left)
EF	total_1	-0.36 [-0.494, -0.207]*	Mean MD in posterior corona radiata on FA skeleton (left)
EF	beta1_2	0.206 [0.0214, 0.399]*	Mean MD in posterior corona radiata on FA skeleton (left)
EF	beta4_2	0.21 [0.052, 0.336]*	Mean MD in posterior corona radiata on FA skeleton (left)
EF	c_prime_2	-0.251 [-0.401, -0.014]*	Mean MD in posterior corona radiata on FA skeleton (left)
EF	total_2	-0.252 [-0.395, -0.0436]*	Mean MD in posterior corona radiata on FA skeleton (left)
EF	diff_beta4_2_0	0.212 [0.0356, 0.455]*	Mean MD in posterior corona radiata on FA skeleton (left)
EF	diff_c_prime_1_0	-0.282 [-0.586, -0.0551]*	Mean MD in posterior corona radiata on FA skeleton (left)
EF	diff_total_1_0	-0.31 [-0.606, -0.0808]*	Mean MD in posterior corona radiata on FA skeleton (left)
VS	beta1_0	0.213 [0.0157, 0.61]*	Mean MD in posterior corona radiata on FA skeleton (left)
VS	c_prime_0	-0.241 [-0.437, -0.0705]*	Mean MD in posterior corona radiata on FA skeleton (left)
VS	total_0	-0.223 [-0.396, -0.0501]*	Mean MD in posterior corona radiata on FA skeleton (left)
VS	beta1_1	0.179 [0.0191, 0.388]*	Mean MD in posterior corona radiata on FA skeleton (left)
VS	beta4_1	0.172 [0.0111, 0.388]*	Mean MD in posterior corona radiata on FA skeleton (left)
VS	beta1_2	0.206 [0.0214, 0.399]*	Mean MD in posterior corona radiata on FA skeleton (left)
VS	beta4_2	0.21 [0.052, 0.336]*	Mean MD in posterior corona radiata on FA skeleton (left)
VS	c_prime_2	-0.306 [-0.484, -0.0955]*	Mean MD in posterior corona radiata on FA skeleton (left)
VS	total_2	-0.292 [-0.475, -0.112]*	Mean MD in posterior corona radiata on FA skeleton (left)
VS	diff_beta4_2_0	0.212 [0.0356, 0.455]*	Mean MD in posterior corona radiata on FA skeleton (left)
MMSE	beta1_0	0.213 [0.0157, 0.61]*	Mean MD in posterior thalamic radiation on FA skeleton (right)
MMSE	beta3_0	-0.295 [-0.524, -0.106]*	Mean MD in posterior thalamic radiation on FA skeleton (right)
MMSE	c_prime_0	-0.314 [-0.52, -0.144]*	Mean MD in posterior thalamic radiation on FA skeleton (right)
MMSE	total_0	-0.287 [-0.498, -0.1]*	Mean MD in posterior thalamic radiation on FA skeleton (right)
MMSE	beta1_1	0.179 [0.0191, 0.388]*	Mean MD in posterior thalamic radiation on FA skeleton (right)
MMSE	beta4_1	0.198 [0.0315, 0.373]*	Mean MD in posterior thalamic radiation on FA skeleton (right)
MMSE	c_prime_1	-0.494 [-0.673, -0.235]*	Mean MD in posterior thalamic radiation on FA skeleton (right)
MMSE	ind_1	-0.0397 [-0.128, -6.378e-04]*	Mean MD in posterior thalamic radiation on FA skeleton (right)
MMSE	total_1	-0.533 [-0.705, -0.268]*	Mean MD in posterior thalamic radiation on FA skeleton (right)
MMSE	beta1_2	0.206 [0.0214, 0.399]*	Mean MD in posterior thalamic radiation on FA skeleton (right)
MMSE	beta4_2	0.222 [0.0788, 0.362]*	Mean MD in posterior thalamic radiation on FA skeleton (right)
MMSE	ind1_2	-4.317e-03 [-0.0295, -2.049e-04]*	Mean MD in posterior thalamic radiation on FA skeleton (right)
MMSE	ind3_2	-0.0302 [-0.0958, -9.022e-04]*	Mean MD in posterior thalamic radiation on FA skeleton (right)
MMSE	ind_2	-0.0473 [-0.159, -3.530e-03]*	Mean MD in posterior thalamic radiation on FA skeleton (right)
MMSE	total_2	-0.262 [-0.469, -0.0361]*	Mean MD in posterior thalamic radiation on FA skeleton (right)
MMSE	diff_beta4_2_0	0.22 [0.0157, 0.446]*	Mean MD in posterior thalamic radiation on FA skeleton (right)
MoCA	beta1_0	0.213 [0.0157, 0.61]*	Mean MD in posterior thalamic radiation on FA skeleton (right)
MoCA	beta3_0	-0.258 [-0.37, -0.132]*	Mean MD in posterior thalamic radiation on FA skeleton (right)
MoCA	c_prime_0	-0.235 [-0.382, -0.0852]*	Mean MD in posterior thalamic radiation on FA skeleton (right)
MoCA	total_0	-0.22 [-0.363, -0.0657]*	Mean MD in posterior thalamic radiation on FA skeleton (right)
MoCA	beta1_1	0.179 [0.0191, 0.388]*	Mean MD in posterior thalamic radiation on FA skeleton (right)
MoCA	beta4_1	0.198 [0.0315, 0.373]*	Mean MD in posterior thalamic radiation on FA skeleton (right)
MoCA	beta3_1	-0.172 [-0.31, -0.0111]*	Mean MD in posterior thalamic radiation on FA skeleton (right)
MoCA	c_prime_1	-0.431 [-0.576, -0.196]*	Mean MD in posterior thalamic radiation on FA skeleton (right)
MoCA	ind3_1	-0.0339 [-0.088, -2.522e-03]*	Mean MD in posterior thalamic radiation on FA skeleton (right)
MoCA	ind_1	-0.0414 [-0.112, -2.661e-03]*	Mean MD in posterior thalamic radiation on FA skeleton (right)
MoCA	total_1	-0.472 [-0.612, -0.227]*	Mean MD in posterior thalamic radiation on FA skeleton (right)
MoCA	beta1_2	0.206 [0.0214, 0.399]*	Mean MD in posterior thalamic radiation on FA skeleton (right)
MoCA	beta4_2	0.222 [0.0788, 0.362]*	Mean MD in posterior thalamic radiation on FA skeleton (right)

MoCA	c_prime_2	-0.331 [-0.482, -0.123]*	Mean MD in posterior thalamic radiation on FA skeleton (right)
MoCA	total_2	-0.361 [-0.526, -0.184]*	Mean MD in posterior thalamic radiation on FA skeleton (right)
MoCA	diff_beta4_2_0	0.22 [0.0157, 0.446]*	Mean MD in posterior thalamic radiation on FA skeleton (right)
EM	beta1_0	0.213 [0.0157, 0.61]*	Mean MD in posterior thalamic radiation on FA skeleton (right)
EM	beta3_0	-0.241 [-0.359, -0.0471]*	Mean MD in posterior thalamic radiation on FA skeleton (right)
EM	c_prime_0	-0.237 [-0.366, -0.147]*	Mean MD in posterior thalamic radiation on FA skeleton (right)
EM	total_0	-0.25 [-0.352, -0.146]*	Mean MD in posterior thalamic radiation on FA skeleton (right)
EM	beta1_1	0.179 [0.0191, 0.388]*	Mean MD in posterior thalamic radiation on FA skeleton (right)
EM	beta4_1	0.198 [0.0315, 0.373]*	Mean MD in posterior thalamic radiation on FA skeleton (right)
EM	beta3_1	-0.242 [-0.377, -0.0895]*	Mean MD in posterior thalamic radiation on FA skeleton (right)
EM	ind3_1	-0.0468 [-0.11, -8.800e-03]*	Mean MD in posterior thalamic radiation on FA skeleton (right)
EM	ind_1	-0.0663 [-0.17, -0.0153]*	Mean MD in posterior thalamic radiation on FA skeleton (right)
EM	total_1	-0.204 [-0.332, -0.0676]*	Mean MD in posterior thalamic radiation on FA skeleton (right)
EM	beta1_2	0.206 [0.0214, 0.399]*	Mean MD in posterior thalamic radiation on FA skeleton (right)
EM	beta4_2	0.222 [0.0788, 0.362]*	Mean MD in posterior thalamic radiation on FA skeleton (right)
EM	diff_beta3_2_0	0.275 [6.871e-03, 0.445]*	Mean MD in posterior thalamic radiation on FA skeleton (right)
EM	diff_beta3_2_1	0.276 [0.0479, 0.468]*	Mean MD in posterior thalamic radiation on FA skeleton (right)
EM	diff_beta4_2_0	0.22 [0.0157, 0.446]*	Mean MD in posterior thalamic radiation on FA skeleton (right)
ISP	beta1_0	0.213 [0.0157, 0.61]*	Mean MD in posterior thalamic radiation on FA skeleton (right)
ISP	beta3_0	-0.141 [-0.27, -0.0113]*	Mean MD in posterior thalamic radiation on FA skeleton (right)
ISP	c_prime_0	-0.181 [-0.31, -0.0105]*	Mean MD in posterior thalamic radiation on FA skeleton (right)
ISP	total_0	-0.169 [-0.307, -7.943e-03]*	Mean MD in posterior thalamic radiation on FA skeleton (right)
ISP	beta1_1	0.179 [0.0191, 0.388]*	Mean MD in posterior thalamic radiation on FA skeleton (right)
ISP	beta4_1	0.198 [0.0315, 0.373]*	Mean MD in posterior thalamic radiation on FA skeleton (right)
ISP	c_prime_1	-0.403 [-0.517, -0.253]*	Mean MD in posterior thalamic radiation on FA skeleton (right)
ISP	total_1	-0.399 [-0.514, -0.25]*	Mean MD in posterior thalamic radiation on FA skeleton (right)
ISP	beta1_2	0.206 [0.0214, 0.399]*	Mean MD in posterior thalamic radiation on FA skeleton (right)
ISP	beta4_2	0.222 [0.0788, 0.362]*	Mean MD in posterior thalamic radiation on FA skeleton (right)
ISP	c_prime_2	-0.287 [-0.419, -0.112]*	Mean MD in posterior thalamic radiation on FA skeleton (right)
ISP	total_2	-0.267 [-0.389, -0.118]*	Mean MD in posterior thalamic radiation on FA skeleton (right)
ISP	diff_beta4_2_0	0.22 [0.0157, 0.446]*	Mean MD in posterior thalamic radiation on FA skeleton (right)
ISP	diff_c_prime_1_0	-0.222 [-0.422, -0.0265]*	Mean MD in posterior thalamic radiation on FA skeleton (right)
ISP	diff_total_1_0	-0.23 [-0.426, -0.0261]*	Mean MD in posterior thalamic radiation on FA skeleton (right)
LF	beta1_0	0.213 [0.0157, 0.61]*	Mean MD in posterior thalamic radiation on FA skeleton (right)
LF	beta3_0	-0.17 [-0.303, -0.0203]*	Mean MD in posterior thalamic radiation on FA skeleton (right)
LF	c_prime_0	-0.233 [-0.371, -0.0907]*	Mean MD in posterior thalamic radiation on FA skeleton (right)
LF	total_0	-0.233 [-0.359, -0.0909]*	Mean MD in posterior thalamic radiation on FA skeleton (right)
LF	beta1_1	0.179 [0.0191, 0.388]*	Mean MD in posterior thalamic radiation on FA skeleton (right)
LF	beta4_1	0.198 [0.0315, 0.373]*	Mean MD in posterior thalamic radiation on FA skeleton (right)
LF	c_prime_1	-0.228 [-0.369, -0.0966]*	Mean MD in posterior thalamic radiation on FA skeleton (right)
LF	total_1	-0.23 [-0.369, -0.1]*	Mean MD in posterior thalamic radiation on FA skeleton (right)
LF	beta1_2	0.206 [0.0214, 0.399]*	Mean MD in posterior thalamic radiation on FA skeleton (right)
LF	beta4_2	0.222 [0.0788, 0.362]*	Mean MD in posterior thalamic radiation on FA skeleton (right)
LF	c_prime_2	-0.295 [-0.439, -0.111]*	Mean MD in posterior thalamic radiation on FA skeleton (right)
LF	total_2	-0.294 [-0.436, -0.133]*	Mean MD in posterior thalamic radiation on FA skeleton (right)
LF	diff_beta4_2_0	0.22 [0.0157, 0.446]*	Mean MD in posterior thalamic radiation on FA skeleton (right)
EF	beta1_0	0.213 [0.0157, 0.61]*	Mean MD in posterior thalamic radiation on FA skeleton (right)
EF	beta3_0	-0.191 [-0.331, -0.0702]*	Mean MD in posterior thalamic radiation on FA skeleton (right)
EF	beta1_1	0.179 [0.0191, 0.388]*	Mean MD in posterior thalamic radiation on FA skeleton (right)
EF	beta4_1	0.198 [0.0315, 0.373]*	Mean MD in posterior thalamic radiation on FA skeleton (right)
EF	c_prime_1	-0.342 [-0.468, -0.19]*	Mean MD in posterior thalamic radiation on FA skeleton (right)
EF	total_1	-0.36 [-0.494, -0.207]*	Mean MD in posterior thalamic radiation on FA skeleton (right)
EF	beta1_2	0.206 [0.0214, 0.399]*	Mean MD in posterior thalamic radiation on FA skeleton (right)
EF	beta4_2	0.222 [0.0788, 0.362]*	Mean MD in posterior thalamic radiation on FA skeleton (right)
EF	c_prime_2	-0.245 [-0.395, -0.0161]*	Mean MD in posterior thalamic radiation on FA skeleton (right)
EF	total_2	-0.252 [-0.395, -0.0436]*	Mean MD in posterior thalamic radiation on FA skeleton (right)
EF	diff_beta4_2_0	0.22 [0.0157, 0.446]*	Mean MD in posterior thalamic radiation on FA skeleton (right)
EF	diff_c_prime_1_0	-0.285 [-0.59, -0.0655]*	Mean MD in posterior thalamic radiation on FA skeleton (right)
EF	diff_total_1_0	-0.31 [-0.606, -0.0808]*	Mean MD in posterior thalamic radiation on FA skeleton (right)
VS	beta1_0	0.213 [0.0157, 0.61]*	Mean MD in posterior thalamic radiation on FA skeleton (right)
VS	beta3_0	-0.213 [-0.357, -0.0608]*	Mean MD in posterior thalamic radiation on FA skeleton (right)
VS	c_prime_0	-0.245 [-0.427, -0.0721]*	Mean MD in posterior thalamic radiation on FA skeleton (right)
VS	total_0	-0.223 [-0.396, -0.0501]*	Mean MD in posterior thalamic radiation on FA skeleton (right)
VS	beta1_1	0.179 [0.0191, 0.388]*	Mean MD in posterior thalamic radiation on FA skeleton (right)
VS	beta4_1	0.198 [0.0315, 0.373]*	Mean MD in posterior thalamic radiation on FA skeleton (right)
VS	beta3_1	-0.199 [-0.348, -0.0241]*	Mean MD in posterior thalamic radiation on FA skeleton (right)
VS	ind3_1	-0.0399 [-0.102, -3.078e-03]*	Mean MD in posterior thalamic radiation on FA skeleton (right)
VS	beta1_2	0.206 [0.0214, 0.399]*	Mean MD in posterior thalamic radiation on FA skeleton (right)
VS	beta4_2	0.222 [0.0788, 0.362]*	Mean MD in posterior thalamic radiation on FA skeleton (right)
VS	c_prime_2	-0.302 [-0.476, -0.1]*	Mean MD in posterior thalamic radiation on FA skeleton (right)
VS	total_2	-0.292 [-0.475, -0.112]*	Mean MD in posterior thalamic radiation on FA skeleton (right)
VS	diff_beta3_2_0	0.244 [0.0099, 0.427]*	Mean MD in posterior thalamic radiation on FA skeleton (right)
VS	diff_beta4_2_0	0.22 [0.0157, 0.446]*	Mean MD in posterior thalamic radiation on FA skeleton (right)
MMSE	beta1_0	0.213 [0.0157, 0.61]*	Mean MD in posterior thalamic radiation on FA skeleton (left)
MMSE	beta3_0	-0.199 [-0.442, -0.0129]*	Mean MD in posterior thalamic radiation on FA skeleton (left)
MMSE	c_prime_0	-0.317 [-0.523, -0.137]*	Mean MD in posterior thalamic radiation on FA skeleton (left)
MMSE	total_0	-0.287 [-0.498, -0.1]*	Mean MD in posterior thalamic radiation on FA skeleton (left)
MMSE	beta1_1	0.179 [0.0191, 0.388]*	Mean MD in posterior thalamic radiation on FA skeleton (left)
MMSE	c_prime_1	-0.505 [-0.69, -0.241]*	Mean MD in posterior thalamic radiation on FA skeleton (left)
MMSE	total_1	-0.533 [-0.705, -0.268]*	Mean MD in posterior thalamic radiation on FA skeleton (left)
MMSE	beta1_2	0.206 [0.0214, 0.399]*	Mean MD in posterior thalamic radiation on FA skeleton (left)
MMSE	beta4_2	0.201 [0.0878, 0.335]*	Mean MD in posterior thalamic radiation on FA skeleton (left)
MMSE	total_2	-0.262 [-0.469, -0.0361]*	Mean MD in posterior thalamic radiation on FA skeleton (left)
MMSE	diff_beta4_2_0	0.205 [0.0567, 0.436]*	Mean MD in posterior thalamic radiation on FA skeleton (left)
MoCA	beta1_0	0.213 [0.0157, 0.61]*	Mean MD in posterior thalamic radiation on FA skeleton (left)
MoCA	beta3_0	-0.243 [-0.39, -0.108]*	Mean MD in posterior thalamic radiation on FA skeleton (left)
MoCA	c_prime_0	-0.236 [-0.381, -0.0839]*	Mean MD in posterior thalamic radiation on FA skeleton (left)
MoCA	total_0	-0.22 [-0.363, -0.0657]*	Mean MD in posterior thalamic radiation on FA skeleton (left)
MoCA	beta1_1	0.179 [0.0191, 0.388]*	Mean MD in posterior thalamic radiation on FA skeleton (left)
MoCA	c_prime_1	-0.448 [-0.6, -0.206]*	Mean MD in posterior thalamic radiation on FA skeleton (left)
MoCA	total_1	-0.472 [-0.612, -0.227]*	Mean MD in posterior thalamic radiation on FA skeleton (left)
MoCA	beta1_2	0.206 [0.0214, 0.399]*	Mean MD in posterior thalamic radiation on FA skeleton (left)
MoCA	beta4_2	0.201 [0.0878, 0.335]*	Mean MD in posterior thalamic radiation on FA skeleton (left)
MoCA	c_prime_2	-0.328 [-0.492, -0.135]*	Mean MD in posterior thalamic radiation on FA skeleton (left)
MoCA	total_2	-0.361 [-0.526, -0.184]*	Mean MD in posterior thalamic radiation on FA skeleton (left)
MoCA	diff_beta4_2_0	0.205 [0.0567, 0.436]*	Mean MD in posterior thalamic radiation on FA skeleton (left)

EM	beta1_0	0.213 [0.0157, 0.61]*	Mean MD in posterior thalamic radiation on FA skeleton (left)
EM	c_prime_0	-0.236 [-0.37, -0.148]*	Mean MD in posterior thalamic radiation on FA skeleton (left)
EM	total_0	-0.25 [-0.352, -0.146]*	Mean MD in posterior thalamic radiation on FA skeleton (left)
EM	beta1_1	0.179 [0.0191, 0.388]*	Mean MD in posterior thalamic radiation on FA skeleton (left)
EM	beta3_1	-0.217 [-0.37, -0.0456]*	Mean MD in posterior thalamic radiation on FA skeleton (left)
EM	c_prime_1	-0.16 [-0.305, -5.381e-03]*	Mean MD in posterior thalamic radiation on FA skeleton (left)
EM	total_1	-0.204 [-0.332, -0.0676]*	Mean MD in posterior thalamic radiation on FA skeleton (left)
EM	beta1_2	0.206 [0.0214, 0.399]*	Mean MD in posterior thalamic radiation on FA skeleton (left)
EM	beta4_2	0.201 [0.0878, 0.335]*	Mean MD in posterior thalamic radiation on FA skeleton (left)
EM	diff_beta4_2_0	0.205 [0.0567, 0.436]*	Mean MD in posterior thalamic radiation on FA skeleton (left)
ISP	beta1_0	0.213 [0.0157, 0.61]*	Mean MD in posterior thalamic radiation on FA skeleton (left)
ISP	c_prime_0	-0.182 [-0.312, -0.0115]*	Mean MD in posterior thalamic radiation on FA skeleton (left)
ISP	total_0	-0.169 [-0.307, -7.943e-03]*	Mean MD in posterior thalamic radiation on FA skeleton (left)
ISP	beta1_1	0.179 [0.0191, 0.388]*	Mean MD in posterior thalamic radiation on FA skeleton (left)
ISP	c_prime_1	-0.4 [-0.515, -0.253]*	Mean MD in posterior thalamic radiation on FA skeleton (left)
ISP	total_1	-0.399 [-0.514, -0.25]*	Mean MD in posterior thalamic radiation on FA skeleton (left)
ISP	beta1_2	0.206 [0.0214, 0.399]*	Mean MD in posterior thalamic radiation on FA skeleton (left)
ISP	beta4_2	0.201 [0.0878, 0.335]*	Mean MD in posterior thalamic radiation on FA skeleton (left)
ISP	c_prime_2	-0.261 [-0.384, -0.0938]*	Mean MD in posterior thalamic radiation on FA skeleton (left)
ISP	ind3_2	-0.0288 [-0.094, -4.039e-03]*	Mean MD in posterior thalamic radiation on FA skeleton (left)
ISP	total_2	-0.267 [-0.389, -0.118]*	Mean MD in posterior thalamic radiation on FA skeleton (left)
ISP	diff_beta4_2_0	0.205 [0.0567, 0.436]*	Mean MD in posterior thalamic radiation on FA skeleton (left)
ISP	diff_c_prime_1_0	-0.219 [-0.421, -0.0254]*	Mean MD in posterior thalamic radiation on FA skeleton (left)
ISP	diff_total_1_0	-0.23 [-0.426, -0.0261]*	Mean MD in posterior thalamic radiation on FA skeleton (left)
LF	beta1_0	0.213 [0.0157, 0.61]*	Mean MD in posterior thalamic radiation on FA skeleton (left)
LF	beta3_0	-0.174 [-0.302, -1.452e-03]*	Mean MD in posterior thalamic radiation on FA skeleton (left)
LF	c_prime_0	-0.233 [-0.374, -0.0902]*	Mean MD in posterior thalamic radiation on FA skeleton (left)
LF	total_0	-0.233 [-0.359, -0.0909]*	Mean MD in posterior thalamic radiation on FA skeleton (left)
LF	beta1_1	0.179 [0.0191, 0.388]*	Mean MD in posterior thalamic radiation on FA skeleton (left)
LF	c_prime_1	-0.234 [-0.369, -0.0979]*	Mean MD in posterior thalamic radiation on FA skeleton (left)
LF	total_1	-0.23 [-0.369, -0.1]*	Mean MD in posterior thalamic radiation on FA skeleton (left)
LF	beta1_2	0.206 [0.0214, 0.399]*	Mean MD in posterior thalamic radiation on FA skeleton (left)
LF	beta4_2	0.201 [0.0878, 0.335]*	Mean MD in posterior thalamic radiation on FA skeleton (left)
LF	c_prime_2	-0.298 [-0.449, -0.12]*	Mean MD in posterior thalamic radiation on FA skeleton (left)
LF	total_2	-0.294 [-0.436, -0.133]*	Mean MD in posterior thalamic radiation on FA skeleton (left)
LF	diff_beta4_2_0	0.205 [0.0567, 0.436]*	Mean MD in posterior thalamic radiation on FA skeleton (left)
EF	beta1_0	0.213 [0.0157, 0.61]*	Mean MD in posterior thalamic radiation on FA skeleton (left)
EF	beta3_0	-0.134 [-0.243, -3.416e-03]*	Mean MD in posterior thalamic radiation on FA skeleton (left)
EF	beta1_1	0.179 [0.0191, 0.388]*	Mean MD in posterior thalamic radiation on FA skeleton (left)
EF	beta3_1	-0.138 [-0.278, -7.360e-03]*	Mean MD in posterior thalamic radiation on FA skeleton (left)
EF	c_prime_1	-0.339 [-0.462, -0.184]*	Mean MD in posterior thalamic radiation on FA skeleton (left)
EF	total_1	-0.36 [-0.494, -0.207]*	Mean MD in posterior thalamic radiation on FA skeleton (left)
EF	beta1_2	0.206 [0.0214, 0.399]*	Mean MD in posterior thalamic radiation on FA skeleton (left)
EF	beta4_2	0.201 [0.0878, 0.335]*	Mean MD in posterior thalamic radiation on FA skeleton (left)
EF	beta3_2	-0.166 [-0.324, -0.0208]*	Mean MD in posterior thalamic radiation on FA skeleton (left)
EF	ind3_2	-0.0339 [-0.0982, -7.337e-03]*	Mean MD in posterior thalamic radiation on FA skeleton (left)
EF	ind_2	-0.0331 [-0.115, -2.244e-03]*	Mean MD in posterior thalamic radiation on FA skeleton (left)
EF	total_2	-0.252 [-0.395, -0.0436]*	Mean MD in posterior thalamic radiation on FA skeleton (left)
EF	diff_beta4_2_0	0.205 [0.0567, 0.436]*	Mean MD in posterior thalamic radiation on FA skeleton (left)
EF	diff_ind3_2_0	-0.0324 [-0.106, -5.210e-03]*	Mean MD in posterior thalamic radiation on FA skeleton (left)
EF	diff_ind_2_0	-0.0395 [-0.136, -9.113e-04]*	Mean MD in posterior thalamic radiation on FA skeleton (left)
EF	diff_c_prime_1_0	-0.282 [-0.594, -0.0625]*	Mean MD in posterior thalamic radiation on FA skeleton (left)
EF	diff_total_1_0	-0.31 [-0.606, -0.0808]*	Mean MD in posterior thalamic radiation on FA skeleton (left)
VS	beta1_0	0.213 [0.0157, 0.61]*	Mean MD in posterior thalamic radiation on FA skeleton (left)
VS	c_prime_0	-0.245 [-0.431, -0.0717]*	Mean MD in posterior thalamic radiation on FA skeleton (left)
VS	total_0	-0.223 [-0.396, -0.0501]*	Mean MD in posterior thalamic radiation on FA skeleton (left)
VS	beta1_1	0.179 [0.0191, 0.388]*	Mean MD in posterior thalamic radiation on FA skeleton (left)
VS	beta3_1	-0.157 [-0.301, -0.0179]*	Mean MD in posterior thalamic radiation on FA skeleton (left)
VS	c_prime_1	-0.185 [-0.363, -2.920e-03]*	Mean MD in posterior thalamic radiation on FA skeleton (left)
VS	beta1_2	0.206 [0.0214, 0.399]*	Mean MD in posterior thalamic radiation on FA skeleton (left)
VS	beta4_2	0.201 [0.0878, 0.335]*	Mean MD in posterior thalamic radiation on FA skeleton (left)
VS	c_prime_2	-0.306 [-0.489, -0.102]*	Mean MD in posterior thalamic radiation on FA skeleton (left)
VS	total_2	-0.292 [-0.475, -0.112]*	Mean MD in posterior thalamic radiation on FA skeleton (left)
VS	diff_beta3_2_1	0.209 [9.037e-04, 0.431]*	Mean MD in posterior thalamic radiation on FA skeleton (left)
VS	diff_beta4_2_0	0.205 [0.0567, 0.436]*	Mean MD in posterior thalamic radiation on FA skeleton (left)
MMSE	beta1_0	0.213 [0.0157, 0.61]*	Mean MD in sagittal stratum on FA skeleton (right)
MMSE	beta3_0	-0.323 [-0.531, -0.121]*	Mean MD in sagittal stratum on FA skeleton (right)
MMSE	c_prime_0	-0.327 [-0.51, -0.154]*	Mean MD in sagittal stratum on FA skeleton (right)
MMSE	total_0	-0.287 [-0.498, -0.1]*	Mean MD in sagittal stratum on FA skeleton (right)
MMSE	beta1_1	0.179 [0.0191, 0.388]*	Mean MD in sagittal stratum on FA skeleton (right)
MMSE	beta4_1	0.256 [0.0339, 0.421]*	Mean MD in sagittal stratum on FA skeleton (right)
MMSE	c_prime_1	-0.487 [-0.656, -0.229]*	Mean MD in sagittal stratum on FA skeleton (right)
MMSE	ind3_1	-0.0336 [-0.106, -6.975e-04]*	Mean MD in sagittal stratum on FA skeleton (right)
MMSE	ind_1	-0.0468 [-0.127, -6.372e-03]*	Mean MD in sagittal stratum on FA skeleton (right)
MMSE	total_1	-0.533 [-0.705, -0.268]*	Mean MD in sagittal stratum on FA skeleton (right)
MMSE	beta1_2	0.206 [0.0214, 0.399]*	Mean MD in sagittal stratum on FA skeleton (right)
MMSE	beta2_2	0.338 [0.111, 0.62]*	Mean MD in sagittal stratum on FA skeleton (right)
MMSE	beta3_2	-0.211 [-0.444, -0.0379]*	Mean MD in sagittal stratum on FA skeleton (right)
MMSE	ind1_2	-0.0138 [-0.069, -2.645e-03]*	Mean MD in sagittal stratum on FA skeleton (right)
MMSE	ind3_2	-0.0288 [-0.0894, -8.790e-04]*	Mean MD in sagittal stratum on FA skeleton (right)
MMSE	ind_2	-0.046 [-0.139, -3.210e-03]*	Mean MD in sagittal stratum on FA skeleton (right)
MMSE	total_2	-0.262 [-0.469, -0.0361]*	Mean MD in sagittal stratum on FA skeleton (right)
MMSE	diff_beta2_2_0	0.285 [0.0536, 0.715]*	Mean MD in sagittal stratum on FA skeleton (right)
MMSE	diff_beta2_2_1	0.341 [0.0569, 0.691]*	Mean MD in sagittal stratum on FA skeleton (right)
MMSE	diff_beta4_1_0	0.286 [0.028, 0.52]*	Mean MD in sagittal stratum on FA skeleton (right)
MMSE	diff_beta5_1_0	-0.16 [-0.491, -3.522e-03]*	Mean MD in sagittal stratum on FA skeleton (right)
MMSE	diff_ind1_2_1	-0.0133 [-0.0683, -2.076e-03]*	Mean MD in sagittal stratum on FA skeleton (right)
MMSE	diff_ind_1_0	-0.0871 [-0.212, -4.501e-03]*	Mean MD in sagittal stratum on FA skeleton (right)
MMSE	diff_ind_2_0	-0.0863 [-0.226, -5.207e-03]*	Mean MD in sagittal stratum on FA skeleton (right)
MoCA	beta1_0	0.213 [0.0157, 0.61]*	Mean MD in sagittal stratum on FA skeleton (right)
MoCA	beta3_0	-0.284 [-0.416, -0.139]*	Mean MD in sagittal stratum on FA skeleton (right)
MoCA	c_prime_0	-0.245 [-0.376, -0.0854]*	Mean MD in sagittal stratum on FA skeleton (right)
MoCA	total_0	-0.22 [-0.363, -0.0657]*	Mean MD in sagittal stratum on FA skeleton (right)
MoCA	beta1_1	0.179 [0.0191, 0.388]*	Mean MD in sagittal stratum on FA skeleton (right)
MoCA	beta4_1	0.256 [0.0339, 0.421]*	Mean MD in sagittal stratum on FA skeleton (right)
MoCA	beta3_1	-0.186 [-0.326, -0.0491]*	Mean MD in sagittal stratum on FA skeleton (right)

MoCA	c_prime_1	-0.42 [-0.559, -0.189]*	Mean MD in sagittal stratum on FA skeleton (right)
MoCA	ind3_1	-0.045 [-0.1, -8.273e-03]*	Mean MD in sagittal stratum on FA skeleton (right)
MoCA	ind_1	-0.0526 [-0.119, -0.01]*	Mean MD in sagittal stratum on FA skeleton (right)
MoCA	total_1	-0.472 [-0.612, -0.227]*	Mean MD in sagittal stratum on FA skeleton (right)
MoCA	beta1_2	0.206 [0.0214, 0.399]*	Mean MD in sagittal stratum on FA skeleton (right)
MoCA	beta2_2	0.338 [0.111, 0.62]*	Mean MD in sagittal stratum on FA skeleton (right)
MoCA	c_prime_2	-0.333 [-0.488, -0.135]*	Mean MD in sagittal stratum on FA skeleton (right)
MoCA	ind1_2	-7.518e-03 [-0.0441, -3.588e-04]*	Mean MD in sagittal stratum on FA skeleton (right)
MoCA	total_2	-0.361 [-0.526, -0.184]*	Mean MD in sagittal stratum on FA skeleton (right)
MoCA	diff_beta2_2_0	0.285 [0.0536, 0.715]*	Mean MD in sagittal stratum on FA skeleton (right)
MoCA	diff_beta2_2_1	0.341 [0.0569, 0.691]*	Mean MD in sagittal stratum on FA skeleton (right)
MoCA	diff_beta4_1_0	0.286 [0.028, 0.52]*	Mean MD in sagittal stratum on FA skeleton (right)
MoCA	diff_ind3_1_0	-0.0551 [-0.132, -3.058e-04]*	Mean MD in sagittal stratum on FA skeleton (right)
MoCA	diff_ind_1_0	-0.0784 [-0.171, -0.0103]*	Mean MD in sagittal stratum on FA skeleton (right)
EM	beta1_0	0.213 [0.0157, 0.61]*	Mean MD in sagittal stratum on FA skeleton (right)
EM	beta3_0	-0.172 [-0.306, -0.0103]*	Mean MD in sagittal stratum on FA skeleton (right)
EM	c_prime_0	-0.243 [-0.367, -0.153]*	Mean MD in sagittal stratum on FA skeleton (right)
EM	total_0	-0.25 [-0.352, -0.146]*	Mean MD in sagittal stratum on FA skeleton (right)
EM	beta1_1	0.179 [0.0191, 0.388]*	Mean MD in sagittal stratum on FA skeleton (right)
EM	beta4_1	0.256 [0.0339, 0.421]*	Mean MD in sagittal stratum on FA skeleton (right)
EM	c_prime_1	-0.163 [-0.295, -7.829e-03]*	Mean MD in sagittal stratum on FA skeleton (right)
EM	total_1	-0.204 [-0.332, -0.0676]*	Mean MD in sagittal stratum on FA skeleton (right)
EM	beta1_2	0.206 [0.0214, 0.399]*	Mean MD in sagittal stratum on FA skeleton (right)
EM	beta2_2	0.338 [0.111, 0.62]*	Mean MD in sagittal stratum on FA skeleton (right)
EM	diff_beta2_2_0	0.285 [0.0536, 0.715]*	Mean MD in sagittal stratum on FA skeleton (right)
EM	diff_beta2_2_1	0.341 [0.0569, 0.691]*	Mean MD in sagittal stratum on FA skeleton (right)
EM	diff_beta4_1_0	0.286 [0.028, 0.52]*	Mean MD in sagittal stratum on FA skeleton (right)
ISP	beta1_0	0.213 [0.0157, 0.61]*	Mean MD in sagittal stratum on FA skeleton (right)
ISP	c_prime_0	-0.187 [-0.308, -3.982e-03]*	Mean MD in sagittal stratum on FA skeleton (right)
ISP	total_0	-0.169 [-0.307, -7.943e-03]*	Mean MD in sagittal stratum on FA skeleton (right)
ISP	beta1_1	0.179 [0.0191, 0.388]*	Mean MD in sagittal stratum on FA skeleton (right)
ISP	beta4_1	0.256 [0.0339, 0.421]*	Mean MD in sagittal stratum on FA skeleton (right)
ISP	c_prime_1	-0.412 [-0.533, -0.257]*	Mean MD in sagittal stratum on FA skeleton (right)
ISP	total_1	-0.399 [-0.514, -0.25]*	Mean MD in sagittal stratum on FA skeleton (right)
ISP	beta1_2	0.206 [0.0214, 0.399]*	Mean MD in sagittal stratum on FA skeleton (right)
ISP	beta2_2	0.338 [0.111, 0.62]*	Mean MD in sagittal stratum on FA skeleton (right)
ISP	c_prime_2	-0.29 [-0.425, -0.122]*	Mean MD in sagittal stratum on FA skeleton (right)
ISP	total_2	-0.267 [-0.389, -0.118]*	Mean MD in sagittal stratum on FA skeleton (right)
ISP	diff_beta2_2_0	0.285 [0.0536, 0.715]*	Mean MD in sagittal stratum on FA skeleton (right)
ISP	diff_beta2_2_1	0.341 [0.0569, 0.691]*	Mean MD in sagittal stratum on FA skeleton (right)
ISP	diff_beta4_1_0	0.286 [0.028, 0.52]*	Mean MD in sagittal stratum on FA skeleton (right)
ISP	diff_c_prime_1_0	-0.224 [-0.429, -0.0256]*	Mean MD in sagittal stratum on FA skeleton (right)
ISP	diff_total_1_0	-0.23 [-0.426, -0.0261]*	Mean MD in sagittal stratum on FA skeleton (right)
LF	beta1_0	0.213 [0.0157, 0.61]*	Mean MD in sagittal stratum on FA skeleton (right)
LF	c_prime_0	-0.239 [-0.372, -0.0818]*	Mean MD in sagittal stratum on FA skeleton (right)
LF	total_0	-0.233 [-0.359, -0.0909]*	Mean MD in sagittal stratum on FA skeleton (right)
LF	beta1_1	0.179 [0.0191, 0.388]*	Mean MD in sagittal stratum on FA skeleton (right)
LF	beta4_1	0.256 [0.0339, 0.421]*	Mean MD in sagittal stratum on FA skeleton (right)
LF	c_prime_1	-0.204 [-0.349, -0.0711]*	Mean MD in sagittal stratum on FA skeleton (right)
LF	ind3_1	-0.0376 [-0.0878, -3.773e-04]*	Mean MD in sagittal stratum on FA skeleton (right)
LF	total_1	-0.23 [-0.369, -0.1]*	Mean MD in sagittal stratum on FA skeleton (right)
LF	beta1_2	0.206 [0.0214, 0.399]*	Mean MD in sagittal stratum on FA skeleton (right)
LF	beta2_2	0.338 [0.111, 0.62]*	Mean MD in sagittal stratum on FA skeleton (right)
LF	c_prime_2	-0.296 [-0.439, -0.119]*	Mean MD in sagittal stratum on FA skeleton (right)
LF	total_2	-0.294 [-0.436, -0.133]*	Mean MD in sagittal stratum on FA skeleton (right)
LF	diff_beta2_2_0	0.285 [0.0536, 0.715]*	Mean MD in sagittal stratum on FA skeleton (right)
LF	diff_beta2_2_1	0.341 [0.0569, 0.691]*	Mean MD in sagittal stratum on FA skeleton (right)
LF	diff_beta4_1_0	0.286 [0.028, 0.52]*	Mean MD in sagittal stratum on FA skeleton (right)
EF	beta1_0	0.213 [0.0157, 0.61]*	Mean MD in sagittal stratum on FA skeleton (right)
EF	beta3_0	-0.157 [-0.283, -0.0305]*	Mean MD in sagittal stratum on FA skeleton (right)
EF	beta1_1	0.179 [0.0191, 0.388]*	Mean MD in sagittal stratum on FA skeleton (right)
EF	beta4_1	0.256 [0.0339, 0.421]*	Mean MD in sagittal stratum on FA skeleton (right)
EF	c_prime_1	-0.342 [-0.471, -0.186]*	Mean MD in sagittal stratum on FA skeleton (right)
EF	total_1	-0.36 [-0.494, -0.207]*	Mean MD in sagittal stratum on FA skeleton (right)
EF	beta1_2	0.206 [0.0214, 0.399]*	Mean MD in sagittal stratum on FA skeleton (right)
EF	beta2_2	0.338 [0.111, 0.62]*	Mean MD in sagittal stratum on FA skeleton (right)
EF	c_prime_2	-0.257 [-0.411, -0.0253]*	Mean MD in sagittal stratum on FA skeleton (right)
EF	total_2	-0.252 [-0.395, -0.0436]*	Mean MD in sagittal stratum on FA skeleton (right)
EF	diff_beta2_2_0	0.285 [0.0536, 0.715]*	Mean MD in sagittal stratum on FA skeleton (right)
EF	diff_beta2_2_1	0.341 [0.0569, 0.691]*	Mean MD in sagittal stratum on FA skeleton (right)
EF	diff_beta4_1_0	0.286 [0.028, 0.52]*	Mean MD in sagittal stratum on FA skeleton (right)
EF	diff_c_prime_1_0	-0.278 [-0.59, -0.0552]*	Mean MD in sagittal stratum on FA skeleton (right)
EF	diff_total_1_0	-0.31 [-0.606, -0.0808]*	Mean MD in sagittal stratum on FA skeleton (right)
VS	beta1_0	0.213 [0.0157, 0.61]*	Mean MD in sagittal stratum on FA skeleton (right)
VS	beta3_0	-0.185 [-0.326, -0.0257]*	Mean MD in sagittal stratum on FA skeleton (right)
VS	c_prime_0	-0.251 [-0.424, -0.0726]*	Mean MD in sagittal stratum on FA skeleton (right)
VS	total_0	-0.223 [-0.396, -0.0501]*	Mean MD in sagittal stratum on FA skeleton (right)
VS	beta1_1	0.179 [0.0191, 0.388]*	Mean MD in sagittal stratum on FA skeleton (right)
VS	beta4_1	0.256 [0.0339, 0.421]*	Mean MD in sagittal stratum on FA skeleton (right)
VS	beta3_1	-0.225 [-0.386, -0.0658]*	Mean MD in sagittal stratum on FA skeleton (right)
VS	ind3_1	-0.0564 [-0.121, -8.821e-03]*	Mean MD in sagittal stratum on FA skeleton (right)
VS	beta1_2	0.206 [0.0214, 0.399]*	Mean MD in sagittal stratum on FA skeleton (right)
VS	beta2_2	0.338 [0.111, 0.62]*	Mean MD in sagittal stratum on FA skeleton (right)
VS	c_prime_2	-0.293 [-0.469, -0.0917]*	Mean MD in sagittal stratum on FA skeleton (right)
VS	total_2	-0.292 [-0.475, -0.112]*	Mean MD in sagittal stratum on FA skeleton (right)
VS	diff_beta2_2_0	0.285 [0.0536, 0.715]*	Mean MD in sagittal stratum on FA skeleton (right)
VS	diff_beta2_2_1	0.341 [0.0569, 0.691]*	Mean MD in sagittal stratum on FA skeleton (right)
VS	diff_beta4_1_0	0.286 [0.028, 0.52]*	Mean MD in sagittal stratum on FA skeleton (right)
VS	diff_ind3_1_0	-0.0618 [-0.139, -7.710e-03]*	Mean MD in sagittal stratum on FA skeleton (right)
MMSE	beta1_0	0.213 [0.0157, 0.61]*	Mean MD in sagittal stratum on FA skeleton (left)
MMSE	beta3_0	-0.228 [-0.464, -0.042]*	Mean MD in sagittal stratum on FA skeleton (left)
MMSE	c_prime_0	-0.325 [-0.525, -0.146]*	Mean MD in sagittal stratum on FA skeleton (left)
MMSE	total_0	-0.287 [-0.498, -0.1]*	Mean MD in sagittal stratum on FA skeleton (left)
MMSE	beta1_1	0.179 [0.0191, 0.388]*	Mean MD in sagittal stratum on FA skeleton (left)
MMSE	beta4_1	0.235 [0.0534, 0.472]*	Mean MD in sagittal stratum on FA skeleton (left)
MMSE	beta3_1	-0.22 [-0.477, -0.05]*	Mean MD in sagittal stratum on FA skeleton (left)

MMSE	c_prime_1	-0.47 [-0.655, -0.205]*	Mean MD in sagittal stratum on FA skeleton (left)
MMSE	ind3_1	-0.0506 [-0.175, -9.052e-03]*	Mean MD in sagittal stratum on FA skeleton (left)
MMSE	ind_1	-0.0638 [-0.21, -9.408e-03]*	Mean MD in sagittal stratum on FA skeleton (left)
MMSE	total_1	-0.533 [-0.705, -0.268]*	Mean MD in sagittal stratum on FA skeleton (left)
MMSE	beta1_2	0.206 [0.0214, 0.399]*	Mean MD in sagittal stratum on FA skeleton (left)
MMSE	beta2_2	0.178 [8.466e-04, 0.39]*	Mean MD in sagittal stratum on FA skeleton (left)
MMSE	beta4_2	0.175 [0.0208, 0.307]*	Mean MD in sagittal stratum on FA skeleton (left)
MMSE	ind1_2	-6.508e-03 [-0.0412, -4.992e-04]*	Mean MD in sagittal stratum on FA skeleton (left)
MMSE	ind3_2	-0.0294 [-0.0963, -2.844e-03]*	Mean MD in sagittal stratum on FA skeleton (left)
MMSE	ind_2	-0.0465 [-0.15, -3.972e-03]*	Mean MD in sagittal stratum on FA skeleton (left)
MMSE	total_2	-0.262 [-0.469, -0.0361]*	Mean MD in sagittal stratum on FA skeleton (left)
MMSE	diff_beta4_1_0	0.283 [0.0686, 0.58]*	Mean MD in sagittal stratum on FA skeleton (left)
MMSE	diff_beta4_2_0	0.223 [0.0401, 0.447]*	Mean MD in sagittal stratum on FA skeleton (left)
MMSE	diff_ind3_1_0	-0.057 [-0.187, -0.0124]*	Mean MD in sagittal stratum on FA skeleton (left)
MMSE	diff_ind3_2_0	-0.0358 [-0.117, -4.393e-03]*	Mean MD in sagittal stratum on FA skeleton (left)
MMSE	diff_ind_1_0	-0.102 [-0.263, -0.0175]*	Mean MD in sagittal stratum on FA skeleton (left)
MMSE	diff_ind_2_0	-0.0844 [-0.236, -0.0152]*	Mean MD in sagittal stratum on FA skeleton (left)
MoCA	beta1_0	0.213 [0.0157, 0.61]*	Mean MD in sagittal stratum on FA skeleton (left)
MoCA	beta3_0	-0.263 [-0.406, -0.114]*	Mean MD in sagittal stratum on FA skeleton (left)
MoCA	c_prime_0	-0.246 [-0.391, -0.0966]*	Mean MD in sagittal stratum on FA skeleton (left)
MoCA	total_0	-0.22 [-0.363, -0.0657]*	Mean MD in sagittal stratum on FA skeleton (left)
MoCA	beta1_1	0.179 [0.0191, 0.388]*	Mean MD in sagittal stratum on FA skeleton (left)
MoCA	beta4_1	0.235 [0.0534, 0.472]*	Mean MD in sagittal stratum on FA skeleton (left)
MoCA	beta3_1	-0.23 [-0.412, -0.095]*	Mean MD in sagittal stratum on FA skeleton (left)
MoCA	c_prime_1	-0.412 [-0.562, -0.174]*	Mean MD in sagittal stratum on FA skeleton (left)
MoCA	ind3_1	-0.0526 [-0.143, -0.0132]*	Mean MD in sagittal stratum on FA skeleton (left)
MoCA	ind_1	-0.0602 [-0.179, -0.0101]*	Mean MD in sagittal stratum on FA skeleton (left)
MoCA	total_1	-0.472 [-0.612, -0.227]*	Mean MD in sagittal stratum on FA skeleton (left)
MoCA	beta1_2	0.206 [0.0214, 0.399]*	Mean MD in sagittal stratum on FA skeleton (left)
MoCA	beta2_2	0.178 [8.466e-04, 0.39]*	Mean MD in sagittal stratum on FA skeleton (left)
MoCA	beta4_2	0.175 [0.0208, 0.307]*	Mean MD in sagittal stratum on FA skeleton (left)
MoCA	c_prime_2	-0.322 [-0.474, -0.13]*	Mean MD in sagittal stratum on FA skeleton (left)
MoCA	ind1_2	-5.454e-03 [-0.0345, -2.950e-04]*	Mean MD in sagittal stratum on FA skeleton (left)
MoCA	ind3_2	-0.0252 [-0.0843, -1.707e-03]*	Mean MD in sagittal stratum on FA skeleton (left)
MoCA	ind_2	-0.0387 [-0.121, -6.484e-04]*	Mean MD in sagittal stratum on FA skeleton (left)
MoCA	total_2	-0.361 [-0.526, -0.184]*	Mean MD in sagittal stratum on FA skeleton (left)
MoCA	diff_beta4_1_0	0.283 [0.0686, 0.58]*	Mean MD in sagittal stratum on FA skeleton (left)
MoCA	diff_beta4_2_0	0.223 [0.0401, 0.447]*	Mean MD in sagittal stratum on FA skeleton (left)
MoCA	diff_ind3_1_0	-0.0638 [-0.162, -0.0135]*	Mean MD in sagittal stratum on FA skeleton (left)
MoCA	diff_ind3_2_0	-0.0364 [-0.113, -4.960e-04]*	Mean MD in sagittal stratum on FA skeleton (left)
MoCA	diff_ind_1_0	-0.0871 [-0.211, -0.0205]*	Mean MD in sagittal stratum on FA skeleton (left)
MoCA	diff_ind_2_0	-0.0656 [-0.177, -0.0136]*	Mean MD in sagittal stratum on FA skeleton (left)
EM	beta1_0	0.213 [0.0157, 0.61]*	Mean MD in sagittal stratum on FA skeleton (left)
EM	c_prime_0	-0.244 [-0.372, -0.155]*	Mean MD in sagittal stratum on FA skeleton (left)
EM	total_0	-0.25 [-0.352, -0.146]*	Mean MD in sagittal stratum on FA skeleton (left)
EM	beta1_1	0.179 [0.0191, 0.388]*	Mean MD in sagittal stratum on FA skeleton (left)
EM	beta4_1	0.235 [0.0534, 0.472]*	Mean MD in sagittal stratum on FA skeleton (left)
EM	ind_1	-0.0481 [-0.142, -2.760e-03]*	Mean MD in sagittal stratum on FA skeleton (left)
EM	total_1	-0.204 [-0.332, -0.0676]*	Mean MD in sagittal stratum on FA skeleton (left)
EM	beta1_2	0.206 [0.0214, 0.399]*	Mean MD in sagittal stratum on FA skeleton (left)
EM	beta2_2	0.178 [8.466e-04, 0.39]*	Mean MD in sagittal stratum on FA skeleton (left)
EM	beta4_2	0.175 [0.0208, 0.307]*	Mean MD in sagittal stratum on FA skeleton (left)
EM	diff_beta4_1_0	0.283 [0.0686, 0.58]*	Mean MD in sagittal stratum on FA skeleton (left)
EM	diff_beta4_2_0	0.223 [0.0401, 0.447]*	Mean MD in sagittal stratum on FA skeleton (left)
ISP	beta1_0	0.213 [0.0157, 0.61]*	Mean MD in sagittal stratum on FA skeleton (left)
ISP	c_prime_0	-0.187 [-0.316, -0.0121]*	Mean MD in sagittal stratum on FA skeleton (left)
ISP	total_0	-0.169 [-0.307, -7.943e-03]*	Mean MD in sagittal stratum on FA skeleton (left)
ISP	beta1_1	0.179 [0.0191, 0.388]*	Mean MD in sagittal stratum on FA skeleton (left)
ISP	beta4_1	0.235 [0.0534, 0.472]*	Mean MD in sagittal stratum on FA skeleton (left)
ISP	c_prime_1	-0.398 [-0.51, -0.245]*	Mean MD in sagittal stratum on FA skeleton (left)
ISP	total_1	-0.399 [-0.514, -0.25]*	Mean MD in sagittal stratum on FA skeleton (left)
ISP	beta1_2	0.206 [0.0214, 0.399]*	Mean MD in sagittal stratum on FA skeleton (left)
ISP	beta2_2	0.178 [8.466e-04, 0.39]*	Mean MD in sagittal stratum on FA skeleton (left)
ISP	beta4_2	0.175 [0.0208, 0.307]*	Mean MD in sagittal stratum on FA skeleton (left)
ISP	c_prime_2	-0.278 [-0.41, -0.117]*	Mean MD in sagittal stratum on FA skeleton (left)
ISP	total_2	-0.267 [-0.389, -0.118]*	Mean MD in sagittal stratum on FA skeleton (left)
ISP	diff_beta4_1_0	0.283 [0.0686, 0.58]*	Mean MD in sagittal stratum on FA skeleton (left)
ISP	diff_beta4_2_0	0.223 [0.0401, 0.447]*	Mean MD in sagittal stratum on FA skeleton (left)
ISP	diff_c_prime_1_0	-0.211 [-0.414, -0.0134]*	Mean MD in sagittal stratum on FA skeleton (left)
ISP	diff_total_1_0	-0.23 [-0.426, -0.0261]*	Mean MD in sagittal stratum on FA skeleton (left)
LF	beta1_0	0.213 [0.0157, 0.61]*	Mean MD in sagittal stratum on FA skeleton (left)
LF	c_prime_0	-0.24 [-0.375, -0.0891]*	Mean MD in sagittal stratum on FA skeleton (left)
LF	total_0	-0.233 [-0.359, -0.0909]*	Mean MD in sagittal stratum on FA skeleton (left)
LF	beta1_1	0.179 [0.0191, 0.388]*	Mean MD in sagittal stratum on FA skeleton (left)
LF	beta4_1	0.235 [0.0534, 0.472]*	Mean MD in sagittal stratum on FA skeleton (left)
LF	c_prime_1	-0.216 [-0.361, -0.0773]*	Mean MD in sagittal stratum on FA skeleton (left)
LF	total_1	-0.23 [-0.369, -0.1]*	Mean MD in sagittal stratum on FA skeleton (left)
LF	beta1_2	0.206 [0.0214, 0.399]*	Mean MD in sagittal stratum on FA skeleton (left)
LF	beta2_2	0.178 [8.466e-04, 0.39]*	Mean MD in sagittal stratum on FA skeleton (left)
LF	beta4_2	0.175 [0.0208, 0.307]*	Mean MD in sagittal stratum on FA skeleton (left)
LF	c_prime_2	-0.297 [-0.441, -0.118]*	Mean MD in sagittal stratum on FA skeleton (left)
LF	total_2	-0.294 [-0.436, -0.133]*	Mean MD in sagittal stratum on FA skeleton (left)
LF	diff_beta4_1_0	0.283 [0.0686, 0.58]*	Mean MD in sagittal stratum on FA skeleton (left)
LF	diff_beta4_2_0	0.223 [0.0401, 0.447]*	Mean MD in sagittal stratum on FA skeleton (left)
EF	beta1_0	0.213 [0.0157, 0.61]*	Mean MD in sagittal stratum on FA skeleton (left)
EF	beta3_0	-0.146 [-0.292, -6.628e-03]*	Mean MD in sagittal stratum on FA skeleton (left)
EF	beta1_1	0.179 [0.0191, 0.388]*	Mean MD in sagittal stratum on FA skeleton (left)
EF	beta4_1	0.235 [0.0534, 0.472]*	Mean MD in sagittal stratum on FA skeleton (left)
EF	beta3_1	-0.163 [-0.312, -0.0552]*	Mean MD in sagittal stratum on FA skeleton (left)
EF	c_prime_1	-0.316 [-0.436, -0.166]*	Mean MD in sagittal stratum on FA skeleton (left)
EF	ind3_1	-0.0394 [-0.121, -6.118e-03]*	Mean MD in sagittal stratum on FA skeleton (left)
EF	ind_1	-0.0436 [-0.15, -1.421e-03]*	Mean MD in sagittal stratum on FA skeleton (left)
EF	total_1	-0.36 [-0.494, -0.207]*	Mean MD in sagittal stratum on FA skeleton (left)
EF	beta1_2	0.206 [0.0214, 0.399]*	Mean MD in sagittal stratum on FA skeleton (left)
EF	beta2_2	0.178 [8.466e-04, 0.39]*	Mean MD in sagittal stratum on FA skeleton (left)
EF	beta4_2	0.175 [0.0208, 0.307]*	Mean MD in sagittal stratum on FA skeleton (left)

EF	c_prime_2	-0.237 [-0.393, -0.0154]*	Mean MD in sagittal stratum on FA skeleton (left)
EF	total_2	-0.252 [-0.395, -0.0436]*	Mean MD in sagittal stratum on FA skeleton (left)
EF	diff_beta4_1_0	0.283 [0.0686, 0.58]*	Mean MD in sagittal stratum on FA skeleton (left)
EF	diff_beta4_2_0	0.223 [0.0401, 0.447]*	Mean MD in sagittal stratum on FA skeleton (left)
EF	diff_ind3_1_0	-0.0443 [-0.131, -8.646e-03]*	Mean MD in sagittal stratum on FA skeleton (left)
EF	diff_ind_1_0	-0.0564 [-0.164, -5.673e-03]*	Mean MD in sagittal stratum on FA skeleton (left)
EF	diff_c_prime_1_0	-0.253 [-0.554, -0.0309]*	Mean MD in sagittal stratum on FA skeleton (left)
EF	diff_total_1_0	-0.31 [-0.606, -0.0808]*	Mean MD in sagittal stratum on FA skeleton (left)
VS	beta1_0	0.213 [0.0157, 0.61]*	Mean MD in sagittal stratum on FA skeleton (left)
VS	c_prime_0	-0.25 [-0.432, -0.0737]*	Mean MD in sagittal stratum on FA skeleton (left)
VS	total_0	-0.223 [-0.396, -0.0501]*	Mean MD in sagittal stratum on FA skeleton (left)
VS	beta1_1	0.179 [0.0191, 0.388]*	Mean MD in sagittal stratum on FA skeleton (left)
VS	beta4_1	0.235 [0.0534, 0.472]*	Mean MD in sagittal stratum on FA skeleton (left)
VS	beta3_1	-0.203 [-0.361, -0.0159]*	Mean MD in sagittal stratum on FA skeleton (left)
VS	ind3_1	-0.048 [-0.13, -4.202e-03]*	Mean MD in sagittal stratum on FA skeleton (left)
VS	beta1_2	0.206 [0.0214, 0.399]*	Mean MD in sagittal stratum on FA skeleton (left)
VS	beta2_2	0.178 [8.466e-04, 0.39]*	Mean MD in sagittal stratum on FA skeleton (left)
VS	beta4_2	0.175 [0.0208, 0.307]*	Mean MD in sagittal stratum on FA skeleton (left)
VS	c_prime_2	-0.291 [-0.476, -0.0875]*	Mean MD in sagittal stratum on FA skeleton (left)
VS	total_2	-0.292 [-0.475, -0.112]*	Mean MD in sagittal stratum on FA skeleton (left)
VS	diff_beta4_1_0	0.283 [0.0686, 0.58]*	Mean MD in sagittal stratum on FA skeleton (left)
VS	diff_beta4_2_0	0.223 [0.0401, 0.447]*	Mean MD in sagittal stratum on FA skeleton (left)
VS	diff_ind3_1_0	-0.0525 [-0.14, -6.241e-03]*	Mean MD in sagittal stratum on FA skeleton (left)
MMSE	beta1_0	0.213 [0.0157, 0.61]*	Mean MD in cingulum cingulate gyrus on FA skeleton (left)
MMSE	beta3_0	-0.226 [-0.427, -0.0818]*	Mean MD in cingulum cingulate gyrus on FA skeleton (left)
MMSE	c_prime_0	-0.338 [-0.535, -0.153]*	Mean MD in cingulum cingulate gyrus on FA skeleton (left)
MMSE	total_0	-0.287 [-0.498, -0.1]*	Mean MD in cingulum cingulate gyrus on FA skeleton (left)
MMSE	beta1_1	0.179 [0.0191, 0.388]*	Mean MD in cingulum cingulate gyrus on FA skeleton (left)
MMSE	beta4_1	0.178 [0.0209, 0.336]*	Mean MD in cingulum cingulate gyrus on FA skeleton (left)
MMSE	c_prime_1	-0.503 [-0.682, -0.228]*	Mean MD in cingulum cingulate gyrus on FA skeleton (left)
MMSE	total_1	-0.533 [-0.705, -0.268]*	Mean MD in cingulum cingulate gyrus on FA skeleton (left)
MMSE	beta1_2	0.206 [0.0214, 0.399]*	Mean MD in cingulum cingulate gyrus on FA skeleton (left)
MMSE	beta2_2	0.182 [0.0382, 0.326]*	Mean MD in cingulum cingulate gyrus on FA skeleton (left)
MMSE	beta4_2	0.226 [0.0507, 0.386]*	Mean MD in cingulum cingulate gyrus on FA skeleton (left)
MMSE	beta3_2	-0.182 [-0.36, -0.0554]*	Mean MD in cingulum cingulate gyrus on FA skeleton (left)
MMSE	ind1_2	-6.450e-03 [-0.0304, -1.454e-03]*	Mean MD in cingulum cingulate gyrus on FA skeleton (left)
MMSE	ind3_2	-0.0397 [-0.11, -0.0101]*	Mean MD in cingulum cingulate gyrus on FA skeleton (left)
MMSE	ind_2	-0.0568 [-0.163, -0.0149]*	Mean MD in cingulum cingulate gyrus on FA skeleton (left)
MMSE	total_2	-0.262 [-0.469, -0.0361]*	Mean MD in cingulum cingulate gyrus on FA skeleton (left)
MMSE	diff_beta4_1_0	0.269 [0.0357, 0.495]*	Mean MD in cingulum cingulate gyrus on FA skeleton (left)
MMSE	diff_beta4_2_0	0.317 [0.0668, 0.541]*	Mean MD in cingulum cingulate gyrus on FA skeleton (left)
MMSE	diff_ind3_2_0	-0.0595 [-0.144, -0.0148]*	Mean MD in cingulum cingulate gyrus on FA skeleton (left)
MMSE	diff_ind_1_0	-0.0812 [-0.195, -4.294e-03]*	Mean MD in cingulum cingulate gyrus on FA skeleton (left)
MMSE	diff_ind_2_0	-0.108 [-0.25, -0.0302]*	Mean MD in cingulum cingulate gyrus on FA skeleton (left)
MoCA	beta1_0	0.213 [0.0157, 0.61]*	Mean MD in cingulum cingulate gyrus on FA skeleton (left)
MoCA	beta3_0	-0.18 [-0.336, -0.0352]*	Mean MD in cingulum cingulate gyrus on FA skeleton (left)
MoCA	c_prime_0	-0.251 [-0.397, -0.0902]*	Mean MD in cingulum cingulate gyrus on FA skeleton (left)
MoCA	total_0	-0.22 [-0.363, -0.0657]*	Mean MD in cingulum cingulate gyrus on FA skeleton (left)
MoCA	beta1_1	0.179 [0.0191, 0.388]*	Mean MD in cingulum cingulate gyrus on FA skeleton (left)
MoCA	beta4_1	0.178 [0.0209, 0.336]*	Mean MD in cingulum cingulate gyrus on FA skeleton (left)
MoCA	beta3_1	-0.17 [-0.275, -0.0521]*	Mean MD in cingulum cingulate gyrus on FA skeleton (left)
MoCA	c_prime_1	-0.433 [-0.581, -0.184]*	Mean MD in cingulum cingulate gyrus on FA skeleton (left)
MoCA	ind3_1	-0.0314 [-0.0774, -2.858e-03]*	Mean MD in cingulum cingulate gyrus on FA skeleton (left)
MoCA	total_1	-0.472 [-0.612, -0.227]*	Mean MD in cingulum cingulate gyrus on FA skeleton (left)
MoCA	beta1_2	0.206 [0.0214, 0.399]*	Mean MD in cingulum cingulate gyrus on FA skeleton (left)
MoCA	beta2_2	0.182 [0.0382, 0.326]*	Mean MD in cingulum cingulate gyrus on FA skeleton (left)
MoCA	beta4_2	0.226 [0.0507, 0.386]*	Mean MD in cingulum cingulate gyrus on FA skeleton (left)
MoCA	c_prime_2	-0.329 [-0.493, -0.141]*	Mean MD in cingulum cingulate gyrus on FA skeleton (left)
MoCA	ind1_2	-2.847e-03 [-0.0188, -1.284e-04]*	Mean MD in cingulum cingulate gyrus on FA skeleton (left)
MoCA	total_2	-0.361 [-0.526, -0.184]*	Mean MD in cingulum cingulate gyrus on FA skeleton (left)
MoCA	diff_beta4_1_0	0.269 [0.0357, 0.495]*	Mean MD in cingulum cingulate gyrus on FA skeleton (left)
MoCA	diff_beta4_2_0	0.317 [0.0668, 0.541]*	Mean MD in cingulum cingulate gyrus on FA skeleton (left)
MoCA	diff_ind3_1_0	-0.0471 [-0.107, -3.519e-03]*	Mean MD in cingulum cingulate gyrus on FA skeleton (left)
MoCA	diff_ind_1_0	-0.0704 [-0.159, -8.256e-03]*	Mean MD in cingulum cingulate gyrus on FA skeleton (left)
MoCA	diff_ind_2_0	-0.063 [-0.17, -7.105e-03]*	Mean MD in cingulum cingulate gyrus on FA skeleton (left)
EM	beta1_0	0.213 [0.0157, 0.61]*	Mean MD in cingulum cingulate gyrus on FA skeleton (left)
EM	c_prime_0	-0.246 [-0.366, -0.151]*	Mean MD in cingulum cingulate gyrus on FA skeleton (left)
EM	total_0	-0.25 [-0.352, -0.146]*	Mean MD in cingulum cingulate gyrus on FA skeleton (left)
EM	beta1_1	0.179 [0.0191, 0.388]*	Mean MD in cingulum cingulate gyrus on FA skeleton (left)
EM	beta4_1	0.178 [0.0209, 0.336]*	Mean MD in cingulum cingulate gyrus on FA skeleton (left)
EM	beta3_1	-0.172 [-0.303, -0.0312]*	Mean MD in cingulum cingulate gyrus on FA skeleton (left)
EM	c_prime_1	-0.155 [-0.289, -2.588e-03]*	Mean MD in cingulum cingulate gyrus on FA skeleton (left)
EM	ind3_1	-0.0291 [-0.0766, -3.989e-03]*	Mean MD in cingulum cingulate gyrus on FA skeleton (left)
EM	ind_1	-0.0485 [-0.127, -8.712e-03]*	Mean MD in cingulum cingulate gyrus on FA skeleton (left)
EM	total_1	-0.204 [-0.332, -0.0676]*	Mean MD in cingulum cingulate gyrus on FA skeleton (left)
EM	beta1_2	0.206 [0.0214, 0.399]*	Mean MD in cingulum cingulate gyrus on FA skeleton (left)
EM	beta2_2	0.182 [0.0382, 0.326]*	Mean MD in cingulum cingulate gyrus on FA skeleton (left)
EM	beta4_2	0.226 [0.0507, 0.386]*	Mean MD in cingulum cingulate gyrus on FA skeleton (left)
EM	diff_beta4_1_0	0.269 [0.0357, 0.495]*	Mean MD in cingulum cingulate gyrus on FA skeleton (left)
EM	diff_beta4_2_0	0.317 [0.0668, 0.541]*	Mean MD in cingulum cingulate gyrus on FA skeleton (left)
EM	diff_ind3_1_0	-0.0348 [-0.0886, -4.392e-03]*	Mean MD in cingulum cingulate gyrus on FA skeleton (left)
ISP	beta1_0	0.213 [0.0157, 0.61]*	Mean MD in cingulum cingulate gyrus on FA skeleton (left)
ISP	c_prime_0	-0.184 [-0.317, -5.704e-03]*	Mean MD in cingulum cingulate gyrus on FA skeleton (left)
ISP	total_0	-0.169 [-0.307, -7.943e-03]*	Mean MD in cingulum cingulate gyrus on FA skeleton (left)
ISP	beta1_1	0.179 [0.0191, 0.388]*	Mean MD in cingulum cingulate gyrus on FA skeleton (left)
ISP	beta4_1	0.178 [0.0209, 0.336]*	Mean MD in cingulum cingulate gyrus on FA skeleton (left)
ISP	c_prime_1	-0.396 [-0.513, -0.246]*	Mean MD in cingulum cingulate gyrus on FA skeleton (left)
ISP	total_1	-0.399 [-0.514, -0.25]*	Mean MD in cingulum cingulate gyrus on FA skeleton (left)
ISP	beta1_2	0.206 [0.0214, 0.399]*	Mean MD in cingulum cingulate gyrus on FA skeleton (left)
ISP	beta2_2	0.182 [0.0382, 0.326]*	Mean MD in cingulum cingulate gyrus on FA skeleton (left)
ISP	beta4_2	0.226 [0.0507, 0.386]*	Mean MD in cingulum cingulate gyrus on FA skeleton (left)
ISP	c_prime_2	-0.288 [-0.432, -0.123]*	Mean MD in cingulum cingulate gyrus on FA skeleton (left)
ISP	total_2	-0.267 [-0.389, -0.118]*	Mean MD in cingulum cingulate gyrus on FA skeleton (left)
ISP	diff_beta4_1_0	0.269 [0.0357, 0.495]*	Mean MD in cingulum cingulate gyrus on FA skeleton (left)
ISP	diff_beta4_2_0	0.317 [0.0668, 0.541]*	Mean MD in cingulum cingulate gyrus on FA skeleton (left)
ISP	diff_c_prime_1_0	-0.212 [-0.422, -0.0179]*	Mean MD in cingulum cingulate gyrus on FA skeleton (left)

ISP	diff_total_1_0	-0.23 [-0.426, -0.0261]*	Mean MD in cingulum cingulate gyrus on FA skeleton (left)
LF	beta1_0	0.213 [0.0157, 0.61]*	Mean MD in cingulum cingulate gyrus on FA skeleton (left)
LF	c_prime_0	-0.227 [-0.369, -0.0669]*	Mean MD in cingulum cingulate gyrus on FA skeleton (left)
LF	total_0	-0.233 [-0.359, -0.0909]*	Mean MD in cingulum cingulate gyrus on FA skeleton (left)
LF	beta1_1	0.179 [0.0191, 0.388]*	Mean MD in cingulum cingulate gyrus on FA skeleton (left)
LF	beta4_1	0.178 [0.0209, 0.336]*	Mean MD in cingulum cingulate gyrus on FA skeleton (left)
LF	c_prime_1	-0.21 [-0.352, -0.0834]*	Mean MD in cingulum cingulate gyrus on FA skeleton (left)
LF	total_1	-0.23 [-0.369, -0.1]*	Mean MD in cingulum cingulate gyrus on FA skeleton (left)
LF	beta1_2	0.206 [0.0214, 0.399]*	Mean MD in cingulum cingulate gyrus on FA skeleton (left)
LF	beta2_2	0.182 [0.0382, 0.326]*	Mean MD in cingulum cingulate gyrus on FA skeleton (left)
LF	beta4_2	0.226 [0.0507, 0.386]*	Mean MD in cingulum cingulate gyrus on FA skeleton (left)
LF	c_prime_2	-0.28 [-0.426, -0.102]*	Mean MD in cingulum cingulate gyrus on FA skeleton (left)
LF	ind1_2	-4.619e-03 [-0.0237, -2.601e-04]*	Mean MD in cingulum cingulate gyrus on FA skeleton (left)
LF	total_2	-0.294 [-0.436, -0.133]*	Mean MD in cingulum cingulate gyrus on FA skeleton (left)
LF	diff_beta3_1_0	-0.243 [-0.422, -0.023]*	Mean MD in cingulum cingulate gyrus on FA skeleton (left)
LF	diff_beta3_2_0	-0.2 [-0.387, -0.0102]*	Mean MD in cingulum cingulate gyrus on FA skeleton (left)
LF	diff_beta4_1_0	0.269 [0.0357, 0.495]*	Mean MD in cingulum cingulate gyrus on FA skeleton (left)
LF	diff_beta4_2_0	0.317 [0.0668, 0.541]*	Mean MD in cingulum cingulate gyrus on FA skeleton (left)
LF	diff_ind1_2_0	-5.413e-03 [-0.0239, -5.212e-04]*	Mean MD in cingulum cingulate gyrus on FA skeleton (left)
EF	beta1_0	0.213 [0.0157, 0.61]*	Mean MD in cingulum cingulate gyrus on FA skeleton (left)
EF	beta1_1	0.179 [0.0191, 0.388]*	Mean MD in cingulum cingulate gyrus on FA skeleton (left)
EF	beta4_1	0.178 [0.0209, 0.336]*	Mean MD in cingulum cingulate gyrus on FA skeleton (left)
EF	c_prime_1	-0.337 [-0.466, -0.181]*	Mean MD in cingulum cingulate gyrus on FA skeleton (left)
EF	total_1	-0.36 [-0.494, -0.207]*	Mean MD in cingulum cingulate gyrus on FA skeleton (left)
EF	beta1_2	0.206 [0.0214, 0.399]*	Mean MD in cingulum cingulate gyrus on FA skeleton (left)
EF	beta2_2	0.182 [0.0382, 0.326]*	Mean MD in cingulum cingulate gyrus on FA skeleton (left)
EF	beta4_2	0.226 [0.0507, 0.386]*	Mean MD in cingulum cingulate gyrus on FA skeleton (left)
EF	c_prime_2	-0.253 [-0.415, -0.025]*	Mean MD in cingulum cingulate gyrus on FA skeleton (left)
EF	total_2	-0.252 [-0.395, -0.0436]*	Mean MD in cingulum cingulate gyrus on FA skeleton (left)
EF	diff_beta4_1_0	0.269 [0.0357, 0.495]*	Mean MD in cingulum cingulate gyrus on FA skeleton (left)
EF	diff_beta4_2_0	0.317 [0.0668, 0.541]*	Mean MD in cingulum cingulate gyrus on FA skeleton (left)
EF	diff_c_prime_1_0	-0.276 [-0.583, -0.0524]*	Mean MD in cingulum cingulate gyrus on FA skeleton (left)
EF	diff_total_1_0	-0.31 [-0.606, -0.0808]*	Mean MD in cingulum cingulate gyrus on FA skeleton (left)
VS	beta1_0	0.213 [0.0157, 0.61]*	Mean MD in cingulum cingulate gyrus on FA skeleton (left)
VS	c_prime_0	-0.253 [-0.437, -0.0708]*	Mean MD in cingulum cingulate gyrus on FA skeleton (left)
VS	total_0	-0.223 [-0.396, -0.0501]*	Mean MD in cingulum cingulate gyrus on FA skeleton (left)
VS	beta1_1	0.179 [0.0191, 0.388]*	Mean MD in cingulum cingulate gyrus on FA skeleton (left)
VS	beta4_1	0.178 [0.0209, 0.336]*	Mean MD in cingulum cingulate gyrus on FA skeleton (left)
VS	c_prime_1	-0.195 [-0.379, -3.148e-03]*	Mean MD in cingulum cingulate gyrus on FA skeleton (left)
VS	beta1_2	0.206 [0.0214, 0.399]*	Mean MD in cingulum cingulate gyrus on FA skeleton (left)
VS	beta2_2	0.182 [0.0382, 0.326]*	Mean MD in cingulum cingulate gyrus on FA skeleton (left)
VS	beta4_2	0.226 [0.0507, 0.386]*	Mean MD in cingulum cingulate gyrus on FA skeleton (left)
VS	c_prime_2	-0.283 [-0.465, -0.0884]*	Mean MD in cingulum cingulate gyrus on FA skeleton (left)
VS	total_2	-0.292 [-0.475, -0.112]*	Mean MD in cingulum cingulate gyrus on FA skeleton (left)
VS	diff_beta4_1_0	0.269 [0.0357, 0.495]*	Mean MD in cingulum cingulate gyrus on FA skeleton (left)
VS	diff_beta4_2_0	0.317 [0.0668, 0.541]*	Mean MD in cingulum cingulate gyrus on FA skeleton (left)
MMSE	beta1_0	0.213 [0.0157, 0.61]*	Mean MD in cingulum hippocampus on FA skeleton (left)
MMSE	beta3_0	-0.39 [-0.552, -0.239]*	Mean MD in cingulum hippocampus on FA skeleton (left)
MMSE	c_prime_0	-0.297 [-0.476, -0.123]*	Mean MD in cingulum hippocampus on FA skeleton (left)
MMSE	total_0	-0.287 [-0.498, -0.1]*	Mean MD in cingulum hippocampus on FA skeleton (left)
MMSE	beta1_1	0.179 [0.0191, 0.388]*	Mean MD in cingulum hippocampus on FA skeleton (left)
MMSE	beta3_1	-0.47 [-0.787, -0.273]*	Mean MD in cingulum hippocampus on FA skeleton (left)
MMSE	c_prime_1	-0.398 [-0.558, -0.189]*	Mean MD in cingulum hippocampus on FA skeleton (left)
MMSE	ind3_1	-0.123 [-0.295, -0.0314]*	Mean MD in cingulum hippocampus on FA skeleton (left)
MMSE	ind_1	-0.136 [-0.315, -0.0455]*	Mean MD in cingulum hippocampus on FA skeleton (left)
MMSE	total_1	-0.533 [-0.705, -0.268]*	Mean MD in cingulum hippocampus on FA skeleton (left)
MMSE	beta1_2	0.206 [0.0214, 0.399]*	Mean MD in cingulum hippocampus on FA skeleton (left)
MMSE	beta4_2	0.24 [0.0531, 0.459]*	Mean MD in cingulum hippocampus on FA skeleton (left)
MMSE	beta3_2	-0.286 [-0.507, -0.128]*	Mean MD in cingulum hippocampus on FA skeleton (left)
MMSE	ind3_2	-0.0668 [-0.16, -0.0165]*	Mean MD in cingulum hippocampus on FA skeleton (left)
MMSE	ind_2	-0.0839 [-0.204, -0.0241]*	Mean MD in cingulum hippocampus on FA skeleton (left)
MMSE	total_2	-0.262 [-0.469, -0.0361]*	Mean MD in cingulum hippocampus on FA skeleton (left)
MMSE	diff_ind_1_0	-0.146 [-0.335, -0.0237]*	Mean MD in cingulum hippocampus on FA skeleton (left)
MoCA	beta1_0	0.213 [0.0157, 0.61]*	Mean MD in cingulum hippocampus on FA skeleton (left)
MoCA	beta3_0	-0.394 [-0.514, -0.268]*	Mean MD in cingulum hippocampus on FA skeleton (left)
MoCA	c_prime_0	-0.213 [-0.349, -0.0684]*	Mean MD in cingulum hippocampus on FA skeleton (left)
MoCA	total_0	-0.22 [-0.363, -0.0657]*	Mean MD in cingulum hippocampus on FA skeleton (left)
MoCA	beta1_1	0.179 [0.0191, 0.388]*	Mean MD in cingulum hippocampus on FA skeleton (left)
MoCA	beta3_1	-0.406 [-0.675, -0.233]*	Mean MD in cingulum hippocampus on FA skeleton (left)
MoCA	c_prime_1	-0.359 [-0.502, -0.158]*	Mean MD in cingulum hippocampus on FA skeleton (left)
MoCA	ind3_1	-0.106 [-0.254, -0.0269]*	Mean MD in cingulum hippocampus on FA skeleton (left)
MoCA	ind_1	-0.113 [-0.263, -0.0321]*	Mean MD in cingulum hippocampus on FA skeleton (left)
MoCA	total_1	-0.472 [-0.612, -0.227]*	Mean MD in cingulum hippocampus on FA skeleton (left)
MoCA	beta1_2	0.206 [0.0214, 0.399]*	Mean MD in cingulum hippocampus on FA skeleton (left)
MoCA	beta4_2	0.24 [0.0531, 0.459]*	Mean MD in cingulum hippocampus on FA skeleton (left)
MoCA	beta3_2	-0.192 [-0.385, -0.0192]*	Mean MD in cingulum hippocampus on FA skeleton (left)
MoCA	c_prime_2	-0.297 [-0.445, -0.129]*	Mean MD in cingulum hippocampus on FA skeleton (left)
MoCA	ind3_2	-0.05 [-0.158, -3.564e-03]*	Mean MD in cingulum hippocampus on FA skeleton (left)
MoCA	ind_2	-0.0635 [-0.174, -3.671e-03]*	Mean MD in cingulum hippocampus on FA skeleton (left)
MoCA	total_2	-0.361 [-0.526, -0.184]*	Mean MD in cingulum hippocampus on FA skeleton (left)
MoCA	diff_ind_1_0	-0.107 [-0.275, -9.214e-03]*	Mean MD in cingulum hippocampus on FA skeleton (left)
EM	beta1_0	0.213 [0.0157, 0.61]*	Mean MD in cingulum hippocampus on FA skeleton (left)
EM	c_prime_0	-0.229 [-0.351, -0.131]*	Mean MD in cingulum hippocampus on FA skeleton (left)
EM	total_0	-0.25 [-0.352, -0.146]*	Mean MD in cingulum hippocampus on FA skeleton (left)
EM	beta1_1	0.179 [0.0191, 0.388]*	Mean MD in cingulum hippocampus on FA skeleton (left)
EM	c_prime_1	-0.166 [-0.304, -0.0244]*	Mean MD in cingulum hippocampus on FA skeleton (left)
EM	total_1	-0.204 [-0.332, -0.0676]*	Mean MD in cingulum hippocampus on FA skeleton (left)
EM	beta1_2	0.206 [0.0214, 0.399]*	Mean MD in cingulum hippocampus on FA skeleton (left)
EM	beta4_2	0.24 [0.0531, 0.459]*	Mean MD in cingulum hippocampus on FA skeleton (left)
ISP	beta1_0	0.213 [0.0157, 0.61]*	Mean MD in cingulum hippocampus on FA skeleton (left)
ISP	beta3_0	-0.236 [-0.368, -0.105]*	Mean MD in cingulum hippocampus on FA skeleton (left)
ISP	c_prime_0	-0.167 [-0.29, -1.743e-03]*	Mean MD in cingulum hippocampus on FA skeleton (left)
ISP	total_0	-0.169 [-0.307, -7.943e-03]*	Mean MD in cingulum hippocampus on FA skeleton (left)
ISP	beta1_1	0.179 [0.0191, 0.388]*	Mean MD in cingulum hippocampus on FA skeleton (left)
ISP	c_prime_1	-0.379 [-0.497, -0.238]*	Mean MD in cingulum hippocampus on FA skeleton (left)
ISP	total_1	-0.399 [-0.514, -0.25]*	Mean MD in cingulum hippocampus on FA skeleton (left)

ISP	beta1_2	0.206 [0.0214, 0.399]*	Mean MD in cingulum hippocampus on FA skeleton (left)
ISP	beta4_2	0.24 [0.0531, 0.459]*	Mean MD in cingulum hippocampus on FA skeleton (left)
ISP	beta3_2	-0.133 [-0.298, -0.011]*	Mean MD in cingulum hippocampus on FA skeleton (left)
ISP	c_prime_2	-0.258 [-0.397, -0.098]*	Mean MD in cingulum hippocampus on FA skeleton (left)
ISP	ind3_2	-0.032 [-0.108, -3.164e-03]*	Mean MD in cingulum hippocampus on FA skeleton (left)
ISP	total_2	-0.267 [-0.389, -0.118]*	Mean MD in cingulum hippocampus on FA skeleton (left)
ISP	diff_c_prime_1_0	-0.212 [-0.413, -0.0194]*	Mean MD in cingulum hippocampus on FA skeleton (left)
ISP	diff_total_1_0	-0.23 [-0.426, -0.0261]*	Mean MD in cingulum hippocampus on FA skeleton (left)
LF	beta1_0	0.213 [0.0157, 0.61]*	Mean MD in cingulum hippocampus on FA skeleton (left)
LF	beta3_0	-0.166 [-0.311, -0.0153]*	Mean MD in cingulum hippocampus on FA skeleton (left)
LF	c_prime_0	-0.225 [-0.359, -0.0762]*	Mean MD in cingulum hippocampus on FA skeleton (left)
LF	total_0	-0.233 [-0.359, -0.0909]*	Mean MD in cingulum hippocampus on FA skeleton (left)
LF	beta1_1	0.179 [0.0191, 0.388]*	Mean MD in cingulum hippocampus on FA skeleton (left)
LF	beta3_1	-0.235 [-0.392, -0.0343]*	Mean MD in cingulum hippocampus on FA skeleton (left)
LF	c_prime_1	-0.18 [-0.322, -0.0319]*	Mean MD in cingulum hippocampus on FA skeleton (left)
LF	ind3_1	-0.0617 [-0.157, -3.286e-03]*	Mean MD in cingulum hippocampus on FA skeleton (left)
LF	total_1	-0.23 [-0.369, -0.1]*	Mean MD in cingulum hippocampus on FA skeleton (left)
LF	beta1_2	0.206 [0.0214, 0.399]*	Mean MD in cingulum hippocampus on FA skeleton (left)
LF	beta4_2	0.24 [0.0531, 0.459]*	Mean MD in cingulum hippocampus on FA skeleton (left)
LF	beta3_2	-0.197 [-0.348, -0.0209]*	Mean MD in cingulum hippocampus on FA skeleton (left)
LF	c_prime_2	-0.263 [-0.395, -0.089]*	Mean MD in cingulum hippocampus on FA skeleton (left)
LF	ind3_2	-0.0465 [-0.125, -5.791e-03]*	Mean MD in cingulum hippocampus on FA skeleton (left)
LF	total_2	-0.294 [-0.436, -0.133]*	Mean MD in cingulum hippocampus on FA skeleton (left)
EF	beta1_0	0.213 [0.0157, 0.61]*	Mean MD in cingulum hippocampus on FA skeleton (left)
EF	beta1_1	0.179 [0.0191, 0.388]*	Mean MD in cingulum hippocampus on FA skeleton (left)
EF	c_prime_1	-0.317 [-0.454, -0.164]*	Mean MD in cingulum hippocampus on FA skeleton (left)
EF	total_1	-0.36 [-0.494, -0.207]*	Mean MD in cingulum hippocampus on FA skeleton (left)
EF	beta1_2	0.206 [0.0214, 0.399]*	Mean MD in cingulum hippocampus on FA skeleton (left)
EF	beta4_2	0.24 [0.0531, 0.459]*	Mean MD in cingulum hippocampus on FA skeleton (left)
EF	beta3_2	-0.143 [-0.324, -1.794e-03]*	Mean MD in cingulum hippocampus on FA skeleton (left)
EF	ind3_2	-0.0326 [-0.108, -3.471e-03]*	Mean MD in cingulum hippocampus on FA skeleton (left)
EF	total_2	-0.252 [-0.395, -0.0436]*	Mean MD in cingulum hippocampus on FA skeleton (left)
EF	diff_c_prime_1_0	-0.266 [-0.575, -0.042]*	Mean MD in cingulum hippocampus on FA skeleton (left)
EF	diff_total_1_0	-0.31 [-0.606, -0.0808]*	Mean MD in cingulum hippocampus on FA skeleton (left)
VS	beta1_0	0.213 [0.0157, 0.61]*	Mean MD in cingulum hippocampus on FA skeleton (left)
VS	beta3_0	-0.234 [-0.381, -0.0968]*	Mean MD in cingulum hippocampus on FA skeleton (left)
VS	c_prime_0	-0.233 [-0.411, -0.0587]*	Mean MD in cingulum hippocampus on FA skeleton (left)
VS	total_0	-0.223 [-0.396, -0.0501]*	Mean MD in cingulum hippocampus on FA skeleton (left)
VS	beta1_1	0.179 [0.0191, 0.388]*	Mean MD in cingulum hippocampus on FA skeleton (left)
VS	beta3_1	-0.224 [-0.411, -0.0734]*	Mean MD in cingulum hippocampus on FA skeleton (left)
VS	ind3_1	-0.058 [-0.156, -8.288e-03]*	Mean MD in cingulum hippocampus on FA skeleton (left)
VS	beta1_2	0.206 [0.0214, 0.399]*	Mean MD in cingulum hippocampus on FA skeleton (left)
VS	beta4_2	0.24 [0.0531, 0.459]*	Mean MD in cingulum hippocampus on FA skeleton (left)
VS	beta3_2	-0.223 [-0.401, -0.0556]*	Mean MD in cingulum hippocampus on FA skeleton (left)
VS	c_prime_2	-0.238 [-0.423, -0.0723]*	Mean MD in cingulum hippocampus on FA skeleton (left)
VS	ind3_2	-0.0562 [-0.157, -6.722e-03]*	Mean MD in cingulum hippocampus on FA skeleton (left)
VS	total_2	-0.292 [-0.475, -0.112]*	Mean MD in cingulum hippocampus on FA skeleton (left)
MMSE	beta1_0	0.213 [0.0157, 0.61]*	Mean MD in superior longitudinal fasciculus on FA skeleton (right)
MMSE	c_prime_0	-0.322 [-0.516, -0.13]*	Mean MD in superior longitudinal fasciculus on FA skeleton (right)
MMSE	total_0	-0.287 [-0.498, -0.1]*	Mean MD in superior longitudinal fasciculus on FA skeleton (right)
MMSE	beta1_1	0.179 [0.0191, 0.388]*	Mean MD in superior longitudinal fasciculus on FA skeleton (right)
MMSE	c_prime_1	-0.502 [-0.675, -0.236]*	Mean MD in superior longitudinal fasciculus on FA skeleton (right)
MMSE	ind_1	-0.0311 [-0.104, -2.083e-04]*	Mean MD in superior longitudinal fasciculus on FA skeleton (right)
MMSE	total_1	-0.533 [-0.705, -0.268]*	Mean MD in superior longitudinal fasciculus on FA skeleton (right)
MMSE	beta1_2	0.206 [0.0214, 0.399]*	Mean MD in superior longitudinal fasciculus on FA skeleton (right)
MMSE	beta2_2	0.334 [0.16, 0.566]*	Mean MD in superior longitudinal fasciculus on FA skeleton (right)
MMSE	beta4_2	0.194 [0.0187, 0.335]*	Mean MD in superior longitudinal fasciculus on FA skeleton (right)
MMSE	beta3_2	-0.203 [-0.422, -0.053]*	Mean MD in superior longitudinal fasciculus on FA skeleton (right)
MMSE	ind1_2	-0.0134 [-0.0659, -3.319e-03]*	Mean MD in superior longitudinal fasciculus on FA skeleton (right)
MMSE	ind3_2	-0.0373 [-0.109, -7.679e-03]*	Mean MD in superior longitudinal fasciculus on FA skeleton (right)
MMSE	ind_2	-0.0544 [-0.159, -7.820e-03]*	Mean MD in superior longitudinal fasciculus on FA skeleton (right)
MMSE	total_2	-0.262 [-0.469, -0.0361]*	Mean MD in superior longitudinal fasciculus on FA skeleton (right)
MMSE	diff_beta2_2_0	0.304 [0.123, 0.61]*	Mean MD in superior longitudinal fasciculus on FA skeleton (right)
MMSE	diff_ind1_2_0	-0.0131 [-0.065, -2.929e-03]*	Mean MD in superior longitudinal fasciculus on FA skeleton (right)
MMSE	diff_ind1_2_1	-0.011 [-0.0622, -7.156e-04]*	Mean MD in superior longitudinal fasciculus on FA skeleton (right)
MMSE	diff_ind3_2_0	-0.0412 [-0.114, -4.293e-03]*	Mean MD in superior longitudinal fasciculus on FA skeleton (right)
MMSE	diff_ind_1_0	-0.0665 [-0.178, -3.124e-04]*	Mean MD in superior longitudinal fasciculus on FA skeleton (right)
MMSE	diff_ind_2_0	-0.0899 [-0.233, -0.0163]*	Mean MD in superior longitudinal fasciculus on FA skeleton (right)
MoCA	beta1_0	0.213 [0.0157, 0.61]*	Mean MD in superior longitudinal fasciculus on FA skeleton (right)
MoCA	c_prime_0	-0.241 [-0.38, -0.0735]*	Mean MD in superior longitudinal fasciculus on FA skeleton (right)
MoCA	total_0	-0.22 [-0.363, -0.0657]*	Mean MD in superior longitudinal fasciculus on FA skeleton (right)
MoCA	beta1_1	0.179 [0.0191, 0.388]*	Mean MD in superior longitudinal fasciculus on FA skeleton (right)
MoCA	beta3_1	-0.135 [-0.251, -3.337e-03]*	Mean MD in superior longitudinal fasciculus on FA skeleton (right)
MoCA	c_prime_1	-0.442 [-0.585, -0.198]*	Mean MD in superior longitudinal fasciculus on FA skeleton (right)
MoCA	total_1	-0.472 [-0.612, -0.227]*	Mean MD in superior longitudinal fasciculus on FA skeleton (right)
MoCA	beta1_2	0.206 [0.0214, 0.399]*	Mean MD in superior longitudinal fasciculus on FA skeleton (right)
MoCA	beta2_2	0.334 [0.16, 0.566]*	Mean MD in superior longitudinal fasciculus on FA skeleton (right)
MoCA	beta4_2	0.194 [0.0187, 0.335]*	Mean MD in superior longitudinal fasciculus on FA skeleton (right)
MoCA	c_prime_2	-0.329 [-0.48, -0.134]*	Mean MD in superior longitudinal fasciculus on FA skeleton (right)
MoCA	ind1_2	-6.449e-03 [-0.0405, -5.110e-04]*	Mean MD in superior longitudinal fasciculus on FA skeleton (right)
MoCA	total_2	-0.361 [-0.526, -0.184]*	Mean MD in superior longitudinal fasciculus on FA skeleton (right)
MoCA	diff_beta2_2_0	0.304 [0.123, 0.61]*	Mean MD in superior longitudinal fasciculus on FA skeleton (right)
EM	beta1_0	0.213 [0.0157, 0.61]*	Mean MD in superior longitudinal fasciculus on FA skeleton (right)
EM	c_prime_0	-0.243 [-0.363, -0.144]*	Mean MD in superior longitudinal fasciculus on FA skeleton (right)
EM	total_0	-0.25 [-0.352, -0.146]*	Mean MD in superior longitudinal fasciculus on FA skeleton (right)
EM	beta1_1	0.179 [0.0191, 0.388]*	Mean MD in superior longitudinal fasciculus on FA skeleton (right)
EM	beta3_1	-0.134 [-0.265, -0.0221]*	Mean MD in superior longitudinal fasciculus on FA skeleton (right)
EM	c_prime_1	-0.163 [-0.295, -0.0104]*	Mean MD in superior longitudinal fasciculus on FA skeleton (right)
EM	ind3_1	-0.0206 [-0.0639, -8.788e-04]*	Mean MD in superior longitudinal fasciculus on FA skeleton (right)
EM	ind_1	-0.0401 [-0.124, -3.531e-03]*	Mean MD in superior longitudinal fasciculus on FA skeleton (right)
EM	total_1	-0.204 [-0.332, -0.0676]*	Mean MD in superior longitudinal fasciculus on FA skeleton (right)
EM	beta1_2	0.206 [0.0214, 0.399]*	Mean MD in superior longitudinal fasciculus on FA skeleton (right)
EM	beta2_2	0.334 [0.16, 0.566]*	Mean MD in superior longitudinal fasciculus on FA skeleton (right)
EM	beta4_2	0.194 [0.0187, 0.335]*	Mean MD in superior longitudinal fasciculus on FA skeleton (right)
EM	diff_beta2_2_0	0.304 [0.123, 0.61]*	Mean MD in superior longitudinal fasciculus on FA skeleton (right)
ISP	beta1_0	0.213 [0.0157, 0.61]*	Mean MD in superior longitudinal fasciculus on FA skeleton (right)

ISP	c_prime_0	-0.183 [-0.309, -4.977e-04]*	Mean MD in superior longitudinal fasciculus on FA skeleton (right)
ISP	total_0	-0.169 [-0.307, -7.943e-03]*	Mean MD in superior longitudinal fasciculus on FA skeleton (right)
ISP	beta1_1	0.179 [0.0191, 0.388]*	Mean MD in superior longitudinal fasciculus on FA skeleton (right)
ISP	c_prime_1	-0.402 [-0.519, -0.256]*	Mean MD in superior longitudinal fasciculus on FA skeleton (right)
ISP	total_1	-0.399 [-0.514, -0.25]*	Mean MD in superior longitudinal fasciculus on FA skeleton (right)
ISP	beta1_2	0.206 [0.0214, 0.399]*	Mean MD in superior longitudinal fasciculus on FA skeleton (right)
ISP	beta2_2	0.334 [0.16, 0.566]*	Mean MD in superior longitudinal fasciculus on FA skeleton (right)
ISP	beta4_2	0.194 [0.0187, 0.335]*	Mean MD in superior longitudinal fasciculus on FA skeleton (right)
ISP	c_prime_2	-0.293 [-0.429, -0.124]*	Mean MD in superior longitudinal fasciculus on FA skeleton (right)
ISP	total_2	-0.267 [-0.389, -0.118]*	Mean MD in superior longitudinal fasciculus on FA skeleton (right)
ISP	diff_beta2_2_0	0.304 [0.123, 0.61]*	Mean MD in superior longitudinal fasciculus on FA skeleton (right)
ISP	diff_c_prime_1_0	-0.22 [-0.433, -0.0313]*	Mean MD in superior longitudinal fasciculus on FA skeleton (right)
ISP	diff_total_1_0	-0.23 [-0.426, -0.0261]*	Mean MD in superior longitudinal fasciculus on FA skeleton (right)
LF	beta1_0	0.213 [0.0157, 0.61]*	Mean MD in superior longitudinal fasciculus on FA skeleton (right)
LF	c_prime_0	-0.236 [-0.367, -0.0707]*	Mean MD in superior longitudinal fasciculus on FA skeleton (right)
LF	total_0	-0.233 [-0.359, -0.0909]*	Mean MD in superior longitudinal fasciculus on FA skeleton (right)
LF	beta1_1	0.179 [0.0191, 0.388]*	Mean MD in superior longitudinal fasciculus on FA skeleton (right)
LF	c_prime_1	-0.229 [-0.372, -0.0984]*	Mean MD in superior longitudinal fasciculus on FA skeleton (right)
LF	total_1	-0.23 [-0.369, -0.1]*	Mean MD in superior longitudinal fasciculus on FA skeleton (right)
LF	beta1_2	0.206 [0.0214, 0.399]*	Mean MD in superior longitudinal fasciculus on FA skeleton (right)
LF	beta2_2	0.334 [0.16, 0.566]*	Mean MD in superior longitudinal fasciculus on FA skeleton (right)
LF	beta4_2	0.194 [0.0187, 0.335]*	Mean MD in superior longitudinal fasciculus on FA skeleton (right)
LF	c_prime_2	-0.292 [-0.439, -0.116]*	Mean MD in superior longitudinal fasciculus on FA skeleton (right)
LF	total_2	-0.294 [-0.436, -0.133]*	Mean MD in superior longitudinal fasciculus on FA skeleton (right)
LF	diff_beta2_2_0	0.304 [0.123, 0.61]*	Mean MD in superior longitudinal fasciculus on FA skeleton (right)
EF	beta1_0	0.213 [0.0157, 0.61]*	Mean MD in superior longitudinal fasciculus on FA skeleton (right)
EF	beta1_1	0.179 [0.0191, 0.388]*	Mean MD in superior longitudinal fasciculus on FA skeleton (right)
EF	c_prime_1	-0.342 [-0.471, -0.191]*	Mean MD in superior longitudinal fasciculus on FA skeleton (right)
EF	total_1	-0.36 [-0.494, -0.207]*	Mean MD in superior longitudinal fasciculus on FA skeleton (right)
EF	beta1_2	0.206 [0.0214, 0.399]*	Mean MD in superior longitudinal fasciculus on FA skeleton (right)
EF	beta2_2	0.334 [0.16, 0.566]*	Mean MD in superior longitudinal fasciculus on FA skeleton (right)
EF	beta4_2	0.194 [0.0187, 0.335]*	Mean MD in superior longitudinal fasciculus on FA skeleton (right)
EF	c_prime_2	-0.25 [-0.406, -0.0202]*	Mean MD in superior longitudinal fasciculus on FA skeleton (right)
EF	total_2	-0.252 [-0.395, -0.0436]*	Mean MD in superior longitudinal fasciculus on FA skeleton (right)
EF	diff_beta2_2_0	0.304 [0.123, 0.61]*	Mean MD in superior longitudinal fasciculus on FA skeleton (right)
EF	diff_c_prime_1_0	-0.28 [-0.596, -0.0641]*	Mean MD in superior longitudinal fasciculus on FA skeleton (right)
EF	diff_total_1_0	-0.31 [-0.606, -0.0808]*	Mean MD in superior longitudinal fasciculus on FA skeleton (right)
VS	beta1_0	0.213 [0.0157, 0.61]*	Mean MD in superior longitudinal fasciculus on FA skeleton (right)
VS	c_prime_0	-0.249 [-0.425, -0.0626]*	Mean MD in superior longitudinal fasciculus on FA skeleton (right)
VS	total_0	-0.223 [-0.396, -0.0501]*	Mean MD in superior longitudinal fasciculus on FA skeleton (right)
VS	beta1_1	0.179 [0.0191, 0.388]*	Mean MD in superior longitudinal fasciculus on FA skeleton (right)
VS	c_prime_1	-0.189 [-0.371, -3.349e-03]*	Mean MD in superior longitudinal fasciculus on FA skeleton (right)
VS	beta1_2	0.206 [0.0214, 0.399]*	Mean MD in superior longitudinal fasciculus on FA skeleton (right)
VS	beta2_2	0.334 [0.16, 0.566]*	Mean MD in superior longitudinal fasciculus on FA skeleton (right)
VS	beta4_2	0.194 [0.0187, 0.335]*	Mean MD in superior longitudinal fasciculus on FA skeleton (right)
VS	c_prime_2	-0.288 [-0.467, -0.0929]*	Mean MD in superior longitudinal fasciculus on FA skeleton (right)
VS	total_2	-0.292 [-0.475, -0.112]*	Mean MD in superior longitudinal fasciculus on FA skeleton (right)
VS	diff_beta2_2_0	0.304 [0.123, 0.61]*	Mean MD in superior longitudinal fasciculus on FA skeleton (right)
MMSE	beta1_0	0.213 [0.0157, 0.61]*	Mean MD in superior longitudinal fasciculus on FA skeleton (left)
MMSE	c_prime_0	-0.322 [-0.522, -0.132]*	Mean MD in superior longitudinal fasciculus on FA skeleton (left)
MMSE	total_0	-0.287 [-0.498, -0.1]*	Mean MD in superior longitudinal fasciculus on FA skeleton (left)
MMSE	beta1_1	0.179 [0.0191, 0.388]*	Mean MD in superior longitudinal fasciculus on FA skeleton (left)
MMSE	beta4_1	0.186 [0.0183, 0.352]*	Mean MD in superior longitudinal fasciculus on FA skeleton (left)
MMSE	beta3_1	-0.184 [-0.355, -0.0984]*	Mean MD in superior longitudinal fasciculus on FA skeleton (left)
MMSE	c_prime_1	-0.485 [-0.654, -0.216]*	Mean MD in superior longitudinal fasciculus on FA skeleton (left)
MMSE	ind3_1	-0.0355 [-0.0915, -5.630e-03]*	Mean MD in superior longitudinal fasciculus on FA skeleton (left)
MMSE	ind_1	-0.0487 [-0.14, -7.639e-03]*	Mean MD in superior longitudinal fasciculus on FA skeleton (left)
MMSE	total_1	-0.533 [-0.705, -0.268]*	Mean MD in superior longitudinal fasciculus on FA skeleton (left)
MMSE	beta1_2	0.206 [0.0214, 0.399]*	Mean MD in superior longitudinal fasciculus on FA skeleton (left)
MMSE	beta3_2	-0.208 [-0.423, -0.0711]*	Mean MD in superior longitudinal fasciculus on FA skeleton (left)
MMSE	ind1_2	-0.0101 [-0.0566, -5.423e-04]*	Mean MD in superior longitudinal fasciculus on FA skeleton (left)
MMSE	ind_2	-0.0356 [-0.135, -1.592e-03]*	Mean MD in superior longitudinal fasciculus on FA skeleton (left)
MMSE	total_2	-0.262 [-0.469, -0.0361]*	Mean MD in superior longitudinal fasciculus on FA skeleton (left)
MMSE	diff_beta4_1_0	0.228 [0.0312, 0.464]*	Mean MD in superior longitudinal fasciculus on FA skeleton (left)
MMSE	diff_ind3_1_0	-0.0389 [-0.0969, -6.528e-03]*	Mean MD in superior longitudinal fasciculus on FA skeleton (left)
MMSE	diff_ind_1_0	-0.0837 [-0.203, -0.0134]*	Mean MD in superior longitudinal fasciculus on FA skeleton (left)
MMSE	diff_ind_2_0	-0.0706 [-0.217, -7.459e-03]*	Mean MD in superior longitudinal fasciculus on FA skeleton (left)
MoCA	beta1_0	0.213 [0.0157, 0.61]*	Mean MD in superior longitudinal fasciculus on FA skeleton (left)
MoCA	c_prime_0	-0.242 [-0.38, -0.0767]*	Mean MD in superior longitudinal fasciculus on FA skeleton (left)
MoCA	total_0	-0.22 [-0.363, -0.0657]*	Mean MD in superior longitudinal fasciculus on FA skeleton (left)
MoCA	beta1_1	0.179 [0.0191, 0.388]*	Mean MD in superior longitudinal fasciculus on FA skeleton (left)
MoCA	beta4_1	0.186 [0.0183, 0.352]*	Mean MD in superior longitudinal fasciculus on FA skeleton (left)
MoCA	beta3_1	-0.19 [-0.341, -0.0817]*	Mean MD in superior longitudinal fasciculus on FA skeleton (left)
MoCA	c_prime_1	-0.428 [-0.57, -0.189]*	Mean MD in superior longitudinal fasciculus on FA skeleton (left)
MoCA	ind3_1	-0.0364 [-0.0918, -4.538e-03]*	Mean MD in superior longitudinal fasciculus on FA skeleton (left)
MoCA	ind_1	-0.044 [-0.123, -2.144e-03]*	Mean MD in superior longitudinal fasciculus on FA skeleton (left)
MoCA	total_1	-0.472 [-0.612, -0.227]*	Mean MD in superior longitudinal fasciculus on FA skeleton (left)
MoCA	beta1_2	0.206 [0.0214, 0.399]*	Mean MD in superior longitudinal fasciculus on FA skeleton (left)
MoCA	beta3_2	-0.176 [-0.362, -0.0434]*	Mean MD in superior longitudinal fasciculus on FA skeleton (left)
MoCA	c_prime_2	-0.332 [-0.475, -0.143]*	Mean MD in superior longitudinal fasciculus on FA skeleton (left)
MoCA	ind1_2	-8.684e-03 [-0.0428, -9.446e-05]*	Mean MD in superior longitudinal fasciculus on FA skeleton (left)
MoCA	total_2	-0.361 [-0.526, -0.184]*	Mean MD in superior longitudinal fasciculus on FA skeleton (left)
MoCA	diff_beta4_1_0	0.228 [0.0312, 0.464]*	Mean MD in superior longitudinal fasciculus on FA skeleton (left)
MoCA	diff_ind3_1_0	-0.0433 [-0.0944, -5.144e-03]*	Mean MD in superior longitudinal fasciculus on FA skeleton (left)
MoCA	diff_ind_1_0	-0.0666 [-0.151, -7.577e-03]*	Mean MD in superior longitudinal fasciculus on FA skeleton (left)
MoCA	diff_ind_2_0	-0.0513 [-0.15, -3.918e-03]*	Mean MD in superior longitudinal fasciculus on FA skeleton (left)
EM	beta1_0	0.213 [0.0157, 0.61]*	Mean MD in superior longitudinal fasciculus on FA skeleton (left)
EM	c_prime_0	-0.242 [-0.366, -0.146]*	Mean MD in superior longitudinal fasciculus on FA skeleton (left)
EM	total_0	-0.25 [-0.352, -0.146]*	Mean MD in superior longitudinal fasciculus on FA skeleton (left)
EM	beta1_1	0.179 [0.0191, 0.388]*	Mean MD in superior longitudinal fasciculus on FA skeleton (left)
EM	beta4_1	0.186 [0.0183, 0.352]*	Mean MD in superior longitudinal fasciculus on FA skeleton (left)
EM	beta3_1	-0.25 [-0.381, -0.14]*	Mean MD in superior longitudinal fasciculus on FA skeleton (left)
EM	ind3_1	-0.0457 [-0.103, -8.743e-03]*	Mean MD in superior longitudinal fasciculus on FA skeleton (left)
EM	ind_1	-0.0652 [-0.153, -0.0151]*	Mean MD in superior longitudinal fasciculus on FA skeleton (left)
EM	total_1	-0.204 [-0.332, -0.0676]*	Mean MD in superior longitudinal fasciculus on FA skeleton (left)
EM	beta1_2	0.206 [0.0214, 0.399]*	Mean MD in superior longitudinal fasciculus on FA skeleton (left)

EM	ind1_2	-5.356e-03 [-0.0372, -5.080e-04]*	Mean MD in superior longitudinal fasciculus on FA skeleton (left)
EM	ind_2	-0.0332 [-0.099, -1.485e-04]*	Mean MD in superior longitudinal fasciculus on FA skeleton (left)
EM	diff_beta3_1_0	-0.171 [-0.37, -1.272e-03]*	Mean MD in superior longitudinal fasciculus on FA skeleton (left)
EM	diff_beta4_1_0	0.228 [0.0312, 0.464]*	Mean MD in superior longitudinal fasciculus on FA skeleton (left)
EM	diff_ind3_1_0	-0.0476 [-0.11, -8.772e-03]*	Mean MD in superior longitudinal fasciculus on FA skeleton (left)
EM	diff_ind_1_0	-0.0571 [-0.159, -1.257e-03]*	Mean MD in superior longitudinal fasciculus on FA skeleton (left)
ISP	beta1_0	0.213 [0.0157, 0.61]*	Mean MD in superior longitudinal fasciculus on FA skeleton (left)
ISP	c_prime_0	-0.184 [-0.31, -2.226e-03]*	Mean MD in superior longitudinal fasciculus on FA skeleton (left)
ISP	total_0	-0.169 [-0.307, -7.943e-03]*	Mean MD in superior longitudinal fasciculus on FA skeleton (left)
ISP	beta1_1	0.179 [0.0191, 0.388]*	Mean MD in superior longitudinal fasciculus on FA skeleton (left)
ISP	beta4_1	0.186 [0.0183, 0.352]*	Mean MD in superior longitudinal fasciculus on FA skeleton (left)
ISP	beta3_1	-0.122 [-0.256, -0.0131]*	Mean MD in superior longitudinal fasciculus on FA skeleton (left)
ISP	c_prime_1	-0.387 [-0.501, -0.246]*	Mean MD in superior longitudinal fasciculus on FA skeleton (left)
ISP	ind3_1	-0.0242 [-0.0739, -9.200e-04]*	Mean MD in superior longitudinal fasciculus on FA skeleton (left)
ISP	total_1	-0.399 [-0.514, -0.25]*	Mean MD in superior longitudinal fasciculus on FA skeleton (left)
ISP	beta1_2	0.206 [0.0214, 0.399]*	Mean MD in superior longitudinal fasciculus on FA skeleton (left)
ISP	beta3_2	-0.163 [-0.324, -0.0413]*	Mean MD in superior longitudinal fasciculus on FA skeleton (left)
ISP	c_prime_2	-0.273 [-0.401, -0.123]*	Mean MD in superior longitudinal fasciculus on FA skeleton (left)
ISP	ind1_2	-7.054e-03 [-0.0543, -1.340e-03]*	Mean MD in superior longitudinal fasciculus on FA skeleton (left)
ISP	total_2	-0.267 [-0.389, -0.118]*	Mean MD in superior longitudinal fasciculus on FA skeleton (left)
ISP	diff_beta4_1_0	0.228 [0.0312, 0.464]*	Mean MD in superior longitudinal fasciculus on FA skeleton (left)
ISP	diff_ind1_2_0	-7.055e-03 [-0.048, -1.106e-03]*	Mean MD in superior longitudinal fasciculus on FA skeleton (left)
ISP	diff_c_prime_1_0	-0.203 [-0.415, -0.0165]*	Mean MD in superior longitudinal fasciculus on FA skeleton (left)
ISP	diff_total_1_0	-0.23 [-0.426, -0.0261]*	Mean MD in superior longitudinal fasciculus on FA skeleton (left)
LF	beta1_0	0.213 [0.0157, 0.61]*	Mean MD in superior longitudinal fasciculus on FA skeleton (left)
LF	c_prime_0	-0.237 [-0.37, -0.0788]*	Mean MD in superior longitudinal fasciculus on FA skeleton (left)
LF	total_0	-0.233 [-0.359, -0.0909]*	Mean MD in superior longitudinal fasciculus on FA skeleton (left)
LF	beta1_1	0.179 [0.0191, 0.388]*	Mean MD in superior longitudinal fasciculus on FA skeleton (left)
LF	beta4_1	0.186 [0.0183, 0.352]*	Mean MD in superior longitudinal fasciculus on FA skeleton (left)
LF	c_prime_1	-0.217 [-0.36, -0.0793]*	Mean MD in superior longitudinal fasciculus on FA skeleton (left)
LF	ind3_1	-0.0247 [-0.0674, -6.101e-05]*	Mean MD in superior longitudinal fasciculus on FA skeleton (left)
LF	total_1	-0.23 [-0.369, -0.1]*	Mean MD in superior longitudinal fasciculus on FA skeleton (left)
LF	beta1_2	0.206 [0.0214, 0.399]*	Mean MD in superior longitudinal fasciculus on FA skeleton (left)
LF	beta3_2	-0.148 [-0.301, -1.226e-03]*	Mean MD in superior longitudinal fasciculus on FA skeleton (left)
LF	c_prime_2	-0.293 [-0.438, -0.123]*	Mean MD in superior longitudinal fasciculus on FA skeleton (left)
LF	total_2	-0.294 [-0.436, -0.133]*	Mean MD in superior longitudinal fasciculus on FA skeleton (left)
LF	diff_beta4_1_0	0.228 [0.0312, 0.464]*	Mean MD in superior longitudinal fasciculus on FA skeleton (left)
EF	beta1_0	0.213 [0.0157, 0.61]*	Mean MD in superior longitudinal fasciculus on FA skeleton (left)
EF	beta1_1	0.179 [0.0191, 0.388]*	Mean MD in superior longitudinal fasciculus on FA skeleton (left)
EF	beta4_1	0.186 [0.0183, 0.352]*	Mean MD in superior longitudinal fasciculus on FA skeleton (left)
EF	beta3_1	-0.128 [-0.288, -0.0162]*	Mean MD in superior longitudinal fasciculus on FA skeleton (left)
EF	c_prime_1	-0.33 [-0.462, -0.181]*	Mean MD in superior longitudinal fasciculus on FA skeleton (left)
EF	ind3_1	-0.026 [-0.0849, -7.488e-04]*	Mean MD in superior longitudinal fasciculus on FA skeleton (left)
EF	total_1	-0.36 [-0.494, -0.207]*	Mean MD in superior longitudinal fasciculus on FA skeleton (left)
EF	beta1_2	0.206 [0.0214, 0.399]*	Mean MD in superior longitudinal fasciculus on FA skeleton (left)
EF	beta3_2	-0.181 [-0.332, -0.0636]*	Mean MD in superior longitudinal fasciculus on FA skeleton (left)
EF	c_prime_2	-0.234 [-0.383, -0.0196]*	Mean MD in superior longitudinal fasciculus on FA skeleton (left)
EF	ind1_2	-7.579e-03 [-0.0485, -1.513e-03]*	Mean MD in superior longitudinal fasciculus on FA skeleton (left)
EF	total_2	-0.252 [-0.395, -0.0436]*	Mean MD in superior longitudinal fasciculus on FA skeleton (left)
EF	diff_beta4_1_0	0.228 [0.0312, 0.464]*	Mean MD in superior longitudinal fasciculus on FA skeleton (left)
EF	diff_ind1_2_0	-7.316e-03 [-0.0471, -1.137e-03]*	Mean MD in superior longitudinal fasciculus on FA skeleton (left)
EF	diff_ind3_1_0	-0.0291 [-0.0849, -8.186e-04]*	Mean MD in superior longitudinal fasciculus on FA skeleton (left)
EF	diff_c_prime_1_0	-0.268 [-0.582, -0.052]*	Mean MD in superior longitudinal fasciculus on FA skeleton (left)
EF	diff_total_1_0	-0.31 [-0.606, -0.0808]*	Mean MD in superior longitudinal fasciculus on FA skeleton (left)
VS	beta1_0	0.213 [0.0157, 0.61]*	Mean MD in superior longitudinal fasciculus on FA skeleton (left)
VS	c_prime_0	-0.248 [-0.427, -0.0632]*	Mean MD in superior longitudinal fasciculus on FA skeleton (left)
VS	total_0	-0.223 [-0.396, -0.0501]*	Mean MD in superior longitudinal fasciculus on FA skeleton (left)
VS	beta1_1	0.179 [0.0191, 0.388]*	Mean MD in superior longitudinal fasciculus on FA skeleton (left)
VS	beta4_1	0.186 [0.0183, 0.352]*	Mean MD in superior longitudinal fasciculus on FA skeleton (left)
VS	c_prime_1	-0.179 [-0.363, -1.197e-03]*	Mean MD in superior longitudinal fasciculus on FA skeleton (left)
VS	beta1_2	0.206 [0.0214, 0.399]*	Mean MD in superior longitudinal fasciculus on FA skeleton (left)
VS	c_prime_2	-0.29 [-0.467, -0.0928]*	Mean MD in superior longitudinal fasciculus on FA skeleton (left)
VS	total_2	-0.292 [-0.475, -0.112]*	Mean MD in superior longitudinal fasciculus on FA skeleton (left)
VS	diff_beta4_1_0	0.228 [0.0312, 0.464]*	Mean MD in superior longitudinal fasciculus on FA skeleton (left)
MMSE	beta1_0	0.213 [0.0157, 0.61]*	Mean MD in tapetum on FA skeleton (left)
MMSE	beta3_0	-0.395 [-0.556, -0.235]*	Mean MD in tapetum on FA skeleton (left)
MMSE	c_prime_0	-0.341 [-0.515, -0.176]*	Mean MD in tapetum on FA skeleton (left)
MMSE	ind1_0	-0.0103 [-0.0398, -3.871e-03]*	Mean MD in tapetum on FA skeleton (left)
MMSE	total_0	-0.287 [-0.498, -0.1]*	Mean MD in tapetum on FA skeleton (left)
MMSE	beta1_1	0.179 [0.0191, 0.388]*	Mean MD in tapetum on FA skeleton (left)
MMSE	beta4_1	0.156 [0.023, 0.28]*	Mean MD in tapetum on FA skeleton (left)
MMSE	beta3_1	-0.308 [-0.57, -0.071]*	Mean MD in tapetum on FA skeleton (left)
MMSE	c_prime_1	-0.472 [-0.642, -0.207]*	Mean MD in tapetum on FA skeleton (left)
MMSE	ind1_1	4.846e-03 [1.370e-04, 0.038]*	Mean MD in tapetum on FA skeleton (left)
MMSE	ind3_1	-0.0482 [-0.128, -6.906e-03]*	Mean MD in tapetum on FA skeleton (left)
MMSE	ind_1	-0.0614 [-0.153, -7.543e-03]*	Mean MD in tapetum on FA skeleton (left)
MMSE	total_1	-0.533 [-0.705, -0.268]*	Mean MD in tapetum on FA skeleton (left)
MMSE	beta1_2	0.206 [0.0214, 0.399]*	Mean MD in tapetum on FA skeleton (left)
MMSE	beta4_2	0.263 [0.0435, 0.601]*	Mean MD in tapetum on FA skeleton (left)
MMSE	beta3_2	-0.328 [-0.519, -0.176]*	Mean MD in tapetum on FA skeleton (left)
MMSE	ind3_2	-0.078 [-0.177, -0.0199]*	Mean MD in tapetum on FA skeleton (left)
MMSE	ind_2	-0.0951 [-0.225, -0.0308]*	Mean MD in tapetum on FA skeleton (left)
MMSE	total_2	-0.262 [-0.469, -0.0361]*	Mean MD in tapetum on FA skeleton (left)
MMSE	diff_beta2_1_0	-0.274 [-0.475, -0.0181]*	Mean MD in tapetum on FA skeleton (left)
MMSE	diff_beta4_1_0	0.208 [7.417e-03, 0.426]*	Mean MD in tapetum on FA skeleton (left)
MMSE	diff_beta4_2_0	0.316 [0.0436, 0.685]*	Mean MD in tapetum on FA skeleton (left)
MMSE	diff_beta5_1_0	-0.241 [-0.566, -0.0856]*	Mean MD in tapetum on FA skeleton (left)
MMSE	diff_beta5_2_0	-0.247 [-0.585, -0.0567]*	Mean MD in tapetum on FA skeleton (left)
MMSE	diff_ind1_1_0	0.0151 [6.765e-03, 0.055]*	Mean MD in tapetum on FA skeleton (left)
MMSE	diff_ind2_1_0	-0.0599 [-0.189, -3.922e-03]*	Mean MD in tapetum on FA skeleton (left)
MMSE	diff_ind2_2_0	-0.06 [-0.198, -3.686e-03]*	Mean MD in tapetum on FA skeleton (left)
MMSE	diff_ind3_2_0	-0.101 [-0.227, -0.0228]*	Mean MD in tapetum on FA skeleton (left)
MMSE	diff_ind_1_0	-0.116 [-0.247, -0.0177]*	Mean MD in tapetum on FA skeleton (left)
MMSE	diff_ind_2_0	-0.149 [-0.315, -0.0494]*	Mean MD in tapetum on FA skeleton (left)
MMSE	diff_c_prime_2_1	0.305 [0.0115, 0.588]*	Mean MD in tapetum on FA skeleton (left)
MoCA	beta1_0	0.213 [0.0157, 0.61]*	Mean MD in tapetum on FA skeleton (left)

MoCA	beta3_0	-0.366 [-0.483, -0.234]*	Mean MD in tapetum on FA skeleton (left)
MoCA	c_prime_0	-0.255 [-0.377, -0.109]*	Mean MD in tapetum on FA skeleton (left)
MoCA	ind1_0	-9.530e-03 [-0.0353, -2.928e-03]*	Mean MD in tapetum on FA skeleton (left)
MoCA	total_0	-0.22 [-0.363, -0.0657]*	Mean MD in tapetum on FA skeleton (left)
MoCA	beta1_1	0.179 [0.0191, 0.388]*	Mean MD in tapetum on FA skeleton (left)
MoCA	beta4_1	0.156 [0.023, 0.28]*	Mean MD in tapetum on FA skeleton (left)
MoCA	beta3_1	-0.276 [-0.476, -0.0609]*	Mean MD in tapetum on FA skeleton (left)
MoCA	c_prime_1	-0.422 [-0.569, -0.185]*	Mean MD in tapetum on FA skeleton (left)
MoCA	ind3_1	-0.0429 [-0.099, -3.444e-03]*	Mean MD in tapetum on FA skeleton (left)
MoCA	ind_1	-0.0505 [-0.123, -1.749e-04]*	Mean MD in tapetum on FA skeleton (left)
MoCA	total_1	-0.472 [-0.612, -0.227]*	Mean MD in tapetum on FA skeleton (left)
MoCA	beta1_2	0.206 [0.0214, 0.399]*	Mean MD in tapetum on FA skeleton (left)
MoCA	beta4_2	0.263 [0.0435, 0.601]*	Mean MD in tapetum on FA skeleton (left)
MoCA	beta3_2	-0.325 [-0.444, -0.228]*	Mean MD in tapetum on FA skeleton (left)
MoCA	c_prime_2	-0.262 [-0.382, -0.108]*	Mean MD in tapetum on FA skeleton (left)
MoCA	ind3_2	-0.0858 [-0.225, -0.0161]*	Mean MD in tapetum on FA skeleton (left)
MoCA	ind_2	-0.099 [-0.243, -0.0231]*	Mean MD in tapetum on FA skeleton (left)
MoCA	total_2	-0.361 [-0.526, -0.184]*	Mean MD in tapetum on FA skeleton (left)
MoCA	diff_beta2_1_0	-0.274 [-0.475, -0.0181]*	Mean MD in tapetum on FA skeleton (left)
MoCA	diff_beta4_1_0	0.208 [7.417e-03, 0.426]*	Mean MD in tapetum on FA skeleton (left)
MoCA	diff_beta4_2_0	0.316 [0.0436, 0.685]*	Mean MD in tapetum on FA skeleton (left)
MoCA	diff_beta5_1_0	-0.168 [-0.421, -0.0233]*	Mean MD in tapetum on FA skeleton (left)
MoCA	diff_ind1_1_0	0.0137 [5.649e-03, 0.0453]*	Mean MD in tapetum on FA skeleton (left)
MoCA	diff_ind2_1_0	-0.037 [-0.124, -1.596e-03]*	Mean MD in tapetum on FA skeleton (left)
MoCA	diff_ind2_2_0	-0.0403 [-0.133, -2.784e-03]*	Mean MD in tapetum on FA skeleton (left)
MoCA	diff_ind3_2_0	-0.106 [-0.255, -0.017]*	Mean MD in tapetum on FA skeleton (left)
MoCA	diff_ind_1_0	-0.0859 [-0.187, -6.172e-03]*	Mean MD in tapetum on FA skeleton (left)
MoCA	diff_ind_2_0	-0.135 [-0.292, -0.036]*	Mean MD in tapetum on FA skeleton (left)
EM	beta1_0	0.213 [0.0157, 0.61]*	Mean MD in tapetum on FA skeleton (left)
EM	beta3_0	-0.263 [-0.355, -0.0964]*	Mean MD in tapetum on FA skeleton (left)
EM	c_prime_0	-0.254 [-0.366, -0.154]*	Mean MD in tapetum on FA skeleton (left)
EM	ind1_0	-6.843e-03 [-0.0274, -1.978e-03]*	Mean MD in tapetum on FA skeleton (left)
EM	total_0	-0.25 [-0.352, -0.146]*	Mean MD in tapetum on FA skeleton (left)
EM	beta1_1	0.179 [0.0191, 0.388]*	Mean MD in tapetum on FA skeleton (left)
EM	beta4_1	0.156 [0.023, 0.28]*	Mean MD in tapetum on FA skeleton (left)
EM	c_prime_1	-0.178 [-0.327, -0.0344]*	Mean MD in tapetum on FA skeleton (left)
EM	total_1	-0.204 [-0.332, -0.0676]*	Mean MD in tapetum on FA skeleton (left)
EM	beta1_2	0.206 [0.0214, 0.399]*	Mean MD in tapetum on FA skeleton (left)
EM	beta4_2	0.263 [0.0435, 0.601]*	Mean MD in tapetum on FA skeleton (left)
EM	diff_beta2_1_0	-0.274 [-0.475, -0.0181]*	Mean MD in tapetum on FA skeleton (left)
EM	diff_beta4_1_0	0.208 [7.417e-03, 0.426]*	Mean MD in tapetum on FA skeleton (left)
EM	diff_beta4_2_0	0.316 [0.0436, 0.685]*	Mean MD in tapetum on FA skeleton (left)
EM	diff_ind1_1_0	7.480e-03 [6.863e-04, 0.0296]*	Mean MD in tapetum on FA skeleton (left)
EM	diff_ind1_2_0	6.819e-03 [1.089e-03, 0.0281]*	Mean MD in tapetum on FA skeleton (left)
ISP	beta1_0	0.213 [0.0157, 0.61]*	Mean MD in tapetum on FA skeleton (left)
ISP	beta3_0	-0.307 [-0.434, -0.204]*	Mean MD in tapetum on FA skeleton (left)
ISP	c_prime_0	-0.197 [-0.312, -0.0349]*	Mean MD in tapetum on FA skeleton (left)
ISP	ind1_0	-8.206e-03 [-0.0295, -2.080e-03]*	Mean MD in tapetum on FA skeleton (left)
ISP	total_0	-0.169 [-0.307, -7.943e-03]*	Mean MD in tapetum on FA skeleton (left)
ISP	beta1_1	0.179 [0.0191, 0.388]*	Mean MD in tapetum on FA skeleton (left)
ISP	beta4_1	0.156 [0.023, 0.28]*	Mean MD in tapetum on FA skeleton (left)
ISP	c_prime_1	-0.391 [-0.522, -0.251]*	Mean MD in tapetum on FA skeleton (left)
ISP	total_1	-0.399 [-0.514, -0.25]*	Mean MD in tapetum on FA skeleton (left)
ISP	beta1_2	0.206 [0.0214, 0.399]*	Mean MD in tapetum on FA skeleton (left)
ISP	beta4_2	0.263 [0.0435, 0.601]*	Mean MD in tapetum on FA skeleton (left)
ISP	beta3_2	-0.232 [-0.37, -0.103]*	Mean MD in tapetum on FA skeleton (left)
ISP	c_prime_2	-0.235 [-0.37, -0.0851]*	Mean MD in tapetum on FA skeleton (left)
ISP	ind3_2	-0.0547 [-0.138, -0.0162]*	Mean MD in tapetum on FA skeleton (left)
ISP	total_2	-0.267 [-0.389, -0.118]*	Mean MD in tapetum on FA skeleton (left)
ISP	diff_beta2_1_0	-0.274 [-0.475, -0.0181]*	Mean MD in tapetum on FA skeleton (left)
ISP	diff_beta4_1_0	0.208 [7.417e-03, 0.426]*	Mean MD in tapetum on FA skeleton (left)
ISP	diff_beta4_2_0	0.316 [0.0436, 0.685]*	Mean MD in tapetum on FA skeleton (left)
ISP	diff_ind1_1_0	0.0103 [2.554e-03, 0.0372]*	Mean MD in tapetum on FA skeleton (left)
ISP	diff_ind3_2_0	-0.0705 [-0.169, -0.0147]*	Mean MD in tapetum on FA skeleton (left)
ISP	diff_c_prime_1_0	-0.194 [-0.392, -8.248e-03]*	Mean MD in tapetum on FA skeleton (left)
ISP	diff_total_1_0	-0.23 [-0.426, -0.0261]*	Mean MD in tapetum on FA skeleton (left)
LF	beta1_0	0.213 [0.0157, 0.61]*	Mean MD in tapetum on FA skeleton (left)
LF	beta3_0	-0.23 [-0.354, -0.101]*	Mean MD in tapetum on FA skeleton (left)
LF	c_prime_0	-0.245 [-0.382, -0.103]*	Mean MD in tapetum on FA skeleton (left)
LF	ind1_0	-6.038e-03 [-0.0244, -1.700e-03]*	Mean MD in tapetum on FA skeleton (left)
LF	total_0	-0.233 [-0.359, -0.0909]*	Mean MD in tapetum on FA skeleton (left)
LF	beta1_1	0.179 [0.0191, 0.388]*	Mean MD in tapetum on FA skeleton (left)
LF	beta4_1	0.156 [0.023, 0.28]*	Mean MD in tapetum on FA skeleton (left)
LF	c_prime_1	-0.214 [-0.364, -0.0834]*	Mean MD in tapetum on FA skeleton (left)
LF	total_1	-0.23 [-0.369, -0.1]*	Mean MD in tapetum on FA skeleton (left)
LF	beta1_2	0.206 [0.0214, 0.399]*	Mean MD in tapetum on FA skeleton (left)
LF	beta4_2	0.263 [0.0435, 0.601]*	Mean MD in tapetum on FA skeleton (left)
LF	beta3_2	-0.236 [-0.359, -0.121]*	Mean MD in tapetum on FA skeleton (left)
LF	c_prime_2	-0.248 [-0.391, -0.0754]*	Mean MD in tapetum on FA skeleton (left)
LF	ind3_2	-0.061 [-0.144, -6.256e-03]*	Mean MD in tapetum on FA skeleton (left)
LF	total_2	-0.294 [-0.436, -0.133]*	Mean MD in tapetum on FA skeleton (left)
LF	diff_beta2_1_0	-0.274 [-0.475, -0.0181]*	Mean MD in tapetum on FA skeleton (left)
LF	diff_beta4_1_0	0.208 [7.417e-03, 0.426]*	Mean MD in tapetum on FA skeleton (left)
LF	diff_beta4_2_0	0.316 [0.0436, 0.685]*	Mean MD in tapetum on FA skeleton (left)
LF	diff_ind1_1_0	8.898e-03 [2.760e-03, 0.033]*	Mean MD in tapetum on FA skeleton (left)
LF	diff_ind3_2_0	-0.0707 [-0.167, -8.995e-03]*	Mean MD in tapetum on FA skeleton (left)
EF	beta1_0	0.213 [0.0157, 0.61]*	Mean MD in tapetum on FA skeleton (left)
EF	beta1_1	0.179 [0.0191, 0.388]*	Mean MD in tapetum on FA skeleton (left)
EF	beta4_1	0.156 [0.023, 0.28]*	Mean MD in tapetum on FA skeleton (left)
EF	c_prime_1	-0.329 [-0.471, -0.186]*	Mean MD in tapetum on FA skeleton (left)
EF	total_1	-0.36 [-0.494, -0.207]*	Mean MD in tapetum on FA skeleton (left)
EF	beta1_2	0.206 [0.0214, 0.399]*	Mean MD in tapetum on FA skeleton (left)
EF	beta4_2	0.263 [0.0435, 0.601]*	Mean MD in tapetum on FA skeleton (left)
EF	beta3_2	-0.215 [-0.349, -0.0751]*	Mean MD in tapetum on FA skeleton (left)
EF	ind3_2	-0.0491 [-0.123, -0.0164]*	Mean MD in tapetum on FA skeleton (left)
EF	ind_2	-0.0483 [-0.135, -7.824e-03]*	Mean MD in tapetum on FA skeleton (left)

EF	total_2	-0.252 [-0.395, -0.0436]*	Mean MD in tapetum on FA skeleton (left)
EF	diff_beta2_1_0	-0.274 [-0.475, -0.0181]*	Mean MD in tapetum on FA skeleton (left)
EF	diff_beta4_1_0	0.208 [7.417e-03, 0.426]*	Mean MD in tapetum on FA skeleton (left)
EF	diff_beta4_2_0	0.316 [0.0436, 0.685]*	Mean MD in tapetum on FA skeleton (left)
EF	diff_ind3_2_0	-0.0545 [-0.129, -0.0138]*	Mean MD in tapetum on FA skeleton (left)
EF	diff_ind_2_0	-0.0616 [-0.155, -8.683e-03]*	Mean MD in tapetum on FA skeleton (left)
EF	diff_c_prime_1_0	-0.266 [-0.595, -0.0452]*	Mean MD in tapetum on FA skeleton (left)
EF	diff_total_1_0	-0.31 [-0.606, -0.0808]*	Mean MD in tapetum on FA skeleton (left)
VS	beta1_0	0.213 [0.0157, 0.61]*	Mean MD in tapetum on FA skeleton (left)
VS	beta3_0	-0.335 [-0.465, -0.193]*	Mean MD in tapetum on FA skeleton (left)
VS	beta5_0	0.14 [0.0191, 0.292]*	Mean MD in tapetum on FA skeleton (left)
VS	c_prime_0	-0.265 [-0.426, -0.0953]*	Mean MD in tapetum on FA skeleton (left)
VS	ind1_0	-8.532e-03 [-0.0331, -3.883e-03]*	Mean MD in tapetum on FA skeleton (left)
VS	ind2_0	0.0311 [9.075e-04, 0.128]*	Mean MD in tapetum on FA skeleton (left)
VS	total_0	-0.223 [-0.396, -0.0501]*	Mean MD in tapetum on FA skeleton (left)
VS	beta1_1	0.179 [0.0191, 0.388]*	Mean MD in tapetum on FA skeleton (left)
VS	beta4_1	0.156 [0.023, 0.28]*	Mean MD in tapetum on FA skeleton (left)
VS	c_prime_1	-0.181 [-0.364, -8.494e-03]*	Mean MD in tapetum on FA skeleton (left)
VS	beta1_2	0.206 [0.0214, 0.399]*	Mean MD in tapetum on FA skeleton (left)
VS	beta4_2	0.263 [0.0435, 0.601]*	Mean MD in tapetum on FA skeleton (left)
VS	beta3_2	-0.2 [-0.344, -0.0939]*	Mean MD in tapetum on FA skeleton (left)
VS	c_prime_2	-0.239 [-0.413, -0.0698]*	Mean MD in tapetum on FA skeleton (left)
VS	ind3_2	-0.0552 [-0.174, -6.637e-03]*	Mean MD in tapetum on FA skeleton (left)
VS	total_2	-0.292 [-0.475, -0.112]*	Mean MD in tapetum on FA skeleton (left)
VS	diff_beta2_1_0	-0.274 [-0.475, -0.0181]*	Mean MD in tapetum on FA skeleton (left)
VS	diff_beta4_1_0	0.208 [7.417e-03, 0.426]*	Mean MD in tapetum on FA skeleton (left)
VS	diff_beta4_2_0	0.316 [0.0436, 0.685]*	Mean MD in tapetum on FA skeleton (left)
VS	diff_ind1_1_0	0.0107 [5.221e-03, 0.0436]*	Mean MD in tapetum on FA skeleton (left)
VS	diff_ind1_2_0	9.644e-03 [8.240e-04, 0.0323]*	Mean MD in tapetum on FA skeleton (left)
VS	diff_ind3_2_0	-0.0742 [-0.2, -5.038e-03]*	Mean MD in tapetum on FA skeleton (left)
VS	diff_ind_2_0	-0.0943 [-0.231, -0.0133]*	Mean MD in tapetum on FA skeleton (left)

Table S5

cognition	term	effect	description
MoCA	ind_1	-0.0399 [-0.11, -8.852e-04]*	Mean MD in retrolenticular part of internal capsule on FA skeleton (right)
EM	ind_1	-0.0499 [-0.135, -5.809e-03]*	Mean MD in retrolenticular part of internal capsule on FA skeleton (right)
MMSE	ind_1	-0.0311 [-0.121, -4.083e-04]*	Mean MD in retrolenticular part of internal capsule on FA skeleton (left)
MMSE	ind_2	-0.0387 [-0.138, -1.731e-03]*	Mean MD in retrolenticular part of internal capsule on FA skeleton (left)
MMSE	diff_ind_1_0	-0.0649 [-0.19, -1.219e-03]*	Mean MD in retrolenticular part of internal capsule on FA skeleton (left)
MMSE	diff_ind_2_0	-0.0725 [-0.227, -8.518e-03]*	Mean MD in retrolenticular part of internal capsule on FA skeleton (left)
MoCA	ind_2	-0.0304 [-0.112, -1.811e-04]*	Mean MD in retrolenticular part of internal capsule on FA skeleton (left)
MoCA	diff_ind_2_0	-0.0535 [-0.158, -3.070e-03]*	Mean MD in retrolenticular part of internal capsule on FA skeleton (left)
EM	ind_1	-0.0481 [-0.136, -4.012e-03]*	Mean MD in retrolenticular part of internal capsule on FA skeleton (left)
MMSE	diff_ind_2_0	-0.0787 [-0.216, -1.456e-03]*	Mean MD in superior corona radiata on FA skeleton (right)
MoCA	diff_ind_2_0	-0.0518 [-0.152, -1.518e-04]*	Mean MD in superior corona radiata on FA skeleton (right)
EM	ind_1	-0.0427 [-0.15, -6.541e-04]*	Mean MD in superior corona radiata on FA skeleton (right)
EM	ind_1	-0.0449 [-0.154, -7.223e-04]*	Mean MD in superior corona radiata on FA skeleton (left)
MMSE	ind_2	-0.0557 [-0.154, -0.013]*	Mean MD in posterior corona radiata on FA skeleton (right)
MMSE	diff_ind_2_0	-0.0848 [-0.242, -0.0156]*	Mean MD in posterior corona radiata on FA skeleton (right)
MoCA	ind_1	-0.043 [-0.125, -2.504e-03]*	Mean MD in posterior corona radiata on FA skeleton (right)
EM	ind_1	-0.0527 [-0.15, -9.172e-03]*	Mean MD in posterior corona radiata on FA skeleton (right)
MMSE	ind_1	-0.0443 [-0.146, -2.435e-03]*	Mean MD in posterior corona radiata on FA skeleton (left)
MMSE	ind_2	-0.046 [-0.15, -1.621e-03]*	Mean MD in posterior corona radiata on FA skeleton (left)
MMSE	diff_ind_2_0	-0.069 [-0.246, -4.690e-03]*	Mean MD in posterior corona radiata on FA skeleton (left)
MoCA	ind_2	-0.041 [-0.129, -4.711e-03]*	Mean MD in posterior corona radiata on FA skeleton (left)
MoCA	diff_ind_2_0	-0.0521 [-0.179, -3.498e-03]*	Mean MD in posterior corona radiata on FA skeleton (left)
EM	ind_1	-0.0599 [-0.165, -0.0122]*	Mean MD in posterior corona radiata on FA skeleton (left)
EM	diff_ind_1_0	-0.0412 [-0.195, -6.274e-04]*	Mean MD in posterior corona radiata on FA skeleton (left)
MMSE	ind_1	-0.0397 [-0.128, -6.378e-04]*	Mean MD in posterior thalamic radiation on FA skeleton (right)
MMSE	ind_2	-0.0473 [-0.159, -3.530e-03]*	Mean MD in posterior thalamic radiation on FA skeleton (right)
MoCA	ind_1	-0.0414 [-0.112, -2.661e-03]*	Mean MD in posterior thalamic radiation on FA skeleton (right)
EM	ind_1	-0.0663 [-0.17, -0.0153]*	Mean MD in posterior thalamic radiation on FA skeleton (right)
EF	ind_2	-0.0331 [-0.115, -2.244e-03]*	Mean MD in posterior thalamic radiation on FA skeleton (left)
EF	diff_ind_2_0	-0.0395 [-0.136, -9.113e-04]*	Mean MD in posterior thalamic radiation on FA skeleton (left)
MMSE	ind_1	-0.0468 [-0.127, -6.372e-03]*	Mean MD in sagittal stratum on FA skeleton (right)
MMSE	ind_2	-0.046 [-0.139, -3.210e-03]*	Mean MD in sagittal stratum on FA skeleton (right)
MMSE	diff_ind_1_0	-0.0871 [-0.212, -4.501e-03]*	Mean MD in sagittal stratum on FA skeleton (right)
MMSE	diff_ind_2_0	-0.0863 [-0.226, -5.207e-03]*	Mean MD in sagittal stratum on FA skeleton (right)
MoCA	ind_1	-0.0526 [-0.119, -0.01]*	Mean MD in sagittal stratum on FA skeleton (right)
MoCA	diff_ind_1_0	-0.0784 [-0.171, -0.0103]*	Mean MD in sagittal stratum on FA skeleton (right)
MMSE	ind_1	-0.0638 [-0.21, -9.408e-03]*	Mean MD in sagittal stratum on FA skeleton (left)
MMSE	ind_2	-0.0465 [-0.15, -3.972e-03]*	Mean MD in sagittal stratum on FA skeleton (left)
MMSE	diff_ind_1_0	-0.102 [-0.263, -0.0175]*	Mean MD in sagittal stratum on FA skeleton (left)
MMSE	diff_ind_2_0	-0.0844 [-0.236, -0.0152]*	Mean MD in sagittal stratum on FA skeleton (left)
MoCA	ind_1	-0.0602 [-0.179, -0.0101]*	Mean MD in sagittal stratum on FA skeleton (left)
MoCA	ind_2	-0.0387 [-0.121, -6.484e-04]*	Mean MD in sagittal stratum on FA skeleton (left)
MoCA	diff_ind_1_0	-0.0871 [-0.211, -0.0205]*	Mean MD in sagittal stratum on FA skeleton (left)
MoCA	diff_ind_2_0	-0.0656 [-0.177, -0.0136]*	Mean MD in sagittal stratum on FA skeleton (left)
EM	ind_1	-0.0481 [-0.142, -2.760e-03]*	Mean MD in sagittal stratum on FA skeleton (left)
EF	ind_1	-0.0436 [-0.15, -1.421e-03]*	Mean MD in sagittal stratum on FA skeleton (left)
EF	diff_ind_1_0	-0.0564 [-0.164, -5.673e-03]*	Mean MD in sagittal stratum on FA skeleton (left)
MMSE	ind_2	-0.0568 [-0.163, -0.0149]*	Mean MD in cingulum cingulate gyrus on FA skeleton (left)
MMSE	diff_ind_1_0	-0.0812 [-0.195, -4.294e-03]*	Mean MD in cingulum cingulate gyrus on FA skeleton (left)
MMSE	diff_ind_2_0	-0.108 [-0.25, -0.0302]*	Mean MD in cingulum cingulate gyrus on FA skeleton (left)
MoCA	diff_ind_1_0	-0.0704 [-0.159, -8.256e-03]*	Mean MD in cingulum cingulate gyrus on FA skeleton (left)
MoCA	diff_ind_2_0	-0.063 [-0.17, -7.105e-03]*	Mean MD in cingulum cingulate gyrus on FA skeleton (left)
EM	ind_1	-0.0485 [-0.127, -8.712e-03]*	Mean MD in cingulum cingulate gyrus on FA skeleton (left)
MMSE	ind_1	-0.136 [-0.315, -0.0455]*	Mean MD in cingulum hippocampus on FA skeleton (left)
MMSE	ind_2	-0.0839 [-0.204, -0.0241]*	Mean MD in cingulum hippocampus on FA skeleton (left)
MMSE	diff_ind_1_0	-0.146 [-0.335, -0.0237]*	Mean MD in cingulum hippocampus on FA skeleton (left)
MoCA	ind_1	-0.113 [-0.263, -0.0321]*	Mean MD in cingulum hippocampus on FA skeleton (left)
MoCA	ind_2	-0.0635 [-0.174, -3.671e-03]*	Mean MD in cingulum hippocampus on FA skeleton (left)
MoCA	diff_ind_1_0	-0.107 [-0.275, -9.214e-03]*	Mean MD in cingulum hippocampus on FA skeleton (left)
MMSE	ind_1	-0.0311 [-0.104, -2.083e-04]*	Mean MD in superior longitudinal fasciculus on FA skeleton (right)
MMSE	ind_2	-0.0544 [-0.159, -7.820e-03]*	Mean MD in superior longitudinal fasciculus on FA skeleton (right)
MMSE	diff_ind_1_0	-0.0665 [-0.178, -3.124e-04]*	Mean MD in superior longitudinal fasciculus on FA skeleton (right)
MMSE	diff_ind_2_0	-0.0899 [-0.233, -0.0163]*	Mean MD in superior longitudinal fasciculus on FA skeleton (right)
EM	ind_1	-0.0401 [-0.124, -3.531e-03]*	Mean MD in superior longitudinal fasciculus on FA skeleton (right)
MMSE	ind_1	-0.0487 [-0.14, -7.639e-03]*	Mean MD in superior longitudinal fasciculus on FA skeleton (left)
MMSE	ind_2	-0.0356 [-0.135, -1.592e-03]*	Mean MD in superior longitudinal fasciculus on FA skeleton (left)
MMSE	diff_ind_1_0	-0.0837 [-0.203, -0.0134]*	Mean MD in superior longitudinal fasciculus on FA skeleton (left)
MMSE	diff_ind_2_0	-0.0706 [-0.217, -7.459e-03]*	Mean MD in superior longitudinal fasciculus on FA skeleton (left)
MoCA	ind_1	-0.044 [-0.123, -2.144e-03]*	Mean MD in superior longitudinal fasciculus on FA skeleton (left)
MoCA	diff_ind_1_0	-0.0666 [-0.151, -7.577e-03]*	Mean MD in superior longitudinal fasciculus on FA skeleton (left)
MoCA	diff_ind_2_0	-0.0513 [-0.15, -3.918e-03]*	Mean MD in superior longitudinal fasciculus on FA skeleton (left)
EM	ind_1	-0.0652 [-0.153, -0.0151]*	Mean MD in superior longitudinal fasciculus on FA skeleton (left)
EM	ind_2	-0.0332 [-0.099, -1.485e-04]*	Mean MD in superior longitudinal fasciculus on FA skeleton (left)
EM	diff_ind_1_0	-0.0571 [-0.159, -1.257e-03]*	Mean MD in superior longitudinal fasciculus on FA skeleton (left)
MMSE	ind_1	-0.0614 [-0.153, -7.543e-03]*	Mean MD in tapetum on FA skeleton (left)
MMSE	ind_2	-0.0951 [-0.225, -0.0308]*	Mean MD in tapetum on FA skeleton (left)
MMSE	diff_ind_1_0	-0.116 [-0.247, -0.0177]*	Mean MD in tapetum on FA skeleton (left)

MMSE	diff_ind_2_0	-0.149 [-0.315, -0.0494]*	Mean MD in tapetum on FA skeleton (left)
MoCA	ind_1	-0.0505 [-0.123, -1.749e-04]*	Mean MD in tapetum on FA skeleton (left)
MoCA	ind_2	-0.099 [-0.243, -0.0231]*	Mean MD in tapetum on FA skeleton (left)
MoCA	diff_ind_1_0	-0.0859 [-0.187, -6.172e-03]*	Mean MD in tapetum on FA skeleton (left)
MoCA	diff_ind_2_0	-0.135 [-0.292, -0.036]*	Mean MD in tapetum on FA skeleton (left)
EF	ind_2	-0.0483 [-0.135, -7.824e-03]*	Mean MD in tapetum on FA skeleton (left)
EF	diff_ind_2_0	-0.0616 [-0.155, -8.683e-03]*	Mean MD in tapetum on FA skeleton (left)
VS	diff_ind_2_0	-0.0943 [-0.231, -0.0133]*	Mean MD in tapetum on FA skeleton (left)

Table S6

description	effect of ptau181	effect of ptau181*WMHV tertile
Volume of grey matter in Frontal Pole (left)	β = -0.024, p = 0.8, pFDR = 0.99	B = -0.029, p = 0.64, pFDR = 0.92
Volume of grey matter in Frontal Pole (right)	β = -0.029, p = 0.76, pFDR = 0.99	B = 0.015, p = 0.81, pFDR = 0.92
Volume of grey matter in Insular Cortex (left)	β = -0.013, p = 0.89, pFDR = 0.99	B = -0.016, p = 0.8, pFDR = 0.92
Volume of grey matter in Insular Cortex (right)	β = 0.042, p = 0.67, pFDR = 0.99	B = -0.052, p = 0.42, pFDR = 0.92
Volume of grey matter in Superior Frontal Gyrus (left)	β = 0.02, p = 0.84, pFDR = 0.99	B = -0.097, p = 0.13, pFDR = 0.72
Volume of grey matter in Superior Frontal Gyrus (right)	β = 0.14, p = 0.14, pFDR = 0.63	B = -0.15, p = 0.017, pFDR = 0.72
Volume of grey matter in Middle Frontal Gyrus (left)	β = 0.23, p = 0.022, pFDR = 0.34	B = -0.1, p = 0.12, pFDR = 0.72
Volume of grey matter in Middle Frontal Gyrus (right)	β = -0.024, p = 0.81, pFDR = 0.99	B = -0.034, p = 0.6, pFDR = 0.92
Volume of grey matter in Inferior Frontal Gyrus, pars triangularis (left)	β = -0.15, p = 0.14, pFDR = 0.63	B = 0.052, p = 0.43, pFDR = 0.92
Volume of grey matter in Inferior Frontal Gyrus, pars triangularis (right)	β = -0.11, p = 0.25, pFDR = 0.72	B = 0.039, p = 0.55, pFDR = 0.92
Volume of grey matter in Inferior Frontal Gyrus, pars opercularis (left)	β = -0.034, p = 0.73, pFDR = 0.99	B = 0.051, p = 0.43, pFDR = 0.92
Volume of grey matter in Inferior Frontal Gyrus, pars opercularis (right)	β = 0.018, p = 0.85, pFDR = 0.99	B = 1.40e-03, p = 0.98, pFDR = 0.98
Volume of grey matter in Precentral Gyrus (left)	β = 0.18, p = 0.04, pFDR = 0.46	B = -0.079, p = 0.17, pFDR = 0.72
Volume of grey matter in Precentral Gyrus (right)	β = 0.056, p = 0.54, pFDR = 0.93	B = -0.092, p = 0.13, pFDR = 0.72
Volume of grey matter in Temporal Pole (left)	β = -0.048, p = 0.62, pFDR = 0.96	B = -0.02, p = 0.76, pFDR = 0.92
Volume of grey matter in Temporal Pole (right)	β = -0.037, p = 0.7, pFDR = 0.99	B = -0.028, p = 0.66, pFDR = 0.92
Volume of grey matter in Superior Temporal Gyrus, anterior division (left)	β = 0.024, p = 0.81, pFDR = 0.99	B = -0.089, p = 0.18, pFDR = 0.72
Volume of grey matter in Superior Temporal Gyrus, anterior division (right)	β = -0.096, p = 0.34, pFDR = 0.79	B = 0.016, p = 0.8, pFDR = 0.92
Volume of grey matter in Superior Temporal Gyrus, posterior division (left)	β = -0.066, p = 0.51, pFDR = 0.93	B = -0.013, p = 0.84, pFDR = 0.92
Volume of grey matter in Superior Temporal Gyrus, posterior division (right)	β = -0.12, p = 0.25, pFDR = 0.72	B = -0.011, p = 0.87, pFDR = 0.93
Volume of grey matter in Middle Temporal Gyrus, anterior division (left)	β = -0.026, p = 0.8, pFDR = 0.99	B = -0.037, p = 0.58, pFDR = 0.92
Volume of grey matter in Middle Temporal Gyrus, anterior division (right)	β = 7.15e-03, p = 0.94, pFDR = 0.99	B = -0.077, p = 0.25, pFDR = 0.74
Volume of grey matter in Middle Temporal Gyrus, posterior division (left)	β = -0.023, p = 0.82, pFDR = 0.99	B = -0.096, p = 0.14, pFDR = 0.72
Volume of grey matter in Middle Temporal Gyrus, posterior division (right)	β = -0.12, p = 0.22, pFDR = 0.72	B = -0.022, p = 0.74, pFDR = 0.92
Volume of grey matter in Middle Temporal Gyrus, temporooccipital part (left)	β = -0.013, p = 0.9, pFDR = 0.99	B = -0.13, p = 0.054, pFDR = 0.72
Volume of grey matter in Middle Temporal Gyrus, temporooccipital part (right)	β = -0.29, p = 3.55e-03, pFDR = 0.15	B = 0.13, p = 0.055, pFDR = 0.72
Volume of grey matter in Inferior Temporal Gyrus, anterior division (left)	β = 0.016, p = 0.87, pFDR = 0.99	B = -0.056, p = 0.39, pFDR = 0.92
Volume of grey matter in Inferior Temporal Gyrus, anterior division (right)	β = -0.15, p = 0.14, pFDR = 0.63	B = 0.071, p = 0.28, pFDR = 0.77
Volume of grey matter in Inferior Temporal Gyrus, posterior division (left)	β = -0.019, p = 0.85, pFDR = 0.99	B = -0.041, p = 0.54, pFDR = 0.92
Volume of grey matter in Inferior Temporal Gyrus, posterior division (right)	β = -0.068, p = 0.5, pFDR = 0.93	B = -0.049, p = 0.46, pFDR = 0.92
Volume of grey matter in Inferior Temporal Gyrus, temporooccipital part (left)	β = -0.15, p = 0.13, pFDR = 0.63	B = -1.46e-03, p = 0.98, pFDR = 0.98
Volume of grey matter in Inferior Temporal Gyrus, temporooccipital part (right)	β = -0.063, p = 0.52, pFDR = 0.93	B = -0.083, p = 0.21, pFDR = 0.72
Volume of grey matter in Postcentral Gyrus (left)	β = 0.21, p = 0.014, pFDR = 0.29	B = -0.15, p = 8.83e-03, pFDR = 0.72
Volume of grey matter in Postcentral Gyrus (right)	β = 0.1, p = 0.25, pFDR = 0.72	B = -0.13, p = 0.033, pFDR = 0.72
Volume of grey matter in Superior Parietal Lobule (left)	β = 0.015, p = 0.88, pFDR = 0.99	B = -0.024, p = 0.71, pFDR = 0.92
Volume of grey matter in Superior Parietal Lobule (right)	β = -8.18e-04, p = 0.99, pFDR = 0.99	B = -0.065, p = 0.32, pFDR = 0.79
Volume of grey matter in Supramarginal Gyrus, anterior division (left)	β = 0.15, p = 0.14, pFDR = 0.63	B = -0.13, p = 0.053, pFDR = 0.72
Volume of grey matter in Supramarginal Gyrus, anterior division (right)	β = -0.18, p = 0.061, pFDR = 0.53	B = 0.041, p = 0.52, pFDR = 0.92
Volume of grey matter in Supramarginal Gyrus, posterior division (left)	β = -0.098, p = 0.32, pFDR = 0.79	B = 0.015, p = 0.81, pFDR = 0.92
Volume of grey matter in Supramarginal Gyrus, posterior division (right)	β = -0.058, p = 0.56, pFDR = 0.93	B = -0.017, p = 0.8, pFDR = 0.92
Volume of grey matter in Angular Gyrus (left)	β = -0.083, p = 0.41, pFDR = 0.88	B = 0.044, p = 0.51, pFDR = 0.92
Volume of grey matter in Angular Gyrus (right)	β = -0.098, p = 0.32, pFDR = 0.79	B = 0.017, p = 0.8, pFDR = 0.92
Volume of grey matter in Lateral Occipital Cortex, superior division (left)	β = -0.17, p = 0.079, pFDR = 0.61	B = -0.026, p = 0.67, pFDR = 0.92
Volume of grey matter in Lateral Occipital Cortex, superior division (right)	β = -0.091, p = 0.35, pFDR = 0.79	B = -0.072, p = 0.27, pFDR = 0.76
Volume of grey matter in Lateral Occipital Cortex, inferior division (left)	β = -0.1, p = 0.3, pFDR = 0.78	B = -0.055, p = 0.4, pFDR = 0.92
Volume of grey matter in Lateral Occipital Cortex, inferior division (right)	β = -0.12, p = 0.21, pFDR = 0.72	B = -0.018, p = 0.78, pFDR = 0.92
Volume of grey matter in Intracalcarine Cortex (left)	β = 0.11, p = 0.25, pFDR = 0.72	B = -0.031, p = 0.63, pFDR = 0.92
Volume of grey matter in Intracalcarine Cortex (right)	β = 0.11, p = 0.25, pFDR = 0.72	B = -0.019, p = 0.77, pFDR = 0.92
Volume of grey matter in Frontal Medial Cortex (left)	β = -0.027, p = 0.79, pFDR = 0.99	B = -1.45e-03, p = 0.98, pFDR = 0.98
Volume of grey matter in Frontal Medial Cortex (right)	β = -0.049, p = 0.61, pFDR = 0.96	B = -1.53e-03, p = 0.98, pFDR = 0.98
Volume of grey matter in Juxtapositional Lobule Cortex (formerly Supplementary Motor Cortex) (left)	β = 0.12, p = 0.22, pFDR = 0.72	B = -0.086, p = 0.19, pFDR = 0.72
Volume of grey matter in Juxtapositional Lobule Cortex (formerly Supplementary Motor Cortex) (right)	β = 0.095, p = 0.34, pFDR = 0.79	B = -0.084, p = 0.2, pFDR = 0.72
Volume of grey matter in Subcallosal Cortex (left)	β = -0.16, p = 0.12, pFDR = 0.63	B = 0.066, p = 0.32, pFDR = 0.79
Volume of grey matter in Subcallosal Cortex (right)	β = -0.055, p = 0.58, pFDR = 0.94	B = 0.022, p = 0.74, pFDR = 0.92
Volume of grey matter in Paracingulate Gyrus (left)	β = 0.17, p = 0.1, pFDR = 0.62	B = -0.077, p = 0.25, pFDR = 0.74
Volume of grey matter in Paracingulate Gyrus (right)	β = 0.19, p = 0.057, pFDR = 0.53	B = -0.11, p = 0.097, pFDR = 0.72
Volume of grey matter in Cingulate Gyrus, anterior division (left)	β = 0.018, p = 0.86, pFDR = 0.99	B = 0.082, p = 0.22, pFDR = 0.72
Volume of grey matter in Cingulate Gyrus, anterior division (right)	β = 0.091, p = 0.35, pFDR = 0.79	B = 0.026, p = 0.69, pFDR = 0.92
Volume of grey matter in Cingulate Gyrus, posterior division (left)	β = -0.028, p = 0.78, pFDR = 0.99	B = 3.83e-03, p = 0.95, pFDR = 0.98
Volume of grey matter in Cingulate Gyrus, posterior division (right)	β = -0.13, p = 0.2, pFDR = 0.72	B = 0.039, p = 0.55, pFDR = 0.92
Volume of grey matter in Precuneous Cortex (left)	β = -2.82e-03, p = 0.98, pFDR = 0.99	B = -0.1, p = 0.11, pFDR = 0.72
Volume of grey matter in Precuneous Cortex (right)	β = -0.052, p = 0.59, pFDR = 0.95	B = -0.082, p = 0.21, pFDR = 0.72
Volume of grey matter in Cuneal Cortex (left)	β = 0.07, p = 0.49, pFDR = 0.93	B = -0.069, p = 0.3, pFDR = 0.79
Volume of grey matter in Cuneal Cortex (right)	β = -9.08e-04, p = 0.99, pFDR = 0.99	B = -8.13e-03, p = 0.9, pFDR = 0.96
Volume of grey matter in Frontal Orbital Cortex (left)	β = -0.014, p = 0.89, pFDR = 0.99	B = -0.019, p = 0.77, pFDR = 0.92
Volume of grey matter in Frontal Orbital Cortex (right)	β = 0.023, p = 0.81, pFDR = 0.99	B = -0.02, p = 0.76, pFDR = 0.92
Volume of grey matter in Parahippocampal Gyrus, anterior division (left)	β = -0.15, p = 0.12, pFDR = 0.63	B = 0.016, p = 0.8, pFDR = 0.92
Volume of grey matter in Parahippocampal Gyrus, anterior division (right)	β = -0.18, p = 0.064, pFDR = 0.53	B = 0.11, p = 0.098, pFDR = 0.72
Volume of grey matter in Parahippocampal Gyrus, posterior division (left)	β = 0.022, p = 0.82, pFDR = 0.99	B = -0.074, p = 0.25, pFDR = 0.74
Volume of grey matter in Parahippocampal Gyrus, posterior division (right)	β = -0.074, p = 0.42, pFDR = 0.89	B = 0.043, p = 0.48, pFDR = 0.92
Volume of grey matter in Lingual Gyrus (left)	β = -0.02, p = 0.84, pFDR = 0.99	B = 0.023, p = 0.72, pFDR = 0.92
Volume of grey matter in Lingual Gyrus (right)	β = 0.058, p = 0.55, pFDR = 0.93	B = -0.033, p = 0.61, pFDR = 0.92
Volume of grey matter in Temporal Fusiform Cortex, anterior division (left)	β = 0.1, p = 0.29, pFDR = 0.78	B = -0.088, p = 0.18, pFDR = 0.72
Volume of grey matter in Temporal Fusiform Cortex, anterior division (right)	β = -0.059, p = 0.55, pFDR = 0.93	B = 0.028, p = 0.67, pFDR = 0.92
Volume of grey matter in Temporal Fusiform Cortex, posterior division (left)	β = -0.011, p = 0.91, pFDR = 0.99	B = -0.025, p = 0.71, pFDR = 0.92
Volume of grey matter in Temporal Fusiform Cortex, posterior division (right)	β = -0.11, p = 0.25, pFDR = 0.72	B = 0.062, p = 0.33, pFDR = 0.82
Volume of grey matter in Temporal Occipital Fusiform Cortex (left)	β = -0.069, p = 0.49, pFDR = 0.93	B = 0.023, p = 0.73, pFDR = 0.92
Volume of grey matter in Temporal Occipital Fusiform Cortex (right)	β = -0.13, p = 0.19, pFDR = 0.72	B = 0.02, p = 0.76, pFDR = 0.92
Volume of grey matter in Occipital Fusiform Gyrus (left)	β = 0.088, p = 0.35, pFDR = 0.79	B = -0.09, p = 0.15, pFDR = 0.72
Volume of grey matter in Occipital Fusiform Gyrus (right)	β = 0.013, p = 0.89, pFDR = 0.99	B = -0.085, p = 0.18, pFDR = 0.72
Volume of grey matter in Frontal Operculum Cortex (left)	β = 0.068, p = 0.49, pFDR = 0.93	B = -0.021, p = 0.75, pFDR = 0.92
Volume of grey matter in Frontal Operculum Cortex (right)	β = 0.039, p = 0.69, pFDR = 0.99	B = -0.038, p = 0.57, pFDR = 0.92
Volume of grey matter in Central Opercular Cortex (left)	β = -0.066, p = 0.51, pFDR = 0.93	B = 0.074, p = 0.26, pFDR = 0.76
Volume of grey matter in Central Opercular Cortex (right)	β = 0.1, p = 0.3, pFDR = 0.78	B = -0.1, p = 0.13, pFDR = 0.72
Volume of grey matter in Parietal Operculum Cortex (left)	β = 0.2, p = 0.047, pFDR = 0.49	B = -0.085, p = 0.2, pFDR = 0.72
Volume of grey matter in Parietal Operculum Cortex (right)	β = 0.035, p = 0.73, pFDR = 0.99	B = -0.034, p = 0.61, pFDR = 0.92
Volume of grey matter in Planum Polare (left)	β = 0.078, p = 0.43, pFDR = 0.9	B = -0.047, p = 0.48, pFDR = 0.92
Volume of grey matter in Planum Polare (right)	β = 0.12, p = 0.2, pFDR = 0.72	B = -0.099, p = 0.12, pFDR = 0.72
Volume of grey matter in Heschl's Gyrus (includes H1 and H2) (left)	β = 0.22, p = 0.022, pFDR = 0.34	B = -0.12, p = 0.051, pFDR = 0.72
Volume of grey matter in Heschl's Gyrus (includes H1 and H2) (right)	β = 0.089, p = 0.35, pFDR = 0.79	B = -0.082, p = 0.2, pFDR = 0.72
Volume of grey matter in Planum Temporale (left)	β = 0.17, p = 0.092, pFDR = 0.62	B = -0.1, p = 0.12, pFDR = 0.72
Volume of grey matter in Planum Temporale (right)	β = -8.05e-03, p = 0.94, pFDR = 0.99	B = -0.018, p = 0.79, pFDR = 0.92
Volume of grey matter in Supracalcarine Cortex (left)	β = -0.057, p = 0.57, pFDR = 0.94	B = 0.051, p = 0.44, pFDR = 0.92
Volume of grey matter in Supracalcarine Cortex (right)	β = 4.04e-03, p = 0.97, pFDR = 0.99	B = 0.047, p = 0.48, pFDR = 0.92
Volume of grey matter in Occipital Pole (left)	β = -0.059, p = 0.54, pFDR = 0.93	B = -0.04, p = 0.53, pFDR = 0.92
Volume of grey matter in Occipital Pole (right)	β = -0.059, p = 0.53, pFDR = 0.93	B = -0.024, p = 0.7, pFDR = 0.92
Volume of grey matter in Thalamus (left)	β = -0.013, p = 0.89, pFDR = 0.99	B = 0.073, p = 0.24, pFDR = 0.74
Volume of grey matter in Thalamus (right)	β = 0.068, p = 0.47, pFDR = 0.93	B = -0.023, p = 0.71, pFDR = 0.92
Volume of grey matter in Caudate (left)	β = -0.13, p = 0.14, pFDR = 0.63	B = 0.11, p = 0.062, pFDR = 0.72
Volume of grey matter in Caudate (right)	β = -0.11, p = 0.2, pFDR = 0.72	B = 0.087, p = 0.14, pFDR = 0.72
Volume of grey matter in Putamen (left)	β = -0.15, p = 0.099, pFDR = 0.62	B = 0.048, p = 0.44, pFDR = 0.92
Volume of grey matter in Putamen (right)	β = -0.19, p = 0.036, pFDR = 0.46	B = 0.1, p = 0.1, pFDR = 0.72
Volume of grey matter in Pallidum (left)	β = 3.36e-03, p = 0.97, pFDR = 0.99	B = 0.077, p = 0.21, pFDR = 0.72
Volume of grey matter in Pallidum (right)	β = 0.017, p = 0.86, pFDR = 0.99	B = 0.096, p = 0.12, pFDR = 0.72
Volume of grey matter in Hippocampus (left)	β = -0.24, p = 5.59e-03, pFDR = 0.17	B = 6.71e-03, p = 0.91, pFDR = 0.96
Volume of grey matter in Hippocampus (right)	β = -0.24, p = 7.13e-03, pFDR = 0.18	B = 0.024, p = 0.67, pFDR = 0.92
Volume of grey matter in Amygdala (left)	β = -0.27, p = 2.61e-03, pFDR = 0.15	B = 0.1, p = 0.083, pFDR = 0.72
Volume of grey matter in Amygdala (right)	β = -0.31, p = 4.40e-04, pFDR = 0.055	B = 0.14, p = 0.018, pFDR = 0.72
Volume of grey matter in Ventral Striatum (left)	β = 2.46e-03, p = 0.98, pFDR = 0.99	B = -0.019, p = 0.76, pFDR = 0.92
Volume of grey matter in Ventral Striatum (right)	β = 8.50e-03, p = 0.93, pFDR = 0.99	B = 0.011, p = 0.86, pFDR = 0.93
Volume of grey matter in Frontal Pole (left)	β = -0.014, p = 0.87, pFDR = 1	B = -0.019, p = 0.74, pFDR = 0.95
Volume of grey matter in Frontal Pole (right)	β = -0.019, p = 0.84, pFDR = 1	B = 0.018, p = 0.77, pFDR = 0.95
Volume of grey matter in Insular Cortex (left)	β = 7.49e-03, p = 0.93, pFDR = 1	B = -0.016, p = 0.77, pFDR = 0.95

**Volume of grey matter in Insular Cortex (right)**	β = 0.046, p = 0.58, pFDR = 0.99	B = -0.038, p = 0.48, pFDR = 0.95
**Volume of grey matter in Superior Frontal Gyrus (left)**	β = 0.017, p = 0.85, pFDR = 1	B = -0.084, p = 0.17, pFDR = 0.78
**Volume of grey matter in Superior Frontal Gyrus (right)**	β = 0.12, p = 0.19, pFDR = 0.75	B = -0.13, p = 0.032, pFDR = 0.78
**Volume of grey matter in Middle Frontal Gyrus (left)**	β = 0.21, p = 0.025, pFDR = 0.39	B = -0.089, p = 0.16, pFDR = 0.78
**Volume of grey matter in Middle Frontal Gyrus (right)**	β = -0.017, p = 0.85, pFDR = 1	B = -0.026, p = 0.67, pFDR = 0.95
**Volume of grey matter in Inferior Frontal Gyrus, pars triangularis (left)**	β = -0.14, p = 0.15, pFDR = 0.75	B = 0.053, p = 0.41, pFDR = 0.94
**Volume of grey matter in Inferior Frontal Gyrus, pars triangularis (right)**	β = -0.1, p = 0.29, pFDR = 0.75	B = 0.042, p = 0.52, pFDR = 0.95
**Volume of grey matter in Inferior Frontal Gyrus, pars opercularis (left)**	β = -0.025, p = 0.8, pFDR = 1	B = 0.052, p = 0.43, pFDR = 0.95
**Volume of grey matter in Inferior Frontal Gyrus, pars opercularis (right)**	β = 0.013, p = 0.89, pFDR = 1	B = 8.50e-03, p = 0.89, pFDR = 0.95
**Volume of grey matter in Precentral Gyrus (left)**	β = 0.18, p = 0.046, pFDR = 0.48	B = -0.069, p = 0.24, pFDR = 0.78
**Volume of grey matter in Precentral Gyrus (right)**	β = 0.052, p = 0.56, pFDR = 0.97	B = -0.077, p = 0.19, pFDR = 0.78
**Volume of grey matter in Temporal Pole (left)**	β = -0.047, p = 0.62, pFDR = 1	B = -0.014, p = 0.82, pFDR = 0.95
**Volume of grey matter in Temporal Pole (right)**	β = -0.029, p = 0.77, pFDR = 1	B = -0.024, p = 0.7, pFDR = 0.95
**Volume of grey matter in Superior Temporal Gyrus, anterior division (left)**	β = 0.021, p = 0.83, pFDR = 1	B = -0.082, p = 0.19, pFDR = 0.78
**Volume of grey matter in Superior Temporal Gyrus, anterior division (right)**	β = -0.086, p = 0.36, pFDR = 0.83	B = 0.015, p = 0.81, pFDR = 0.95
**Volume of grey matter in Superior Temporal Gyrus, posterior division (left)**	β = -0.063, p = 0.5, pFDR = 0.94	B = -9.63e-03, p = 0.88, pFDR = 0.95
**Volume of grey matter in Superior Temporal Gyrus, posterior division (right)**	β = -0.096, p = 0.28, pFDR = 0.75	B = -4.70e-03, p = 0.94, pFDR = 0.95
**Volume of grey matter in Middle Temporal Gyrus, anterior division (left)**	β = -0.02, p = 0.83, pFDR = 1	B = -0.03, p = 0.63, pFDR = 0.95
**Volume of grey matter in Middle Temporal Gyrus, anterior division (right)**	β = 0.022, p = 0.82, pFDR = 1	B = -0.079, p = 0.21, pFDR = 0.78
**Volume of grey matter in Middle Temporal Gyrus, posterior division (left)**	β = -0.021, p = 0.83, pFDR = 1	B = -0.085, p = 0.17, pFDR = 0.78
**Volume of grey matter in Middle Temporal Gyrus, posterior division (right)**	β = -0.1, p = 0.28, pFDR = 0.75	B = -0.024, p = 0.7, pFDR = 0.95
**Volume of grey matter in Middle Temporal Gyrus, temporooccipital part (left)**	β = -0.021, p = 0.82, pFDR = 1	B = -0.11, p = 0.085, pFDR = 0.78
**Volume of grey matter in Middle Temporal Gyrus, temporooccipital part (right)**	β = -0.29, p = 2.66e-03, pFDR = 0.17	B = 0.13, p = 0.048, pFDR = 0.78
**Volume of grey matter in Inferior Temporal Gyrus, anterior division (left)**	β = 0.032, p = 0.74, pFDR = 1	B = -0.054, p = 0.39, pFDR = 0.94
**Volume of grey matter in Inferior Temporal Gyrus, anterior division (right)**	β = -0.14, p = 0.13, pFDR = 0.69	B = 0.08, p = 0.21, pFDR = 0.78
**Volume of grey matter in Inferior Temporal Gyrus, posterior division (left)**	β = -3.25e-03, p = 0.97, pFDR = 1	B = -0.037, p = 0.54, pFDR = 0.95
**Volume of grey matter in Inferior Temporal Gyrus, posterior division (right)**	β = -0.076, p = 0.42, pFDR = 0.88	B = -0.033, p = 0.59, pFDR = 0.95
**Volume of grey matter in Inferior Temporal Gyrus, temporooccipital part (left)**	β = -0.14, p = 0.12, pFDR = 0.69	B = 4.51e-03, p = 0.94, pFDR = 0.95
**Volume of grey matter in Inferior Temporal Gyrus, temporooccipital part (right)**	β = -0.066, p = 0.47, pFDR = 0.92	B = -0.067, p = 0.27, pFDR = 0.81
**Volume of grey matter in Postcentral Gyrus (left)**	β = 0.21, p = 0.016, pFDR = 0.29	B = -0.14, p = 0.015, pFDR = 0.78
**Volume of grey matter in Postcentral Gyrus (right)**	β = 0.1, p = 0.26, pFDR = 0.75	B = -0.12, p = 0.049, pFDR = 0.78
**Volume of grey matter in Superior Parietal Lobule (left)**	β = 0.011, p = 0.91, pFDR = 1	B = -0.016, p = 0.8, pFDR = 0.95
**Volume of grey matter in Superior Parietal Lobule (right)**	β = 7.49e-03, p = 0.94, pFDR = 1	B = -0.066, p = 0.31, pFDR = 0.87
**Volume of grey matter in Supramarginal Gyrus, anterior division (left)**	β = 0.15, p = 0.13, pFDR = 0.69	B = -0.12, p = 0.062, pFDR = 0.78
**Volume of grey matter in Supramarginal Gyrus, anterior division (right)**	β = -0.18, p = 0.065, pFDR = 0.58	B = 0.042, p = 0.52, pFDR = 0.95
**Volume of grey matter in Supramarginal Gyrus, posterior division (left)**	β = -0.096, p = 0.31, pFDR = 0.76	B = 0.022, p = 0.72, pFDR = 0.95
**Volume of grey matter in Supramarginal Gyrus, posterior division (right)**	β = -0.044, p = 0.66, pFDR = 1	B = -0.023, p = 0.73, pFDR = 0.95
**Volume of grey matter in Angular Gyrus (left)**	β = -0.082, p = 0.39, pFDR = 0.86	B = 0.046, p = 0.47, pFDR = 0.95
**Volume of grey matter in Angular Gyrus (right)**	β = -0.086, p = 0.38, pFDR = 0.86	B = 0.019, p = 0.78, pFDR = 0.95
**Volume of grey matter in Lateral Occipital Cortex, superior division (left)**	β = -0.15, p = 0.094, pFDR = 0.59	B = -0.016, p = 0.79, pFDR = 0.95
**Volume of grey matter in Lateral Occipital Cortex, superior division (right)**	β = -0.098, p = 0.28, pFDR = 0.75	B = -0.046, p = 0.44, pFDR = 0.95
**Volume of grey matter in Lateral Occipital Cortex, inferior division (left)**	β = -0.1, p = 0.26, pFDR = 0.75	B = -0.036, p = 0.55, pFDR = 0.95
**Volume of grey matter in Lateral Occipital Cortex, inferior division (right)**	β = -0.11, p = 0.22, pFDR = 0.75	B = -8.67e-03, p = 0.88, pFDR = 0.95
**Volume of grey matter in Intracalcarine Cortex (left)**	β = 0.12, p = 0.21, pFDR = 0.75	B = -0.03, p = 0.63, pFDR = 0.95
**Volume of grey matter in Intracalcarine Cortex (right)**	β = 0.12, p = 0.21, pFDR = 0.75	B = -0.02, p = 0.74, pFDR = 0.95
**Volume of grey matter in Frontal Medial Cortex (left)**	β = -0.026, p = 0.79, pFDR = 1	B = 4.99e-03, p = 0.94, pFDR = 0.95
**Volume of grey matter in Frontal Medial Cortex (right)**	β = -0.047, p = 0.62, pFDR = 1	B = 6.98e-03, p = 0.91, pFDR = 0.95
**Volume of grey matter in Juxtapositional Lobule Cortex (formerly Supplementary Motor Cortex) (left)**	β = 0.11, p = 0.26, pFDR = 0.75	B = -0.079, p = 0.21, pFDR = 0.78
**Volume of grey matter in Juxtapositional Lobule Cortex (formerly Supplementary Motor Cortex) (right)**	β = 0.08, p = 0.39, pFDR = 0.86	B = -0.071, p = 0.25, pFDR = 0.78
**Volume of grey matter in Subcallosal Cortex (left)**	β = -0.1, p = 0.2, pFDR = 0.75	B = 0.047, p = 0.37, pFDR = 0.92
**Volume of grey matter in Subcallosal Cortex (right)**	β = -0.018, p = 0.82, pFDR = 1	B = 0.013, p = 0.81, pFDR = 0.95
**Volume of grey matter in Paracingulate Gyrus (left)**	β = 0.16, p = 0.078, pFDR = 0.59	B = -0.07, p = 0.24, pFDR = 0.78
**Volume of grey matter in Paracingulate Gyrus (right)**	β = 0.18, p = 0.045, pFDR = 0.48	B = -0.095, p = 0.11, pFDR = 0.78
**Volume of grey matter in Cingulate Gyrus, anterior division (left)**	β = 0.028, p = 0.76, pFDR = 1	B = 0.074, p = 0.24, pFDR = 0.78
**Volume of grey matter in Cingulate Gyrus, anterior division (right)**	β = 0.1, p = 0.29, pFDR = 0.75	B = 0.02, p = 0.74, pFDR = 0.95
**Volume of grey matter in Cingulate Gyrus, posterior division (left)**	β = -0.021, p = 0.8, pFDR = 1	B = 0.013, p = 0.82, pFDR = 0.95
**Volume of grey matter in Cingulate Gyrus, posterior division (right)**	β = -0.1, p = 0.21, pFDR = 0.75	B = 0.043, p = 0.44, pFDR = 0.95
**Volume of grey matter in Precuneous Cortex (left)**	β = 5.44e-03, p = 0.95, pFDR = 1	B = -0.086, p = 0.14, pFDR = 0.78
**Volume of grey matter in Precuneous Cortex (right)**	β = -0.045, p = 0.61, pFDR = 1	B = -0.062, p = 0.28, pFDR = 0.82
**Volume of grey matter in Cuneal Cortex (left)**	β = 0.071, p = 0.44, pFDR = 0.88	B = -0.059, p = 0.32, pFDR = 0.89
**Volume of grey matter in Cuneal Cortex (right)**	β = 0.014, p = 0.88, pFDR = 1	B = -6.85e-03, p = 0.91, pFDR = 0.95
**Volume of grey matter in Frontal Orbital Cortex (left)**	β = -7.80e-03, p = 0.93, pFDR = 1	B = -9.84e-03, p = 0.86, pFDR = 0.95
**Volume of grey matter in Frontal Orbital Cortex (right)**	β = 0.036, p = 0.69, pFDR = 1	B = -0.016, p = 0.78, pFDR = 0.95
**Volume of grey matter in Parahippocampal Gyrus, anterior division (left)**	β = -0.14, p = 0.13, pFDR = 0.69	B = 0.016, p = 0.79, pFDR = 0.95
**Volume of grey matter in Parahippocampal Gyrus, anterior division (right)**	β = -0.15, p = 0.08, pFDR = 0.59	B = 0.096, p = 0.098, pFDR = 0.78
**Volume of grey matter in Parahippocampal Gyrus, posterior division (left)**	β = 0.025, p = 0.79, pFDR = 1	B = -0.071, p = 0.25, pFDR = 0.78
**Volume of grey matter in Parahippocampal Gyrus, posterior division (right)**	β = -0.072, p = 0.45, pFDR = 0.89	B = 0.049, p = 0.44, pFDR = 0.95
**Volume of grey matter in Lingual Gyrus (left)**	β = -2.55e-03, p = 0.98, pFDR = 1	B = 0.022, p = 0.7, pFDR = 0.95
**Volume of grey matter in Lingual Gyrus (right)**	β = 0.055, p = 0.52, pFDR = 0.96	B = -0.023, p = 0.68, pFDR = 0.95
**Volume of grey matter in Temporal Fusiform Cortex, anterior division (left)**	β = 0.094, p = 0.31, pFDR = 0.76	B = -0.073, p = 0.23, pFDR = 0.78
**Volume of grey matter in Temporal Fusiform Cortex, anterior division (right)**	β = -0.056, p = 0.56, pFDR = 0.97	B = 0.033, p = 0.61, pFDR = 0.95
**Volume of grey matter in Temporal Fusiform Cortex, posterior division (left)**	β = 1.21e-04, p = 1, pFDR = 1	B = -0.021, p = 0.73, pFDR = 0.95
**Volume of grey matter in Temporal Fusiform Cortex, posterior division (right)**	β = -0.088, p = 0.32, pFDR = 0.76	B = 0.058, p = 0.33, pFDR = 0.89
**Volume of grey matter in Temporal Occipital Fusiform Cortex (left)**	β = -0.064, p = 0.5, pFDR = 0.94	B = 0.027, p = 0.68, pFDR = 0.95
**Volume of grey matter in Temporal Occipital Fusiform Cortex (right)**	β = -0.11, p = 0.23, pFDR = 0.75	B = 0.019, p = 0.75, pFDR = 0.95
**Volume of grey matter in Occipital Fusiform Gyrus (left)**	β = 0.096, p = 0.32, pFDR = 0.76	B = -0.089, p = 0.16, pFDR = 0.78
**Volume of grey matter in Occipital Fusiform Gyrus (right)**	β = 0.014, p = 0.88, pFDR = 1	B = -0.08, p = 0.22, pFDR = 0.78
**Volume of grey matter in Frontal Operculum Cortex (left)**	β = 0.073, p = 0.43, pFDR = 0.88	B = -0.022, p = 0.71, pFDR = 0.95
**Volume of grey matter in Frontal Operculum Cortex (right)**	β = 0.044, p = 0.63, pFDR = 1	B = -0.033, p = 0.6, pFDR = 0.95
**Volume of grey matter in Central Opercular Cortex (left)**	β = -0.032, p = 0.71, pFDR = 1	B = 0.051, p = 0.38, pFDR = 0.92
**Volume of grey matter in Central Opercular Cortex (right)**	β = 0.1, p = 0.24, pFDR = 0.75	B = -0.088, p = 0.13, pFDR = 0.78
**Volume of grey matter in Parietal Operculum Cortex (left)**	β = 0.18, p = 0.056, pFDR = 0.54	B = -0.075, p = 0.23, pFDR = 0.78
**Volume of grey matter in Parietal Operculum Cortex (right)**	β = 0.03, p = 0.74, pFDR = 1	B = -0.026, p = 0.68, pFDR = 0.95
**Volume of grey matter in Planum Polare (left)**	β = 0.091, p = 0.32, pFDR = 0.76	B = -0.05, p = 0.41, pFDR = 0.94
**Volume of grey matter in Planum Polare (right)**	β = 0.12, p = 0.18, pFDR = 0.75	B = -0.085, p = 0.14, pFDR = 0.78
**Volume of grey matter in Heschl's Gyrus (includes H1 and H2) (left)**	β = 0.22, p = 0.015, pFDR = 0.29	B = -0.12, p = 0.047, pFDR = 0.78
**Volume of grey matter in Heschl's Gyrus (includes H1 and H2) (right)**	β = 0.099, p = 0.28, pFDR = 0.75	B = -0.078, p = 0.2, pFDR = 0.78
**Volume of grey matter in Planum Temporale (left)**	β = 0.15, p = 0.09, pFDR = 0.59	B = -0.091, p = 0.12, pFDR = 0.78
**Volume of grey matter in Planum Temporale (right)**	β = -5.13e-03, p = 0.95, pFDR = 1	B = -9.71e-03, p = 0.87, pFDR = 0.95
**Volume of grey matter in Supracalcarine Cortex (left)**	β = -0.043, p = 0.63, pFDR = 1	B = 0.045, p = 0.46, pFDR = 0.95
**Volume of grey matter in Supracalcarine Cortex (right)**	β = 0.011, p = 0.9, pFDR = 1	B = 0.042, p = 0.49, pFDR = 0.95
**Volume of grey matter in Occipital Pole (left)**	β = -0.065, p = 0.49, pFDR = 0.94	B = -0.025, p = 0.69, pFDR = 0.95
**Volume of grey matter in Occipital Pole (right)**	β = -0.055, p = 0.54, pFDR = 0.96	B = -0.01, p = 0.86, pFDR = 0.95
**Volume of grey matter in Thalamus (left)**	β = 6.72e-03, p = 0.94, pFDR = 1	B = 0.068, p = 0.27, pFDR = 0.82
**Volume of grey matter in Thalamus (right)**	β = 0.075, p = 0.42, pFDR = 0.88	B = -0.019, p = 0.75, pFDR = 0.95
**Volume of grey matter in Caudate (left)**	β = -0.12, p = 0.16, pFDR = 0.75	B = 0.11, p = 0.065, pFDR = 0.78
**Volume of grey matter in Caudate (right)**	β = -0.11, p = 0.23, pFDR = 0.75	B = 0.086, p = 0.13, pFDR = 0.78
**Volume of grey matter in Putamen (left)**	β = -0.16, p = 0.09, pFDR = 0.59	B = 0.06, p = 0.35, pFDR = 0.89
**Volume of grey matter in Putamen (right)**	β = -0.2, p = 0.035, pFDR = 0.48	B = 0.11, p = 0.084, pFDR = 0.78
**Volume of grey matter in Pallidum (left)**	β = -7.78e-04, p = 0.99, pFDR = 1	B = 0.078, p = 0.21, pFDR = 0.78
**Volume of grey matter in Pallidum (right)**	β = 0.015, p = 0.88, pFDR = 1	B = 0.097, p = 0.12, pFDR = 0.78
**Volume of grey matter in Hippocampus (left)**	β = -0.22, p = 9.47e-03, pFDR = 0.29	B = 2.53e-03, p = 0.96, pFDR = 0.96
**Volume of grey matter in Hippocampus (right)**	β = -0.22, p = 0.013, pFDR = 0.29	B = 0.019, p = 0.74, pFDR = 0.95
**Volume of grey matter in Amygdala (left)**	β = -0.24, p = 4.21e-03, pFDR = 0.18	B = 0.098, p = 0.081, pFDR = 0.78
**Volume of grey matter in Amygdala (right)**	β = -0.27, p = 1.00e-03, pFDR = 0.13	B = 0.13, p = 0.021, pFDR = 0.78
**Volume of grey matter in Ventral Striatum (left)**	β = 0.019, p = 0.83, pFDR = 1	B = -0.018, p = 0.76, pFDR = 0.95
**Volume of grey matter in Ventral Striatum (right)**	β = 0.019, p = 0.83, pFDR = 1	B = 0.012, p = 0.83, pFDR = 0.95

Table S7

description	effect of ptau181	effect of ptau181*CSVD burden
**Volume of thalamus (left)**	β = -0.052, p = 0.42, pFDR = 0.79	B = 0.011, p = 0.82, pFDR = 0.98
**Volume of thalamus (right)**	β = -0.077, p = 0.24, pFDR = 0.7	B = 8.40e-03, p = 0.86, pFDR = 0.98
**Volume of caudate (left)**	β = 0.046, p = 0.52, pFDR = 0.86	B = -0.061, p = 0.26, pFDR = 0.95
**Volume of caudate (right)**	β = 0.017, p = 0.82, pFDR = 0.93	B = -0.051, p = 0.35, pFDR = 0.95
**Volume of putamen (left)**	β = -0.15, p = 0.033, pFDR = 0.25	B = 0.042, p = 0.42, pFDR = 0.95
**Volume of putamen (right)**	β = -0.099, p = 0.16, pFDR = 0.59	B = -7.21e-03, p = 0.89, pFDR = 0.98
**Volume of pallidum (left)**	β = -0.1, p = 0.18, pFDR = 0.66	B = -0.021, p = 0.72, pFDR = 0.98
**Volume of pallidum (right)**	β = -0.19, p = 0.011, pFDR = 0.12	B = 0.049, p = 0.38, pFDR = 0.95
**Volume of hippocampus (left)**	β = -0.16, p = 0.02, pFDR = 0.17	B = -0.036, p = 0.47, pFDR = 0.95
**Volume of hippocampus (right)**	β = -0.23, p = 4.23e-04, pFDR = 0.053	B = 0.013, p = 0.79, pFDR = 0.98
**Volume of amygdala (left)**	β = 0.083, p = 0.29, pFDR = 0.71	B = -0.11, p = 0.06, pFDR = 0.95
**Volume of amygdala (right)**	β = -0.039, p = 0.62, pFDR = 0.88	B = -0.088, p = 0.13, pFDR = 0.95
**Volume of accumbens (left)**	β = -4.53e-03, p = 0.95, pFDR = 0.97	B = -0.11, p = 0.034, pFDR = 0.95
**Volume of accumbens (right)**	β = -0.2, p = 2.27e-03, pFDR = 0.07	B = 0.05, p = 0.31, pFDR = 0.95
**Volume of brain stem + 4th ventricle**	β = 2.76e-03, p = 0.97, pFDR = 0.98	B = -3.74e-03, p = 0.95, pFDR = 0.99

Table S8

cognition	term	effect	description
MMSE	beta1_0	0.213 [0.0157, 0.61]*	Mean FA in posterior corona radiata on FA skeleton (left)
MMSE	c_prime_0	-0.32 [-0.518, -0.127]*	Mean FA in posterior corona radiata on FA skeleton (left)
MMSE	total_0	-0.287 [-0.498, -0.1]*	Mean FA in posterior corona radiata on FA skeleton (left)
MMSE	beta1_1	0.179 [0.0191, 0.388]*	Mean FA in posterior corona radiata on FA skeleton (left)
MMSE	c_prime_1	-0.52 [-0.7, -0.245]*	Mean FA in posterior corona radiata on FA skeleton (left)
MMSE	total_1	-0.533 [-0.705, -0.268]*	Mean FA in posterior corona radiata on FA skeleton (left)
MMSE	beta1_2	0.206 [0.0214, 0.399]*	Mean FA in posterior corona radiata on FA skeleton (left)
MMSE	beta2_2	-0.197 [-0.441, -0.0271]*	Mean FA in posterior corona radiata on FA skeleton (left)
MMSE	total_2	-0.262 [-0.469, -0.0361]*	Mean FA in posterior corona radiata on FA skeleton (left)
MMSE	diff_beta2_2_0	-0.272 [-0.586, -0.0803]*	Mean FA in posterior corona radiata on FA skeleton (left)
MoCA	beta1_0	0.213 [0.0157, 0.61]*	Mean FA in posterior corona radiata on FA skeleton (left)
MoCA	beta3_0	0.126 [0.0171, 0.25]*	Mean FA in posterior corona radiata on FA skeleton (left)
MoCA	c_prime_0	-0.241 [-0.386, -0.0812]*	Mean FA in posterior corona radiata on FA skeleton (left)
MoCA	total_0	-0.22 [-0.363, -0.0657]*	Mean FA in posterior corona radiata on FA skeleton (left)
MoCA	beta1_1	0.179 [0.0191, 0.388]*	Mean FA in posterior corona radiata on FA skeleton (left)
MoCA	c_prime_1	-0.464 [-0.614, -0.213]*	Mean FA in posterior corona radiata on FA skeleton (left)
MoCA	total_1	-0.472 [-0.612, -0.227]*	Mean FA in posterior corona radiata on FA skeleton (left)
MoCA	beta1_2	0.206 [0.0214, 0.399]*	Mean FA in posterior corona radiata on FA skeleton (left)
MoCA	beta2_2	-0.197 [-0.441, -0.0271]*	Mean FA in posterior corona radiata on FA skeleton (left)
MoCA	c_prime_2	-0.331 [-0.479, -0.13]*	Mean FA in posterior corona radiata on FA skeleton (left)
MoCA	total_2	-0.361 [-0.526, -0.184]*	Mean FA in posterior corona radiata on FA skeleton (left)
MoCA	diff_beta2_2_0	-0.272 [-0.586, -0.0803]*	Mean FA in posterior corona radiata on FA skeleton (left)
MoCA	diff_ind1_2_0	-7.304e-03 [-0.0349, -3.797e-04]*	Mean FA in posterior corona radiata on FA skeleton (left)
EM	beta1_0	0.213 [0.0157, 0.61]*	Mean FA in posterior corona radiata on FA skeleton (left)
EM	c_prime_0	-0.241 [-0.36, -0.143]*	Mean FA in posterior corona radiata on FA skeleton (left)
EM	total_0	-0.25 [-0.352, -0.146]*	Mean FA in posterior corona radiata on FA skeleton (left)
EM	beta1_1	0.179 [0.0191, 0.388]*	Mean FA in posterior corona radiata on FA skeleton (left)
EM	c_prime_1	-0.183 [-0.326, -0.0334]*	Mean FA in posterior corona radiata on FA skeleton (left)
EM	total_1	-0.204 [-0.332, -0.0676]*	Mean FA in posterior corona radiata on FA skeleton (left)
EM	beta1_2	0.206 [0.0214, 0.399]*	Mean FA in posterior corona radiata on FA skeleton (left)
EM	beta2_2	-0.197 [-0.441, -0.0271]*	Mean FA in posterior corona radiata on FA skeleton (left)
EM	diff_beta2_2_0	-0.272 [-0.586, -0.0803]*	Mean FA in posterior corona radiata on FA skeleton (left)
ISP	beta1_0	0.213 [0.0157, 0.61]*	Mean FA in posterior corona radiata on FA skeleton (left)
ISP	c_prime_0	-0.185 [-0.318, -0.0108]*	Mean FA in posterior corona radiata on FA skeleton (left)
ISP	total_0	-0.169 [-0.307, -7.943e-03]*	Mean FA in posterior corona radiata on FA skeleton (left)
ISP	beta1_1	0.179 [0.0191, 0.388]*	Mean FA in posterior corona radiata on FA skeleton (left)
ISP	c_prime_1	-0.411 [-0.524, -0.259]*	Mean FA in posterior corona radiata on FA skeleton (left)
ISP	total_1	-0.399 [-0.514, -0.25]*	Mean FA in posterior corona radiata on FA skeleton (left)
ISP	beta1_2	0.206 [0.0214, 0.399]*	Mean FA in posterior corona radiata on FA skeleton (left)
ISP	beta2_2	-0.197 [-0.441, -0.0271]*	Mean FA in posterior corona radiata on FA skeleton (left)
ISP	c_prime_2	-0.285 [-0.418, -0.119]*	Mean FA in posterior corona radiata on FA skeleton (left)
ISP	total_2	-0.267 [-0.389, -0.118]*	Mean FA in posterior corona radiata on FA skeleton (left)
ISP	diff_beta2_2_0	-0.272 [-0.586, -0.0803]*	Mean FA in posterior corona radiata on FA skeleton (left)
ISP	diff_c_prime_1_0	-0.226 [-0.431, -0.0257]*	Mean FA in posterior corona radiata on FA skeleton (left)
ISP	diff_total_1_0	-0.23 [-0.426, -0.0261]*	Mean FA in posterior corona radiata on FA skeleton (left)
LF	beta1_0	0.213 [0.0157, 0.61]*	Mean FA in posterior corona radiata on FA skeleton (left)
LF	beta3_0	0.115 [0.0117, 0.227]*	Mean FA in posterior corona radiata on FA skeleton (left)
LF	c_prime_0	-0.238 [-0.376, -0.0849]*	Mean FA in posterior corona radiata on FA skeleton (left)
LF	total_0	-0.233 [-0.359, -0.0909]*	Mean FA in posterior corona radiata on FA skeleton (left)
LF	beta1_1	0.179 [0.0191, 0.388]*	Mean FA in posterior corona radiata on FA skeleton (left)
LF	c_prime_1	-0.242 [-0.376, -0.105]*	Mean FA in posterior corona radiata on FA skeleton (left)
LF	total_1	-0.23 [-0.369, -0.1]*	Mean FA in posterior corona radiata on FA skeleton (left)
LF	beta1_2	0.206 [0.0214, 0.399]*	Mean FA in posterior corona radiata on FA skeleton (left)
LF	beta2_2	-0.197 [-0.441, -0.0271]*	Mean FA in posterior corona radiata on FA skeleton (left)
LF	c_prime_2	-0.306 [-0.451, -0.127]*	Mean FA in posterior corona radiata on FA skeleton (left)
LF	total_2	-0.294 [-0.436, -0.133]*	Mean FA in posterior corona radiata on FA skeleton (left)
LF	diff_beta2_2_0	-0.272 [-0.586, -0.0803]*	Mean FA in posterior corona radiata on FA skeleton (left)
EF	beta1_0	0.213 [0.0157, 0.61]*	Mean FA in posterior corona radiata on FA skeleton (left)
EF	beta3_0	0.161 [0.0342, 0.418]*	Mean FA in posterior corona radiata on FA skeleton (left)
EF	beta1_1	0.179 [0.0191, 0.388]*	Mean FA in posterior corona radiata on FA skeleton (left)
EF	c_prime_1	-0.354 [-0.486, -0.196]*	Mean FA in posterior corona radiata on FA skeleton (left)
EF	total_1	-0.36 [-0.494, -0.207]*	Mean FA in posterior corona radiata on FA skeleton (left)
EF	beta1_2	0.206 [0.0214, 0.399]*	Mean FA in posterior corona radiata on FA skeleton (left)
EF	beta2_2	-0.197 [-0.441, -0.0271]*	Mean FA in posterior corona radiata on FA skeleton (left)
EF	c_prime_2	-0.241 [-0.391, -0.0141]*	Mean FA in posterior corona radiata on FA skeleton (left)
EF	total_2	-0.252 [-0.395, -0.0436]*	Mean FA in posterior corona radiata on FA skeleton (left)
EF	diff_beta2_2_0	-0.272 [-0.586, -0.0803]*	Mean FA in posterior corona radiata on FA skeleton (left)
EF	diff_ind1_2_0	-6.967e-03 [-0.032, -3.392e-05]*	Mean FA in posterior corona radiata on FA skeleton (left)
EF	diff_c_prime_1_0	-0.29 [-0.592, -0.0657]*	Mean FA in posterior corona radiata on FA skeleton (left)
EF	diff_total_1_0	-0.31 [-0.606, -0.0808]*	Mean FA in posterior corona radiata on FA skeleton (left)
VS	beta1_0	0.213 [0.0157, 0.61]*	Mean FA in posterior corona radiata on FA skeleton (left)
VS	beta3_0	0.107 [1.859e-03, 0.239]*	Mean FA in posterior corona radiata on FA skeleton (left)
VS	c_prime_0	-0.249 [-0.429, -0.0711]*	Mean FA in posterior corona radiata on FA skeleton (left)
VS	total_0	-0.223 [-0.396, -0.0501]*	Mean FA in posterior corona radiata on FA skeleton (left)
VS	beta1_1	0.179 [0.0191, 0.388]*	Mean FA in posterior corona radiata on FA skeleton (left)
VS	c_prime_1	-0.202 [-0.381, -0.0122]*	Mean FA in posterior corona radiata on FA skeleton (left)
VS	beta1_2	0.206 [0.0214, 0.399]*	Mean FA in posterior corona radiata on FA skeleton (left)
VS	beta2_2	-0.197 [-0.441, -0.0271]*	Mean FA in posterior corona radiata on FA skeleton (left)
VS	c_prime_2	-0.299 [-0.475, -0.0958]*	Mean FA in posterior corona radiata on FA skeleton (left)
VS	total_2	-0.292 [-0.475, -0.112]*	Mean FA in posterior corona radiata on FA skeleton (left)
VS	diff_beta2_2_0	-0.272 [-0.586, -0.0803]*	Mean FA in posterior corona radiata on FA skeleton (left)
MMSE	beta1_0	0.213 [0.0157, 0.61]*	Mean MD in retrolenticular part of internal capsule on FA skeleton (right)

MMSE	beta3_0	-0.258 [-0.493, -0.0673]*	Mean MD in retrolenticular part of internal capsule on FA skeleton (right)
MMSE	c_prime_0	-0.316 [-0.505, -0.138]*	Mean MD in retrolenticular part of internal capsule on FA skeleton (right)
MMSE	total_0	-0.287 [-0.498, -0.1]*	Mean MD in retrolenticular part of internal capsule on FA skeleton (right)
MMSE	beta1_1	0.179 [0.0191, 0.388]*	Mean MD in retrolenticular part of internal capsule on FA skeleton (right)
MMSE	beta4_1	0.208 [0.0472, 0.373]*	Mean MD in retrolenticular part of internal capsule on FA skeleton (right)
MMSE	c_prime_1	-0.505 [-0.685, -0.249]*	Mean MD in retrolenticular part of internal capsule on FA skeleton (right)
MMSE	total_1	-0.533 [-0.705, -0.268]*	Mean MD in retrolenticular part of internal capsule on FA skeleton (right)
MMSE	beta1_2	0.206 [0.0214, 0.399]*	Mean MD in retrolenticular part of internal capsule on FA skeleton (right)
MMSE	beta2_2	0.336 [0.181, 0.532]*	Mean MD in retrolenticular part of internal capsule on FA skeleton (right)
MMSE	beta4_2	0.196 [0.0109, 0.325]*	Mean MD in retrolenticular part of internal capsule on FA skeleton (right)
MMSE	total_2	-0.262 [-0.469, -0.0361]*	Mean MD in retrolenticular part of internal capsule on FA skeleton (right)
MMSE	diff_beta2_2_0	0.275 [0.0721, 0.649]*	Mean MD in retrolenticular part of internal capsule on FA skeleton (right)
MMSE	diff_beta2_2_1	0.27 [0.0101, 0.524]*	Mean MD in retrolenticular part of internal capsule on FA skeleton (right)
MoCA	beta1_0	0.213 [0.0157, 0.61]*	Mean MD in retrolenticular part of internal capsule on FA skeleton (right)
MoCA	beta3_0	-0.211 [-0.348, -0.0563]*	Mean MD in retrolenticular part of internal capsule on FA skeleton (right)
MoCA	c_prime_0	-0.236 [-0.376, -0.0753]*	Mean MD in retrolenticular part of internal capsule on FA skeleton (right)
MoCA	total_0	-0.22 [-0.363, -0.0657]*	Mean MD in retrolenticular part of internal capsule on FA skeleton (right)
MoCA	beta1_1	0.179 [0.0191, 0.388]*	Mean MD in retrolenticular part of internal capsule on FA skeleton (right)
MoCA	beta4_1	0.208 [0.0472, 0.373]*	Mean MD in retrolenticular part of internal capsule on FA skeleton (right)
MoCA	c_prime_1	-0.432 [-0.578, -0.194]*	Mean MD in retrolenticular part of internal capsule on FA skeleton (right)
MoCA	ind_1	-0.0399 [-0.11, -8.852e-04]*	Mean MD in retrolenticular part of internal capsule on FA skeleton (right)
MoCA	total_1	-0.472 [-0.612, -0.227]*	Mean MD in retrolenticular part of internal capsule on FA skeleton (right)
MoCA	beta1_2	0.206 [0.0214, 0.399]*	Mean MD in retrolenticular part of internal capsule on FA skeleton (right)
MoCA	beta2_2	0.336 [0.181, 0.532]*	Mean MD in retrolenticular part of internal capsule on FA skeleton (right)
MoCA	beta4_2	0.196 [0.0109, 0.325]*	Mean MD in retrolenticular part of internal capsule on FA skeleton (right)
MoCA	c_prime_2	-0.346 [-0.506, -0.146]*	Mean MD in retrolenticular part of internal capsule on FA skeleton (right)
MoCA	total_2	-0.361 [-0.526, -0.184]*	Mean MD in retrolenticular part of internal capsule on FA skeleton (right)
MoCA	diff_beta2_2_0	0.275 [0.0721, 0.649]*	Mean MD in retrolenticular part of internal capsule on FA skeleton (right)
MoCA	diff_beta2_2_1	0.27 [0.0101, 0.524]*	Mean MD in retrolenticular part of internal capsule on FA skeleton (right)
EM	beta1_0	0.213 [0.0157, 0.61]*	Mean MD in retrolenticular part of internal capsule on FA skeleton (right)
EM	c_prime_0	-0.238 [-0.367, -0.145]*	Mean MD in retrolenticular part of internal capsule on FA skeleton (right)
EM	total_0	-0.25 [-0.352, -0.146]*	Mean MD in retrolenticular part of internal capsule on FA skeleton (right)
EM	beta1_1	0.179 [0.0191, 0.388]*	Mean MD in retrolenticular part of internal capsule on FA skeleton (right)
EM	beta4_1	0.208 [0.0472, 0.373]*	Mean MD in retrolenticular part of internal capsule on FA skeleton (right)
EM	beta3_1	-0.151 [-0.289, -0.0102]*	Mean MD in retrolenticular part of internal capsule on FA skeleton (right)
EM	ind3_1	-0.0304 [-0.0824, -3.106e-03]*	Mean MD in retrolenticular part of internal capsule on FA skeleton (right)
EM	ind_1	-0.0499 [-0.135, -5.809e-03]*	Mean MD in retrolenticular part of internal capsule on FA skeleton (right)
EM	total_1	-0.204 [-0.332, -0.0676]*	Mean MD in retrolenticular part of internal capsule on FA skeleton (right)
EM	beta1_2	0.206 [0.0214, 0.399]*	Mean MD in retrolenticular part of internal capsule on FA skeleton (right)
EM	beta2_2	0.336 [0.181, 0.532]*	Mean MD in retrolenticular part of internal capsule on FA skeleton (right)
EM	beta4_2	0.196 [0.0109, 0.325]*	Mean MD in retrolenticular part of internal capsule on FA skeleton (right)
EM	diff_beta2_2_0	0.275 [0.0721, 0.649]*	Mean MD in retrolenticular part of internal capsule on FA skeleton (right)
EM	diff_beta2_2_1	0.27 [0.0101, 0.524]*	Mean MD in retrolenticular part of internal capsule on FA skeleton (right)
EM	diff_beta3_2_1	0.236 [7.189e-03, 0.466]*	Mean MD in retrolenticular part of internal capsule on FA skeleton (right)
ISP	beta1_0	0.213 [0.0157, 0.61]*	Mean MD in retrolenticular part of internal capsule on FA skeleton (right)
ISP	c_prime_0	-0.182 [-0.306, -6.469e-03]*	Mean MD in retrolenticular part of internal capsule on FA skeleton (right)
ISP	total_0	-0.169 [-0.307, -7.943e-03]*	Mean MD in retrolenticular part of internal capsule on FA skeleton (right)
ISP	beta1_1	0.179 [0.0191, 0.388]*	Mean MD in retrolenticular part of internal capsule on FA skeleton (right)
ISP	beta4_1	0.208 [0.0472, 0.373]*	Mean MD in retrolenticular part of internal capsule on FA skeleton (right)
ISP	c_prime_1	-0.4 [-0.519, -0.254]*	Mean MD in retrolenticular part of internal capsule on FA skeleton (right)
ISP	total_1	-0.399 [-0.514, -0.25]*	Mean MD in retrolenticular part of internal capsule on FA skeleton (right)
ISP	beta1_2	0.206 [0.0214, 0.399]*	Mean MD in retrolenticular part of internal capsule on FA skeleton (right)
ISP	beta2_2	0.336 [0.181, 0.532]*	Mean MD in retrolenticular part of internal capsule on FA skeleton (right)
ISP	beta4_2	0.196 [0.0109, 0.325]*	Mean MD in retrolenticular part of internal capsule on FA skeleton (right)
ISP	c_prime_2	-0.295 [-0.434, -0.12]*	Mean MD in retrolenticular part of internal capsule on FA skeleton (right)
ISP	total_2	-0.267 [-0.389, -0.118]*	Mean MD in retrolenticular part of internal capsule on FA skeleton (right)
ISP	diff_beta2_2_0	0.275 [0.0721, 0.649]*	Mean MD in retrolenticular part of internal capsule on FA skeleton (right)
ISP	diff_beta2_2_1	0.27 [0.0101, 0.524]*	Mean MD in retrolenticular part of internal capsule on FA skeleton (right)
ISP	diff_c_prime_1_0	-0.217 [-0.43, -0.0314]*	Mean MD in retrolenticular part of internal capsule on FA skeleton (right)
ISP	diff_total_1_0	-0.23 [-0.426, -0.0261]*	Mean MD in retrolenticular part of internal capsule on FA skeleton (right)
LF	beta1_0	0.213 [0.0157, 0.61]*	Mean MD in retrolenticular part of internal capsule on FA skeleton (right)
LF	c_prime_0	-0.236 [-0.369, -0.0802]*	Mean MD in retrolenticular part of internal capsule on FA skeleton (right)
LF	total_0	-0.233 [-0.359, -0.0909]*	Mean MD in retrolenticular part of internal capsule on FA skeleton (right)
LF	beta1_1	0.179 [0.0191, 0.388]*	Mean MD in retrolenticular part of internal capsule on FA skeleton (right)
LF	beta4_1	0.208 [0.0472, 0.373]*	Mean MD in retrolenticular part of internal capsule on FA skeleton (right)
LF	c_prime_1	-0.202 [-0.34, -0.0767]*	Mean MD in retrolenticular part of internal capsule on FA skeleton (right)
LF	ind3_1	-0.04 [-0.089, -5.488e-04]*	Mean MD in retrolenticular part of internal capsule on FA skeleton (right)
LF	total_1	-0.23 [-0.369, -0.1]*	Mean MD in retrolenticular part of internal capsule on FA skeleton (right)
LF	beta1_2	0.206 [0.0214, 0.399]*	Mean MD in retrolenticular part of internal capsule on FA skeleton (right)
LF	beta2_2	0.336 [0.181, 0.532]*	Mean MD in retrolenticular part of internal capsule on FA skeleton (right)
LF	beta4_2	0.196 [0.0109, 0.325]*	Mean MD in retrolenticular part of internal capsule on FA skeleton (right)
LF	c_prime_2	-0.295 [-0.441, -0.108]*	Mean MD in retrolenticular part of internal capsule on FA skeleton (right)
LF	total_2	-0.294 [-0.436, -0.133]*	Mean MD in retrolenticular part of internal capsule on FA skeleton (right)
LF	diff_beta2_2_0	0.275 [0.0721, 0.649]*	Mean MD in retrolenticular part of internal capsule on FA skeleton (right)
LF	diff_beta2_2_1	0.27 [0.0101, 0.524]*	Mean MD in retrolenticular part of internal capsule on FA skeleton (right)
EF	beta1_0	0.213 [0.0157, 0.61]*	Mean MD in retrolenticular part of internal capsule on FA skeleton (right)
EF	beta1_1	0.179 [0.0191, 0.388]*	Mean MD in retrolenticular part of internal capsule on FA skeleton (right)
EF	beta4_1	0.208 [0.0472, 0.373]*	Mean MD in retrolenticular part of internal capsule on FA skeleton (right)
EF	c_prime_1	-0.346 [-0.477, -0.194]*	Mean MD in retrolenticular part of internal capsule on FA skeleton (right)
EF	total_1	-0.36 [-0.494, -0.207]*	Mean MD in retrolenticular part of internal capsule on FA skeleton (right)
EF	beta1_2	0.206 [0.0214, 0.399]*	Mean MD in retrolenticular part of internal capsule on FA skeleton (right)
EF	beta2_2	0.336 [0.181, 0.532]*	Mean MD in retrolenticular part of internal capsule on FA skeleton (right)
EF	beta4_2	0.196 [0.0109, 0.325]*	Mean MD in retrolenticular part of internal capsule on FA skeleton (right)
EF	c_prime_2	-0.259 [-0.414, -0.0211]*	Mean MD in retrolenticular part of internal capsule on FA skeleton (right)

EF	total_2	-0.252 [-0.395, -0.0436]*	Mean MD in retrolenticular part of internal capsule on FA skeleton (right)
EF	diff_beta2_2_0	0.275 [0.0721, 0.649]*	Mean MD in retrolenticular part of internal capsule on FA skeleton (right)
EF	diff_beta2_2_1	0.27 [0.0101, 0.524]*	Mean MD in retrolenticular part of internal capsule on FA skeleton (right)
EF	diff_c_prime_1_0	-0.288 [-0.608, -0.0711]*	Mean MD in retrolenticular part of internal capsule on FA skeleton (right)
EF	diff_total_1_0	-0.31 [-0.606, -0.0808]*	Mean MD in retrolenticular part of internal capsule on FA skeleton (right)
VS	beta1_0	0.213 [0.0157, 0.61]*	Mean MD in retrolenticular part of internal capsule on FA skeleton (right)
VS	c_prime_0	-0.245 [-0.423, -0.063]*	Mean MD in retrolenticular part of internal capsule on FA skeleton (right)
VS	total_0	-0.223 [-0.396, -0.0501]*	Mean MD in retrolenticular part of internal capsule on FA skeleton (right)
VS	beta1_1	0.179 [0.0191, 0.388]*	Mean MD in retrolenticular part of internal capsule on FA skeleton (right)
VS	beta4_1	0.208 [0.0472, 0.373]*	Mean MD in retrolenticular part of internal capsule on FA skeleton (right)
VS	beta3_1	-0.181 [-0.346, -0.0126]*	Mean MD in retrolenticular part of internal capsule on FA skeleton (right)
VS	ind3_1	-0.0379 [-0.106, -3.567e-03]*	Mean MD in retrolenticular part of internal capsule on FA skeleton (right)
VS	beta1_2	0.206 [0.0214, 0.399]*	Mean MD in retrolenticular part of internal capsule on FA skeleton (right)
VS	beta2_2	0.336 [0.181, 0.532]*	Mean MD in retrolenticular part of internal capsule on FA skeleton (right)
VS	beta4_2	0.196 [0.0109, 0.325]*	Mean MD in retrolenticular part of internal capsule on FA skeleton (right)
VS	c_prime_2	-0.302 [-0.484, -0.106]*	Mean MD in retrolenticular part of internal capsule on FA skeleton (right)
VS	total_2	-0.292 [-0.475, -0.112]*	Mean MD in retrolenticular part of internal capsule on FA skeleton (right)
VS	diff_beta2_2_0	0.275 [0.0721, 0.649]*	Mean MD in retrolenticular part of internal capsule on FA skeleton (right)
VS	diff_beta2_2_1	0.27 [0.0101, 0.524]*	Mean MD in retrolenticular part of internal capsule on FA skeleton (right)
VS	diff_beta3_2_1	0.218 [3.366e-03, 0.454]*	Mean MD in retrolenticular part of internal capsule on FA skeleton (right)
MMSE	beta1_0	0.213 [0.0157, 0.61]*	Mean MD in retrolenticular part of internal capsule on FA skeleton (left)
MMSE	beta3_0	-0.172 [-0.369, -8.501e-03]*	Mean MD in retrolenticular part of internal capsule on FA skeleton (left)
MMSE	c_prime_0	-0.321 [-0.524, -0.139]*	Mean MD in retrolenticular part of internal capsule on FA skeleton (left)
MMSE	total_0	-0.287 [-0.498, -0.1]*	Mean MD in retrolenticular part of internal capsule on FA skeleton (left)
MMSE	beta1_1	0.179 [0.0191, 0.388]*	Mean MD in retrolenticular part of internal capsule on FA skeleton (left)
MMSE	c_prime_1	-0.502 [-0.679, -0.238]*	Mean MD in retrolenticular part of internal capsule on FA skeleton (left)
MMSE	ind_1	-0.0311 [-0.121, -4.083e-04]*	Mean MD in retrolenticular part of internal capsule on FA skeleton (left)
MMSE	total_1	-0.533 [-0.705, -0.268]*	Mean MD in retrolenticular part of internal capsule on FA skeleton (left)
MMSE	beta1_2	0.206 [0.0214, 0.399]*	Mean MD in retrolenticular part of internal capsule on FA skeleton (left)
MMSE	beta3_2	-0.191 [-0.434, -0.0337]*	Mean MD in retrolenticular part of internal capsule on FA skeleton (left)
MMSE	ind1_2	-8.389e-03 [-0.0486, -5.600e-04]*	Mean MD in retrolenticular part of internal capsule on FA skeleton (left)
MMSE	ind3_2	-0.0216 [-0.0741, -1.432e-03]*	Mean MD in retrolenticular part of internal capsule on FA skeleton (left)
MMSE	ind_2	-0.0387 [-0.138, -1.731e-03]*	Mean MD in retrolenticular part of internal capsule on FA skeleton (left)
MMSE	total_2	-0.262 [-0.469, -0.0361]*	Mean MD in retrolenticular part of internal capsule on FA skeleton (left)
MMSE	diff_ind_1_0	-0.0649 [-0.19, -1.219e-03]*	Mean MD in retrolenticular part of internal capsule on FA skeleton (left)
MMSE	diff_ind_2_0	-0.0725 [-0.227, -8.518e-03]*	Mean MD in retrolenticular part of internal capsule on FA skeleton (left)
MoCA	beta1_0	0.213 [0.0157, 0.61]*	Mean MD in retrolenticular part of internal capsule on FA skeleton (left)
MoCA	beta3_0	-0.223 [-0.351, -0.0926]*	Mean MD in retrolenticular part of internal capsule on FA skeleton (left)
MoCA	c_prime_0	-0.243 [-0.387, -0.0874]*	Mean MD in retrolenticular part of internal capsule on FA skeleton (left)
MoCA	total_0	-0.22 [-0.363, -0.0657]*	Mean MD in retrolenticular part of internal capsule on FA skeleton (left)
MoCA	beta1_1	0.179 [0.0191, 0.388]*	Mean MD in retrolenticular part of internal capsule on FA skeleton (left)
MoCA	c_prime_1	-0.449 [-0.596, -0.208]*	Mean MD in retrolenticular part of internal capsule on FA skeleton (left)
MoCA	total_1	-0.472 [-0.612, -0.227]*	Mean MD in retrolenticular part of internal capsule on FA skeleton (left)
MoCA	beta1_2	0.206 [0.0214, 0.399]*	Mean MD in retrolenticular part of internal capsule on FA skeleton (left)
MoCA	beta3_2	-0.156 [-0.334, -7.051e-03]*	Mean MD in retrolenticular part of internal capsule on FA skeleton (left)
MoCA	c_prime_2	-0.33 [-0.489, -0.137]*	Mean MD in retrolenticular part of internal capsule on FA skeleton (left)
MoCA	ind1_2	-6.949e-03 [-0.0345, -1.326e-04]*	Mean MD in retrolenticular part of internal capsule on FA skeleton (left)
MoCA	ind3_2	-0.0169 [-0.0525, -5.930e-04]*	Mean MD in retrolenticular part of internal capsule on FA skeleton (left)
MoCA	ind_2	-0.0304 [-0.112, -1.811e-04]*	Mean MD in retrolenticular part of internal capsule on FA skeleton (left)
MoCA	total_2	-0.361 [-0.526, -0.184]*	Mean MD in retrolenticular part of internal capsule on FA skeleton (left)
MoCA	diff_ind_2_0	-0.0535 [-0.158, -3.070e-03]*	Mean MD in retrolenticular part of internal capsule on FA skeleton (left)
EM	beta1_0	0.213 [0.0157, 0.61]*	Mean MD in retrolenticular part of internal capsule on FA skeleton (left)
EM	c_prime_0	-0.241 [-0.371, -0.152]*	Mean MD in retrolenticular part of internal capsule on FA skeleton (left)
EM	total_0	-0.25 [-0.352, -0.146]*	Mean MD in retrolenticular part of internal capsule on FA skeleton (left)
EM	beta1_1	0.179 [0.0191, 0.388]*	Mean MD in retrolenticular part of internal capsule on FA skeleton (left)
EM	beta3_1	-0.181 [-0.332, -0.0249]*	Mean MD in retrolenticular part of internal capsule on FA skeleton (left)
EM	ind_1	-0.0481 [-0.136, -4.012e-03]*	Mean MD in retrolenticular part of internal capsule on FA skeleton (left)
EM	total_1	-0.204 [-0.332, -0.0676]*	Mean MD in retrolenticular part of internal capsule on FA skeleton (left)
EM	beta1_2	0.206 [0.0214, 0.399]*	Mean MD in retrolenticular part of internal capsule on FA skeleton (left)
ISP	beta1_0	0.213 [0.0157, 0.61]*	Mean MD in retrolenticular part of internal capsule on FA skeleton (left)
ISP	c_prime_0	-0.185 [-0.312, -9.413e-03]*	Mean MD in retrolenticular part of internal capsule on FA skeleton (left)
ISP	total_0	-0.169 [-0.307, -7.943e-03]*	Mean MD in retrolenticular part of internal capsule on FA skeleton (left)
ISP	beta1_1	0.179 [0.0191, 0.388]*	Mean MD in retrolenticular part of internal capsule on FA skeleton (left)
ISP	c_prime_1	-0.406 [-0.519, -0.254]*	Mean MD in retrolenticular part of internal capsule on FA skeleton (left)
ISP	total_1	-0.399 [-0.514, -0.25]*	Mean MD in retrolenticular part of internal capsule on FA skeleton (left)
ISP	beta1_2	0.206 [0.0214, 0.399]*	Mean MD in retrolenticular part of internal capsule on FA skeleton (left)
ISP	c_prime_2	-0.273 [-0.4, -0.113]*	Mean MD in retrolenticular part of internal capsule on FA skeleton (left)
ISP	ind1_2	-4.980e-03 [-0.0393, -1.205e-03]*	Mean MD in retrolenticular part of internal capsule on FA skeleton (left)
ISP	total_2	-0.267 [-0.389, -0.118]*	Mean MD in retrolenticular part of internal capsule on FA skeleton (left)
ISP	diff_ind1_2_0	-4.674e-03 [-0.0356, -6.851e-05]*	Mean MD in retrolenticular part of internal capsule on FA skeleton (left)
ISP	diff_c_prime_1_0	-0.221 [-0.428, -0.0295]*	Mean MD in retrolenticular part of internal capsule on FA skeleton (left)
ISP	diff_total_1_0	-0.23 [-0.426, -0.0261]*	Mean MD in retrolenticular part of internal capsule on FA skeleton (left)
LF	beta1_0	0.213 [0.0157, 0.61]*	Mean MD in retrolenticular part of internal capsule on FA skeleton (left)
LF	c_prime_0	-0.238 [-0.373, -0.0836]*	Mean MD in retrolenticular part of internal capsule on FA skeleton (left)
LF	total_0	-0.233 [-0.359, -0.0909]*	Mean MD in retrolenticular part of internal capsule on FA skeleton (left)
LF	beta1_1	0.179 [0.0191, 0.388]*	Mean MD in retrolenticular part of internal capsule on FA skeleton (left)
LF	c_prime_1	-0.242 [-0.382, -0.109]*	Mean MD in retrolenticular part of internal capsule on FA skeleton (left)
LF	total_1	-0.23 [-0.369, -0.1]*	Mean MD in retrolenticular part of internal capsule on FA skeleton (left)
LF	beta1_2	0.206 [0.0214, 0.399]*	Mean MD in retrolenticular part of internal capsule on FA skeleton (left)
LF	c_prime_2	-0.301 [-0.448, -0.124]*	Mean MD in retrolenticular part of internal capsule on FA skeleton (left)
LF	total_2	-0.294 [-0.436, -0.133]*	Mean MD in retrolenticular part of internal capsule on FA skeleton (left)
EF	beta1_0	0.213 [0.0157, 0.61]*	Mean MD in retrolenticular part of internal capsule on FA skeleton (left)
EF	beta3_0	-0.139 [-0.324, -3.288e-03]*	Mean MD in retrolenticular part of internal capsule on FA skeleton (left)
EF	beta1_1	0.179 [0.0191, 0.388]*	Mean MD in retrolenticular part of internal capsule on FA skeleton (left)

EF	c_prime_1	-0.35 [-0.472, -0.196]*	Mean MD in retrolenticular part of internal capsule on FA skeleton (left)
EF	total_1	-0.36 [-0.494, -0.207]*	Mean MD in retrolenticular part of internal capsule on FA skeleton (left)
EF	beta1_2	0.206 [0.0214, 0.399]*	Mean MD in retrolenticular part of internal capsule on FA skeleton (left)
EF	beta3_2	-0.182 [-0.348, -0.0352]*	Mean MD in retrolenticular part of internal capsule on FA skeleton (left)
EF	c_prime_2	-0.231 [-0.382, -0.0113]*	Mean MD in retrolenticular part of internal capsule on FA skeleton (left)
EF	ind1_2	-6.768e-03 [-0.0411, -1.941e-03]*	Mean MD in retrolenticular part of internal capsule on FA skeleton (left)
EF	ind3_2	-0.0223 [-0.0722, -5.806e-04]*	Mean MD in retrolenticular part of internal capsule on FA skeleton (left)
EF	total_2	-0.252 [-0.395, -0.0436]*	Mean MD in retrolenticular part of internal capsule on FA skeleton (left)
EF	diff_ind1_2_0	-5.977e-03 [-0.0371, -2.230e-04]*	Mean MD in retrolenticular part of internal capsule on FA skeleton (left)
EF	diff_c_prime_1_0	-0.288 [-0.588, -0.0635]*	Mean MD in retrolenticular part of internal capsule on FA skeleton (left)
EF	diff_total_1_0	-0.31 [-0.606, -0.0808]*	Mean MD in retrolenticular part of internal capsule on FA skeleton (left)
VS	beta1_0	0.213 [0.0157, 0.61]*	Mean MD in retrolenticular part of internal capsule on FA skeleton (left)
VS	c_prime_0	-0.248 [-0.429, -0.0698]*	Mean MD in retrolenticular part of internal capsule on FA skeleton (left)
VS	total_0	-0.223 [-0.396, -0.0501]*	Mean MD in retrolenticular part of internal capsule on FA skeleton (left)
VS	beta1_1	0.179 [0.0191, 0.388]*	Mean MD in retrolenticular part of internal capsule on FA skeleton (left)
VS	c_prime_1	-0.188 [-0.371, -8.930e-03]*	Mean MD in retrolenticular part of internal capsule on FA skeleton (left)
VS	beta1_2	0.206 [0.0214, 0.399]*	Mean MD in retrolenticular part of internal capsule on FA skeleton (left)
VS	c_prime_2	-0.292 [-0.471, -0.0878]*	Mean MD in retrolenticular part of internal capsule on FA skeleton (left)
VS	total_2	-0.292 [-0.475, -0.112]*	Mean MD in retrolenticular part of internal capsule on FA skeleton (left)
MMSE	beta1_0	0.213 [0.0157, 0.61]*	Mean MD in superior corona radiata on FA skeleton (right)
MMSE	c_prime_0	-0.328 [-0.527, -0.133]*	Mean MD in superior corona radiata on FA skeleton (right)
MMSE	total_0	-0.287 [-0.498, -0.1]*	Mean MD in superior corona radiata on FA skeleton (right)
MMSE	beta1_1	0.179 [0.0191, 0.388]*	Mean MD in superior corona radiata on FA skeleton (right)
MMSE	c_prime_1	-0.505 [-0.687, -0.243]*	Mean MD in superior corona radiata on FA skeleton (right)
MMSE	total_1	-0.533 [-0.705, -0.268]*	Mean MD in superior corona radiata on FA skeleton (right)
MMSE	beta1_2	0.206 [0.0214, 0.399]*	Mean MD in superior corona radiata on FA skeleton (right)
MMSE	beta2_2	0.251 [0.0853, 0.412]*	Mean MD in superior corona radiata on FA skeleton (right)
MMSE	beta4_2	0.169 [0.0349, 0.291]*	Mean MD in superior corona radiata on FA skeleton (right)
MMSE	total_2	-0.262 [-0.469, -0.0361]*	Mean MD in superior corona radiata on FA skeleton (right)
MMSE	diff_beta2_2_0	0.21 [0.0481, 0.473]*	Mean MD in superior corona radiata on FA skeleton (right)
MMSE	diff_beta4_2_0	0.255 [0.0217, 0.466]*	Mean MD in superior corona radiata on FA skeleton (right)
MMSE	diff_ind_2_0	-0.0787 [-0.216, -1.456e-03]*	Mean MD in superior corona radiata on FA skeleton (right)
MoCA	beta1_0	0.213 [0.0157, 0.61]*	Mean MD in superior corona radiata on FA skeleton (right)
MoCA	c_prime_0	-0.246 [-0.39, -0.0782]*	Mean MD in superior corona radiata on FA skeleton (right)
MoCA	total_0	-0.22 [-0.363, -0.0657]*	Mean MD in superior corona radiata on FA skeleton (right)
MoCA	beta1_1	0.179 [0.0191, 0.388]*	Mean MD in superior corona radiata on FA skeleton (right)
MoCA	beta3_1	-0.16 [-0.268, -4.841e-03]*	Mean MD in superior corona radiata on FA skeleton (right)
MoCA	c_prime_1	-0.444 [-0.593, -0.206]*	Mean MD in superior corona radiata on FA skeleton (right)
MoCA	total_1	-0.472 [-0.612, -0.227]*	Mean MD in superior corona radiata on FA skeleton (right)
MoCA	beta1_2	0.206 [0.0214, 0.399]*	Mean MD in superior corona radiata on FA skeleton (right)
MoCA	beta2_2	0.251 [0.0853, 0.412]*	Mean MD in superior corona radiata on FA skeleton (right)
MoCA	beta4_2	0.169 [0.0349, 0.291]*	Mean MD in superior corona radiata on FA skeleton (right)
MoCA	c_prime_2	-0.335 [-0.493, -0.138]*	Mean MD in superior corona radiata on FA skeleton (right)
MoCA	total_2	-0.361 [-0.526, -0.184]*	Mean MD in superior corona radiata on FA skeleton (right)
MoCA	diff_beta2_2_0	0.21 [0.0481, 0.473]*	Mean MD in superior corona radiata on FA skeleton (right)
MoCA	diff_beta4_2_0	0.255 [0.0217, 0.466]*	Mean MD in superior corona radiata on FA skeleton (right)
MoCA	diff_ind_2_0	-0.0518 [-0.152, -1.518e-04]*	Mean MD in superior corona radiata on FA skeleton (right)
EM	beta1_0	0.213 [0.0157, 0.61]*	Mean MD in superior corona radiata on FA skeleton (right)
EM	c_prime_0	-0.25 [-0.372, -0.153]*	Mean MD in superior corona radiata on FA skeleton (right)
EM	total_0	-0.25 [-0.352, -0.146]*	Mean MD in superior corona radiata on FA skeleton (right)
EM	beta1_1	0.179 [0.0191, 0.388]*	Mean MD in superior corona radiata on FA skeleton (right)
EM	beta3_1	-0.207 [-0.335, -0.0712]*	Mean MD in superior corona radiata on FA skeleton (right)
EM	ind_1	-0.0427 [-0.15, -6.541e-04]*	Mean MD in superior corona radiata on FA skeleton (right)
EM	total_1	-0.204 [-0.332, -0.0676]*	Mean MD in superior corona radiata on FA skeleton (right)
EM	beta1_2	0.206 [0.0214, 0.399]*	Mean MD in superior corona radiata on FA skeleton (right)
EM	beta2_2	0.251 [0.0853, 0.412]*	Mean MD in superior corona radiata on FA skeleton (right)
EM	beta4_2	0.169 [0.0349, 0.291]*	Mean MD in superior corona radiata on FA skeleton (right)
EM	diff_beta2_2_0	0.21 [0.0481, 0.473]*	Mean MD in superior corona radiata on FA skeleton (right)
EM	diff_beta3_2_1	0.257 [0.0462, 0.453]*	Mean MD in superior corona radiata on FA skeleton (right)
EM	diff_beta4_2_0	0.255 [0.0217, 0.466]*	Mean MD in superior corona radiata on FA skeleton (right)
ISP	beta1_0	0.213 [0.0157, 0.61]*	Mean MD in superior corona radiata on FA skeleton (right)
ISP	c_prime_0	-0.187 [-0.317, -2.219e-03]*	Mean MD in superior corona radiata on FA skeleton (right)
ISP	total_0	-0.169 [-0.307, -7.943e-03]*	Mean MD in superior corona radiata on FA skeleton (right)
ISP	beta1_1	0.179 [0.0191, 0.388]*	Mean MD in superior corona radiata on FA skeleton (right)
ISP	c_prime_1	-0.404 [-0.524, -0.258]*	Mean MD in superior corona radiata on FA skeleton (right)
ISP	total_1	-0.399 [-0.514, -0.25]*	Mean MD in superior corona radiata on FA skeleton (right)
ISP	beta1_2	0.206 [0.0214, 0.399]*	Mean MD in superior corona radiata on FA skeleton (right)
ISP	beta2_2	0.251 [0.0853, 0.412]*	Mean MD in superior corona radiata on FA skeleton (right)
ISP	beta4_2	0.169 [0.0349, 0.291]*	Mean MD in superior corona radiata on FA skeleton (right)
ISP	c_prime_2	-0.295 [-0.433, -0.129]*	Mean MD in superior corona radiata on FA skeleton (right)
ISP	total_2	-0.267 [-0.389, -0.118]*	Mean MD in superior corona radiata on FA skeleton (right)
ISP	diff_beta2_2_0	0.21 [0.0481, 0.473]*	Mean MD in superior corona radiata on FA skeleton (right)
ISP	diff_beta4_2_0	0.255 [0.0217, 0.466]*	Mean MD in superior corona radiata on FA skeleton (right)
ISP	diff_c_prime_1_0	-0.217 [-0.432, -0.0258]*	Mean MD in superior corona radiata on FA skeleton (right)
ISP	diff_total_1_0	-0.23 [-0.426, -0.0261]*	Mean MD in superior corona radiata on FA skeleton (right)
LF	beta1_0	0.213 [0.0157, 0.61]*	Mean MD in superior corona radiata on FA skeleton (right)
LF	c_prime_0	-0.239 [-0.375, -0.0748]*	Mean MD in superior corona radiata on FA skeleton (right)
LF	total_0	-0.233 [-0.359, -0.0909]*	Mean MD in superior corona radiata on FA skeleton (right)
LF	beta1_1	0.179 [0.0191, 0.388]*	Mean MD in superior corona radiata on FA skeleton (right)
LF	c_prime_1	-0.237 [-0.374, -0.104]*	Mean MD in superior corona radiata on FA skeleton (right)
LF	total_1	-0.23 [-0.369, -0.1]*	Mean MD in superior corona radiata on FA skeleton (right)
LF	beta1_2	0.206 [0.0214, 0.399]*	Mean MD in superior corona radiata on FA skeleton (right)
LF	beta2_2	0.251 [0.0853, 0.412]*	Mean MD in superior corona radiata on FA skeleton (right)
LF	beta4_2	0.169 [0.0349, 0.291]*	Mean MD in superior corona radiata on FA skeleton (right)

LF	c_prime_2	-0.314 [-0.463, -0.137]*	Mean MD in superior corona radiata on FA skeleton (right)
LF	total_2	-0.294 [-0.436, -0.133]*	Mean MD in superior corona radiata on FA skeleton (right)
LF	diff_beta2_2_0	0.21 [0.0481, 0.473]*	Mean MD in superior corona radiata on FA skeleton (right)
LF	diff_beta4_2_0	0.255 [0.0217, 0.466]*	Mean MD in superior corona radiata on FA skeleton (right)
EF	beta1_0	0.213 [0.0157, 0.61]*	Mean MD in superior corona radiata on FA skeleton (right)
EF	beta1_1	0.179 [0.0191, 0.388]*	Mean MD in superior corona radiata on FA skeleton (right)
EF	c_prime_1	-0.347 [-0.479, -0.197]*	Mean MD in superior corona radiata on FA skeleton (right)
EF	total_1	-0.36 [-0.494, -0.207]*	Mean MD in superior corona radiata on FA skeleton (right)
EF	beta1_2	0.206 [0.0214, 0.399]*	Mean MD in superior corona radiata on FA skeleton (right)
EF	beta2_2	0.251 [0.0853, 0.412]*	Mean MD in superior corona radiata on FA skeleton (right)
EF	beta4_2	0.169 [0.0349, 0.291]*	Mean MD in superior corona radiata on FA skeleton (right)
EF	c_prime_2	-0.249 [-0.406, -0.0227]*	Mean MD in superior corona radiata on FA skeleton (right)
EF	total_2	-0.252 [-0.395, -0.0436]*	Mean MD in superior corona radiata on FA skeleton (right)
EF	diff_beta2_2_0	0.21 [0.0481, 0.473]*	Mean MD in superior corona radiata on FA skeleton (right)
EF	diff_beta4_2_0	0.255 [0.0217, 0.466]*	Mean MD in superior corona radiata on FA skeleton (right)
EF	diff_c_prime_1_0	-0.282 [-0.592, -0.0646]*	Mean MD in superior corona radiata on FA skeleton (right)
EF	diff_total_1_0	-0.31 [-0.606, -0.0808]*	Mean MD in superior corona radiata on FA skeleton (right)
VS	beta1_0	0.213 [0.0157, 0.61]*	Mean MD in superior corona radiata on FA skeleton (right)
VS	c_prime_0	-0.25 [-0.427, -0.0602]*	Mean MD in superior corona radiata on FA skeleton (right)
VS	total_0	-0.223 [-0.396, -0.0501]*	Mean MD in superior corona radiata on FA skeleton (right)
VS	beta1_1	0.179 [0.0191, 0.388]*	Mean MD in superior corona radiata on FA skeleton (right)
VS	c_prime_1	-0.194 [-0.379, -0.0118]*	Mean MD in superior corona radiata on FA skeleton (right)
VS	beta1_2	0.206 [0.0214, 0.399]*	Mean MD in superior corona radiata on FA skeleton (right)
VS	beta2_2	0.251 [0.0853, 0.412]*	Mean MD in superior corona radiata on FA skeleton (right)
VS	beta4_2	0.169 [0.0349, 0.291]*	Mean MD in superior corona radiata on FA skeleton (right)
VS	c_prime_2	-0.31 [-0.493, -0.11]*	Mean MD in superior corona radiata on FA skeleton (right)
VS	total_2	-0.292 [-0.475, -0.112]*	Mean MD in superior corona radiata on FA skeleton (right)
VS	diff_beta2_2_0	0.21 [0.0481, 0.473]*	Mean MD in superior corona radiata on FA skeleton (right)
VS	diff_beta4_2_0	0.255 [0.0217, 0.466]*	Mean MD in superior corona radiata on FA skeleton (right)
MMSE	beta1_0	0.213 [0.0157, 0.61]*	Mean MD in superior corona radiata on FA skeleton (left)
MMSE	c_prime_0	-0.327 [-0.528, -0.131]*	Mean MD in superior corona radiata on FA skeleton (left)
MMSE	total_0	-0.287 [-0.498, -0.1]*	Mean MD in superior corona radiata on FA skeleton (left)
MMSE	beta1_1	0.179 [0.0191, 0.388]*	Mean MD in superior corona radiata on FA skeleton (left)
MMSE	beta3_1	-0.166 [-0.318, -0.0588]*	Mean MD in superior corona radiata on FA skeleton (left)
MMSE	c_prime_1	-0.498 [-0.676, -0.238]*	Mean MD in superior corona radiata on FA skeleton (left)
MMSE	total_1	-0.533 [-0.705, -0.268]*	Mean MD in superior corona radiata on FA skeleton (left)
MMSE	beta1_2	0.206 [0.0214, 0.399]*	Mean MD in superior corona radiata on FA skeleton (left)
MMSE	beta2_2	0.283 [0.119, 0.469]*	Mean MD in superior corona radiata on FA skeleton (left)
MMSE	ind1_2	-8.322e-03 [-0.0467, -1.027e-03]*	Mean MD in superior corona radiata on FA skeleton (left)
MMSE	total_2	-0.262 [-0.469, -0.0361]*	Mean MD in superior corona radiata on FA skeleton (left)
MMSE	diff_beta2_2_0	0.182 [0.0118, 0.488]*	Mean MD in superior corona radiata on FA skeleton (left)
MoCA	beta1_0	0.213 [0.0157, 0.61]*	Mean MD in superior corona radiata on FA skeleton (left)
MoCA	c_prime_0	-0.244 [-0.387, -0.0742]*	Mean MD in superior corona radiata on FA skeleton (left)
MoCA	total_0	-0.22 [-0.363, -0.0657]*	Mean MD in superior corona radiata on FA skeleton (left)
MoCA	beta1_1	0.179 [0.0191, 0.388]*	Mean MD in superior corona radiata on FA skeleton (left)
MoCA	beta3_1	-0.187 [-0.304, -0.0772]*	Mean MD in superior corona radiata on FA skeleton (left)
MoCA	c_prime_1	-0.44 [-0.586, -0.203]*	Mean MD in superior corona radiata on FA skeleton (left)
MoCA	total_1	-0.472 [-0.612, -0.227]*	Mean MD in superior corona radiata on FA skeleton (left)
MoCA	beta1_2	0.206 [0.0214, 0.399]*	Mean MD in superior corona radiata on FA skeleton (left)
MoCA	beta2_2	0.283 [0.119, 0.469]*	Mean MD in superior corona radiata on FA skeleton (left)
MoCA	c_prime_2	-0.342 [-0.493, -0.147]*	Mean MD in superior corona radiata on FA skeleton (left)
MoCA	ind1_2	-5.597e-03 [-0.0326, -8.691e-04]*	Mean MD in superior corona radiata on FA skeleton (left)
MoCA	total_2	-0.361 [-0.526, -0.184]*	Mean MD in superior corona radiata on FA skeleton (left)
MoCA	diff_beta2_2_0	0.182 [0.0118, 0.488]*	Mean MD in superior corona radiata on FA skeleton (left)
EM	beta1_0	0.213 [0.0157, 0.61]*	Mean MD in superior corona radiata on FA skeleton (left)
EM	c_prime_0	-0.252 [-0.377, -0.154]*	Mean MD in superior corona radiata on FA skeleton (left)
EM	total_0	-0.25 [-0.352, -0.146]*	Mean MD in superior corona radiata on FA skeleton (left)
EM	beta1_1	0.179 [0.0191, 0.388]*	Mean MD in superior corona radiata on FA skeleton (left)
EM	beta3_1	-0.222 [-0.351, -0.0866]*	Mean MD in superior corona radiata on FA skeleton (left)
EM	c_prime_1	-0.159 [-0.295, -3.951e-04]*	Mean MD in superior corona radiata on FA skeleton (left)
EM	ind_1	-0.0449 [-0.154, -7.223e-04]*	Mean MD in superior corona radiata on FA skeleton (left)
EM	total_1	-0.204 [-0.332, -0.0676]*	Mean MD in superior corona radiata on FA skeleton (left)
EM	beta1_2	0.206 [0.0214, 0.399]*	Mean MD in superior corona radiata on FA skeleton (left)
EM	beta2_2	0.283 [0.119, 0.469]*	Mean MD in superior corona radiata on FA skeleton (left)
EM	diff_beta2_2_0	0.182 [0.0118, 0.488]*	Mean MD in superior corona radiata on FA skeleton (left)
ISP	beta1_0	0.213 [0.0157, 0.61]*	Mean MD in superior corona radiata on FA skeleton (left)
ISP	c_prime_0	-0.186 [-0.317, -1.961e-03]*	Mean MD in superior corona radiata on FA skeleton (left)
ISP	total_0	-0.169 [-0.307, -7.943e-03]*	Mean MD in superior corona radiata on FA skeleton (left)
ISP	beta1_1	0.179 [0.0191, 0.388]*	Mean MD in superior corona radiata on FA skeleton (left)
ISP	c_prime_1	-0.399 [-0.517, -0.251]*	Mean MD in superior corona radiata on FA skeleton (left)
ISP	total_1	-0.399 [-0.514, -0.25]*	Mean MD in superior corona radiata on FA skeleton (left)
ISP	beta1_2	0.206 [0.0214, 0.399]*	Mean MD in superior corona radiata on FA skeleton (left)
ISP	beta2_2	0.283 [0.119, 0.469]*	Mean MD in superior corona radiata on FA skeleton (left)
ISP	c_prime_2	-0.289 [-0.42, -0.129]*	Mean MD in superior corona radiata on FA skeleton (left)
ISP	total_2	-0.267 [-0.389, -0.118]*	Mean MD in superior corona radiata on FA skeleton (left)
ISP	diff_beta2_2_0	0.182 [0.0118, 0.488]*	Mean MD in superior corona radiata on FA skeleton (left)
ISP	diff_c_prime_1_0	-0.214 [-0.431, -0.0249]*	Mean MD in superior corona radiata on FA skeleton (left)
ISP	diff_total_1_0	-0.23 [-0.426, -0.0261]*	Mean MD in superior corona radiata on FA skeleton (left)
LF	beta1_0	0.213 [0.0157, 0.61]*	Mean MD in superior corona radiata on FA skeleton (left)
LF	c_prime_0	-0.237 [-0.376, -0.0768]*	Mean MD in superior corona radiata on FA skeleton (left)
LF	total_0	-0.233 [-0.359, -0.0909]*	Mean MD in superior corona radiata on FA skeleton (left)
LF	beta1_1	0.179 [0.0191, 0.388]*	Mean MD in superior corona radiata on FA skeleton (left)
LF	c_prime_1	-0.237 [-0.376, -0.102]*	Mean MD in superior corona radiata on FA skeleton (left)
LF	total_1	-0.23 [-0.369, -0.1]*	Mean MD in superior corona radiata on FA skeleton (left)

LF	beta1_2	0.206 [0.0214, 0.399]*	Mean MD in superior corona radiata on FA skeleton (left)
LF	beta2_2	0.283 [0.119, 0.469]*	Mean MD in superior corona radiata on FA skeleton (left)
LF	c_prime_2	-0.309 [-0.454, -0.132]*	Mean MD in superior corona radiata on FA skeleton (left)
LF	total_2	-0.294 [-0.436, -0.133]*	Mean MD in superior corona radiata on FA skeleton (left)
LF	diff_beta2_2_0	0.182 [0.0118, 0.488]*	Mean MD in superior corona radiata on FA skeleton (left)
EF	beta1_0	0.213 [0.0157, 0.61]*	Mean MD in superior corona radiata on FA skeleton (left)
EF	beta1_1	0.179 [0.0191, 0.388]*	Mean MD in superior corona radiata on FA skeleton (left)
EF	c_prime_1	-0.34 [-0.471, -0.189]*	Mean MD in superior corona radiata on FA skeleton (left)
EF	total_1	-0.36 [-0.494, -0.207]*	Mean MD in superior corona radiata on FA skeleton (left)
EF	beta1_2	0.206 [0.0214, 0.399]*	Mean MD in superior corona radiata on FA skeleton (left)
EF	beta2_2	0.283 [0.119, 0.469]*	Mean MD in superior corona radiata on FA skeleton (left)
EF	c_prime_2	-0.248 [-0.4, -0.0303]*	Mean MD in superior corona radiata on FA skeleton (left)
EF	ind1_2	-5.338e-03 [-0.0339, -5.307e-05]*	Mean MD in superior corona radiata on FA skeleton (left)
EF	total_2	-0.252 [-0.395, -0.0436]*	Mean MD in superior corona radiata on FA skeleton (left)
EF	diff_beta2_2_0	0.182 [0.0118, 0.488]*	Mean MD in superior corona radiata on FA skeleton (left)
EF	diff_c_prime_1_0	-0.277 [-0.59, -0.0596]*	Mean MD in superior corona radiata on FA skeleton (left)
EF	diff_total_1_0	-0.31 [-0.606, -0.0808]*	Mean MD in superior corona radiata on FA skeleton (left)
VS	beta1_0	0.213 [0.0157, 0.61]*	Mean MD in superior corona radiata on FA skeleton (left)
VS	c_prime_0	-0.25 [-0.428, -0.0596]*	Mean MD in superior corona radiata on FA skeleton (left)
VS	total_0	-0.223 [-0.396, -0.0501]*	Mean MD in superior corona radiata on FA skeleton (left)
VS	beta1_1	0.179 [0.0191, 0.388]*	Mean MD in superior corona radiata on FA skeleton (left)
VS	c_prime_1	-0.19 [-0.376, -0.0132]*	Mean MD in superior corona radiata on FA skeleton (left)
VS	beta1_2	0.206 [0.0214, 0.399]*	Mean MD in superior corona radiata on FA skeleton (left)
VS	beta2_2	0.283 [0.119, 0.469]*	Mean MD in superior corona radiata on FA skeleton (left)
VS	c_prime_2	-0.301 [-0.477, -0.0966]*	Mean MD in superior corona radiata on FA skeleton (left)
VS	total_2	-0.292 [-0.475, -0.112]*	Mean MD in superior corona radiata on FA skeleton (left)
VS	diff_beta2_2_0	0.182 [0.0118, 0.488]*	Mean MD in superior corona radiata on FA skeleton (left)
MMSE	beta1_0	0.213 [0.0157, 0.61]*	Mean MD in posterior corona radiata on FA skeleton (right)
MMSE	c_prime_0	-0.316 [-0.52, -0.136]*	Mean MD in posterior corona radiata on FA skeleton (right)
MMSE	total_0	-0.287 [-0.498, -0.1]*	Mean MD in posterior corona radiata on FA skeleton (right)
MMSE	beta1_1	0.179 [0.0191, 0.388]*	Mean MD in posterior corona radiata on FA skeleton (right)
MMSE	beta4_1	0.199 [0.0452, 0.398]*	Mean MD in posterior corona radiata on FA skeleton (right)
MMSE	c_prime_1	-0.495 [-0.677, -0.236]*	Mean MD in posterior corona radiata on FA skeleton (right)
MMSE	total_1	-0.533 [-0.705, -0.268]*	Mean MD in posterior corona radiata on FA skeleton (right)
MMSE	beta1_2	0.206 [0.0214, 0.399]*	Mean MD in posterior corona radiata on FA skeleton (right)
MMSE	beta4_2	0.191 [0.0103, 0.319]*	Mean MD in posterior corona radiata on FA skeleton (right)
MMSE	beta3_2	-0.218 [-0.426, -0.0605]*	Mean MD in posterior corona radiata on FA skeleton (right)
MMSE	ind1_2	-7.593e-03 [-0.0489, -6.137e-04]*	Mean MD in posterior corona radiata on FA skeleton (right)
MMSE	ind3_2	-0.0385 [-0.106, -8.799e-03]*	Mean MD in posterior corona radiata on FA skeleton (right)
MMSE	ind_2	-0.0557 [-0.154, -0.013]*	Mean MD in posterior corona radiata on FA skeleton (right)
MMSE	total_2	-0.262 [-0.469, -0.0361]*	Mean MD in posterior corona radiata on FA skeleton (right)
MMSE	diff_beta4_1_0	0.215 [0.0206, 0.491]*	Mean MD in posterior corona radiata on FA skeleton (right)
MMSE	diff_ind3_2_0	-0.0362 [-0.126, -5.344e-03]*	Mean MD in posterior corona radiata on FA skeleton (right)
MMSE	diff_ind_2_0	-0.0848 [-0.242, -0.0156]*	Mean MD in posterior corona radiata on FA skeleton (right)
MoCA	beta1_0	0.213 [0.0157, 0.61]*	Mean MD in posterior corona radiata on FA skeleton (right)
MoCA	beta3_0	-0.212 [-0.353, -0.0457]*	Mean MD in posterior corona radiata on FA skeleton (right)
MoCA	c_prime_0	-0.237 [-0.386, -0.0828]*	Mean MD in posterior corona radiata on FA skeleton (right)
MoCA	total_0	-0.22 [-0.363, -0.0657]*	Mean MD in posterior corona radiata on FA skeleton (right)
MoCA	beta1_1	0.179 [0.0191, 0.388]*	Mean MD in posterior corona radiata on FA skeleton (right)
MoCA	beta4_1	0.199 [0.0452, 0.398]*	Mean MD in posterior corona radiata on FA skeleton (right)
MoCA	c_prime_1	-0.429 [-0.579, -0.185]*	Mean MD in posterior corona radiata on FA skeleton (right)
MoCA	ind3_1	-0.0354 [-0.1, -2.183e-03]*	Mean MD in posterior corona radiata on FA skeleton (right)
MoCA	ind_1	-0.043 [-0.125, -2.504e-03]*	Mean MD in posterior corona radiata on FA skeleton (right)
MoCA	total_1	-0.472 [-0.612, -0.227]*	Mean MD in posterior corona radiata on FA skeleton (right)
MoCA	beta1_2	0.206 [0.0214, 0.399]*	Mean MD in posterior corona radiata on FA skeleton (right)
MoCA	beta4_2	0.191 [0.0103, 0.319]*	Mean MD in posterior corona radiata on FA skeleton (right)
MoCA	c_prime_2	-0.326 [-0.474, -0.113]*	Mean MD in posterior corona radiata on FA skeleton (right)
MoCA	ind1_2	-4.491e-03 [-0.0362, -8.968e-05]*	Mean MD in posterior corona radiata on FA skeleton (right)
MoCA	ind3_2	-0.021 [-0.0839, -7.882e-04]*	Mean MD in posterior corona radiata on FA skeleton (right)
MoCA	total_2	-0.361 [-0.526, -0.184]*	Mean MD in posterior corona radiata on FA skeleton (right)
MoCA	diff_beta4_1_0	0.215 [0.0206, 0.491]*	Mean MD in posterior corona radiata on FA skeleton (right)
EM	beta1_0	0.213 [0.0157, 0.61]*	Mean MD in posterior corona radiata on FA skeleton (right)
EM	c_prime_0	-0.236 [-0.377, -0.152]*	Mean MD in posterior corona radiata on FA skeleton (right)
EM	total_0	-0.25 [-0.352, -0.146]*	Mean MD in posterior corona radiata on FA skeleton (right)
EM	beta1_1	0.179 [0.0191, 0.388]*	Mean MD in posterior corona radiata on FA skeleton (right)
EM	beta4_1	0.199 [0.0452, 0.398]*	Mean MD in posterior corona radiata on FA skeleton (right)
EM	beta3_1	-0.173 [-0.313, -0.0246]*	Mean MD in posterior corona radiata on FA skeleton (right)
EM	ind3_1	-0.0332 [-0.0921, -4.567e-03]*	Mean MD in posterior corona radiata on FA skeleton (right)
EM	ind_1	-0.0527 [-0.15, -9.172e-03]*	Mean MD in posterior corona radiata on FA skeleton (right)
EM	total_1	-0.204 [-0.332, -0.0676]*	Mean MD in posterior corona radiata on FA skeleton (right)
EM	beta1_2	0.206 [0.0214, 0.399]*	Mean MD in posterior corona radiata on FA skeleton (right)
EM	beta4_2	0.191 [0.0103, 0.319]*	Mean MD in posterior corona radiata on FA skeleton (right)
EM	diff_beta4_1_0	0.215 [0.0206, 0.491]*	Mean MD in posterior corona radiata on FA skeleton (right)
ISP	beta1_0	0.213 [0.0157, 0.61]*	Mean MD in posterior corona radiata on FA skeleton (right)
ISP	c_prime_0	-0.183 [-0.311, -0.0118]*	Mean MD in posterior corona radiata on FA skeleton (right)
ISP	total_0	-0.169 [-0.307, -7.943e-03]*	Mean MD in posterior corona radiata on FA skeleton (right)
ISP	beta1_1	0.179 [0.0191, 0.388]*	Mean MD in posterior corona radiata on FA skeleton (right)
ISP	beta4_1	0.199 [0.0452, 0.398]*	Mean MD in posterior corona radiata on FA skeleton (right)
ISP	c_prime_1	-0.406 [-0.522, -0.256]*	Mean MD in posterior corona radiata on FA skeleton (right)
ISP	total_1	-0.399 [-0.514, -0.25]*	Mean MD in posterior corona radiata on FA skeleton (right)
ISP	beta1_2	0.206 [0.0214, 0.399]*	Mean MD in posterior corona radiata on FA skeleton (right)
ISP	beta4_2	0.191 [0.0103, 0.319]*	Mean MD in posterior corona radiata on FA skeleton (right)
ISP	c_prime_2	-0.282 [-0.409, -0.109]*	Mean MD in posterior corona radiata on FA skeleton (right)
ISP	total_2	-0.267 [-0.389, -0.118]*	Mean MD in posterior corona radiata on FA skeleton (right)

ISP	diff_beta4_1_0	0.215 [0.0206, 0.491]*	Mean MD in posterior corona radiata on FA skeleton (right)
ISP	diff_c_prime_1_0	-0.223 [-0.424, -0.03]*	Mean MD in posterior corona radiata on FA skeleton (right)
ISP	diff_total_1_0	-0.23 [-0.426, -0.0261]*	Mean MD in posterior corona radiata on FA skeleton (right)
LF	beta1_0	0.213 [0.0157, 0.61]*	Mean MD in posterior corona radiata on FA skeleton (right)
LF	c_prime_0	-0.236 [-0.371, -0.0851]*	Mean MD in posterior corona radiata on FA skeleton (right)
LF	total_0	-0.233 [-0.359, -0.0909]*	Mean MD in posterior corona radiata on FA skeleton (right)
LF	beta1_1	0.179 [0.0191, 0.388]*	Mean MD in posterior corona radiata on FA skeleton (right)
LF	beta4_1	0.199 [0.0452, 0.398]*	Mean MD in posterior corona radiata on FA skeleton (right)
LF	c_prime_1	-0.224 [-0.363, -0.0936]*	Mean MD in posterior corona radiata on FA skeleton (right)
LF	total_1	-0.23 [-0.369, -0.1]*	Mean MD in posterior corona radiata on FA skeleton (right)
LF	beta1_2	0.206 [0.0214, 0.399]*	Mean MD in posterior corona radiata on FA skeleton (right)
LF	beta4_2	0.191 [0.0103, 0.319]*	Mean MD in posterior corona radiata on FA skeleton (right)
LF	c_prime_2	-0.312 [-0.457, -0.127]*	Mean MD in posterior corona radiata on FA skeleton (right)
LF	total_2	-0.294 [-0.436, -0.133]*	Mean MD in posterior corona radiata on FA skeleton (right)
LF	diff_beta4_1_0	0.215 [0.0206, 0.491]*	Mean MD in posterior corona radiata on FA skeleton (right)
EF	beta1_0	0.213 [0.0157, 0.61]*	Mean MD in posterior corona radiata on FA skeleton (right)
EF	beta1_1	0.179 [0.0191, 0.388]*	Mean MD in posterior corona radiata on FA skeleton (right)
EF	beta4_1	0.199 [0.0452, 0.398]*	Mean MD in posterior corona radiata on FA skeleton (right)
EF	c_prime_1	-0.341 [-0.468, -0.187]*	Mean MD in posterior corona radiata on FA skeleton (right)
EF	total_1	-0.36 [-0.494, -0.207]*	Mean MD in posterior corona radiata on FA skeleton (right)
EF	beta1_2	0.206 [0.0214, 0.399]*	Mean MD in posterior corona radiata on FA skeleton (right)
EF	beta4_2	0.191 [0.0103, 0.319]*	Mean MD in posterior corona radiata on FA skeleton (right)
EF	c_prime_2	-0.24 [-0.389, -4.810e-03]*	Mean MD in posterior corona radiata on FA skeleton (right)
EF	total_2	-0.252 [-0.395, -0.0436]*	Mean MD in posterior corona radiata on FA skeleton (right)
EF	diff_beta4_1_0	0.215 [0.0206, 0.491]*	Mean MD in posterior corona radiata on FA skeleton (right)
EF	diff_c_prime_1_0	-0.283 [-0.595, -0.061]*	Mean MD in posterior corona radiata on FA skeleton (right)
EF	diff_total_1_0	-0.31 [-0.606, -0.0808]*	Mean MD in posterior corona radiata on FA skeleton (right)
VS	beta1_0	0.213 [0.0157, 0.61]*	Mean MD in posterior corona radiata on FA skeleton (right)
VS	c_prime_0	-0.245 [-0.429, -0.0667]*	Mean MD in posterior corona radiata on FA skeleton (right)
VS	total_0	-0.223 [-0.396, -0.0501]*	Mean MD in posterior corona radiata on FA skeleton (right)
VS	beta1_1	0.179 [0.0191, 0.388]*	Mean MD in posterior corona radiata on FA skeleton (right)
VS	beta4_1	0.199 [0.0452, 0.398]*	Mean MD in posterior corona radiata on FA skeleton (right)
VS	ind3_1	-0.0315 [-0.1, -8.149e-04]*	Mean MD in posterior corona radiata on FA skeleton (right)
VS	beta1_2	0.206 [0.0214, 0.399]*	Mean MD in posterior corona radiata on FA skeleton (right)
VS	beta4_2	0.191 [0.0103, 0.319]*	Mean MD in posterior corona radiata on FA skeleton (right)
VS	c_prime_2	-0.3 [-0.475, -0.0894]*	Mean MD in posterior corona radiata on FA skeleton (right)
VS	total_2	-0.292 [-0.475, -0.112]*	Mean MD in posterior corona radiata on FA skeleton (right)
VS	diff_beta4_1_0	0.215 [0.0206, 0.491]*	Mean MD in posterior corona radiata on FA skeleton (right)
MMSE	beta1_0	0.213 [0.0157, 0.61]*	Mean MD in posterior corona radiata on FA skeleton (left)
MMSE	beta3_0	-0.224 [-0.515, -6.815e-03]*	Mean MD in posterior corona radiata on FA skeleton (left)
MMSE	c_prime_0	-0.31 [-0.527, -0.139]*	Mean MD in posterior corona radiata on FA skeleton (left)
MMSE	total_0	-0.287 [-0.498, -0.1]*	Mean MD in posterior corona radiata on FA skeleton (left)
MMSE	beta1_1	0.179 [0.0191, 0.388]*	Mean MD in posterior corona radiata on FA skeleton (left)
MMSE	beta4_1	0.172 [0.0111, 0.388]*	Mean MD in posterior corona radiata on FA skeleton (left)
MMSE	c_prime_1	-0.489 [-0.665, -0.218]*	Mean MD in posterior corona radiata on FA skeleton (left)
MMSE	ind_1	-0.0443 [-0.146, -2.435e-03]*	Mean MD in posterior corona radiata on FA skeleton (left)
MMSE	total_1	-0.533 [-0.705, -0.268]*	Mean MD in posterior corona radiata on FA skeleton (left)
MMSE	beta1_2	0.206 [0.0214, 0.399]*	Mean MD in posterior corona radiata on FA skeleton (left)
MMSE	beta4_2	0.21 [0.052, 0.336]*	Mean MD in posterior corona radiata on FA skeleton (left)
MMSE	ind1_2	-5.276e-03 [-0.0383, -1.384e-04]*	Mean MD in posterior corona radiata on FA skeleton (left)
MMSE	ind3_2	-0.0289 [-0.0958, -3.138e-03]*	Mean MD in posterior corona radiata on FA skeleton (left)
MMSE	ind_2	-0.046 [-0.15, -1.621e-03]*	Mean MD in posterior corona radiata on FA skeleton (left)
MMSE	total_2	-0.262 [-0.469, -0.0361]*	Mean MD in posterior corona radiata on FA skeleton (left)
MMSE	diff_beta4_2_0	0.212 [0.0356, 0.455]*	Mean MD in posterior corona radiata on FA skeleton (left)
MMSE	diff_ind_2_0	-0.069 [-0.246, -4.690e-03]*	Mean MD in posterior corona radiata on FA skeleton (left)
MoCA	beta1_0	0.213 [0.0157, 0.61]*	Mean MD in posterior corona radiata on FA skeleton (left)
MoCA	beta3_0	-0.264 [-0.423, -0.0555]*	Mean MD in posterior corona radiata on FA skeleton (left)
MoCA	c_prime_0	-0.231 [-0.388, -0.0841]*	Mean MD in posterior corona radiata on FA skeleton (left)
MoCA	total_0	-0.22 [-0.363, -0.0657]*	Mean MD in posterior corona radiata on FA skeleton (left)
MoCA	beta1_1	0.179 [0.0191, 0.388]*	Mean MD in posterior corona radiata on FA skeleton (left)
MoCA	beta4_1	0.172 [0.0111, 0.388]*	Mean MD in posterior corona radiata on FA skeleton (left)
MoCA	beta3_1	-0.182 [-0.3, -0.0338]*	Mean MD in posterior corona radiata on FA skeleton (left)
MoCA	c_prime_1	-0.431 [-0.577, -0.178]*	Mean MD in posterior corona radiata on FA skeleton (left)
MoCA	ind3_1	-0.0336 [-0.099, -6.586e-04]*	Mean MD in posterior corona radiata on FA skeleton (left)
MoCA	total_1	-0.472 [-0.612, -0.227]*	Mean MD in posterior corona radiata on FA skeleton (left)
MoCA	beta1_2	0.206 [0.0214, 0.399]*	Mean MD in posterior corona radiata on FA skeleton (left)
MoCA	beta4_2	0.21 [0.052, 0.336]*	Mean MD in posterior corona radiata on FA skeleton (left)
MoCA	beta3_2	-0.144 [-0.326, -3.843e-03]*	Mean MD in posterior corona radiata on FA skeleton (left)
MoCA	c_prime_2	-0.32 [-0.47, -0.111]*	Mean MD in posterior corona radiata on FA skeleton (left)
MoCA	ind1_2	-5.057e-03 [-0.0348, -2.317e-04]*	Mean MD in posterior corona radiata on FA skeleton (left)
MoCA	ind3_2	-0.0276 [-0.0873, -5.294e-03]*	Mean MD in posterior corona radiata on FA skeleton (left)
MoCA	ind_2	-0.041 [-0.129, -4.711e-03]*	Mean MD in posterior corona radiata on FA skeleton (left)
MoCA	total_2	-0.361 [-0.526, -0.184]*	Mean MD in posterior corona radiata on FA skeleton (left)
MoCA	diff_beta4_2_0	0.212 [0.0356, 0.455]*	Mean MD in posterior corona radiata on FA skeleton (left)
MoCA	diff_ind_2_0	-0.0521 [-0.179, -3.498e-03]*	Mean MD in posterior corona radiata on FA skeleton (left)
EM	beta1_0	0.213 [0.0157, 0.61]*	Mean MD in posterior corona radiata on FA skeleton (left)
EM	c_prime_0	-0.231 [-0.378, -0.151]*	Mean MD in posterior corona radiata on FA skeleton (left)
EM	total_0	-0.25 [-0.352, -0.146]*	Mean MD in posterior corona radiata on FA skeleton (left)
EM	beta1_1	0.179 [0.0191, 0.388]*	Mean MD in posterior corona radiata on FA skeleton (left)
EM	beta4_1	0.172 [0.0111, 0.388]*	Mean MD in posterior corona radiata on FA skeleton (left)
EM	beta3_1	-0.249 [-0.377, -0.1]*	Mean MD in posterior corona radiata on FA skeleton (left)
EM	ind3_1	-0.0404 [-0.107, -5.828e-03]*	Mean MD in posterior corona radiata on FA skeleton (left)
EM	ind_1	-0.0599 [-0.165, -0.0122]*	Mean MD in posterior corona radiata on FA skeleton (left)
EM	total_1	-0.204 [-0.332, -0.0676]*	Mean MD in posterior corona radiata on FA skeleton (left)

EM	beta1_2	0.206 [0.0214, 0.399]*	Mean MD in posterior corona radiata on FA skeleton (left)
EM	beta4_2	0.21 [0.052, 0.336]*	Mean MD in posterior corona radiata on FA skeleton (left)
EM	diff_beta3_2_1	0.277 [0.0393, 0.46]*	Mean MD in posterior corona radiata on FA skeleton (left)
EM	diff_beta4_2_0	0.212 [0.0356, 0.455]*	Mean MD in posterior corona radiata on FA skeleton (left)
EM	diff_ind3_1_0	-0.0316 [-0.134, -1.738e-03]*	Mean MD in posterior corona radiata on FA skeleton (left)
EM	diff_ind_1_0	-0.0412 [-0.195, -6.274e-04]*	Mean MD in posterior corona radiata on FA skeleton (left)
ISP	beta1_0	0.213 [0.0157, 0.61]*	Mean MD in posterior corona radiata on FA skeleton (left)
ISP	beta3_0	-0.189 [-0.331, -1.062e-03]*	Mean MD in posterior corona radiata on FA skeleton (left)
ISP	c_prime_0	-0.177 [-0.313, -0.0158]*	Mean MD in posterior corona radiata on FA skeleton (left)
ISP	total_0	-0.169 [-0.307, -7.943e-03]*	Mean MD in posterior corona radiata on FA skeleton (left)
ISP	beta1_1	0.179 [0.0191, 0.388]*	Mean MD in posterior corona radiata on FA skeleton (left)
ISP	beta4_1	0.172 [0.0111, 0.388]*	Mean MD in posterior corona radiata on FA skeleton (left)
ISP	c_prime_1	-0.397 [-0.51, -0.247]*	Mean MD in posterior corona radiata on FA skeleton (left)
ISP	total_1	-0.399 [-0.514, -0.25]*	Mean MD in posterior corona radiata on FA skeleton (left)
ISP	beta1_2	0.206 [0.0214, 0.399]*	Mean MD in posterior corona radiata on FA skeleton (left)
ISP	beta4_2	0.21 [0.052, 0.336]*	Mean MD in posterior corona radiata on FA skeleton (left)
ISP	c_prime_2	-0.288 [-0.419, -0.114]*	Mean MD in posterior corona radiata on FA skeleton (left)
ISP	total_2	-0.267 [-0.389, -0.118]*	Mean MD in posterior corona radiata on FA skeleton (left)
ISP	diff_beta4_2_0	0.212 [0.0356, 0.455]*	Mean MD in posterior corona radiata on FA skeleton (left)
ISP	diff_c_prime_1_0	-0.22 [-0.414, -0.0209]*	Mean MD in posterior corona radiata on FA skeleton (left)
ISP	diff_total_1_0	-0.23 [-0.426, -0.0261]*	Mean MD in posterior corona radiata on FA skeleton (left)
LF	beta1_0	0.213 [0.0157, 0.61]*	Mean MD in posterior corona radiata on FA skeleton (left)
LF	c_prime_0	-0.231 [-0.375, -0.093]*	Mean MD in posterior corona radiata on FA skeleton (left)
LF	total_0	-0.233 [-0.359, -0.0909]*	Mean MD in posterior corona radiata on FA skeleton (left)
LF	beta1_1	0.179 [0.0191, 0.388]*	Mean MD in posterior corona radiata on FA skeleton (left)
LF	beta4_1	0.172 [0.0111, 0.388]*	Mean MD in posterior corona radiata on FA skeleton (left)
LF	c_prime_1	-0.232 [-0.376, -0.0963]*	Mean MD in posterior corona radiata on FA skeleton (left)
LF	total_1	-0.23 [-0.369, -0.1]*	Mean MD in posterior corona radiata on FA skeleton (left)
LF	beta1_2	0.206 [0.0214, 0.399]*	Mean MD in posterior corona radiata on FA skeleton (left)
LF	beta4_2	0.21 [0.052, 0.336]*	Mean MD in posterior corona radiata on FA skeleton (left)
LF	c_prime_2	-0.31 [-0.454, -0.121]*	Mean MD in posterior corona radiata on FA skeleton (left)
LF	total_2	-0.294 [-0.436, -0.133]*	Mean MD in posterior corona radiata on FA skeleton (left)
LF	diff_beta4_2_0	0.212 [0.0356, 0.455]*	Mean MD in posterior corona radiata on FA skeleton (left)
EF	beta1_0	0.213 [0.0157, 0.61]*	Mean MD in posterior corona radiata on FA skeleton (left)
EF	beta1_1	0.179 [0.0191, 0.388]*	Mean MD in posterior corona radiata on FA skeleton (left)
EF	beta4_1	0.172 [0.0111, 0.388]*	Mean MD in posterior corona radiata on FA skeleton (left)
EF	c_prime_1	-0.337 [-0.464, -0.183]*	Mean MD in posterior corona radiata on FA skeleton (left)
EF	total_1	-0.36 [-0.494, -0.207]*	Mean MD in posterior corona radiata on FA skeleton (left)
EF	beta1_2	0.206 [0.0214, 0.399]*	Mean MD in posterior corona radiata on FA skeleton (left)
EF	beta4_2	0.21 [0.052, 0.336]*	Mean MD in posterior corona radiata on FA skeleton (left)
EF	c_prime_2	-0.251 [-0.401, -0.014]*	Mean MD in posterior corona radiata on FA skeleton (left)
EF	total_2	-0.252 [-0.395, -0.0436]*	Mean MD in posterior corona radiata on FA skeleton (left)
EF	diff_beta4_2_0	0.212 [0.0356, 0.455]*	Mean MD in posterior corona radiata on FA skeleton (left)
EF	diff_c_prime_1_0	-0.282 [-0.586, -0.0551]*	Mean MD in posterior corona radiata on FA skeleton (left)
EF	diff_total_1_0	-0.31 [-0.606, -0.0808]*	Mean MD in posterior corona radiata on FA skeleton (left)
VS	beta1_0	0.213 [0.0157, 0.61]*	Mean MD in posterior corona radiata on FA skeleton (left)
VS	c_prime_0	-0.241 [-0.437, -0.0705]*	Mean MD in posterior corona radiata on FA skeleton (left)
VS	total_0	-0.223 [-0.396, -0.0501]*	Mean MD in posterior corona radiata on FA skeleton (left)
VS	beta1_1	0.179 [0.0191, 0.388]*	Mean MD in posterior corona radiata on FA skeleton (left)
VS	beta4_1	0.172 [0.0111, 0.388]*	Mean MD in posterior corona radiata on FA skeleton (left)
VS	beta1_2	0.206 [0.0214, 0.399]*	Mean MD in posterior corona radiata on FA skeleton (left)
VS	beta4_2	0.21 [0.052, 0.336]*	Mean MD in posterior corona radiata on FA skeleton (left)
VS	c_prime_2	-0.306 [-0.484, -0.0955]*	Mean MD in posterior corona radiata on FA skeleton (left)
VS	total_2	-0.292 [-0.475, -0.112]*	Mean MD in posterior corona radiata on FA skeleton (left)
VS	diff_beta4_2_0	0.212 [0.0356, 0.455]*	Mean MD in posterior corona radiata on FA skeleton (left)
MMSE	beta1_0	0.213 [0.0157, 0.61]*	Mean MD in posterior thalamic radiation on FA skeleton (right)
MMSE	beta3_0	-0.295 [-0.524, -0.106]*	Mean MD in posterior thalamic radiation on FA skeleton (right)
MMSE	c_prime_0	-0.314 [-0.52, -0.144]*	Mean MD in posterior thalamic radiation on FA skeleton (right)
MMSE	total_0	-0.287 [-0.498, -0.1]*	Mean MD in posterior thalamic radiation on FA skeleton (right)
MMSE	beta1_1	0.179 [0.0191, 0.388]*	Mean MD in posterior thalamic radiation on FA skeleton (right)
MMSE	beta4_1	0.198 [0.0315, 0.373]*	Mean MD in posterior thalamic radiation on FA skeleton (right)
MMSE	c_prime_1	-0.494 [-0.673, -0.235]*	Mean MD in posterior thalamic radiation on FA skeleton (right)
MMSE	ind_1	-0.0397 [-0.128, -6.378e-04]*	Mean MD in posterior thalamic radiation on FA skeleton (right)
MMSE	total_1	-0.533 [-0.705, -0.268]*	Mean MD in posterior thalamic radiation on FA skeleton (right)
MMSE	beta1_2	0.206 [0.0214, 0.399]*	Mean MD in posterior thalamic radiation on FA skeleton (right)
MMSE	beta4_2	0.222 [0.0788, 0.362]*	Mean MD in posterior thalamic radiation on FA skeleton (right)
MMSE	ind1_2	-4.317e-03 [-0.0295, -2.049e-04]*	Mean MD in posterior thalamic radiation on FA skeleton (right)
MMSE	ind3_2	-0.0302 [-0.0958, -9.022e-04]*	Mean MD in posterior thalamic radiation on FA skeleton (right)
MMSE	ind_2	-0.0473 [-0.159, -3.530e-03]*	Mean MD in posterior thalamic radiation on FA skeleton (right)
MMSE	total_2	-0.262 [-0.469, -0.0361]*	Mean MD in posterior thalamic radiation on FA skeleton (right)
MMSE	diff_beta4_2_0	0.22 [0.0157, 0.446]*	Mean MD in posterior thalamic radiation on FA skeleton (right)
MoCA	beta1_0	0.213 [0.0157, 0.61]*	Mean MD in posterior thalamic radiation on FA skeleton (right)
MoCA	beta3_0	-0.258 [-0.37, -0.132]*	Mean MD in posterior thalamic radiation on FA skeleton (right)
MoCA	c_prime_0	-0.235 [-0.382, -0.0852]*	Mean MD in posterior thalamic radiation on FA skeleton (right)
MoCA	total_0	-0.22 [-0.363, -0.0657]*	Mean MD in posterior thalamic radiation on FA skeleton (right)
MoCA	beta1_1	0.179 [0.0191, 0.388]*	Mean MD in posterior thalamic radiation on FA skeleton (right)
MoCA	beta4_1	0.198 [0.0315, 0.373]*	Mean MD in posterior thalamic radiation on FA skeleton (right)
MoCA	beta3_1	-0.172 [-0.31, -0.0111]*	Mean MD in posterior thalamic radiation on FA skeleton (right)
MoCA	c_prime_1	-0.431 [-0.576, -0.196]*	Mean MD in posterior thalamic radiation on FA skeleton (right)
MoCA	ind3_1	-0.0339 [-0.088, -2.522e-03]*	Mean MD in posterior thalamic radiation on FA skeleton (right)
MoCA	ind_1	-0.0414 [-0.112, -2.661e-03]*	Mean MD in posterior thalamic radiation on FA skeleton (right)
MoCA	total_1	-0.472 [-0.612, -0.227]*	Mean MD in posterior thalamic radiation on FA skeleton (right)
MoCA	beta1_2	0.206 [0.0214, 0.399]*	Mean MD in posterior thalamic radiation on FA skeleton (right)
MoCA	beta4_2	0.222 [0.0788, 0.362]*	Mean MD in posterior thalamic radiation on FA skeleton (right)

MoCA	c_prime_2	-0.331 [-0.482, -0.123]*	Mean MD in posterior thalamic radiation on FA skeleton (right)
MoCA	total_2	-0.361 [-0.526, -0.184]*	Mean MD in posterior thalamic radiation on FA skeleton (right)
MoCA	diff_beta4_2_0	0.22 [0.0157, 0.446]*	Mean MD in posterior thalamic radiation on FA skeleton (right)
EM	beta1_0	0.213 [0.0157, 0.61]*	Mean MD in posterior thalamic radiation on FA skeleton (right)
EM	beta3_0	-0.241 [-0.359, -0.0471]*	Mean MD in posterior thalamic radiation on FA skeleton (right)
EM	c_prime_0	-0.237 [-0.366, -0.147]*	Mean MD in posterior thalamic radiation on FA skeleton (right)
EM	total_0	-0.25 [-0.352, -0.146]*	Mean MD in posterior thalamic radiation on FA skeleton (right)
EM	beta1_1	0.179 [0.0191, 0.388]*	Mean MD in posterior thalamic radiation on FA skeleton (right)
EM	beta4_1	0.198 [0.0315, 0.373]*	Mean MD in posterior thalamic radiation on FA skeleton (right)
EM	beta3_1	-0.242 [-0.377, -0.0895]*	Mean MD in posterior thalamic radiation on FA skeleton (right)
EM	ind3_1	-0.0468 [-0.11, -8.800e-03]*	Mean MD in posterior thalamic radiation on FA skeleton (right)
EM	ind_1	-0.0663 [-0.17, -0.0153]*	Mean MD in posterior thalamic radiation on FA skeleton (right)
EM	total_1	-0.204 [-0.332, -0.0676]*	Mean MD in posterior thalamic radiation on FA skeleton (right)
EM	beta1_2	0.206 [0.0214, 0.399]*	Mean MD in posterior thalamic radiation on FA skeleton (right)
EM	beta4_2	0.222 [0.0788, 0.362]*	Mean MD in posterior thalamic radiation on FA skeleton (right)
EM	diff_beta3_2_0	0.275 [6.871e-03, 0.445]*	Mean MD in posterior thalamic radiation on FA skeleton (right)
EM	diff_beta3_2_1	0.276 [0.0479, 0.468]*	Mean MD in posterior thalamic radiation on FA skeleton (right)
EM	diff_beta4_2_0	0.22 [0.0157, 0.446]*	Mean MD in posterior thalamic radiation on FA skeleton (right)
ISP	beta1_0	0.213 [0.0157, 0.61]*	Mean MD in posterior thalamic radiation on FA skeleton (right)
ISP	beta3_0	-0.141 [-0.27, -0.0113]*	Mean MD in posterior thalamic radiation on FA skeleton (right)
ISP	c_prime_0	-0.181 [-0.31, -0.0105]*	Mean MD in posterior thalamic radiation on FA skeleton (right)
ISP	total_0	-0.169 [-0.307, -7.943e-03]*	Mean MD in posterior thalamic radiation on FA skeleton (right)
ISP	beta1_1	0.179 [0.0191, 0.388]*	Mean MD in posterior thalamic radiation on FA skeleton (right)
ISP	beta4_1	0.198 [0.0315, 0.373]*	Mean MD in posterior thalamic radiation on FA skeleton (right)
ISP	c_prime_1	-0.403 [-0.517, -0.253]*	Mean MD in posterior thalamic radiation on FA skeleton (right)
ISP	total_1	-0.399 [-0.514, -0.25]*	Mean MD in posterior thalamic radiation on FA skeleton (right)
ISP	beta1_2	0.206 [0.0214, 0.399]*	Mean MD in posterior thalamic radiation on FA skeleton (right)
ISP	beta4_2	0.222 [0.0788, 0.362]*	Mean MD in posterior thalamic radiation on FA skeleton (right)
ISP	c_prime_2	-0.287 [-0.419, -0.112]*	Mean MD in posterior thalamic radiation on FA skeleton (right)
ISP	total_2	-0.267 [-0.389, -0.118]*	Mean MD in posterior thalamic radiation on FA skeleton (right)
ISP	diff_beta4_2_0	0.22 [0.0157, 0.446]*	Mean MD in posterior thalamic radiation on FA skeleton (right)
ISP	diff_c_prime_1_0	-0.222 [-0.422, -0.0265]*	Mean MD in posterior thalamic radiation on FA skeleton (right)
ISP	diff_total_1_0	-0.23 [-0.426, -0.0261]*	Mean MD in posterior thalamic radiation on FA skeleton (right)
LF	beta1_0	0.213 [0.0157, 0.61]*	Mean MD in posterior thalamic radiation on FA skeleton (right)
LF	beta3_0	-0.17 [-0.303, -0.0203]*	Mean MD in posterior thalamic radiation on FA skeleton (right)
LF	c_prime_0	-0.233 [-0.371, -0.0907]*	Mean MD in posterior thalamic radiation on FA skeleton (right)
LF	total_0	-0.233 [-0.359, -0.0909]*	Mean MD in posterior thalamic radiation on FA skeleton (right)
LF	beta1_1	0.179 [0.0191, 0.388]*	Mean MD in posterior thalamic radiation on FA skeleton (right)
LF	beta4_1	0.198 [0.0315, 0.373]*	Mean MD in posterior thalamic radiation on FA skeleton (right)
LF	c_prime_1	-0.228 [-0.369, -0.0966]*	Mean MD in posterior thalamic radiation on FA skeleton (right)
LF	total_1	-0.23 [-0.369, -0.1]*	Mean MD in posterior thalamic radiation on FA skeleton (right)
LF	beta1_2	0.206 [0.0214, 0.399]*	Mean MD in posterior thalamic radiation on FA skeleton (right)
LF	beta4_2	0.222 [0.0788, 0.362]*	Mean MD in posterior thalamic radiation on FA skeleton (right)
LF	c_prime_2	-0.295 [-0.439, -0.111]*	Mean MD in posterior thalamic radiation on FA skeleton (right)
LF	total_2	-0.294 [-0.436, -0.133]*	Mean MD in posterior thalamic radiation on FA skeleton (right)
LF	diff_beta4_2_0	0.22 [0.0157, 0.446]*	Mean MD in posterior thalamic radiation on FA skeleton (right)
EF	beta1_0	0.213 [0.0157, 0.61]*	Mean MD in posterior thalamic radiation on FA skeleton (right)
EF	beta3_0	-0.191 [-0.331, -0.0702]*	Mean MD in posterior thalamic radiation on FA skeleton (right)
EF	beta1_1	0.179 [0.0191, 0.388]*	Mean MD in posterior thalamic radiation on FA skeleton (right)
EF	beta4_1	0.198 [0.0315, 0.373]*	Mean MD in posterior thalamic radiation on FA skeleton (right)
EF	c_prime_1	-0.342 [-0.468, -0.19]*	Mean MD in posterior thalamic radiation on FA skeleton (right)
EF	total_1	-0.36 [-0.494, -0.207]*	Mean MD in posterior thalamic radiation on FA skeleton (right)
EF	beta1_2	0.206 [0.0214, 0.399]*	Mean MD in posterior thalamic radiation on FA skeleton (right)
EF	beta4_2	0.222 [0.0788, 0.362]*	Mean MD in posterior thalamic radiation on FA skeleton (right)
EF	c_prime_2	-0.245 [-0.395, -0.0161]*	Mean MD in posterior thalamic radiation on FA skeleton (right)
EF	total_2	-0.252 [-0.395, -0.0436]*	Mean MD in posterior thalamic radiation on FA skeleton (right)
EF	diff_beta4_2_0	0.22 [0.0157, 0.446]*	Mean MD in posterior thalamic radiation on FA skeleton (right)
EF	diff_c_prime_1_0	-0.285 [-0.59, -0.0655]*	Mean MD in posterior thalamic radiation on FA skeleton (right)
EF	diff_total_1_0	-0.31 [-0.606, -0.0808]*	Mean MD in posterior thalamic radiation on FA skeleton (right)
VS	beta1_0	0.213 [0.0157, 0.61]*	Mean MD in posterior thalamic radiation on FA skeleton (right)
VS	beta3_0	-0.213 [-0.357, -0.0608]*	Mean MD in posterior thalamic radiation on FA skeleton (right)
VS	c_prime_0	-0.245 [-0.427, -0.0721]*	Mean MD in posterior thalamic radiation on FA skeleton (right)
VS	total_0	-0.223 [-0.396, -0.0501]*	Mean MD in posterior thalamic radiation on FA skeleton (right)
VS	beta1_1	0.179 [0.0191, 0.388]*	Mean MD in posterior thalamic radiation on FA skeleton (right)
VS	beta4_1	0.198 [0.0315, 0.373]*	Mean MD in posterior thalamic radiation on FA skeleton (right)
VS	beta3_1	-0.199 [-0.348, -0.0241]*	Mean MD in posterior thalamic radiation on FA skeleton (right)
VS	ind3_1	-0.0399 [-0.102, -3.078e-03]*	Mean MD in posterior thalamic radiation on FA skeleton (right)
VS	beta1_2	0.206 [0.0214, 0.399]*	Mean MD in posterior thalamic radiation on FA skeleton (right)
VS	beta4_2	0.222 [0.0788, 0.362]*	Mean MD in posterior thalamic radiation on FA skeleton (right)
VS	c_prime_2	-0.302 [-0.476, -0.1]*	Mean MD in posterior thalamic radiation on FA skeleton (right)
VS	total_2	-0.292 [-0.475, -0.112]*	Mean MD in posterior thalamic radiation on FA skeleton (right)
VS	diff_beta3_2_0	0.244 [0.0099, 0.427]*	Mean MD in posterior thalamic radiation on FA skeleton (right)
VS	diff_beta4_2_0	0.22 [0.0157, 0.446]*	Mean MD in posterior thalamic radiation on FA skeleton (right)
MMSE	beta1_0	0.213 [0.0157, 0.61]*	Mean MD in posterior thalamic radiation on FA skeleton (left)
MMSE	beta3_0	-0.199 [-0.442, -0.0129]*	Mean MD in posterior thalamic radiation on FA skeleton (left)
MMSE	c_prime_0	-0.317 [-0.523, -0.137]*	Mean MD in posterior thalamic radiation on FA skeleton (left)
MMSE	total_0	-0.287 [-0.498, -0.1]*	Mean MD in posterior thalamic radiation on FA skeleton (left)
MMSE	beta1_1	0.179 [0.0191, 0.388]*	Mean MD in posterior thalamic radiation on FA skeleton (left)
MMSE	c_prime_1	-0.505 [-0.69, -0.241]*	Mean MD in posterior thalamic radiation on FA skeleton (left)
MMSE	total_1	-0.533 [-0.705, -0.268]*	Mean MD in posterior thalamic radiation on FA skeleton (left)
MMSE	beta1_2	0.206 [0.0214, 0.399]*	Mean MD in posterior thalamic radiation on FA skeleton (left)
MMSE	beta4_2	0.201 [0.0878, 0.335]*	Mean MD in posterior thalamic radiation on FA skeleton (left)
MMSE	total_2	-0.262 [-0.469, -0.0361]*	Mean MD in posterior thalamic radiation on FA skeleton (left)
MMSE	diff_beta4_2_0	0.205 [0.0567, 0.436]*	Mean MD in posterior thalamic radiation on FA skeleton (left)

MoCA	beta1_0	0.213 [0.0157, 0.61]*	Mean MD in posterior thalamic radiation on FA skeleton (left)
MoCA	beta3_0	-0.243 [-0.39, -0.108]*	Mean MD in posterior thalamic radiation on FA skeleton (left)
MoCA	c_prime_0	-0.236 [-0.381, -0.0839]*	Mean MD in posterior thalamic radiation on FA skeleton (left)
MoCA	total_0	-0.22 [-0.363, -0.0657]*	Mean MD in posterior thalamic radiation on FA skeleton (left)
MoCA	beta1_1	0.179 [0.0191, 0.388]*	Mean MD in posterior thalamic radiation on FA skeleton (left)
MoCA	c_prime_1	-0.448 [-0.6, -0.206]*	Mean MD in posterior thalamic radiation on FA skeleton (left)
MoCA	total_1	-0.472 [-0.612, -0.227]*	Mean MD in posterior thalamic radiation on FA skeleton (left)
MoCA	beta1_2	0.206 [0.0214, 0.399]*	Mean MD in posterior thalamic radiation on FA skeleton (left)
MoCA	beta4_2	0.201 [0.0878, 0.335]*	Mean MD in posterior thalamic radiation on FA skeleton (left)
MoCA	c_prime_2	-0.328 [-0.492, -0.135]*	Mean MD in posterior thalamic radiation on FA skeleton (left)
MoCA	total_2	-0.361 [-0.526, -0.184]*	Mean MD in posterior thalamic radiation on FA skeleton (left)
MoCA	diff_beta4_2_0	0.205 [0.0567, 0.436]*	Mean MD in posterior thalamic radiation on FA skeleton (left)
EM	beta1_0	0.213 [0.0157, 0.61]*	Mean MD in posterior thalamic radiation on FA skeleton (left)
EM	c_prime_0	-0.236 [-0.37, -0.148]*	Mean MD in posterior thalamic radiation on FA skeleton (left)
EM	total_0	-0.25 [-0.352, -0.146]*	Mean MD in posterior thalamic radiation on FA skeleton (left)
EM	beta1_1	0.179 [0.0191, 0.388]*	Mean MD in posterior thalamic radiation on FA skeleton (left)
EM	beta3_1	-0.217 [-0.37, -0.0456]*	Mean MD in posterior thalamic radiation on FA skeleton (left)
EM	c_prime_1	-0.16 [-0.305, -5.381e-03]*	Mean MD in posterior thalamic radiation on FA skeleton (left)
EM	total_1	-0.204 [-0.332, -0.0676]*	Mean MD in posterior thalamic radiation on FA skeleton (left)
EM	beta1_2	0.206 [0.0214, 0.399]*	Mean MD in posterior thalamic radiation on FA skeleton (left)
EM	beta4_2	0.201 [0.0878, 0.335]*	Mean MD in posterior thalamic radiation on FA skeleton (left)
EM	diff_beta4_2_0	0.205 [0.0567, 0.436]*	Mean MD in posterior thalamic radiation on FA skeleton (left)
ISP	beta1_0	0.213 [0.0157, 0.61]*	Mean MD in posterior thalamic radiation on FA skeleton (left)
ISP	c_prime_0	-0.182 [-0.312, -0.0115]*	Mean MD in posterior thalamic radiation on FA skeleton (left)
ISP	total_0	-0.169 [-0.307, -7.943e-03]*	Mean MD in posterior thalamic radiation on FA skeleton (left)
ISP	beta1_1	0.179 [0.0191, 0.388]*	Mean MD in posterior thalamic radiation on FA skeleton (left)
ISP	c_prime_1	-0.4 [-0.515, -0.253]*	Mean MD in posterior thalamic radiation on FA skeleton (left)
ISP	total_1	-0.399 [-0.514, -0.25]*	Mean MD in posterior thalamic radiation on FA skeleton (left)
ISP	beta1_2	0.206 [0.0214, 0.399]*	Mean MD in posterior thalamic radiation on FA skeleton (left)
ISP	beta4_2	0.201 [0.0878, 0.335]*	Mean MD in posterior thalamic radiation on FA skeleton (left)
ISP	c_prime_2	-0.261 [-0.384, -0.0938]*	Mean MD in posterior thalamic radiation on FA skeleton (left)
ISP	ind3_2	-0.0288 [-0.094, -4.039e-03]*	Mean MD in posterior thalamic radiation on FA skeleton (left)
ISP	total_2	-0.267 [-0.389, -0.118]*	Mean MD in posterior thalamic radiation on FA skeleton (left)
ISP	diff_beta4_2_0	0.205 [0.0567, 0.436]*	Mean MD in posterior thalamic radiation on FA skeleton (left)
ISP	diff_c_prime_1_0	-0.219 [-0.421, -0.0254]*	Mean MD in posterior thalamic radiation on FA skeleton (left)
ISP	diff_total_1_0	-0.23 [-0.426, -0.0261]*	Mean MD in posterior thalamic radiation on FA skeleton (left)
LF	beta1_0	0.213 [0.0157, 0.61]*	Mean MD in posterior thalamic radiation on FA skeleton (left)
LF	beta3_0	-0.174 [-0.302, -1.452e-03]*	Mean MD in posterior thalamic radiation on FA skeleton (left)
LF	c_prime_0	-0.233 [-0.374, -0.0902]*	Mean MD in posterior thalamic radiation on FA skeleton (left)
LF	total_0	-0.233 [-0.359, -0.0909]*	Mean MD in posterior thalamic radiation on FA skeleton (left)
LF	beta1_1	0.179 [0.0191, 0.388]*	Mean MD in posterior thalamic radiation on FA skeleton (left)
LF	c_prime_1	-0.234 [-0.369, -0.0979]*	Mean MD in posterior thalamic radiation on FA skeleton (left)
LF	total_1	-0.23 [-0.369, -0.1]*	Mean MD in posterior thalamic radiation on FA skeleton (left)
LF	beta1_2	0.206 [0.0214, 0.399]*	Mean MD in posterior thalamic radiation on FA skeleton (left)
LF	beta4_2	0.201 [0.0878, 0.335]*	Mean MD in posterior thalamic radiation on FA skeleton (left)
LF	c_prime_2	-0.298 [-0.449, -0.12]*	Mean MD in posterior thalamic radiation on FA skeleton (left)
LF	total_2	-0.294 [-0.436, -0.133]*	Mean MD in posterior thalamic radiation on FA skeleton (left)
LF	diff_beta4_2_0	0.205 [0.0567, 0.436]*	Mean MD in posterior thalamic radiation on FA skeleton (left)
EF	beta1_0	0.213 [0.0157, 0.61]*	Mean MD in posterior thalamic radiation on FA skeleton (left)
EF	beta3_0	-0.134 [-0.243, -3.416e-03]*	Mean MD in posterior thalamic radiation on FA skeleton (left)
EF	beta1_1	0.179 [0.0191, 0.388]*	Mean MD in posterior thalamic radiation on FA skeleton (left)
EF	beta3_1	-0.138 [-0.278, -7.360e-03]*	Mean MD in posterior thalamic radiation on FA skeleton (left)
EF	c_prime_1	-0.339 [-0.462, -0.184]*	Mean MD in posterior thalamic radiation on FA skeleton (left)
EF	total_1	-0.36 [-0.494, -0.207]*	Mean MD in posterior thalamic radiation on FA skeleton (left)
EF	beta1_2	0.206 [0.0214, 0.399]*	Mean MD in posterior thalamic radiation on FA skeleton (left)
EF	beta4_2	0.201 [0.0878, 0.335]*	Mean MD in posterior thalamic radiation on FA skeleton (left)
EF	beta3_2	-0.166 [-0.324, -0.0208]*	Mean MD in posterior thalamic radiation on FA skeleton (left)
EF	ind3_2	-0.0339 [-0.0982, -7.337e-03]*	Mean MD in posterior thalamic radiation on FA skeleton (left)
EF	ind_2	-0.0331 [-0.115, -2.244e-03]*	Mean MD in posterior thalamic radiation on FA skeleton (left)
EF	total_2	-0.252 [-0.395, -0.0436]*	Mean MD in posterior thalamic radiation on FA skeleton (left)
EF	diff_beta4_2_0	0.205 [0.0567, 0.436]*	Mean MD in posterior thalamic radiation on FA skeleton (left)
EF	diff_ind3_2_0	-0.0324 [-0.106, -5.210e-03]*	Mean MD in posterior thalamic radiation on FA skeleton (left)
EF	diff_ind_2_0	-0.0395 [-0.136, -9.113e-04]*	Mean MD in posterior thalamic radiation on FA skeleton (left)
EF	diff_c_prime_1_0	-0.282 [-0.594, -0.0625]*	Mean MD in posterior thalamic radiation on FA skeleton (left)
EF	diff_total_1_0	-0.31 [-0.606, -0.0808]*	Mean MD in posterior thalamic radiation on FA skeleton (left)
VS	beta1_0	0.213 [0.0157, 0.61]*	Mean MD in posterior thalamic radiation on FA skeleton (left)
VS	c_prime_0	-0.245 [-0.431, -0.0717]*	Mean MD in posterior thalamic radiation on FA skeleton (left)
VS	total_0	-0.223 [-0.396, -0.0501]*	Mean MD in posterior thalamic radiation on FA skeleton (left)
VS	beta1_1	0.179 [0.0191, 0.388]*	Mean MD in posterior thalamic radiation on FA skeleton (left)
VS	beta3_1	-0.157 [-0.301, -0.0179]*	Mean MD in posterior thalamic radiation on FA skeleton (left)
VS	c_prime_1	-0.185 [-0.363, -2.920e-03]*	Mean MD in posterior thalamic radiation on FA skeleton (left)
VS	beta1_2	0.206 [0.0214, 0.399]*	Mean MD in posterior thalamic radiation on FA skeleton (left)
VS	beta4_2	0.201 [0.0878, 0.335]*	Mean MD in posterior thalamic radiation on FA skeleton (left)
VS	c_prime_2	-0.306 [-0.489, -0.102]*	Mean MD in posterior thalamic radiation on FA skeleton (left)
VS	total_2	-0.292 [-0.475, -0.112]*	Mean MD in posterior thalamic radiation on FA skeleton (left)
VS	diff_beta3_2_1	0.209 [9.037e-04, 0.431]*	Mean MD in posterior thalamic radiation on FA skeleton (left)
VS	diff_beta4_2_0	0.205 [0.0567, 0.436]*	Mean MD in posterior thalamic radiation on FA skeleton (left)
MMSE	beta1_0	0.213 [0.0157, 0.61]*	Mean MD in sagittal stratum on FA skeleton (right)
MMSE	beta3_0	-0.323 [-0.531, -0.121]*	Mean MD in sagittal stratum on FA skeleton (right)
MMSE	c_prime_0	-0.327 [-0.51, -0.154]*	Mean MD in sagittal stratum on FA skeleton (right)
MMSE	total_0	-0.287 [-0.498, -0.1]*	Mean MD in sagittal stratum on FA skeleton (right)
MMSE	beta1_1	0.179 [0.0191, 0.388]*	Mean MD in sagittal stratum on FA skeleton (right)
MMSE	beta4_1	0.256 [0.0339, 0.421]*	Mean MD in sagittal stratum on FA skeleton (right)
MMSE	c_prime_1	-0.487 [-0.656, -0.229]*	Mean MD in sagittal stratum on FA skeleton (right)

MMSE	ind3_1	-0.0336 [-0.106, -6.975e-04]*	Mean MD in sagittal stratum on FA skeleton (right)
MMSE	ind_1	-0.0468 [-0.127, -6.372e-03]*	Mean MD in sagittal stratum on FA skeleton (right)
MMSE	total_1	-0.533 [-0.705, -0.268]*	Mean MD in sagittal stratum on FA skeleton (right)
MMSE	beta1_2	0.206 [0.0214, 0.399]*	Mean MD in sagittal stratum on FA skeleton (right)
MMSE	beta2_2	0.338 [0.111, 0.62]*	Mean MD in sagittal stratum on FA skeleton (right)
MMSE	beta3_2	-0.211 [-0.444, -0.0379]*	Mean MD in sagittal stratum on FA skeleton (right)
MMSE	ind1_2	-0.0138 [-0.069, -2.645e-03]*	Mean MD in sagittal stratum on FA skeleton (right)
MMSE	ind3_2	-0.0288 [-0.0894, -8.790e-04]*	Mean MD in sagittal stratum on FA skeleton (right)
MMSE	ind_2	-0.046 [-0.139, -3.210e-03]*	Mean MD in sagittal stratum on FA skeleton (right)
MMSE	total_2	-0.262 [-0.469, -0.0361]*	Mean MD in sagittal stratum on FA skeleton (right)
MMSE	diff_beta2_2_0	0.285 [0.0536, 0.715]*	Mean MD in sagittal stratum on FA skeleton (right)
MMSE	diff_beta2_2_1	0.341 [0.0569, 0.691]*	Mean MD in sagittal stratum on FA skeleton (right)
MMSE	diff_beta4_1_0	0.286 [0.028, 0.52]*	Mean MD in sagittal stratum on FA skeleton (right)
MMSE	diff_beta5_1_0	-0.16 [-0.491, -3.522e-03]*	Mean MD in sagittal stratum on FA skeleton (right)
MMSE	diff_ind1_2_1	-0.0133 [-0.0683, -2.076e-03]*	Mean MD in sagittal stratum on FA skeleton (right)
MMSE	diff_ind_1_0	-0.0871 [-0.212, -4.501e-03]*	Mean MD in sagittal stratum on FA skeleton (right)
MMSE	diff_ind_2_0	-0.0863 [-0.226, -5.207e-03]*	Mean MD in sagittal stratum on FA skeleton (right)
MoCA	beta1_0	0.213 [0.0157, 0.61]*	Mean MD in sagittal stratum on FA skeleton (right)
MoCA	beta3_0	-0.284 [-0.416, -0.139]*	Mean MD in sagittal stratum on FA skeleton (right)
MoCA	c_prime_0	-0.245 [-0.376, -0.0854]*	Mean MD in sagittal stratum on FA skeleton (right)
MoCA	total_0	-0.22 [-0.363, -0.0657]*	Mean MD in sagittal stratum on FA skeleton (right)
MoCA	beta1_1	0.179 [0.0191, 0.388]*	Mean MD in sagittal stratum on FA skeleton (right)
MoCA	beta4_1	0.256 [0.0339, 0.421]*	Mean MD in sagittal stratum on FA skeleton (right)
MoCA	beta3_1	-0.186 [-0.326, -0.0491]*	Mean MD in sagittal stratum on FA skeleton (right)
MoCA	c_prime_1	-0.42 [-0.559, -0.189]*	Mean MD in sagittal stratum on FA skeleton (right)
MoCA	ind3_1	-0.045 [-0.1, -8.273e-03]*	Mean MD in sagittal stratum on FA skeleton (right)
MoCA	ind_1	-0.0526 [-0.119, -0.01]*	Mean MD in sagittal stratum on FA skeleton (right)
MoCA	total_1	-0.472 [-0.612, -0.227]*	Mean MD in sagittal stratum on FA skeleton (right)
MoCA	beta1_2	0.206 [0.0214, 0.399]*	Mean MD in sagittal stratum on FA skeleton (right)
MoCA	beta2_2	0.338 [0.111, 0.62]*	Mean MD in sagittal stratum on FA skeleton (right)
MoCA	c_prime_2	-0.333 [-0.488, -0.135]*	Mean MD in sagittal stratum on FA skeleton (right)
MoCA	ind1_2	-7.518e-03 [-0.0441, -3.588e-04]*	Mean MD in sagittal stratum on FA skeleton (right)
MoCA	total_2	-0.361 [-0.526, -0.184]*	Mean MD in sagittal stratum on FA skeleton (right)
MoCA	diff_beta2_2_0	0.285 [0.0536, 0.715]*	Mean MD in sagittal stratum on FA skeleton (right)
MoCA	diff_beta2_2_1	0.341 [0.0569, 0.691]*	Mean MD in sagittal stratum on FA skeleton (right)
MoCA	diff_beta4_1_0	0.286 [0.028, 0.52]*	Mean MD in sagittal stratum on FA skeleton (right)
MoCA	diff_ind3_1_0	-0.0551 [-0.132, -3.058e-04]*	Mean MD in sagittal stratum on FA skeleton (right)
MoCA	diff_ind_1_0	-0.0784 [-0.171, -0.0103]*	Mean MD in sagittal stratum on FA skeleton (right)
EM	beta1_0	0.213 [0.0157, 0.61]*	Mean MD in sagittal stratum on FA skeleton (right)
EM	beta3_0	-0.172 [-0.306, -0.0103]*	Mean MD in sagittal stratum on FA skeleton (right)
EM	c_prime_0	-0.243 [-0.367, -0.153]*	Mean MD in sagittal stratum on FA skeleton (right)
EM	total_0	-0.25 [-0.352, -0.146]*	Mean MD in sagittal stratum on FA skeleton (right)
EM	beta1_1	0.179 [0.0191, 0.388]*	Mean MD in sagittal stratum on FA skeleton (right)
EM	beta4_1	0.256 [0.0339, 0.421]*	Mean MD in sagittal stratum on FA skeleton (right)
EM	c_prime_1	-0.163 [-0.295, -7.829e-03]*	Mean MD in sagittal stratum on FA skeleton (right)
EM	total_1	-0.204 [-0.332, -0.0676]*	Mean MD in sagittal stratum on FA skeleton (right)
EM	beta1_2	0.206 [0.0214, 0.399]*	Mean MD in sagittal stratum on FA skeleton (right)
EM	beta2_2	0.338 [0.111, 0.62]*	Mean MD in sagittal stratum on FA skeleton (right)
EM	diff_beta2_2_0	0.285 [0.0536, 0.715]*	Mean MD in sagittal stratum on FA skeleton (right)
EM	diff_beta2_2_1	0.341 [0.0569, 0.691]*	Mean MD in sagittal stratum on FA skeleton (right)
EM	diff_beta4_1_0	0.286 [0.028, 0.52]*	Mean MD in sagittal stratum on FA skeleton (right)
ISP	beta1_0	0.213 [0.0157, 0.61]*	Mean MD in sagittal stratum on FA skeleton (right)
ISP	c_prime_0	-0.187 [-0.308, -3.982e-03]*	Mean MD in sagittal stratum on FA skeleton (right)
ISP	total_0	-0.169 [-0.307, -7.943e-03]*	Mean MD in sagittal stratum on FA skeleton (right)
ISP	beta1_1	0.179 [0.0191, 0.388]*	Mean MD in sagittal stratum on FA skeleton (right)
ISP	beta4_1	0.256 [0.0339, 0.421]*	Mean MD in sagittal stratum on FA skeleton (right)
ISP	c_prime_1	-0.412 [-0.533, -0.257]*	Mean MD in sagittal stratum on FA skeleton (right)
ISP	total_1	-0.399 [-0.514, -0.25]*	Mean MD in sagittal stratum on FA skeleton (right)
ISP	beta1_2	0.206 [0.0214, 0.399]*	Mean MD in sagittal stratum on FA skeleton (right)
ISP	beta2_2	0.338 [0.111, 0.62]*	Mean MD in sagittal stratum on FA skeleton (right)
ISP	c_prime_2	-0.29 [-0.425, -0.122]*	Mean MD in sagittal stratum on FA skeleton (right)
ISP	total_2	-0.267 [-0.389, -0.118]*	Mean MD in sagittal stratum on FA skeleton (right)
ISP	diff_beta2_2_0	0.285 [0.0536, 0.715]*	Mean MD in sagittal stratum on FA skeleton (right)
ISP	diff_beta2_2_1	0.341 [0.0569, 0.691]*	Mean MD in sagittal stratum on FA skeleton (right)
ISP	diff_beta4_1_0	0.286 [0.028, 0.52]*	Mean MD in sagittal stratum on FA skeleton (right)
ISP	diff_c_prime_1_0	-0.224 [-0.429, -0.0256]*	Mean MD in sagittal stratum on FA skeleton (right)
ISP	diff_total_1_0	-0.23 [-0.426, -0.0261]*	Mean MD in sagittal stratum on FA skeleton (right)
LF	beta1_0	0.213 [0.0157, 0.61]*	Mean MD in sagittal stratum on FA skeleton (right)
LF	c_prime_0	-0.239 [-0.372, -0.0818]*	Mean MD in sagittal stratum on FA skeleton (right)
LF	total_0	-0.233 [-0.359, -0.0909]*	Mean MD in sagittal stratum on FA skeleton (right)
LF	beta1_1	0.179 [0.0191, 0.388]*	Mean MD in sagittal stratum on FA skeleton (right)
LF	beta4_1	0.256 [0.0339, 0.421]*	Mean MD in sagittal stratum on FA skeleton (right)
LF	c_prime_1	-0.204 [-0.349, -0.0711]*	Mean MD in sagittal stratum on FA skeleton (right)
LF	ind3_1	-0.0376 [-0.0878, -3.773e-04]*	Mean MD in sagittal stratum on FA skeleton (right)
LF	total_1	-0.23 [-0.369, -0.1]*	Mean MD in sagittal stratum on FA skeleton (right)
LF	beta1_2	0.206 [0.0214, 0.399]*	Mean MD in sagittal stratum on FA skeleton (right)
LF	beta2_2	0.338 [0.111, 0.62]*	Mean MD in sagittal stratum on FA skeleton (right)
LF	c_prime_2	-0.296 [-0.439, -0.119]*	Mean MD in sagittal stratum on FA skeleton (right)
LF	total_2	-0.294 [-0.436, -0.133]*	Mean MD in sagittal stratum on FA skeleton (right)
LF	diff_beta2_2_0	0.285 [0.0536, 0.715]*	Mean MD in sagittal stratum on FA skeleton (right)
LF	diff_beta2_2_1	0.341 [0.0569, 0.691]*	Mean MD in sagittal stratum on FA skeleton (right)
LF	diff_beta4_1_0	0.286 [0.028, 0.52]*	Mean MD in sagittal stratum on FA skeleton (right)
EF	beta1_0	0.213 [0.0157, 0.61]*	Mean MD in sagittal stratum on FA skeleton (right)
EF	beta3_0	-0.157 [-0.283, -0.0305]*	Mean MD in sagittal stratum on FA skeleton (right)

EF	beta1_1	0.179 [0.0191, 0.388]*	Mean MD in sagittal stratum on FA skeleton (right)
EF	beta4_1	0.256 [0.0339, 0.421]*	Mean MD in sagittal stratum on FA skeleton (right)
EF	c_prime_1	-0.342 [-0.471, -0.186]*	Mean MD in sagittal stratum on FA skeleton (right)
EF	total_1	-0.36 [-0.494, -0.207]*	Mean MD in sagittal stratum on FA skeleton (right)
EF	beta1_2	0.206 [0.0214, 0.399]*	Mean MD in sagittal stratum on FA skeleton (right)
EF	beta2_2	0.338 [0.111, 0.62]*	Mean MD in sagittal stratum on FA skeleton (right)
EF	c_prime_2	-0.257 [-0.411, -0.0253]*	Mean MD in sagittal stratum on FA skeleton (right)
EF	total_2	-0.252 [-0.395, -0.0436]*	Mean MD in sagittal stratum on FA skeleton (right)
EF	diff_beta2_2_0	0.285 [0.0536, 0.715]*	Mean MD in sagittal stratum on FA skeleton (right)
EF	diff_beta2_2_1	0.341 [0.0569, 0.691]*	Mean MD in sagittal stratum on FA skeleton (right)
EF	diff_beta4_1_0	0.286 [0.028, 0.52]*	Mean MD in sagittal stratum on FA skeleton (right)
EF	diff_c_prime_1_0	-0.278 [-0.59, -0.0552]*	Mean MD in sagittal stratum on FA skeleton (right)
EF	diff_total_1_0	-0.31 [-0.606, -0.0808]*	Mean MD in sagittal stratum on FA skeleton (right)
VS	beta1_0	0.213 [0.0157, 0.61]*	Mean MD in sagittal stratum on FA skeleton (right)
VS	beta3_0	-0.185 [-0.326, -0.0257]*	Mean MD in sagittal stratum on FA skeleton (right)
VS	c_prime_0	-0.251 [-0.424, -0.0726]*	Mean MD in sagittal stratum on FA skeleton (right)
VS	total_0	-0.223 [-0.396, -0.0501]*	Mean MD in sagittal stratum on FA skeleton (right)
VS	beta1_1	0.179 [0.0191, 0.388]*	Mean MD in sagittal stratum on FA skeleton (right)
VS	beta4_1	0.256 [0.0339, 0.421]*	Mean MD in sagittal stratum on FA skeleton (right)
VS	beta3_1	-0.225 [-0.386, -0.0658]*	Mean MD in sagittal stratum on FA skeleton (right)
VS	ind3_1	-0.0564 [-0.121, -8.821e-03]*	Mean MD in sagittal stratum on FA skeleton (right)
VS	beta1_2	0.206 [0.0214, 0.399]*	Mean MD in sagittal stratum on FA skeleton (right)
VS	beta2_2	0.338 [0.111, 0.62]*	Mean MD in sagittal stratum on FA skeleton (right)
VS	c_prime_2	-0.293 [-0.469, -0.0917]*	Mean MD in sagittal stratum on FA skeleton (right)
VS	total_2	-0.292 [-0.475, -0.112]*	Mean MD in sagittal stratum on FA skeleton (right)
VS	diff_beta2_2_0	0.285 [0.0536, 0.715]*	Mean MD in sagittal stratum on FA skeleton (right)
VS	diff_beta2_2_1	0.341 [0.0569, 0.691]*	Mean MD in sagittal stratum on FA skeleton (right)
VS	diff_beta4_1_0	0.286 [0.028, 0.52]*	Mean MD in sagittal stratum on FA skeleton (right)
VS	diff_ind3_1_0	-0.0618 [-0.139, -7.710e-03]*	Mean MD in sagittal stratum on FA skeleton (right)
MMSE	beta1_0	0.213 [0.0157, 0.61]*	Mean MD in sagittal stratum on FA skeleton (left)
MMSE	beta3_0	-0.228 [-0.464, -0.042]*	Mean MD in sagittal stratum on FA skeleton (left)
MMSE	c_prime_0	-0.325 [-0.525, -0.146]*	Mean MD in sagittal stratum on FA skeleton (left)
MMSE	total_0	-0.287 [-0.498, -0.1]*	Mean MD in sagittal stratum on FA skeleton (left)
MMSE	beta1_1	0.179 [0.0191, 0.388]*	Mean MD in sagittal stratum on FA skeleton (left)
MMSE	beta4_1	0.235 [0.0534, 0.472]*	Mean MD in sagittal stratum on FA skeleton (left)
MMSE	beta3_1	-0.22 [-0.477, -0.05]*	Mean MD in sagittal stratum on FA skeleton (left)
MMSE	c_prime_1	-0.47 [-0.655, -0.205]*	Mean MD in sagittal stratum on FA skeleton (left)
MMSE	ind3_1	-0.0506 [-0.175, -9.052e-03]*	Mean MD in sagittal stratum on FA skeleton (left)
MMSE	ind_1	-0.0638 [-0.21, -9.408e-03]*	Mean MD in sagittal stratum on FA skeleton (left)
MMSE	total_1	-0.533 [-0.705, -0.268]*	Mean MD in sagittal stratum on FA skeleton (left)
MMSE	beta1_2	0.206 [0.0214, 0.399]*	Mean MD in sagittal stratum on FA skeleton (left)
MMSE	beta2_2	0.178 [8.466e-04, 0.39]*	Mean MD in sagittal stratum on FA skeleton (left)
MMSE	beta4_2	0.175 [0.0208, 0.307]*	Mean MD in sagittal stratum on FA skeleton (left)
MMSE	ind1_2	-6.508e-03 [-0.0412, -4.992e-04]*	Mean MD in sagittal stratum on FA skeleton (left)
MMSE	ind3_2	-0.0294 [-0.0963, -2.844e-03]*	Mean MD in sagittal stratum on FA skeleton (left)
MMSE	ind_2	-0.0465 [-0.15, -3.972e-03]*	Mean MD in sagittal stratum on FA skeleton (left)
MMSE	total_2	-0.262 [-0.469, -0.0361]*	Mean MD in sagittal stratum on FA skeleton (left)
MMSE	diff_beta4_1_0	0.283 [0.0686, 0.58]*	Mean MD in sagittal stratum on FA skeleton (left)
MMSE	diff_beta4_2_0	0.223 [0.0401, 0.447]*	Mean MD in sagittal stratum on FA skeleton (left)
MMSE	diff_ind3_1_0	-0.057 [-0.187, -0.0124]*	Mean MD in sagittal stratum on FA skeleton (left)
MMSE	diff_ind3_2_0	-0.0358 [-0.117, -4.393e-03]*	Mean MD in sagittal stratum on FA skeleton (left)
MMSE	diff_ind_1_0	-0.102 [-0.263, -0.0175]*	Mean MD in sagittal stratum on FA skeleton (left)
MMSE	diff_ind_2_0	-0.0844 [-0.236, -0.0152]*	Mean MD in sagittal stratum on FA skeleton (left)
MoCA	beta1_0	0.213 [0.0157, 0.61]*	Mean MD in sagittal stratum on FA skeleton (left)
MoCA	beta3_0	-0.263 [-0.406, -0.114]*	Mean MD in sagittal stratum on FA skeleton (left)
MoCA	c_prime_0	-0.246 [-0.391, -0.0966]*	Mean MD in sagittal stratum on FA skeleton (left)
MoCA	total_0	-0.22 [-0.363, -0.0657]*	Mean MD in sagittal stratum on FA skeleton (left)
MoCA	beta1_1	0.179 [0.0191, 0.388]*	Mean MD in sagittal stratum on FA skeleton (left)
MoCA	beta4_1	0.235 [0.0534, 0.472]*	Mean MD in sagittal stratum on FA skeleton (left)
MoCA	beta3_1	-0.23 [-0.412, -0.095]*	Mean MD in sagittal stratum on FA skeleton (left)
MoCA	c_prime_1	-0.412 [-0.562, -0.174]*	Mean MD in sagittal stratum on FA skeleton (left)
MoCA	ind3_1	-0.0526 [-0.143, -0.0132]*	Mean MD in sagittal stratum on FA skeleton (left)
MoCA	ind_1	-0.0602 [-0.179, -0.0101]*	Mean MD in sagittal stratum on FA skeleton (left)
MoCA	total_1	-0.472 [-0.612, -0.227]*	Mean MD in sagittal stratum on FA skeleton (left)
MoCA	beta1_2	0.206 [0.0214, 0.399]*	Mean MD in sagittal stratum on FA skeleton (left)
MoCA	beta2_2	0.178 [8.466e-04, 0.39]*	Mean MD in sagittal stratum on FA skeleton (left)
MoCA	beta4_2	0.175 [0.0208, 0.307]*	Mean MD in sagittal stratum on FA skeleton (left)
MoCA	c_prime_2	-0.322 [-0.474, -0.13]*	Mean MD in sagittal stratum on FA skeleton (left)
MoCA	ind1_2	-5.454e-03 [-0.0345, -2.950e-04]*	Mean MD in sagittal stratum on FA skeleton (left)
MoCA	ind3_2	-0.0252 [-0.0843, -1.707e-03]*	Mean MD in sagittal stratum on FA skeleton (left)
MoCA	ind_2	-0.0387 [-0.121, -6.484e-04]*	Mean MD in sagittal stratum on FA skeleton (left)
MoCA	total_2	-0.361 [-0.526, -0.184]*	Mean MD in sagittal stratum on FA skeleton (left)
MoCA	diff_beta4_1_0	0.283 [0.0686, 0.58]*	Mean MD in sagittal stratum on FA skeleton (left)
MoCA	diff_beta4_2_0	0.223 [0.0401, 0.447]*	Mean MD in sagittal stratum on FA skeleton (left)
MoCA	diff_ind3_1_0	-0.0638 [-0.162, -0.0135]*	Mean MD in sagittal stratum on FA skeleton (left)
MoCA	diff_ind3_2_0	-0.0364 [-0.113, -4.960e-04]*	Mean MD in sagittal stratum on FA skeleton (left)
MoCA	diff_ind_1_0	-0.0871 [-0.211, -0.0205]*	Mean MD in sagittal stratum on FA skeleton (left)
MoCA	diff_ind_2_0	-0.0656 [-0.177, -0.0136]*	Mean MD in sagittal stratum on FA skeleton (left)
EM	beta1_0	0.213 [0.0157, 0.61]*	Mean MD in sagittal stratum on FA skeleton (left)
EM	c_prime_0	-0.244 [-0.372, -0.155]*	Mean MD in sagittal stratum on FA skeleton (left)
EM	total_0	-0.25 [-0.352, -0.146]*	Mean MD in sagittal stratum on FA skeleton (left)
EM	beta1_1	0.179 [0.0191, 0.388]*	Mean MD in sagittal stratum on FA skeleton (left)
EM	beta4_1	0.235 [0.0534, 0.472]*	Mean MD in sagittal stratum on FA skeleton (left)
EM	ind_1	-0.0481 [-0.142, -2.760e-03]*	Mean MD in sagittal stratum on FA skeleton (left)

EM	total_1	-0.204 [-0.332, -0.0676]*	Mean MD in sagittal stratum on FA skeleton (left)
EM	beta1_2	0.206 [0.0214, 0.399]*	Mean MD in sagittal stratum on FA skeleton (left)
EM	beta2_2	0.178 [8.466e-04, 0.39]*	Mean MD in sagittal stratum on FA skeleton (left)
EM	beta4_2	0.175 [0.0208, 0.307]*	Mean MD in sagittal stratum on FA skeleton (left)
EM	diff_beta4_1_0	0.283 [0.0686, 0.58]*	Mean MD in sagittal stratum on FA skeleton (left)
EM	diff_beta4_2_0	0.223 [0.0401, 0.447]*	Mean MD in sagittal stratum on FA skeleton (left)
ISP	beta1_0	0.213 [0.0157, 0.61]*	Mean MD in sagittal stratum on FA skeleton (left)
ISP	c_prime_0	-0.187 [-0.316, -0.0121]*	Mean MD in sagittal stratum on FA skeleton (left)
ISP	total_0	-0.169 [-0.307, -7.943e-03]*	Mean MD in sagittal stratum on FA skeleton (left)
ISP	beta1_1	0.179 [0.0191, 0.388]*	Mean MD in sagittal stratum on FA skeleton (left)
ISP	beta4_1	0.235 [0.0534, 0.472]*	Mean MD in sagittal stratum on FA skeleton (left)
ISP	c_prime_1	-0.398 [-0.51, -0.245]*	Mean MD in sagittal stratum on FA skeleton (left)
ISP	total_1	-0.399 [-0.514, -0.25]*	Mean MD in sagittal stratum on FA skeleton (left)
ISP	beta1_2	0.206 [0.0214, 0.399]*	Mean MD in sagittal stratum on FA skeleton (left)
ISP	beta2_2	0.178 [8.466e-04, 0.39]*	Mean MD in sagittal stratum on FA skeleton (left)
ISP	beta4_2	0.175 [0.0208, 0.307]*	Mean MD in sagittal stratum on FA skeleton (left)
ISP	c_prime_2	-0.278 [-0.41, -0.117]*	Mean MD in sagittal stratum on FA skeleton (left)
ISP	total_2	-0.267 [-0.389, -0.118]*	Mean MD in sagittal stratum on FA skeleton (left)
ISP	diff_beta4_1_0	0.283 [0.0686, 0.58]*	Mean MD in sagittal stratum on FA skeleton (left)
ISP	diff_beta4_2_0	0.223 [0.0401, 0.447]*	Mean MD in sagittal stratum on FA skeleton (left)
ISP	diff_c_prime_1_0	-0.211 [-0.414, -0.0134]*	Mean MD in sagittal stratum on FA skeleton (left)
ISP	diff_total_1_0	-0.23 [-0.426, -0.0261]*	Mean MD in sagittal stratum on FA skeleton (left)
LF	beta1_0	0.213 [0.0157, 0.61]*	Mean MD in sagittal stratum on FA skeleton (left)
LF	c_prime_0	-0.24 [-0.375, -0.0891]*	Mean MD in sagittal stratum on FA skeleton (left)
LF	total_0	-0.233 [-0.359, -0.0909]*	Mean MD in sagittal stratum on FA skeleton (left)
LF	beta1_1	0.179 [0.0191, 0.388]*	Mean MD in sagittal stratum on FA skeleton (left)
LF	beta4_1	0.235 [0.0534, 0.472]*	Mean MD in sagittal stratum on FA skeleton (left)
LF	c_prime_1	-0.216 [-0.361, -0.0773]*	Mean MD in sagittal stratum on FA skeleton (left)
LF	total_1	-0.23 [-0.369, -0.1]*	Mean MD in sagittal stratum on FA skeleton (left)
LF	beta1_2	0.206 [0.0214, 0.399]*	Mean MD in sagittal stratum on FA skeleton (left)
LF	beta2_2	0.178 [8.466e-04, 0.39]*	Mean MD in sagittal stratum on FA skeleton (left)
LF	beta4_2	0.175 [0.0208, 0.307]*	Mean MD in sagittal stratum on FA skeleton (left)
LF	c_prime_2	-0.297 [-0.441, -0.118]*	Mean MD in sagittal stratum on FA skeleton (left)
LF	total_2	-0.294 [-0.436, -0.133]*	Mean MD in sagittal stratum on FA skeleton (left)
LF	diff_beta4_1_0	0.283 [0.0686, 0.58]*	Mean MD in sagittal stratum on FA skeleton (left)
LF	diff_beta4_2_0	0.223 [0.0401, 0.447]*	Mean MD in sagittal stratum on FA skeleton (left)
EF	beta1_0	0.213 [0.0157, 0.61]*	Mean MD in sagittal stratum on FA skeleton (left)
EF	beta3_0	-0.146 [-0.292, -6.628e-03]*	Mean MD in sagittal stratum on FA skeleton (left)
EF	beta1_1	0.179 [0.0191, 0.388]*	Mean MD in sagittal stratum on FA skeleton (left)
EF	beta4_1	0.235 [0.0534, 0.472]*	Mean MD in sagittal stratum on FA skeleton (left)
EF	beta3_1	-0.163 [-0.312, -0.0552]*	Mean MD in sagittal stratum on FA skeleton (left)
EF	c_prime_1	-0.316 [-0.436, -0.166]*	Mean MD in sagittal stratum on FA skeleton (left)
EF	ind3_1	-0.0394 [-0.121, -6.118e-03]*	Mean MD in sagittal stratum on FA skeleton (left)
EF	ind_1	-0.0436 [-0.15, -1.421e-03]*	Mean MD in sagittal stratum on FA skeleton (left)
EF	total_1	-0.36 [-0.494, -0.207]*	Mean MD in sagittal stratum on FA skeleton (left)
EF	beta1_2	0.206 [0.0214, 0.399]*	Mean MD in sagittal stratum on FA skeleton (left)
EF	beta2_2	0.178 [8.466e-04, 0.39]*	Mean MD in sagittal stratum on FA skeleton (left)
EF	beta4_2	0.175 [0.0208, 0.307]*	Mean MD in sagittal stratum on FA skeleton (left)
EF	c_prime_2	-0.237 [-0.393, -0.0154]*	Mean MD in sagittal stratum on FA skeleton (left)
EF	total_2	-0.252 [-0.395, -0.0436]*	Mean MD in sagittal stratum on FA skeleton (left)
EF	diff_beta4_1_0	0.283 [0.0686, 0.58]*	Mean MD in sagittal stratum on FA skeleton (left)
EF	diff_beta4_2_0	0.223 [0.0401, 0.447]*	Mean MD in sagittal stratum on FA skeleton (left)
EF	diff_ind3_1_0	-0.0443 [-0.131, -8.646e-03]*	Mean MD in sagittal stratum on FA skeleton (left)
EF	diff_ind_1_0	-0.0564 [-0.164, -5.673e-03]*	Mean MD in sagittal stratum on FA skeleton (left)
EF	diff_c_prime_1_0	-0.253 [-0.554, -0.0309]*	Mean MD in sagittal stratum on FA skeleton (left)
EF	diff_total_1_0	-0.31 [-0.606, -0.0808]*	Mean MD in sagittal stratum on FA skeleton (left)
VS	beta1_0	0.213 [0.0157, 0.61]*	Mean MD in sagittal stratum on FA skeleton (left)
VS	c_prime_0	-0.25 [-0.432, -0.0737]*	Mean MD in sagittal stratum on FA skeleton (left)
VS	total_0	-0.223 [-0.396, -0.0501]*	Mean MD in sagittal stratum on FA skeleton (left)
VS	beta1_1	0.179 [0.0191, 0.388]*	Mean MD in sagittal stratum on FA skeleton (left)
VS	beta4_1	0.235 [0.0534, 0.472]*	Mean MD in sagittal stratum on FA skeleton (left)
VS	beta3_1	-0.203 [-0.361, -0.0159]*	Mean MD in sagittal stratum on FA skeleton (left)
VS	ind3_1	-0.048 [-0.13, -4.202e-03]*	Mean MD in sagittal stratum on FA skeleton (left)
VS	beta1_2	0.206 [0.0214, 0.399]*	Mean MD in sagittal stratum on FA skeleton (left)
VS	beta2_2	0.178 [8.466e-04, 0.39]*	Mean MD in sagittal stratum on FA skeleton (left)
VS	beta4_2	0.175 [0.0208, 0.307]*	Mean MD in sagittal stratum on FA skeleton (left)
VS	c_prime_2	-0.291 [-0.476, -0.0875]*	Mean MD in sagittal stratum on FA skeleton (left)
VS	total_2	-0.292 [-0.475, -0.112]*	Mean MD in sagittal stratum on FA skeleton (left)
VS	diff_beta4_1_0	0.283 [0.0686, 0.58]*	Mean MD in sagittal stratum on FA skeleton (left)
VS	diff_beta4_2_0	0.223 [0.0401, 0.447]*	Mean MD in sagittal stratum on FA skeleton (left)
VS	diff_ind3_1_0	-0.0525 [-0.14, -6.241e-03]*	Mean MD in sagittal stratum on FA skeleton (left)
MMSE	beta1_0	0.213 [0.0157, 0.61]*	Mean MD in cingulum cingulate gyrus on FA skeleton (left)
MMSE	beta3_0	-0.226 [-0.427, -0.0818]*	Mean MD in cingulum cingulate gyrus on FA skeleton (left)
MMSE	c_prime_0	-0.338 [-0.535, -0.153]*	Mean MD in cingulum cingulate gyrus on FA skeleton (left)
MMSE	total_0	-0.287 [-0.498, -0.1]*	Mean MD in cingulum cingulate gyrus on FA skeleton (left)
MMSE	beta1_1	0.179 [0.0191, 0.388]*	Mean MD in cingulum cingulate gyrus on FA skeleton (left)
MMSE	beta4_1	0.178 [0.0209, 0.336]*	Mean MD in cingulum cingulate gyrus on FA skeleton (left)
MMSE	c_prime_1	-0.503 [-0.682, -0.228]*	Mean MD in cingulum cingulate gyrus on FA skeleton (left)
MMSE	total_1	-0.533 [-0.705, -0.268]*	Mean MD in cingulum cingulate gyrus on FA skeleton (left)
MMSE	beta1_2	0.206 [0.0214, 0.399]*	Mean MD in cingulum cingulate gyrus on FA skeleton (left)
MMSE	beta2_2	0.182 [0.0382, 0.326]*	Mean MD in cingulum cingulate gyrus on FA skeleton (left)
MMSE	beta4_2	0.226 [0.0507, 0.386]*	Mean MD in cingulum cingulate gyrus on FA skeleton (left)
MMSE	beta3_2	-0.182 [-0.36, -0.0554]*	Mean MD in cingulum cingulate gyrus on FA skeleton (left)
MMSE	ind1_2	-6.450e-03 [-0.0304, -1.454e-03]*	Mean MD in cingulum cingulate gyrus on FA skeleton (left)

MMSE	ind3_2	-0.0397 [-0.11, -0.0101]*	Mean MD in cingulum cingulate gyrus on FA skeleton (left)
MMSE	ind_2	-0.0568 [-0.163, -0.0149]*	Mean MD in cingulum cingulate gyrus on FA skeleton (left)
MMSE	total_2	-0.262 [-0.469, -0.0361]*	Mean MD in cingulum cingulate gyrus on FA skeleton (left)
MMSE	diff_beta4_1_0	0.269 [0.0357, 0.495]*	Mean MD in cingulum cingulate gyrus on FA skeleton (left)
MMSE	diff_beta4_2_0	0.317 [0.0668, 0.541]*	Mean MD in cingulum cingulate gyrus on FA skeleton (left)
MMSE	diff_ind3_2_0	-0.0595 [-0.144, -0.0148]*	Mean MD in cingulum cingulate gyrus on FA skeleton (left)
MMSE	diff_ind_1_0	-0.0812 [-0.195, -4.294e-03]*	Mean MD in cingulum cingulate gyrus on FA skeleton (left)
MMSE	diff_ind_2_0	-0.108 [-0.25, -0.0302]*	Mean MD in cingulum cingulate gyrus on FA skeleton (left)
MoCA	beta1_0	0.213 [0.0157, 0.61]*	Mean MD in cingulum cingulate gyrus on FA skeleton (left)
MoCA	beta3_0	-0.18 [-0.336, -0.0352]*	Mean MD in cingulum cingulate gyrus on FA skeleton (left)
MoCA	c_prime_0	-0.251 [-0.397, -0.0902]*	Mean MD in cingulum cingulate gyrus on FA skeleton (left)
MoCA	total_0	-0.22 [-0.363, -0.0657]*	Mean MD in cingulum cingulate gyrus on FA skeleton (left)
MoCA	beta1_1	0.179 [0.0191, 0.388]*	Mean MD in cingulum cingulate gyrus on FA skeleton (left)
MoCA	beta4_1	0.178 [0.0209, 0.336]*	Mean MD in cingulum cingulate gyrus on FA skeleton (left)
MoCA	beta3_1	-0.17 [-0.275, -0.0521]*	Mean MD in cingulum cingulate gyrus on FA skeleton (left)
MoCA	c_prime_1	-0.433 [-0.581, -0.184]*	Mean MD in cingulum cingulate gyrus on FA skeleton (left)
MoCA	ind3_1	-0.0314 [-0.0774, -2.858e-03]*	Mean MD in cingulum cingulate gyrus on FA skeleton (left)
MoCA	total_1	-0.472 [-0.612, -0.227]*	Mean MD in cingulum cingulate gyrus on FA skeleton (left)
MoCA	beta1_2	0.206 [0.0214, 0.399]*	Mean MD in cingulum cingulate gyrus on FA skeleton (left)
MoCA	beta2_2	0.182 [0.0382, 0.326]*	Mean MD in cingulum cingulate gyrus on FA skeleton (left)
MoCA	beta4_2	0.226 [0.0507, 0.386]*	Mean MD in cingulum cingulate gyrus on FA skeleton (left)
MoCA	c_prime_2	-0.329 [-0.493, -0.141]*	Mean MD in cingulum cingulate gyrus on FA skeleton (left)
MoCA	ind1_2	-2.847e-03 [-0.0188, -1.284e-04]*	Mean MD in cingulum cingulate gyrus on FA skeleton (left)
MoCA	total_2	-0.361 [-0.526, -0.184]*	Mean MD in cingulum cingulate gyrus on FA skeleton (left)
MoCA	diff_beta4_1_0	0.269 [0.0357, 0.495]*	Mean MD in cingulum cingulate gyrus on FA skeleton (left)
MoCA	diff_beta4_2_0	0.317 [0.0668, 0.541]*	Mean MD in cingulum cingulate gyrus on FA skeleton (left)
MoCA	diff_ind3_1_0	-0.0471 [-0.107, -3.519e-03]*	Mean MD in cingulum cingulate gyrus on FA skeleton (left)
MoCA	diff_ind_1_0	-0.0704 [-0.159, -8.256e-03]*	Mean MD in cingulum cingulate gyrus on FA skeleton (left)
MoCA	diff_ind_2_0	-0.063 [-0.17, -7.105e-03]*	Mean MD in cingulum cingulate gyrus on FA skeleton (left)
EM	beta1_0	0.213 [0.0157, 0.61]*	Mean MD in cingulum cingulate gyrus on FA skeleton (left)
EM	c_prime_0	-0.246 [-0.366, -0.151]*	Mean MD in cingulum cingulate gyrus on FA skeleton (left)
EM	total_0	-0.25 [-0.352, -0.146]*	Mean MD in cingulum cingulate gyrus on FA skeleton (left)
EM	beta1_1	0.179 [0.0191, 0.388]*	Mean MD in cingulum cingulate gyrus on FA skeleton (left)
EM	beta4_1	0.178 [0.0209, 0.336]*	Mean MD in cingulum cingulate gyrus on FA skeleton (left)
EM	beta3_1	-0.172 [-0.303, -0.0312]*	Mean MD in cingulum cingulate gyrus on FA skeleton (left)
EM	c_prime_1	-0.155 [-0.289, -2.588e-03]*	Mean MD in cingulum cingulate gyrus on FA skeleton (left)
EM	ind3_1	-0.0291 [-0.0766, -3.989e-03]*	Mean MD in cingulum cingulate gyrus on FA skeleton (left)
EM	ind_1	-0.0485 [-0.127, -8.712e-03]*	Mean MD in cingulum cingulate gyrus on FA skeleton (left)
EM	total_1	-0.204 [-0.332, -0.0676]*	Mean MD in cingulum cingulate gyrus on FA skeleton (left)
EM	beta1_2	0.206 [0.0214, 0.399]*	Mean MD in cingulum cingulate gyrus on FA skeleton (left)
EM	beta2_2	0.182 [0.0382, 0.326]*	Mean MD in cingulum cingulate gyrus on FA skeleton (left)
EM	beta4_2	0.226 [0.0507, 0.386]*	Mean MD in cingulum cingulate gyrus on FA skeleton (left)
EM	diff_beta4_1_0	0.269 [0.0357, 0.495]*	Mean MD in cingulum cingulate gyrus on FA skeleton (left)
EM	diff_beta4_2_0	0.317 [0.0668, 0.541]*	Mean MD in cingulum cingulate gyrus on FA skeleton (left)
EM	diff_ind3_1_0	-0.0348 [-0.0886, -4.392e-03]*	Mean MD in cingulum cingulate gyrus on FA skeleton (left)
ISP	beta1_0	0.213 [0.0157, 0.61]*	Mean MD in cingulum cingulate gyrus on FA skeleton (left)
ISP	c_prime_0	-0.184 [-0.317, -5.704e-03]*	Mean MD in cingulum cingulate gyrus on FA skeleton (left)
ISP	total_0	-0.169 [-0.307, -7.943e-03]*	Mean MD in cingulum cingulate gyrus on FA skeleton (left)
ISP	beta1_1	0.179 [0.0191, 0.388]*	Mean MD in cingulum cingulate gyrus on FA skeleton (left)
ISP	beta4_1	0.178 [0.0209, 0.336]*	Mean MD in cingulum cingulate gyrus on FA skeleton (left)
ISP	c_prime_1	-0.396 [-0.513, -0.246]*	Mean MD in cingulum cingulate gyrus on FA skeleton (left)
ISP	total_1	-0.399 [-0.514, -0.25]*	Mean MD in cingulum cingulate gyrus on FA skeleton (left)
ISP	beta1_2	0.206 [0.0214, 0.399]*	Mean MD in cingulum cingulate gyrus on FA skeleton (left)
ISP	beta2_2	0.182 [0.0382, 0.326]*	Mean MD in cingulum cingulate gyrus on FA skeleton (left)
ISP	beta4_2	0.226 [0.0507, 0.386]*	Mean MD in cingulum cingulate gyrus on FA skeleton (left)
ISP	c_prime_2	-0.288 [-0.432, -0.123]*	Mean MD in cingulum cingulate gyrus on FA skeleton (left)
ISP	total_2	-0.267 [-0.389, -0.118]*	Mean MD in cingulum cingulate gyrus on FA skeleton (left)
ISP	diff_beta4_1_0	0.269 [0.0357, 0.495]*	Mean MD in cingulum cingulate gyrus on FA skeleton (left)
ISP	diff_beta4_2_0	0.317 [0.0668, 0.541]*	Mean MD in cingulum cingulate gyrus on FA skeleton (left)
ISP	diff_c_prime_1_0	-0.212 [-0.422, -0.0179]*	Mean MD in cingulum cingulate gyrus on FA skeleton (left)
ISP	diff_total_1_0	-0.23 [-0.426, -0.0261]*	Mean MD in cingulum cingulate gyrus on FA skeleton (left)
LF	beta1_0	0.213 [0.0157, 0.61]*	Mean MD in cingulum cingulate gyrus on FA skeleton (left)
LF	c_prime_0	-0.227 [-0.369, -0.0669]*	Mean MD in cingulum cingulate gyrus on FA skeleton (left)
LF	total_0	-0.233 [-0.359, -0.0909]*	Mean MD in cingulum cingulate gyrus on FA skeleton (left)
LF	beta1_1	0.179 [0.0191, 0.388]*	Mean MD in cingulum cingulate gyrus on FA skeleton (left)
LF	beta4_1	0.178 [0.0209, 0.336]*	Mean MD in cingulum cingulate gyrus on FA skeleton (left)
LF	c_prime_1	-0.21 [-0.352, -0.0834]*	Mean MD in cingulum cingulate gyrus on FA skeleton (left)
LF	total_1	-0.23 [-0.369, -0.1]*	Mean MD in cingulum cingulate gyrus on FA skeleton (left)
LF	beta1_2	0.206 [0.0214, 0.399]*	Mean MD in cingulum cingulate gyrus on FA skeleton (left)
LF	beta2_2	0.182 [0.0382, 0.326]*	Mean MD in cingulum cingulate gyrus on FA skeleton (left)
LF	beta4_2	0.226 [0.0507, 0.386]*	Mean MD in cingulum cingulate gyrus on FA skeleton (left)
LF	c_prime_2	-0.28 [-0.426, -0.102]*	Mean MD in cingulum cingulate gyrus on FA skeleton (left)
LF	ind1_2	-4.619e-03 [-0.0237, -2.601e-04]*	Mean MD in cingulum cingulate gyrus on FA skeleton (left)
LF	total_2	-0.294 [-0.436, -0.133]*	Mean MD in cingulum cingulate gyrus on FA skeleton (left)
LF	diff_beta3_1_0	-0.243 [-0.422, -0.023]*	Mean MD in cingulum cingulate gyrus on FA skeleton (left)
LF	diff_beta3_2_0	-0.2 [-0.387, -0.0102]*	Mean MD in cingulum cingulate gyrus on FA skeleton (left)
LF	diff_beta4_1_0	0.269 [0.0357, 0.495]*	Mean MD in cingulum cingulate gyrus on FA skeleton (left)
LF	diff_beta4_2_0	0.317 [0.0668, 0.541]*	Mean MD in cingulum cingulate gyrus on FA skeleton (left)
LF	diff_ind1_2_0	-5.413e-03 [-0.0239, -5.212e-04]*	Mean MD in cingulum cingulate gyrus on FA skeleton (left)
EF	beta1_0	0.213 [0.0157, 0.61]*	Mean MD in cingulum cingulate gyrus on FA skeleton (left)
EF	beta1_1	0.179 [0.0191, 0.388]*	Mean MD in cingulum cingulate gyrus on FA skeleton (left)
EF	beta4_1	0.178 [0.0209, 0.336]*	Mean MD in cingulum cingulate gyrus on FA skeleton (left)
EF	c_prime_1	-0.337 [-0.466, -0.181]*	Mean MD in cingulum cingulate gyrus on FA skeleton (left)
EF	total_1	-0.36 [-0.494, -0.207]*	Mean MD in cingulum cingulate gyrus on FA skeleton (left)

EF	beta1_2	0.206 [0.0214, 0.399]*	Mean MD in cingulum cingulate gyrus on FA skeleton (left)
EF	beta2_2	0.182 [0.0382, 0.326]*	Mean MD in cingulum cingulate gyrus on FA skeleton (left)
EF	beta4_2	0.226 [0.0507, 0.386]*	Mean MD in cingulum cingulate gyrus on FA skeleton (left)
EF	c_prime_2	-0.253 [-0.415, -0.025]*	Mean MD in cingulum cingulate gyrus on FA skeleton (left)
EF	total_2	-0.252 [-0.395, -0.0436]*	Mean MD in cingulum cingulate gyrus on FA skeleton (left)
EF	diff_beta4_1_0	0.269 [0.0357, 0.495]*	Mean MD in cingulum cingulate gyrus on FA skeleton (left)
EF	diff_beta4_2_0	0.317 [0.0668, 0.541]*	Mean MD in cingulum cingulate gyrus on FA skeleton (left)
EF	diff_c_prime_1_0	-0.276 [-0.583, -0.0524]*	Mean MD in cingulum cingulate gyrus on FA skeleton (left)
EF	diff_total_1_0	-0.31 [-0.606, -0.0808]*	Mean MD in cingulum cingulate gyrus on FA skeleton (left)
VS	beta1_0	0.213 [0.0157, 0.61]*	Mean MD in cingulum cingulate gyrus on FA skeleton (left)
VS	c_prime_0	-0.253 [-0.437, -0.0708]*	Mean MD in cingulum cingulate gyrus on FA skeleton (left)
VS	total_0	-0.223 [-0.396, -0.0501]*	Mean MD in cingulum cingulate gyrus on FA skeleton (left)
VS	beta1_1	0.179 [0.0191, 0.388]*	Mean MD in cingulum cingulate gyrus on FA skeleton (left)
VS	beta4_1	0.178 [0.0209, 0.336]*	Mean MD in cingulum cingulate gyrus on FA skeleton (left)
VS	c_prime_1	-0.195 [-0.379, -3.148e-03]*	Mean MD in cingulum cingulate gyrus on FA skeleton (left)
VS	beta1_2	0.206 [0.0214, 0.399]*	Mean MD in cingulum cingulate gyrus on FA skeleton (left)
VS	beta2_2	0.182 [0.0382, 0.326]*	Mean MD in cingulum cingulate gyrus on FA skeleton (left)
VS	beta4_2	0.226 [0.0507, 0.386]*	Mean MD in cingulum cingulate gyrus on FA skeleton (left)
VS	c_prime_2	-0.283 [-0.465, -0.0884]*	Mean MD in cingulum cingulate gyrus on FA skeleton (left)
VS	total_2	-0.292 [-0.475, -0.112]*	Mean MD in cingulum cingulate gyrus on FA skeleton (left)
VS	diff_beta4_1_0	0.269 [0.0357, 0.495]*	Mean MD in cingulum cingulate gyrus on FA skeleton (left)
VS	diff_beta4_2_0	0.317 [0.0668, 0.541]*	Mean MD in cingulum cingulate gyrus on FA skeleton (left)
MMSE	beta1_0	0.213 [0.0157, 0.61]*	Mean MD in cingulum hippocampus on FA skeleton (left)
MMSE	beta3_0	-0.39 [-0.552, -0.239]*	Mean MD in cingulum hippocampus on FA skeleton (left)
MMSE	c_prime_0	-0.297 [-0.476, -0.123]*	Mean MD in cingulum hippocampus on FA skeleton (left)
MMSE	total_0	-0.287 [-0.498, -0.1]*	Mean MD in cingulum hippocampus on FA skeleton (left)
MMSE	beta1_1	0.179 [0.0191, 0.388]*	Mean MD in cingulum hippocampus on FA skeleton (left)
MMSE	beta3_1	-0.47 [-0.787, -0.273]*	Mean MD in cingulum hippocampus on FA skeleton (left)
MMSE	c_prime_1	-0.398 [-0.558, -0.189]*	Mean MD in cingulum hippocampus on FA skeleton (left)
MMSE	ind3_1	-0.123 [-0.295, -0.0314]*	Mean MD in cingulum hippocampus on FA skeleton (left)
MMSE	ind_1	-0.136 [-0.315, -0.0455]*	Mean MD in cingulum hippocampus on FA skeleton (left)
MMSE	total_1	-0.533 [-0.705, -0.268]*	Mean MD in cingulum hippocampus on FA skeleton (left)
MMSE	beta1_2	0.206 [0.0214, 0.399]*	Mean MD in cingulum hippocampus on FA skeleton (left)
MMSE	beta4_2	0.24 [0.0531, 0.459]*	Mean MD in cingulum hippocampus on FA skeleton (left)
MMSE	beta3_2	-0.286 [-0.507, -0.128]*	Mean MD in cingulum hippocampus on FA skeleton (left)
MMSE	ind3_2	-0.0668 [-0.16, -0.0165]*	Mean MD in cingulum hippocampus on FA skeleton (left)
MMSE	ind_2	-0.0839 [-0.204, -0.0241]*	Mean MD in cingulum hippocampus on FA skeleton (left)
MMSE	total_2	-0.262 [-0.469, -0.0361]*	Mean MD in cingulum hippocampus on FA skeleton (left)
MMSE	diff_ind_1_0	-0.146 [-0.335, -0.0237]*	Mean MD in cingulum hippocampus on FA skeleton (left)
MoCA	beta1_0	0.213 [0.0157, 0.61]*	Mean MD in cingulum hippocampus on FA skeleton (left)
MoCA	beta3_0	-0.394 [-0.514, -0.268]*	Mean MD in cingulum hippocampus on FA skeleton (left)
MoCA	c_prime_0	-0.213 [-0.349, -0.0684]*	Mean MD in cingulum hippocampus on FA skeleton (left)
MoCA	total_0	-0.22 [-0.363, -0.0657]*	Mean MD in cingulum hippocampus on FA skeleton (left)
MoCA	beta1_1	0.179 [0.0191, 0.388]*	Mean MD in cingulum hippocampus on FA skeleton (left)
MoCA	beta3_1	-0.406 [-0.675, -0.233]*	Mean MD in cingulum hippocampus on FA skeleton (left)
MoCA	c_prime_1	-0.359 [-0.502, -0.158]*	Mean MD in cingulum hippocampus on FA skeleton (left)
MoCA	ind3_1	-0.106 [-0.254, -0.0269]*	Mean MD in cingulum hippocampus on FA skeleton (left)
MoCA	ind_1	-0.113 [-0.263, -0.0321]*	Mean MD in cingulum hippocampus on FA skeleton (left)
MoCA	total_1	-0.472 [-0.612, -0.227]*	Mean MD in cingulum hippocampus on FA skeleton (left)
MoCA	beta1_2	0.206 [0.0214, 0.399]*	Mean MD in cingulum hippocampus on FA skeleton (left)
MoCA	beta4_2	0.24 [0.0531, 0.459]*	Mean MD in cingulum hippocampus on FA skeleton (left)
MoCA	beta3_2	-0.192 [-0.385, -0.0192]*	Mean MD in cingulum hippocampus on FA skeleton (left)
MoCA	c_prime_2	-0.297 [-0.445, -0.129]*	Mean MD in cingulum hippocampus on FA skeleton (left)
MoCA	ind3_2	-0.05 [-0.158, -3.564e-03]*	Mean MD in cingulum hippocampus on FA skeleton (left)
MoCA	ind_2	-0.0635 [-0.174, -3.671e-03]*	Mean MD in cingulum hippocampus on FA skeleton (left)
MoCA	total_2	-0.361 [-0.526, -0.184]*	Mean MD in cingulum hippocampus on FA skeleton (left)
MoCA	diff_ind_1_0	-0.107 [-0.275, -9.214e-03]*	Mean MD in cingulum hippocampus on FA skeleton (left)
EM	beta1_0	0.213 [0.0157, 0.61]*	Mean MD in cingulum hippocampus on FA skeleton (left)
EM	c_prime_0	-0.229 [-0.351, -0.131]*	Mean MD in cingulum hippocampus on FA skeleton (left)
EM	total_0	-0.25 [-0.352, -0.146]*	Mean MD in cingulum hippocampus on FA skeleton (left)
EM	beta1_1	0.179 [0.0191, 0.388]*	Mean MD in cingulum hippocampus on FA skeleton (left)
EM	c_prime_1	-0.166 [-0.304, -0.0244]*	Mean MD in cingulum hippocampus on FA skeleton (left)
EM	total_1	-0.204 [-0.332, -0.0676]*	Mean MD in cingulum hippocampus on FA skeleton (left)
EM	beta1_2	0.206 [0.0214, 0.399]*	Mean MD in cingulum hippocampus on FA skeleton (left)
EM	beta4_2	0.24 [0.0531, 0.459]*	Mean MD in cingulum hippocampus on FA skeleton (left)
ISP	beta1_0	0.213 [0.0157, 0.61]*	Mean MD in cingulum hippocampus on FA skeleton (left)
ISP	beta3_0	-0.236 [-0.368, -0.105]*	Mean MD in cingulum hippocampus on FA skeleton (left)
ISP	c_prime_0	-0.167 [-0.29, -1.743e-03]*	Mean MD in cingulum hippocampus on FA skeleton (left)
ISP	total_0	-0.169 [-0.307, -7.943e-03]*	Mean MD in cingulum hippocampus on FA skeleton (left)
ISP	beta1_1	0.179 [0.0191, 0.388]*	Mean MD in cingulum hippocampus on FA skeleton (left)
ISP	c_prime_1	-0.379 [-0.497, -0.238]*	Mean MD in cingulum hippocampus on FA skeleton (left)
ISP	total_1	-0.399 [-0.514, -0.25]*	Mean MD in cingulum hippocampus on FA skeleton (left)
ISP	beta1_2	0.206 [0.0214, 0.399]*	Mean MD in cingulum hippocampus on FA skeleton (left)
ISP	beta4_2	0.24 [0.0531, 0.459]*	Mean MD in cingulum hippocampus on FA skeleton (left)
ISP	beta3_2	-0.133 [-0.298, -0.011]*	Mean MD in cingulum hippocampus on FA skeleton (left)
ISP	c_prime_2	-0.258 [-0.397, -0.098]*	Mean MD in cingulum hippocampus on FA skeleton (left)
ISP	ind3_2	-0.032 [-0.108, -3.164e-03]*	Mean MD in cingulum hippocampus on FA skeleton (left)
ISP	total_2	-0.267 [-0.389, -0.118]*	Mean MD in cingulum hippocampus on FA skeleton (left)
ISP	diff_c_prime_1_0	-0.212 [-0.413, -0.0194]*	Mean MD in cingulum hippocampus on FA skeleton (left)
ISP	diff_total_1_0	-0.23 [-0.426, -0.0261]*	Mean MD in cingulum hippocampus on FA skeleton (left)
LF	beta1_0	0.213 [0.0157, 0.61]*	Mean MD in cingulum hippocampus on FA skeleton (left)
LF	beta3_0	-0.166 [-0.311, -0.0153]*	Mean MD in cingulum hippocampus on FA skeleton (left)
LF	c_prime_0	-0.225 [-0.359, -0.0762]*	Mean MD in cingulum hippocampus on FA skeleton (left)
LF	total_0	-0.233 [-0.359, -0.0909]*	Mean MD in cingulum hippocampus on FA skeleton (left)

LF	beta1_1	0.179 [0.0191, 0.388]*	Mean MD in cingulum hippocampus on FA skeleton (left)
LF	beta3_1	-0.235 [-0.392, -0.0343]*	Mean MD in cingulum hippocampus on FA skeleton (left)
LF	c_prime_1	-0.18 [-0.322, -0.0319]*	Mean MD in cingulum hippocampus on FA skeleton (left)
LF	ind3_1	-0.0617 [-0.157, -3.286e-03]*	Mean MD in cingulum hippocampus on FA skeleton (left)
LF	total_1	-0.23 [-0.369, -0.1]*	Mean MD in cingulum hippocampus on FA skeleton (left)
LF	beta1_2	0.206 [0.0214, 0.399]*	Mean MD in cingulum hippocampus on FA skeleton (left)
LF	beta4_2	0.24 [0.0531, 0.459]*	Mean MD in cingulum hippocampus on FA skeleton (left)
LF	beta3_2	-0.197 [-0.348, -0.0209]*	Mean MD in cingulum hippocampus on FA skeleton (left)
LF	c_prime_2	-0.263 [-0.395, -0.089]*	Mean MD in cingulum hippocampus on FA skeleton (left)
LF	ind3_2	-0.0465 [-0.125, -5.791e-03]*	Mean MD in cingulum hippocampus on FA skeleton (left)
LF	total_2	-0.294 [-0.436, -0.133]*	Mean MD in cingulum hippocampus on FA skeleton (left)
EF	beta1_0	0.213 [0.0157, 0.61]*	Mean MD in cingulum hippocampus on FA skeleton (left)
EF	beta1_1	0.179 [0.0191, 0.388]*	Mean MD in cingulum hippocampus on FA skeleton (left)
EF	c_prime_1	-0.317 [-0.454, -0.164]*	Mean MD in cingulum hippocampus on FA skeleton (left)
EF	total_1	-0.36 [-0.494, -0.207]*	Mean MD in cingulum hippocampus on FA skeleton (left)
EF	beta1_2	0.206 [0.0214, 0.399]*	Mean MD in cingulum hippocampus on FA skeleton (left)
EF	beta4_2	0.24 [0.0531, 0.459]*	Mean MD in cingulum hippocampus on FA skeleton (left)
EF	beta3_2	-0.143 [-0.324, -1.794e-03]*	Mean MD in cingulum hippocampus on FA skeleton (left)
EF	ind3_2	-0.0326 [-0.108, -3.471e-03]*	Mean MD in cingulum hippocampus on FA skeleton (left)
EF	total_2	-0.252 [-0.395, -0.0436]*	Mean MD in cingulum hippocampus on FA skeleton (left)
EF	diff_c_prime_1_0	-0.266 [-0.575, -0.042]*	Mean MD in cingulum hippocampus on FA skeleton (left)
EF	diff_total_1_0	-0.31 [-0.606, -0.0808]*	Mean MD in cingulum hippocampus on FA skeleton (left)
VS	beta1_0	0.213 [0.0157, 0.61]*	Mean MD in cingulum hippocampus on FA skeleton (left)
VS	beta3_0	-0.234 [-0.381, -0.0968]*	Mean MD in cingulum hippocampus on FA skeleton (left)
VS	c_prime_0	-0.233 [-0.411, -0.0587]*	Mean MD in cingulum hippocampus on FA skeleton (left)
VS	total_0	-0.223 [-0.396, -0.0501]*	Mean MD in cingulum hippocampus on FA skeleton (left)
VS	beta1_1	0.179 [0.0191, 0.388]*	Mean MD in cingulum hippocampus on FA skeleton (left)
VS	beta3_1	-0.224 [-0.411, -0.0734]*	Mean MD in cingulum hippocampus on FA skeleton (left)
VS	ind3_1	-0.058 [-0.156, -8.288e-03]*	Mean MD in cingulum hippocampus on FA skeleton (left)
VS	beta1_2	0.206 [0.0214, 0.399]*	Mean MD in cingulum hippocampus on FA skeleton (left)
VS	beta4_2	0.24 [0.0531, 0.459]*	Mean MD in cingulum hippocampus on FA skeleton (left)
VS	beta3_2	-0.223 [-0.401, -0.0556]*	Mean MD in cingulum hippocampus on FA skeleton (left)
VS	c_prime_2	-0.238 [-0.423, -0.0723]*	Mean MD in cingulum hippocampus on FA skeleton (left)
VS	ind3_2	-0.0562 [-0.157, -6.722e-03]*	Mean MD in cingulum hippocampus on FA skeleton (left)
VS	total_2	-0.292 [-0.475, -0.112]*	Mean MD in cingulum hippocampus on FA skeleton (left)
MMSE	beta1_0	0.213 [0.0157, 0.61]*	Mean MD in superior longitudinal fasciculus on FA skeleton (right)
MMSE	c_prime_0	-0.322 [-0.516, -0.13]*	Mean MD in superior longitudinal fasciculus on FA skeleton (right)
MMSE	total_0	-0.287 [-0.498, -0.1]*	Mean MD in superior longitudinal fasciculus on FA skeleton (right)
MMSE	beta1_1	0.179 [0.0191, 0.388]*	Mean MD in superior longitudinal fasciculus on FA skeleton (right)
MMSE	c_prime_1	-0.502 [-0.675, -0.236]*	Mean MD in superior longitudinal fasciculus on FA skeleton (right)
MMSE	ind_1	-0.0311 [-0.104, -2.083e-04]*	Mean MD in superior longitudinal fasciculus on FA skeleton (right)
MMSE	total_1	-0.533 [-0.705, -0.268]*	Mean MD in superior longitudinal fasciculus on FA skeleton (right)
MMSE	beta1_2	0.206 [0.0214, 0.399]*	Mean MD in superior longitudinal fasciculus on FA skeleton (right)
MMSE	beta2_2	0.334 [0.16, 0.566]*	Mean MD in superior longitudinal fasciculus on FA skeleton (right)
MMSE	beta4_2	0.194 [0.0187, 0.335]*	Mean MD in superior longitudinal fasciculus on FA skeleton (right)
MMSE	beta3_2	-0.203 [-0.422, -0.053]*	Mean MD in superior longitudinal fasciculus on FA skeleton (right)
MMSE	ind1_2	-0.0134 [-0.0659, -3.319e-03]*	Mean MD in superior longitudinal fasciculus on FA skeleton (right)
MMSE	ind3_2	-0.0373 [-0.109, -7.679e-03]*	Mean MD in superior longitudinal fasciculus on FA skeleton (right)
MMSE	ind_2	-0.0544 [-0.159, -7.820e-03]*	Mean MD in superior longitudinal fasciculus on FA skeleton (right)
MMSE	total_2	-0.262 [-0.469, -0.0361]*	Mean MD in superior longitudinal fasciculus on FA skeleton (right)
MMSE	diff_beta2_2_0	0.304 [0.123, 0.61]*	Mean MD in superior longitudinal fasciculus on FA skeleton (right)
MMSE	diff_ind1_2_0	-0.0131 [-0.065, -2.929e-03]*	Mean MD in superior longitudinal fasciculus on FA skeleton (right)
MMSE	diff_ind1_2_1	-0.011 [-0.0622, -7.156e-04]*	Mean MD in superior longitudinal fasciculus on FA skeleton (right)
MMSE	diff_ind3_2_0	-0.0412 [-0.114, -4.293e-03]*	Mean MD in superior longitudinal fasciculus on FA skeleton (right)
MMSE	diff_ind_1_0	-0.0665 [-0.178, -3.124e-04]*	Mean MD in superior longitudinal fasciculus on FA skeleton (right)
MMSE	diff_ind_2_0	-0.0899 [-0.233, -0.0163]*	Mean MD in superior longitudinal fasciculus on FA skeleton (right)
MoCA	beta1_0	0.213 [0.0157, 0.61]*	Mean MD in superior longitudinal fasciculus on FA skeleton (right)
MoCA	c_prime_0	-0.241 [-0.38, -0.0735]*	Mean MD in superior longitudinal fasciculus on FA skeleton (right)
MoCA	total_0	-0.22 [-0.363, -0.0657]*	Mean MD in superior longitudinal fasciculus on FA skeleton (right)
MoCA	beta1_1	0.179 [0.0191, 0.388]*	Mean MD in superior longitudinal fasciculus on FA skeleton (right)
MoCA	beta3_1	-0.135 [-0.251, -3.337e-03]*	Mean MD in superior longitudinal fasciculus on FA skeleton (right)
MoCA	c_prime_1	-0.442 [-0.585, -0.198]*	Mean MD in superior longitudinal fasciculus on FA skeleton (right)
MoCA	total_1	-0.472 [-0.612, -0.227]*	Mean MD in superior longitudinal fasciculus on FA skeleton (right)
MoCA	beta1_2	0.206 [0.0214, 0.399]*	Mean MD in superior longitudinal fasciculus on FA skeleton (right)
MoCA	beta2_2	0.334 [0.16, 0.566]*	Mean MD in superior longitudinal fasciculus on FA skeleton (right)
MoCA	beta4_2	0.194 [0.0187, 0.335]*	Mean MD in superior longitudinal fasciculus on FA skeleton (right)
MoCA	c_prime_2	-0.329 [-0.48, -0.134]*	Mean MD in superior longitudinal fasciculus on FA skeleton (right)
MoCA	ind1_2	-6.449e-03 [-0.0405, -5.110e-04]*	Mean MD in superior longitudinal fasciculus on FA skeleton (right)
MoCA	total_2	-0.361 [-0.526, -0.184]*	Mean MD in superior longitudinal fasciculus on FA skeleton (right)
MoCA	diff_beta2_2_0	0.304 [0.123, 0.61]*	Mean MD in superior longitudinal fasciculus on FA skeleton (right)
EM	beta1_0	0.213 [0.0157, 0.61]*	Mean MD in superior longitudinal fasciculus on FA skeleton (right)
EM	c_prime_0	-0.243 [-0.363, -0.144]*	Mean MD in superior longitudinal fasciculus on FA skeleton (right)
EM	total_0	-0.25 [-0.352, -0.146]*	Mean MD in superior longitudinal fasciculus on FA skeleton (right)
EM	beta1_1	0.179 [0.0191, 0.388]*	Mean MD in superior longitudinal fasciculus on FA skeleton (right)
EM	beta3_1	-0.134 [-0.265, -0.0221]*	Mean MD in superior longitudinal fasciculus on FA skeleton (right)
EM	c_prime_1	-0.163 [-0.295, -0.0104]*	Mean MD in superior longitudinal fasciculus on FA skeleton (right)
EM	ind3_1	-0.0206 [-0.0639, -8.788e-04]*	Mean MD in superior longitudinal fasciculus on FA skeleton (right)
EM	ind_1	-0.0401 [-0.124, -3.531e-03]*	Mean MD in superior longitudinal fasciculus on FA skeleton (right)
EM	total_1	-0.204 [-0.332, -0.0676]*	Mean MD in superior longitudinal fasciculus on FA skeleton (right)
EM	beta1_2	0.206 [0.0214, 0.399]*	Mean MD in superior longitudinal fasciculus on FA skeleton (right)
EM	beta2_2	0.334 [0.16, 0.566]*	Mean MD in superior longitudinal fasciculus on FA skeleton (right)
EM	beta4_2	0.194 [0.0187, 0.335]*	Mean MD in superior longitudinal fasciculus on FA skeleton (right)
EM	diff_beta2_2_0	0.304 [0.123, 0.61]*	Mean MD in superior longitudinal fasciculus on FA skeleton (right)
ISP	beta1_0	0.213 [0.0157, 0.61]*	Mean MD in superior longitudinal fasciculus on FA skeleton (right)

ISP	c_prime_0	-0.183 [-0.309, -4.977e-04]*	Mean MD in superior longitudinal fasciculus on FA skeleton (right)
ISP	total_0	-0.169 [-0.307, -7.943e-03]*	Mean MD in superior longitudinal fasciculus on FA skeleton (right)
ISP	beta1_1	0.179 [0.0191, 0.388]*	Mean MD in superior longitudinal fasciculus on FA skeleton (right)
ISP	c_prime_1	-0.402 [-0.519, -0.256]*	Mean MD in superior longitudinal fasciculus on FA skeleton (right)
ISP	total_1	-0.399 [-0.514, -0.25]*	Mean MD in superior longitudinal fasciculus on FA skeleton (right)
ISP	beta1_2	0.206 [0.0214, 0.399]*	Mean MD in superior longitudinal fasciculus on FA skeleton (right)
ISP	beta2_2	0.334 [0.16, 0.566]*	Mean MD in superior longitudinal fasciculus on FA skeleton (right)
ISP	beta4_2	0.194 [0.0187, 0.335]*	Mean MD in superior longitudinal fasciculus on FA skeleton (right)
ISP	c_prime_2	-0.293 [-0.429, -0.124]*	Mean MD in superior longitudinal fasciculus on FA skeleton (right)
ISP	total_2	-0.267 [-0.389, -0.118]*	Mean MD in superior longitudinal fasciculus on FA skeleton (right)
ISP	diff_beta2_2_0	0.304 [0.123, 0.61]*	Mean MD in superior longitudinal fasciculus on FA skeleton (right)
ISP	diff_c_prime_1_0	-0.22 [-0.433, -0.0313]*	Mean MD in superior longitudinal fasciculus on FA skeleton (right)
ISP	diff_total_1_0	-0.23 [-0.426, -0.0261]*	Mean MD in superior longitudinal fasciculus on FA skeleton (right)
LF	beta1_0	0.213 [0.0157, 0.61]*	Mean MD in superior longitudinal fasciculus on FA skeleton (right)
LF	c_prime_0	-0.236 [-0.367, -0.0707]*	Mean MD in superior longitudinal fasciculus on FA skeleton (right)
LF	total_0	-0.233 [-0.359, -0.0909]*	Mean MD in superior longitudinal fasciculus on FA skeleton (right)
LF	beta1_1	0.179 [0.0191, 0.388]*	Mean MD in superior longitudinal fasciculus on FA skeleton (right)
LF	c_prime_1	-0.229 [-0.372, -0.0984]*	Mean MD in superior longitudinal fasciculus on FA skeleton (right)
LF	total_1	-0.23 [-0.369, -0.1]*	Mean MD in superior longitudinal fasciculus on FA skeleton (right)
LF	beta1_2	0.206 [0.0214, 0.399]*	Mean MD in superior longitudinal fasciculus on FA skeleton (right)
LF	beta2_2	0.334 [0.16, 0.566]*	Mean MD in superior longitudinal fasciculus on FA skeleton (right)
LF	beta4_2	0.194 [0.0187, 0.335]*	Mean MD in superior longitudinal fasciculus on FA skeleton (right)
LF	c_prime_2	-0.292 [-0.439, -0.116]*	Mean MD in superior longitudinal fasciculus on FA skeleton (right)
LF	total_2	-0.294 [-0.436, -0.133]*	Mean MD in superior longitudinal fasciculus on FA skeleton (right)
LF	diff_beta2_2_0	0.304 [0.123, 0.61]*	Mean MD in superior longitudinal fasciculus on FA skeleton (right)
EF	beta1_0	0.213 [0.0157, 0.61]*	Mean MD in superior longitudinal fasciculus on FA skeleton (right)
EF	beta1_1	0.179 [0.0191, 0.388]*	Mean MD in superior longitudinal fasciculus on FA skeleton (right)
EF	c_prime_1	-0.342 [-0.471, -0.191]*	Mean MD in superior longitudinal fasciculus on FA skeleton (right)
EF	total_1	-0.36 [-0.494, -0.207]*	Mean MD in superior longitudinal fasciculus on FA skeleton (right)
EF	beta1_2	0.206 [0.0214, 0.399]*	Mean MD in superior longitudinal fasciculus on FA skeleton (right)
EF	beta2_2	0.334 [0.16, 0.566]*	Mean MD in superior longitudinal fasciculus on FA skeleton (right)
EF	beta4_2	0.194 [0.0187, 0.335]*	Mean MD in superior longitudinal fasciculus on FA skeleton (right)
EF	c_prime_2	-0.25 [-0.406, -0.0202]*	Mean MD in superior longitudinal fasciculus on FA skeleton (right)
EF	total_2	-0.252 [-0.395, -0.0436]*	Mean MD in superior longitudinal fasciculus on FA skeleton (right)
EF	diff_beta2_2_0	0.304 [0.123, 0.61]*	Mean MD in superior longitudinal fasciculus on FA skeleton (right)
EF	diff_c_prime_1_0	-0.28 [-0.596, -0.0641]*	Mean MD in superior longitudinal fasciculus on FA skeleton (right)
EF	diff_total_1_0	-0.31 [-0.606, -0.0808]*	Mean MD in superior longitudinal fasciculus on FA skeleton (right)
VS	beta1_0	0.213 [0.0157, 0.61]*	Mean MD in superior longitudinal fasciculus on FA skeleton (right)
VS	c_prime_0	-0.249 [-0.425, -0.0626]*	Mean MD in superior longitudinal fasciculus on FA skeleton (right)
VS	total_0	-0.223 [-0.396, -0.0501]*	Mean MD in superior longitudinal fasciculus on FA skeleton (right)
VS	beta1_1	0.179 [0.0191, 0.388]*	Mean MD in superior longitudinal fasciculus on FA skeleton (right)
VS	c_prime_1	-0.189 [-0.371, -3.349e-03]*	Mean MD in superior longitudinal fasciculus on FA skeleton (right)
VS	beta1_2	0.206 [0.0214, 0.399]*	Mean MD in superior longitudinal fasciculus on FA skeleton (right)
VS	beta2_2	0.334 [0.16, 0.566]*	Mean MD in superior longitudinal fasciculus on FA skeleton (right)
VS	beta4_2	0.194 [0.0187, 0.335]*	Mean MD in superior longitudinal fasciculus on FA skeleton (right)
VS	c_prime_2	-0.288 [-0.467, -0.0929]*	Mean MD in superior longitudinal fasciculus on FA skeleton (right)
VS	total_2	-0.292 [-0.475, -0.112]*	Mean MD in superior longitudinal fasciculus on FA skeleton (right)
VS	diff_beta2_2_0	0.304 [0.123, 0.61]*	Mean MD in superior longitudinal fasciculus on FA skeleton (right)
MMSE	beta1_0	0.213 [0.0157, 0.61]*	Mean MD in superior longitudinal fasciculus on FA skeleton (left)
MMSE	c_prime_0	-0.322 [-0.522, -0.132]*	Mean MD in superior longitudinal fasciculus on FA skeleton (left)
MMSE	total_0	-0.287 [-0.498, -0.1]*	Mean MD in superior longitudinal fasciculus on FA skeleton (left)
MMSE	beta1_1	0.179 [0.0191, 0.388]*	Mean MD in superior longitudinal fasciculus on FA skeleton (left)
MMSE	beta4_1	0.186 [0.0183, 0.352]*	Mean MD in superior longitudinal fasciculus on FA skeleton (left)
MMSE	beta3_1	-0.184 [-0.355, -0.0984]*	Mean MD in superior longitudinal fasciculus on FA skeleton (left)
MMSE	c_prime_1	-0.485 [-0.654, -0.216]*	Mean MD in superior longitudinal fasciculus on FA skeleton (left)
MMSE	ind3_1	-0.0355 [-0.0915, -5.630e-03]*	Mean MD in superior longitudinal fasciculus on FA skeleton (left)
MMSE	ind_1	-0.0487 [-0.14, -7.639e-03]*	Mean MD in superior longitudinal fasciculus on FA skeleton (left)
MMSE	total_1	-0.533 [-0.705, -0.268]*	Mean MD in superior longitudinal fasciculus on FA skeleton (left)
MMSE	beta1_2	0.206 [0.0214, 0.399]*	Mean MD in superior longitudinal fasciculus on FA skeleton (left)
MMSE	beta3_2	-0.208 [-0.423, -0.0711]*	Mean MD in superior longitudinal fasciculus on FA skeleton (left)
MMSE	ind1_2	-0.0101 [-0.0566, -5.423e-04]*	Mean MD in superior longitudinal fasciculus on FA skeleton (left)
MMSE	ind_2	-0.0356 [-0.135, -1.592e-03]*	Mean MD in superior longitudinal fasciculus on FA skeleton (left)
MMSE	total_2	-0.262 [-0.469, -0.0361]*	Mean MD in superior longitudinal fasciculus on FA skeleton (left)
MMSE	diff_beta4_1_0	0.228 [0.0312, 0.464]*	Mean MD in superior longitudinal fasciculus on FA skeleton (left)
MMSE	diff_ind3_1_0	-0.0389 [-0.0969, -6.528e-03]*	Mean MD in superior longitudinal fasciculus on FA skeleton (left)
MMSE	diff_ind_1_0	-0.0837 [-0.203, -0.0134]*	Mean MD in superior longitudinal fasciculus on FA skeleton (left)
MMSE	diff_ind_2_0	-0.0706 [-0.217, -7.459e-03]*	Mean MD in superior longitudinal fasciculus on FA skeleton (left)
MoCA	beta1_0	0.213 [0.0157, 0.61]*	Mean MD in superior longitudinal fasciculus on FA skeleton (left)
MoCA	c_prime_0	-0.242 [-0.38, -0.0767]*	Mean MD in superior longitudinal fasciculus on FA skeleton (left)
MoCA	total_0	-0.22 [-0.363, -0.0657]*	Mean MD in superior longitudinal fasciculus on FA skeleton (left)
MoCA	beta1_1	0.179 [0.0191, 0.388]*	Mean MD in superior longitudinal fasciculus on FA skeleton (left)
MoCA	beta4_1	0.186 [0.0183, 0.352]*	Mean MD in superior longitudinal fasciculus on FA skeleton (left)
MoCA	beta3_1	-0.19 [-0.341, -0.0817]*	Mean MD in superior longitudinal fasciculus on FA skeleton (left)
MoCA	c_prime_1	-0.428 [-0.57, -0.189]*	Mean MD in superior longitudinal fasciculus on FA skeleton (left)
MoCA	ind3_1	-0.0364 [-0.0918, -4.538e-03]*	Mean MD in superior longitudinal fasciculus on FA skeleton (left)
MoCA	ind_1	-0.044 [-0.123, -2.144e-03]*	Mean MD in superior longitudinal fasciculus on FA skeleton (left)
MoCA	total_1	-0.472 [-0.612, -0.227]*	Mean MD in superior longitudinal fasciculus on FA skeleton (left)
MoCA	beta1_2	0.206 [0.0214, 0.399]*	Mean MD in superior longitudinal fasciculus on FA skeleton (left)
MoCA	beta3_2	-0.176 [-0.362, -0.0434]*	Mean MD in superior longitudinal fasciculus on FA skeleton (left)
MoCA	c_prime_2	-0.332 [-0.475, -0.143]*	Mean MD in superior longitudinal fasciculus on FA skeleton (left)
MoCA	ind1_2	-8.684e-03 [-0.0428, -9.446e-05]*	Mean MD in superior longitudinal fasciculus on FA skeleton (left)
MoCA	total_2	-0.361 [-0.526, -0.184]*	Mean MD in superior longitudinal fasciculus on FA skeleton (left)
MoCA	diff_beta4_1_0	0.228 [0.0312, 0.464]*	Mean MD in superior longitudinal fasciculus on FA skeleton (left)
MoCA	diff_ind3_1_0	-0.0433 [-0.0944, -5.144e-03]*	Mean MD in superior longitudinal fasciculus on FA skeleton (left)

MoCA	diff_ind_1_0	-0.0666 [-0.151, -7.577e-03]*	Mean MD in superior longitudinal fasciculus on FA skeleton (left)
MoCA	diff_ind_2_0	-0.0513 [-0.15, -3.918e-03]*	Mean MD in superior longitudinal fasciculus on FA skeleton (left)
EM	beta1_0	0.213 [0.0157, 0.61]*	Mean MD in superior longitudinal fasciculus on FA skeleton (left)
EM	c_prime_0	-0.242 [-0.366, -0.146]*	Mean MD in superior longitudinal fasciculus on FA skeleton (left)
EM	total_0	-0.25 [-0.352, -0.146]*	Mean MD in superior longitudinal fasciculus on FA skeleton (left)
EM	beta1_1	0.179 [0.0191, 0.388]*	Mean MD in superior longitudinal fasciculus on FA skeleton (left)
EM	beta4_1	0.186 [0.0183, 0.352]*	Mean MD in superior longitudinal fasciculus on FA skeleton (left)
EM	beta3_1	-0.25 [-0.381, -0.14]*	Mean MD in superior longitudinal fasciculus on FA skeleton (left)
EM	ind3_1	-0.0457 [-0.103, -8.743e-03]*	Mean MD in superior longitudinal fasciculus on FA skeleton (left)
EM	ind_1	-0.0652 [-0.153, -0.0151]*	Mean MD in superior longitudinal fasciculus on FA skeleton (left)
EM	total_1	-0.204 [-0.332, -0.0676]*	Mean MD in superior longitudinal fasciculus on FA skeleton (left)
EM	beta1_2	0.206 [0.0214, 0.399]*	Mean MD in superior longitudinal fasciculus on FA skeleton (left)
EM	ind1_2	-5.356e-03 [-0.0372, -5.080e-04]*	Mean MD in superior longitudinal fasciculus on FA skeleton (left)
EM	ind_2	-0.0332 [-0.099, -1.485e-04]*	Mean MD in superior longitudinal fasciculus on FA skeleton (left)
EM	diff_beta3_1_0	-0.171 [-0.37, -1.272e-03]*	Mean MD in superior longitudinal fasciculus on FA skeleton (left)
EM	diff_beta4_1_0	0.228 [0.0312, 0.464]*	Mean MD in superior longitudinal fasciculus on FA skeleton (left)
EM	diff_ind3_1_0	-0.0476 [-0.11, -8.772e-03]*	Mean MD in superior longitudinal fasciculus on FA skeleton (left)
EM	diff_ind_1_0	-0.0571 [-0.159, -1.257e-03]*	Mean MD in superior longitudinal fasciculus on FA skeleton (left)
ISP	beta1_0	0.213 [0.0157, 0.61]*	Mean MD in superior longitudinal fasciculus on FA skeleton (left)
ISP	c_prime_0	-0.184 [-0.31, -2.226e-03]*	Mean MD in superior longitudinal fasciculus on FA skeleton (left)
ISP	total_0	-0.169 [-0.307, -7.943e-03]*	Mean MD in superior longitudinal fasciculus on FA skeleton (left)
ISP	beta1_1	0.179 [0.0191, 0.388]*	Mean MD in superior longitudinal fasciculus on FA skeleton (left)
ISP	beta4_1	0.186 [0.0183, 0.352]*	Mean MD in superior longitudinal fasciculus on FA skeleton (left)
ISP	beta3_1	-0.122 [-0.256, -0.0131]*	Mean MD in superior longitudinal fasciculus on FA skeleton (left)
ISP	c_prime_1	-0.387 [-0.501, -0.246]*	Mean MD in superior longitudinal fasciculus on FA skeleton (left)
ISP	ind3_1	-0.0242 [-0.0739, -9.200e-04]*	Mean MD in superior longitudinal fasciculus on FA skeleton (left)
ISP	total_1	-0.399 [-0.514, -0.25]*	Mean MD in superior longitudinal fasciculus on FA skeleton (left)
ISP	beta1_2	0.206 [0.0214, 0.399]*	Mean MD in superior longitudinal fasciculus on FA skeleton (left)
ISP	beta3_2	-0.163 [-0.324, -0.0413]*	Mean MD in superior longitudinal fasciculus on FA skeleton (left)
ISP	c_prime_2	-0.273 [-0.401, -0.123]*	Mean MD in superior longitudinal fasciculus on FA skeleton (left)
ISP	ind1_2	-7.054e-03 [-0.0543, -1.340e-03]*	Mean MD in superior longitudinal fasciculus on FA skeleton (left)
ISP	total_2	-0.267 [-0.389, -0.118]*	Mean MD in superior longitudinal fasciculus on FA skeleton (left)
ISP	diff_beta4_1_0	0.228 [0.0312, 0.464]*	Mean MD in superior longitudinal fasciculus on FA skeleton (left)
ISP	diff_ind1_2_0	-7.055e-03 [-0.048, -1.106e-03]*	Mean MD in superior longitudinal fasciculus on FA skeleton (left)
ISP	diff_c_prime_1_0	-0.203 [-0.415, -0.0165]*	Mean MD in superior longitudinal fasciculus on FA skeleton (left)
ISP	diff_total_1_0	-0.23 [-0.426, -0.0261]*	Mean MD in superior longitudinal fasciculus on FA skeleton (left)
LF	beta1_0	0.213 [0.0157, 0.61]*	Mean MD in superior longitudinal fasciculus on FA skeleton (left)
LF	c_prime_0	-0.237 [-0.37, -0.0788]*	Mean MD in superior longitudinal fasciculus on FA skeleton (left)
LF	total_0	-0.233 [-0.359, -0.0909]*	Mean MD in superior longitudinal fasciculus on FA skeleton (left)
LF	beta1_1	0.179 [0.0191, 0.388]*	Mean MD in superior longitudinal fasciculus on FA skeleton (left)
LF	beta4_1	0.186 [0.0183, 0.352]*	Mean MD in superior longitudinal fasciculus on FA skeleton (left)
LF	c_prime_1	-0.217 [-0.36, -0.0793]*	Mean MD in superior longitudinal fasciculus on FA skeleton (left)
LF	ind3_1	-0.0247 [-0.0674, -6.101e-05]*	Mean MD in superior longitudinal fasciculus on FA skeleton (left)
LF	total_1	-0.23 [-0.369, -0.1]*	Mean MD in superior longitudinal fasciculus on FA skeleton (left)
LF	beta1_2	0.206 [0.0214, 0.399]*	Mean MD in superior longitudinal fasciculus on FA skeleton (left)
LF	beta3_2	-0.148 [-0.301, -1.226e-03]*	Mean MD in superior longitudinal fasciculus on FA skeleton (left)
LF	c_prime_2	-0.293 [-0.438, -0.123]*	Mean MD in superior longitudinal fasciculus on FA skeleton (left)
LF	total_2	-0.294 [-0.436, -0.133]*	Mean MD in superior longitudinal fasciculus on FA skeleton (left)
LF	diff_beta4_1_0	0.228 [0.0312, 0.464]*	Mean MD in superior longitudinal fasciculus on FA skeleton (left)
EF	beta1_0	0.213 [0.0157, 0.61]*	Mean MD in superior longitudinal fasciculus on FA skeleton (left)
EF	beta1_1	0.179 [0.0191, 0.388]*	Mean MD in superior longitudinal fasciculus on FA skeleton (left)
EF	beta4_1	0.186 [0.0183, 0.352]*	Mean MD in superior longitudinal fasciculus on FA skeleton (left)
EF	beta3_1	-0.128 [-0.288, -0.0162]*	Mean MD in superior longitudinal fasciculus on FA skeleton (left)
EF	c_prime_1	-0.33 [-0.462, -0.181]*	Mean MD in superior longitudinal fasciculus on FA skeleton (left)
EF	ind3_1	-0.026 [-0.0849, -7.488e-04]*	Mean MD in superior longitudinal fasciculus on FA skeleton (left)
EF	total_1	-0.36 [-0.494, -0.207]*	Mean MD in superior longitudinal fasciculus on FA skeleton (left)
EF	beta1_2	0.206 [0.0214, 0.399]*	Mean MD in superior longitudinal fasciculus on FA skeleton (left)
EF	beta3_2	-0.181 [-0.332, -0.0636]*	Mean MD in superior longitudinal fasciculus on FA skeleton (left)
EF	c_prime_2	-0.234 [-0.383, -0.0196]*	Mean MD in superior longitudinal fasciculus on FA skeleton (left)
EF	ind1_2	-7.579e-03 [-0.0485, -1.513e-03]*	Mean MD in superior longitudinal fasciculus on FA skeleton (left)
EF	total_2	-0.252 [-0.395, -0.0436]*	Mean MD in superior longitudinal fasciculus on FA skeleton (left)
EF	diff_beta4_1_0	0.228 [0.0312, 0.464]*	Mean MD in superior longitudinal fasciculus on FA skeleton (left)
EF	diff_ind1_2_0	-7.316e-03 [-0.0471, -1.137e-03]*	Mean MD in superior longitudinal fasciculus on FA skeleton (left)
EF	diff_ind3_1_0	-0.0291 [-0.0849, -8.186e-04]*	Mean MD in superior longitudinal fasciculus on FA skeleton (left)
EF	diff_c_prime_1_0	-0.268 [-0.582, -0.052]*	Mean MD in superior longitudinal fasciculus on FA skeleton (left)
EF	diff_total_1_0	-0.31 [-0.606, -0.0808]*	Mean MD in superior longitudinal fasciculus on FA skeleton (left)
VS	beta1_0	0.213 [0.0157, 0.61]*	Mean MD in superior longitudinal fasciculus on FA skeleton (left)
VS	c_prime_0	-0.248 [-0.427, -0.0632]*	Mean MD in superior longitudinal fasciculus on FA skeleton (left)
VS	total_0	-0.223 [-0.396, -0.0501]*	Mean MD in superior longitudinal fasciculus on FA skeleton (left)
VS	beta1_1	0.179 [0.0191, 0.388]*	Mean MD in superior longitudinal fasciculus on FA skeleton (left)
VS	beta4_1	0.186 [0.0183, 0.352]*	Mean MD in superior longitudinal fasciculus on FA skeleton (left)
VS	c_prime_1	-0.179 [-0.363, -1.197e-03]*	Mean MD in superior longitudinal fasciculus on FA skeleton (left)
VS	beta1_2	0.206 [0.0214, 0.399]*	Mean MD in superior longitudinal fasciculus on FA skeleton (left)
VS	c_prime_2	-0.29 [-0.467, -0.0928]*	Mean MD in superior longitudinal fasciculus on FA skeleton (left)
VS	total_2	-0.292 [-0.475, -0.112]*	Mean MD in superior longitudinal fasciculus on FA skeleton (left)
VS	diff_beta4_1_0	0.228 [0.0312, 0.464]*	Mean MD in superior longitudinal fasciculus on FA skeleton (left)
MMSE	beta1_0	0.213 [0.0157, 0.61]*	Mean MD in tapetum on FA skeleton (left)
MMSE	beta3_0	-0.395 [-0.556, -0.235]*	Mean MD in tapetum on FA skeleton (left)
MMSE	c_prime_0	-0.341 [-0.515, -0.176]*	Mean MD in tapetum on FA skeleton (left)
MMSE	ind1_0	-0.0103 [-0.0398, -3.871e-03]*	Mean MD in tapetum on FA skeleton (left)
MMSE	total_0	-0.287 [-0.498, -0.1]*	Mean MD in tapetum on FA skeleton (left)
MMSE	beta1_1	0.179 [0.0191, 0.388]*	Mean MD in tapetum on FA skeleton (left)
MMSE	beta4_1	0.156 [0.023, 0.28]*	Mean MD in tapetum on FA skeleton (left)
MMSE	beta3_1	-0.308 [-0.57, -0.071]*	Mean MD in tapetum on FA skeleton (left)

MMSE	c_prime_1	-0.472 [-0.642, -0.207]*	Mean MD in tapetum on FA skeleton (left)
MMSE	ind1_1	4.846e-03 [1.370e-04, 0.038]*	Mean MD in tapetum on FA skeleton (left)
MMSE	ind3_1	-0.0482 [-0.128, -6.906e-03]*	Mean MD in tapetum on FA skeleton (left)
MMSE	ind_1	-0.0614 [-0.153, -7.543e-03]*	Mean MD in tapetum on FA skeleton (left)
MMSE	total_1	-0.533 [-0.705, -0.268]*	Mean MD in tapetum on FA skeleton (left)
MMSE	beta1_2	0.206 [0.0214, 0.399]*	Mean MD in tapetum on FA skeleton (left)
MMSE	beta4_2	0.263 [0.0435, 0.601]*	Mean MD in tapetum on FA skeleton (left)
MMSE	beta3_2	-0.328 [-0.519, -0.176]*	Mean MD in tapetum on FA skeleton (left)
MMSE	ind3_2	-0.078 [-0.177, -0.0199]*	Mean MD in tapetum on FA skeleton (left)
MMSE	ind_2	-0.0951 [-0.225, -0.0308]*	Mean MD in tapetum on FA skeleton (left)
MMSE	total_2	-0.262 [-0.469, -0.0361]*	Mean MD in tapetum on FA skeleton (left)
MMSE	diff_beta2_1_0	-0.274 [-0.475, -0.0181]*	Mean MD in tapetum on FA skeleton (left)
MMSE	diff_beta4_1_0	0.208 [7.417e-03, 0.426]*	Mean MD in tapetum on FA skeleton (left)
MMSE	diff_beta4_2_0	0.316 [0.0436, 0.685]*	Mean MD in tapetum on FA skeleton (left)
MMSE	diff_beta5_1_0	-0.241 [-0.566, -0.0856]*	Mean MD in tapetum on FA skeleton (left)
MMSE	diff_beta5_2_0	-0.247 [-0.585, -0.0567]*	Mean MD in tapetum on FA skeleton (left)
MMSE	diff_ind1_1_0	0.0151 [6.765e-03, 0.055]*	Mean MD in tapetum on FA skeleton (left)
MMSE	diff_ind2_1_0	-0.0599 [-0.189, -3.922e-03]*	Mean MD in tapetum on FA skeleton (left)
MMSE	diff_ind2_2_0	-0.06 [-0.198, -3.686e-03]*	Mean MD in tapetum on FA skeleton (left)
MMSE	diff_ind3_2_0	-0.101 [-0.227, -0.0228]*	Mean MD in tapetum on FA skeleton (left)
MMSE	diff_ind_1_0	-0.116 [-0.247, -0.0177]*	Mean MD in tapetum on FA skeleton (left)
MMSE	diff_ind_2_0	-0.149 [-0.315, -0.0494]*	Mean MD in tapetum on FA skeleton (left)
MMSE	diff_c_prime_2_1	0.305 [0.0115, 0.588]*	Mean MD in tapetum on FA skeleton (left)
MoCA	beta1_0	0.213 [0.0157, 0.61]*	Mean MD in tapetum on FA skeleton (left)
MoCA	beta3_0	-0.366 [-0.483, -0.234]*	Mean MD in tapetum on FA skeleton (left)
MoCA	c_prime_0	-0.255 [-0.377, -0.109]*	Mean MD in tapetum on FA skeleton (left)
MoCA	ind1_0	-9.530e-03 [-0.0353, -2.928e-03]*	Mean MD in tapetum on FA skeleton (left)
MoCA	total_0	-0.22 [-0.363, -0.0657]*	Mean MD in tapetum on FA skeleton (left)
MoCA	beta1_1	0.179 [0.0191, 0.388]*	Mean MD in tapetum on FA skeleton (left)
MoCA	beta4_1	0.156 [0.023, 0.28]*	Mean MD in tapetum on FA skeleton (left)
MoCA	beta3_1	-0.276 [-0.476, -0.0609]*	Mean MD in tapetum on FA skeleton (left)
MoCA	c_prime_1	-0.422 [-0.569, -0.185]*	Mean MD in tapetum on FA skeleton (left)
MoCA	ind3_1	-0.0429 [-0.099, -3.444e-03]*	Mean MD in tapetum on FA skeleton (left)
MoCA	ind_1	-0.0505 [-0.123, -1.749e-04]*	Mean MD in tapetum on FA skeleton (left)
MoCA	total_1	-0.472 [-0.612, -0.227]*	Mean MD in tapetum on FA skeleton (left)
MoCA	beta1_2	0.206 [0.0214, 0.399]*	Mean MD in tapetum on FA skeleton (left)
MoCA	beta4_2	0.263 [0.0435, 0.601]*	Mean MD in tapetum on FA skeleton (left)
MoCA	beta3_2	-0.325 [-0.444, -0.228]*	Mean MD in tapetum on FA skeleton (left)
MoCA	c_prime_2	-0.262 [-0.382, -0.108]*	Mean MD in tapetum on FA skeleton (left)
MoCA	ind3_2	-0.0858 [-0.225, -0.0161]*	Mean MD in tapetum on FA skeleton (left)
MoCA	ind_2	-0.099 [-0.243, -0.0231]*	Mean MD in tapetum on FA skeleton (left)
MoCA	total_2	-0.361 [-0.526, -0.184]*	Mean MD in tapetum on FA skeleton (left)
MoCA	diff_beta2_1_0	-0.274 [-0.475, -0.0181]*	Mean MD in tapetum on FA skeleton (left)
MoCA	diff_beta4_1_0	0.208 [7.417e-03, 0.426]*	Mean MD in tapetum on FA skeleton (left)
MoCA	diff_beta4_2_0	0.316 [0.0436, 0.685]*	Mean MD in tapetum on FA skeleton (left)
MoCA	diff_beta5_1_0	-0.168 [-0.421, -0.0233]*	Mean MD in tapetum on FA skeleton (left)
MoCA	diff_ind1_1_0	0.0137 [5.649e-03, 0.0453]*	Mean MD in tapetum on FA skeleton (left)
MoCA	diff_ind2_1_0	-0.037 [-0.124, -1.596e-03]*	Mean MD in tapetum on FA skeleton (left)
MoCA	diff_ind2_2_0	-0.0403 [-0.133, -2.784e-03]*	Mean MD in tapetum on FA skeleton (left)
MoCA	diff_ind3_2_0	-0.106 [-0.255, -0.017]*	Mean MD in tapetum on FA skeleton (left)
MoCA	diff_ind_1_0	-0.0859 [-0.187, -6.172e-03]*	Mean MD in tapetum on FA skeleton (left)
MoCA	diff_ind_2_0	-0.135 [-0.292, -0.036]*	Mean MD in tapetum on FA skeleton (left)
EM	beta1_0	0.213 [0.0157, 0.61]*	Mean MD in tapetum on FA skeleton (left)
EM	beta3_0	-0.263 [-0.355, -0.0964]*	Mean MD in tapetum on FA skeleton (left)
EM	c_prime_0	-0.254 [-0.366, -0.154]*	Mean MD in tapetum on FA skeleton (left)
EM	ind1_0	-6.843e-03 [-0.0274, -1.978e-03]*	Mean MD in tapetum on FA skeleton (left)
EM	total_0	-0.25 [-0.352, -0.146]*	Mean MD in tapetum on FA skeleton (left)
EM	beta1_1	0.179 [0.0191, 0.388]*	Mean MD in tapetum on FA skeleton (left)
EM	beta4_1	0.156 [0.023, 0.28]*	Mean MD in tapetum on FA skeleton (left)
EM	c_prime_1	-0.178 [-0.327, -0.0344]*	Mean MD in tapetum on FA skeleton (left)
EM	total_1	-0.204 [-0.332, -0.0676]*	Mean MD in tapetum on FA skeleton (left)
EM	beta1_2	0.206 [0.0214, 0.399]*	Mean MD in tapetum on FA skeleton (left)
EM	beta4_2	0.263 [0.0435, 0.601]*	Mean MD in tapetum on FA skeleton (left)
EM	diff_beta2_1_0	-0.274 [-0.475, -0.0181]*	Mean MD in tapetum on FA skeleton (left)
EM	diff_beta4_1_0	0.208 [7.417e-03, 0.426]*	Mean MD in tapetum on FA skeleton (left)
EM	diff_beta4_2_0	0.316 [0.0436, 0.685]*	Mean MD in tapetum on FA skeleton (left)
EM	diff_ind1_1_0	7.480e-03 [6.863e-04, 0.0296]*	Mean MD in tapetum on FA skeleton (left)
EM	diff_ind1_2_0	6.819e-03 [1.089e-03, 0.0281]*	Mean MD in tapetum on FA skeleton (left)
ISP	beta1_0	0.213 [0.0157, 0.61]*	Mean MD in tapetum on FA skeleton (left)
ISP	beta3_0	-0.307 [-0.434, -0.204]*	Mean MD in tapetum on FA skeleton (left)
ISP	c_prime_0	-0.197 [-0.312, -0.0349]*	Mean MD in tapetum on FA skeleton (left)
ISP	ind1_0	-8.206e-03 [-0.0295, -2.080e-03]*	Mean MD in tapetum on FA skeleton (left)
ISP	total_0	-0.169 [-0.307, -7.943e-03]*	Mean MD in tapetum on FA skeleton (left)
ISP	beta1_1	0.179 [0.0191, 0.388]*	Mean MD in tapetum on FA skeleton (left)
ISP	beta4_1	0.156 [0.023, 0.28]*	Mean MD in tapetum on FA skeleton (left)
ISP	c_prime_1	-0.391 [-0.522, -0.251]*	Mean MD in tapetum on FA skeleton (left)
ISP	total_1	-0.399 [-0.514, -0.25]*	Mean MD in tapetum on FA skeleton (left)
ISP	beta1_2	0.206 [0.0214, 0.399]*	Mean MD in tapetum on FA skeleton (left)
ISP	beta4_2	0.263 [0.0435, 0.601]*	Mean MD in tapetum on FA skeleton (left)
ISP	beta3_2	-0.232 [-0.37, -0.103]*	Mean MD in tapetum on FA skeleton (left)
ISP	c_prime_2	-0.235 [-0.37, -0.0851]*	Mean MD in tapetum on FA skeleton (left)
ISP	ind3_2	-0.0547 [-0.138, -0.0162]*	Mean MD in tapetum on FA skeleton (left)
ISP	total_2	-0.267 [-0.389, -0.118]*	Mean MD in tapetum on FA skeleton (left)
ISP	diff_beta2_1_0	-0.274 [-0.475, -0.0181]*	Mean MD in tapetum on FA skeleton (left)

ISP	diff_beta4_1_0	0.208 [7.417e-03, 0.426]*	Mean MD in tapetum on FA skeleton (left)
ISP	diff_beta4_2_0	0.316 [0.0436, 0.685]*	Mean MD in tapetum on FA skeleton (left)
ISP	diff_ind1_1_0	0.0103 [2.554e-03, 0.0372]*	Mean MD in tapetum on FA skeleton (left)
ISP	diff_ind3_2_0	-0.0705 [-0.169, -0.0147]*	Mean MD in tapetum on FA skeleton (left)
ISP	diff_c_prime_1_0	-0.194 [-0.392, -8.248e-03]*	Mean MD in tapetum on FA skeleton (left)
ISP	diff_total_1_0	-0.23 [-0.426, -0.0261]*	Mean MD in tapetum on FA skeleton (left)
LF	beta1_0	0.213 [0.0157, 0.61]*	Mean MD in tapetum on FA skeleton (left)
LF	beta3_0	-0.23 [-0.354, -0.101]*	Mean MD in tapetum on FA skeleton (left)
LF	c_prime_0	-0.245 [-0.382, -0.103]*	Mean MD in tapetum on FA skeleton (left)
LF	ind1_0	-6.038e-03 [-0.0244, -1.700e-03]*	Mean MD in tapetum on FA skeleton (left)
LF	total_0	-0.233 [-0.359, -0.0909]*	Mean MD in tapetum on FA skeleton (left)
LF	beta1_1	0.179 [0.0191, 0.388]*	Mean MD in tapetum on FA skeleton (left)
LF	beta4_1	0.156 [0.023, 0.28]*	Mean MD in tapetum on FA skeleton (left)
LF	c_prime_1	-0.214 [-0.364, -0.0834]*	Mean MD in tapetum on FA skeleton (left)
LF	total_1	-0.23 [-0.369, -0.1]*	Mean MD in tapetum on FA skeleton (left)
LF	beta1_2	0.206 [0.0214, 0.399]*	Mean MD in tapetum on FA skeleton (left)
LF	beta4_2	0.263 [0.0435, 0.601]*	Mean MD in tapetum on FA skeleton (left)
LF	beta3_2	-0.236 [-0.359, -0.121]*	Mean MD in tapetum on FA skeleton (left)
LF	c_prime_2	-0.248 [-0.391, -0.0754]*	Mean MD in tapetum on FA skeleton (left)
LF	ind3_2	-0.061 [-0.144, -6.256e-03]*	Mean MD in tapetum on FA skeleton (left)
LF	total_2	-0.294 [-0.436, -0.133]*	Mean MD in tapetum on FA skeleton (left)
LF	diff_beta2_1_0	-0.274 [-0.475, -0.0181]*	Mean MD in tapetum on FA skeleton (left)
LF	diff_beta4_1_0	0.208 [7.417e-03, 0.426]*	Mean MD in tapetum on FA skeleton (left)
LF	diff_beta4_2_0	0.316 [0.0436, 0.685]*	Mean MD in tapetum on FA skeleton (left)
LF	diff_ind1_1_0	8.898e-03 [2.760e-03, 0.033]*	Mean MD in tapetum on FA skeleton (left)
LF	diff_ind3_2_0	-0.0707 [-0.167, -8.995e-03]*	Mean MD in tapetum on FA skeleton (left)
EF	beta1_0	0.213 [0.0157, 0.61]*	Mean MD in tapetum on FA skeleton (left)
EF	beta1_1	0.179 [0.0191, 0.388]*	Mean MD in tapetum on FA skeleton (left)
EF	beta4_1	0.156 [0.023, 0.28]*	Mean MD in tapetum on FA skeleton (left)
EF	c_prime_1	-0.329 [-0.471, -0.186]*	Mean MD in tapetum on FA skeleton (left)
EF	total_1	-0.36 [-0.494, -0.207]*	Mean MD in tapetum on FA skeleton (left)
EF	beta1_2	0.206 [0.0214, 0.399]*	Mean MD in tapetum on FA skeleton (left)
EF	beta4_2	0.263 [0.0435, 0.601]*	Mean MD in tapetum on FA skeleton (left)
EF	beta3_2	-0.215 [-0.349, -0.0751]*	Mean MD in tapetum on FA skeleton (left)
EF	ind3_2	-0.0491 [-0.123, -0.0164]*	Mean MD in tapetum on FA skeleton (left)
EF	ind_2	-0.0483 [-0.135, -7.824e-03]*	Mean MD in tapetum on FA skeleton (left)
EF	total_2	-0.252 [-0.395, -0.0436]*	Mean MD in tapetum on FA skeleton (left)
EF	diff_beta2_1_0	-0.274 [-0.475, -0.0181]*	Mean MD in tapetum on FA skeleton (left)
EF	diff_beta4_1_0	0.208 [7.417e-03, 0.426]*	Mean MD in tapetum on FA skeleton (left)
EF	diff_beta4_2_0	0.316 [0.0436, 0.685]*	Mean MD in tapetum on FA skeleton (left)
EF	diff_ind3_2_0	-0.0545 [-0.129, -0.0138]*	Mean MD in tapetum on FA skeleton (left)
EF	diff_ind_2_0	-0.0616 [-0.155, -8.683e-03]*	Mean MD in tapetum on FA skeleton (left)
EF	diff_c_prime_1_0	-0.266 [-0.595, -0.0452]*	Mean MD in tapetum on FA skeleton (left)
EF	diff_total_1_0	-0.31 [-0.606, -0.0808]*	Mean MD in tapetum on FA skeleton (left)
VS	beta1_0	0.213 [0.0157, 0.61]*	Mean MD in tapetum on FA skeleton (left)
VS	beta3_0	-0.335 [-0.465, -0.193]*	Mean MD in tapetum on FA skeleton (left)
VS	beta5_0	0.14 [0.0191, 0.292]*	Mean MD in tapetum on FA skeleton (left)
VS	c_prime_0	-0.265 [-0.426, -0.0953]*	Mean MD in tapetum on FA skeleton (left)
VS	ind1_0	-8.532e-03 [-0.0331, -3.883e-03]*	Mean MD in tapetum on FA skeleton (left)
VS	ind2_0	0.0311 [9.075e-04, 0.128]*	Mean MD in tapetum on FA skeleton (left)
VS	total_0	-0.223 [-0.396, -0.0501]*	Mean MD in tapetum on FA skeleton (left)
VS	beta1_1	0.179 [0.0191, 0.388]*	Mean MD in tapetum on FA skeleton (left)
VS	beta4_1	0.156 [0.023, 0.28]*	Mean MD in tapetum on FA skeleton (left)
VS	c_prime_1	-0.181 [-0.364, -8.494e-03]*	Mean MD in tapetum on FA skeleton (left)
VS	beta1_2	0.206 [0.0214, 0.399]*	Mean MD in tapetum on FA skeleton (left)
VS	beta4_2	0.263 [0.0435, 0.601]*	Mean MD in tapetum on FA skeleton (left)
VS	beta3_2	-0.2 [-0.344, -0.0939]*	Mean MD in tapetum on FA skeleton (left)
VS	c_prime_2	-0.239 [-0.413, -0.0698]*	Mean MD in tapetum on FA skeleton (left)
VS	ind3_2	-0.0552 [-0.174, -6.637e-03]*	Mean MD in tapetum on FA skeleton (left)
VS	total_2	-0.292 [-0.475, -0.112]*	Mean MD in tapetum on FA skeleton (left)
VS	diff_beta2_1_0	-0.274 [-0.475, -0.0181]*	Mean MD in tapetum on FA skeleton (left)
VS	diff_beta4_1_0	0.208 [7.417e-03, 0.426]*	Mean MD in tapetum on FA skeleton (left)
VS	diff_beta4_2_0	0.316 [0.0436, 0.685]*	Mean MD in tapetum on FA skeleton (left)
VS	diff_ind1_1_0	0.0107 [5.221e-03, 0.0436]*	Mean MD in tapetum on FA skeleton (left)
VS	diff_ind1_2_0	9.644e-03 [8.240e-04, 0.0323]*	Mean MD in tapetum on FA skeleton (left)
VS	diff_ind3_2_0	-0.0742 [-0.2, -5.038e-03]*	Mean MD in tapetum on FA skeleton (left)
VS	diff_ind_2_0	-0.0943 [-0.231, -0.0133]*	Mean MD in tapetum on FA skeleton (left)

Table S9

description	effect of plasma Aβ42	effect of Aβ42*CSVD burden
Volume of grey matter in Frontal Pole (left)	β = 0.061, p = 0.5, pFDR = 0.92	B = 0.027, p = 0.63, pFDR = 0.96
Volume of grey matter in Frontal Pole (right)	β = 0.076, p = 0.4, pFDR = 0.92	B = -0.045, p = 0.43, pFDR = 0.96
Volume of grey matter in Insular Cortex (left)	β = 0.062, p = 0.5, pFDR = 0.92	B = 7.23e-03, p = 0.9, pFDR = 0.96
Volume of grey matter in Insular Cortex (right)	β = 6.82e-03, p = 0.94, pFDR = 0.99	B = -0.018, p = 0.76, pFDR = 0.96
Volume of grey matter in Superior Frontal Gyrus (left)	β = 0.042, p = 0.65, pFDR = 0.97	B = 0.018, p = 0.76, pFDR = 0.96
Volume of grey matter in Superior Frontal Gyrus (right)	β = 0.016, p = 0.87, pFDR = 0.99	B = -0.021, p = 0.72, pFDR = 0.96
Volume of grey matter in Middle Frontal Gyrus (left)	β = -1.61e-04, p = 1, pFDR = 1	B = -0.029, p = 0.64, pFDR = 0.96
Volume of grey matter in Middle Frontal Gyrus (right)	β = 0.074, p = 0.43, pFDR = 0.92	B = -0.023, p = 0.7, pFDR = 0.96
Volume of grey matter in Inferior Frontal Gyrus, pars triangularis (left)	β = 0.076, p = 0.42, pFDR = 0.92	B = -0.03, p = 0.62, pFDR = 0.96
Volume of grey matter in Inferior Frontal Gyrus, pars triangularis (right)	β = 0.038, p = 0.69, pFDR = 0.99	B = 0.025, p = 0.68, pFDR = 0.96
Volume of grey matter in Inferior Frontal Gyrus, pars opercularis (left)	β = 0.021, p = 0.82, pFDR = 0.99	B = 9.02e-03, p = 0.88, pFDR = 0.96
Volume of grey matter in Inferior Frontal Gyrus, pars opercularis (right)	β = 0.2, p = 0.029, pFDR = 0.58	B = -0.098, p = 0.093, pFDR = 0.91
Volume of grey matter in Precentral Gyrus (left)	β = 0.073, p = 0.38, pFDR = 0.92	B = -0.068, p = 0.2, pFDR = 0.93
Volume of grey matter in Precentral Gyrus (right)	β = 0.026, p = 0.77, pFDR = 0.99	B = -0.047, p = 0.4, pFDR = 0.96
Volume of grey matter in Temporal Pole (left)	β = -0.078, p = 0.39, pFDR = 0.92	B = 0.074, p = 0.2, pFDR = 0.93
Volume of grey matter in Temporal Pole (right)	β = -4.28e-03, p = 0.96, pFDR = 0.99	B = 0.027, p = 0.64, pFDR = 0.96
Volume of grey matter in Superior Temporal Gyrus, anterior division (left)	β = 0.056, p = 0.56, pFDR = 0.92	B = 0.017, p = 0.78, pFDR = 0.96
Volume of grey matter in Superior Temporal Gyrus, anterior division (right)	β = -0.15, p = 0.12, pFDR = 0.91	B = 0.046, p = 0.45, pFDR = 0.96
Volume of grey matter in Superior Temporal Gyrus, posterior division (left)	β = 0.091, p = 0.33, pFDR = 0.92	B = -0.056, p = 0.35, pFDR = 0.96
Volume of grey matter in Superior Temporal Gyrus, posterior division (right)	β = 0.022, p = 0.82, pFDR = 0.99	B = 0.032, p = 0.59, pFDR = 0.96
Volume of grey matter in Middle Temporal Gyrus, anterior division (left)	β = -0.064, p = 0.51, pFDR = 0.92	B = 0.016, p = 0.79, pFDR = 0.96
Volume of grey matter in Middle Temporal Gyrus, anterior division (right)	β = -0.01, p = 0.92, pFDR = 0.99	B = -0.046, p = 0.45, pFDR = 0.96
Volume of grey matter in Middle Temporal Gyrus, posterior division (left)	β = 0.015, p = 0.87, pFDR = 0.99	B = 0.039, p = 0.52, pFDR = 0.96
Volume of grey matter in Middle Temporal Gyrus, posterior division (right)	β = -0.059, p = 0.54, pFDR = 0.92	B = 5.81e-03, p = 0.92, pFDR = 0.96
Volume of grey matter in Middle Temporal Gyrus, temporooccipital part (left)	β = -0.033, p = 0.73, pFDR = 0.99	B = -0.011, p = 0.85, pFDR = 0.96
Volume of grey matter in Middle Temporal Gyrus, temporooccipital part (right)	β = 0.019, p = 0.84, pFDR = 0.99	B = -0.026, p = 0.67, pFDR = 0.96
Volume of grey matter in Inferior Temporal Gyrus, anterior division (left)	β = -0.075, p = 0.42, pFDR = 0.92	B = 0.076, p = 0.2, pFDR = 0.93
Volume of grey matter in Inferior Temporal Gyrus, anterior division (right)	β = -0.064, p = 0.49, pFDR = 0.92	B = -2.24e-03, p = 0.97, pFDR = 0.98
Volume of grey matter in Inferior Temporal Gyrus, posterior division (left)	β = -0.13, p = 0.16, pFDR = 0.92	B = 0.12, p = 0.043, pFDR = 0.91
Volume of grey matter in Inferior Temporal Gyrus, posterior division (right)	β = -0.011, p = 0.91, pFDR = 0.99	B = -0.029, p = 0.63, pFDR = 0.96
Volume of grey matter in Inferior Temporal Gyrus, temporooccipital part (left)	β = 0.012, p = 0.9, pFDR = 0.99	B = -0.051, p = 0.39, pFDR = 0.96
Volume of grey matter in Inferior Temporal Gyrus, temporooccipital part (right)	β = 0.049, p = 0.6, pFDR = 0.94	B = -7.96e-03, p = 0.89, pFDR = 0.96
Volume of grey matter in Postcentral Gyrus (left)	β = -0.05, p = 0.55, pFDR = 0.92	B = -2.08e-03, p = 0.97, pFDR = 0.98
Volume of grey matter in Postcentral Gyrus (right)	β = -0.16, p = 0.071, pFDR = 0.68	B = 0.078, p = 0.15, pFDR = 0.93
Volume of grey matter in Superior Parietal Lobule (left)	β = -0.18, p = 0.052, pFDR = 0.58	B = 0.11, p = 0.054, pFDR = 0.91
Volume of grey matter in Superior Parietal Lobule (right)	β = -0.01, p = 0.91, pFDR = 0.99	B = -0.012, p = 0.84, pFDR = 0.96
Volume of grey matter in Supramarginal Gyrus, anterior division (left)	β = 0.013, p = 0.89, pFDR = 0.99	B = -0.095, p = 0.11, pFDR = 0.91
Volume of grey matter in Supramarginal Gyrus, anterior division (right)	β = -0.2, p = 0.034, pFDR = 0.58	B = 0.11, p = 0.067, pFDR = 0.91
Volume of grey matter in Supramarginal Gyrus, posterior division (left)	β = 0.16, p = 0.087, pFDR = 0.78	B = -0.069, p = 0.24, pFDR = 0.93
Volume of grey matter in Supramarginal Gyrus, posterior division (right)	β = 0.015, p = 0.87, pFDR = 0.99	B = 0.018, p = 0.76, pFDR = 0.96
Volume of grey matter in Angular Gyrus (left)	β = 0.22, p = 0.021, pFDR = 0.58	B = -0.056, p = 0.36, pFDR = 0.96
Volume of grey matter in Angular Gyrus (right)	β = 0.079, p = 0.4, pFDR = 0.92	B = -0.11, p = 0.074, pFDR = 0.91
Volume of grey matter in Lateral Occipital Cortex, superior division (left)	β = 0.06, p = 0.52, pFDR = 0.92	B = -0.011, p = 0.85, pFDR = 0.96
Volume of grey matter in Lateral Occipital Cortex, superior division (right)	β = 0.2, p = 0.036, pFDR = 0.58	B = -0.098, p = 0.1, pFDR = 0.91
Volume of grey matter in Lateral Occipital Cortex, inferior division (left)	β = -0.028, p = 0.77, pFDR = 0.99	B = -0.053, p = 0.38, pFDR = 0.96
Volume of grey matter in Lateral Occipital Cortex, inferior division (right)	β = -0.068, p = 0.47, pFDR = 0.92	B = 0.016, p = 0.78, pFDR = 0.96
Volume of grey matter in Intracalcarine Cortex (left)	β = -0.19, p = 0.05, pFDR = 0.58	B = 0.013, p = 0.82, pFDR = 0.96
Volume of grey matter in Intracalcarine Cortex (right)	β = -0.095, p = 0.31, pFDR = 0.92	B = -0.079, p = 0.18, pFDR = 0.93
Volume of grey matter in Frontal Medial Cortex (left)	β = -0.064, p = 0.5, pFDR = 0.92	B = 0.052, p = 0.38, pFDR = 0.96
Volume of grey matter in Frontal Medial Cortex (right)	β = 0.015, p = 0.87, pFDR = 0.99	B = -0.01, p = 0.86, pFDR = 0.96
Volume of grey matter in Juxtapositional Lobule Cortex (formerly Supplementary Motor Cortex) (left)	β = -0.077, p = 0.41, pFDR = 0.92	B = 0.042, p = 0.47, pFDR = 0.96
Volume of grey matter in Juxtapositional Lobule Cortex (formerly Supplementary Motor Cortex) (right)	β = -0.097, p = 0.3, pFDR = 0.92	B = 0.038, p = 0.52, pFDR = 0.96
Volume of grey matter in Subcallosal Cortex (left)	β = 0.016, p = 0.86, pFDR = 0.99	B = -0.01, p = 0.86, pFDR = 0.96
Volume of grey matter in Subcallosal Cortex (right)	β = -0.044, p = 0.64, pFDR = 0.97	B = 0.017, p = 0.78, pFDR = 0.96
Volume of grey matter in Paracingulate Gyrus (left)	β = 6.21e-03, p = 0.95, pFDR = 0.99	B = -0.054, p = 0.37, pFDR = 0.96
Volume of grey matter in Paracingulate Gyrus (right)	β = 0.011, p = 0.9, pFDR = 0.99	B = -0.09, p = 0.13, pFDR = 0.93
Volume of grey matter in Cingulate Gyrus, anterior division (left)	β = -3.01e-05, p = 1, pFDR = 1	B = 6.51e-03, p = 0.91, pFDR = 0.96
Volume of grey matter in Cingulate Gyrus, anterior division (right)	β = -0.084, p = 0.37, pFDR = 0.92	B = 1.74e-03, p = 0.98, pFDR = 0.98
Volume of grey matter in Cingulate Gyrus, posterior division (left)	β = -0.074, p = 0.43, pFDR = 0.92	B = -0.03, p = 0.62, pFDR = 0.96
Volume of grey matter in Cingulate Gyrus, posterior division (right)	β = 3.59e-03, p = 0.97, pFDR = 0.99	B = 8.92e-03, p = 0.88, pFDR = 0.96
Volume of grey matter in Precuneous Cortex (left)	β = -0.1, p = 0.28, pFDR = 0.92	B = 0.021, p = 0.72, pFDR = 0.96
Volume of grey matter in Precuneous Cortex (right)	β = -0.079, p = 0.4, pFDR = 0.92	B = 0.016, p = 0.79, pFDR = 0.96
Volume of grey matter in Cuneal Cortex (left)	β = 0.087, p = 0.36, pFDR = 0.92	B = -0.02, p = 0.74, pFDR = 0.96
Volume of grey matter in Cuneal Cortex (right)	β = 0.1, p = 0.28, pFDR = 0.92	B = -0.073, p = 0.23, pFDR = 0.93
Volume of grey matter in Frontal Orbital Cortex (left)	β = -0.027, p = 0.77, pFDR = 0.99	B = -0.035, p = 0.55, pFDR = 0.96
Volume of grey matter in Frontal Orbital Cortex (right)	β = -0.14, p = 0.12, pFDR = 0.91	B = 0.046, p = 0.43, pFDR = 0.96
Volume of grey matter in Parahippocampal Gyrus, anterior division (left)	β = -0.014, p = 0.88, pFDR = 0.99	B = 0.037, p = 0.53, pFDR = 0.96
Volume of grey matter in Parahippocampal Gyrus, anterior division (right)	β = 0.026, p = 0.77, pFDR = 0.99	B = -0.031, p = 0.59, pFDR = 0.96
Volume of grey matter in Parahippocampal Gyrus, posterior division (left)	β = 5.35e-03, p = 0.95, pFDR = 0.99	B = -0.089, p = 0.13, pFDR = 0.93
Volume of grey matter in Parahippocampal Gyrus, posterior division (right)	β = -0.18, p = 0.041, pFDR = 0.58	B = 0.063, p = 0.25, pFDR = 0.93
Volume of grey matter in Lingual Gyrus (left)	β = -0.1, p = 0.28, pFDR = 0.92	B = 7.15e-04, p = 0.99, pFDR = 0.99
Volume of grey matter in Lingual Gyrus (right)	β = -0.067, p = 0.47, pFDR = 0.92	B = -6.01e-03, p = 0.92, pFDR = 0.96
Volume of grey matter in Temporal Fusiform Cortex, anterior division (left)	β = -0.056, p = 0.55, pFDR = 0.92	B = 0.1, p = 0.091, pFDR = 0.91
Volume of grey matter in Temporal Fusiform Cortex, anterior division (right)	β = -0.064, p = 0.49, pFDR = 0.92	B = 0.011, p = 0.85, pFDR = 0.96
Volume of grey matter in Temporal Fusiform Cortex, posterior division (left)	β = 0.045, p = 0.63, pFDR = 0.97	B = -0.021, p = 0.72, pFDR = 0.96
Volume of grey matter in Temporal Fusiform Cortex, posterior division (right)	β = -0.077, p = 0.4, pFDR = 0.92	B = -0.028, p = 0.63, pFDR = 0.96
Volume of grey matter in Temporal Occipital Fusiform Cortex (left)	β = 0.01, p = 0.91, pFDR = 0.99	B = -0.1, p = 0.089, pFDR = 0.91
Volume of grey matter in Temporal Occipital Fusiform Cortex (right)	β = 0.052, p = 0.58, pFDR = 0.92	B = -0.016, p = 0.79, pFDR = 0.96
Volume of grey matter in Occipital Fusiform Gyrus (left)	β = -0.049, p = 0.58, pFDR = 0.92	B = -0.036, p = 0.53, pFDR = 0.96
Volume of grey matter in Occipital Fusiform Gyrus (right)	β = -0.058, p = 0.52, pFDR = 0.92	B = -7.44e-03, p = 0.9, pFDR = 0.96
Volume of grey matter in Frontal Operculum Cortex (left)	β = -0.018, p = 0.85, pFDR = 0.99	B = -0.088, p = 0.14, pFDR = 0.93
Volume of grey matter in Frontal Operculum Cortex (right)	β = 0.18, p = 0.05, pFDR = 0.58	B = -0.077, p = 0.2, pFDR = 0.93
Volume of grey matter in Central Opercular Cortex (left)	β = 0.054, p = 0.57, pFDR = 0.92	B = -0.072, p = 0.23, pFDR = 0.93
Volume of grey matter in Central Opercular Cortex (right)	β = -0.13, p = 0.17, pFDR = 0.92	B = -0.03, p = 0.61, pFDR = 0.96
Volume of grey matter in Parietal Operculum Cortex (left)	β = -0.071, p = 0.46, pFDR = 0.92	B = -0.032, p = 0.6, pFDR = 0.96
Volume of grey matter in Parietal Operculum Cortex (right)	β = 0.043, p = 0.65, pFDR = 0.97	B = -0.077, p = 0.2, pFDR = 0.93
Volume of grey matter in Planum Polare (left)	β = 0.18, p = 0.051, pFDR = 0.58	B = -0.083, p = 0.16, pFDR = 0.93
Volume of grey matter in Planum Polare (right)	β = -0.03, p = 0.74, pFDR = 0.99	B = -0.092, p = 0.11, pFDR = 0.91
Volume of grey matter in Heschl's Gyrus (includes H1 and H2) (left)	β = -0.065, p = 0.47, pFDR = 0.92	B = 0.021, p = 0.71, pFDR = 0.96
Volume of grey matter in Heschl's Gyrus (includes H1 and H2) (right)	β = -0.13, p = 0.14, pFDR = 0.92	B = 0.02, p = 0.73, pFDR = 0.96
Volume of grey matter in Planum Temporale (left)	β = -0.066, p = 0.49, pFDR = 0.92	B = -0.027, p = 0.65, pFDR = 0.96
Volume of grey matter in Planum Temporale (right)	β = 0.068, p = 0.47, pFDR = 0.92	B = -0.035, p = 0.56, pFDR = 0.96
Volume of grey matter in Supracalcarine Cortex (left)	β = 0.15, p = 0.12, pFDR = 0.91	B = -0.13, p = 0.03, pFDR = 0.91
Volume of grey matter in Supracalcarine Cortex (right)	β = 0.18, p = 0.056, pFDR = 0.58	B = -0.14, p = 0.017, pFDR = 0.71
Volume of grey matter in Occipital Pole (left)	β = -0.11, p = 0.21, pFDR = 0.92	B = 0.063, p = 0.27, pFDR = 0.96
Volume of grey matter in Occipital Pole (right)	β = -0.075, p = 0.4, pFDR = 0.92	B = 0.036, p = 0.52, pFDR = 0.96

**Volume of grey matter in Thalamus (left)**	β = -0.12, p = 0.17, pFDR = 0.92	B = 0.014, p = 0.8, pFDR = 0.96
**Volume of grey matter in Thalamus (right)**	β = -0.11, p = 0.21, pFDR = 0.92	B = 0.019, p = 0.74, pFDR = 0.96
**Volume of grey matter in Caudate (left)**	β = 0.11, p = 0.19, pFDR = 0.92	B = -0.074, p = 0.17, pFDR = 0.93
**Volume of grey matter in Caudate (right)**	β = 0.13, p = 0.14, pFDR = 0.92	B = -0.048, p = 0.39, pFDR = 0.96
**Volume of grey matter in Putamen (left)**	β = 0.03, p = 0.73, pFDR = 0.99	B = -6.71e-03, p = 0.9, pFDR = 0.96
**Volume of grey matter in Putamen (right)**	β = 0.062, p = 0.48, pFDR = 0.92	B = -0.015, p = 0.79, pFDR = 0.96
**Volume of grey matter in Pallidum (left)**	β = 0.12, p = 0.18, pFDR = 0.92	B = -0.062, p = 0.29, pFDR = 0.96
**Volume of grey matter in Pallidum (right)**	β = 0.074, p = 0.42, pFDR = 0.92	B = -0.042, p = 0.47, pFDR = 0.96
**Volume of grey matter in Hippocampus (left)**	β = 0.078, p = 0.36, pFDR = 0.92	B = 0.031, p = 0.57, pFDR = 0.96
**Volume of grey matter in Hippocampus (right)**	β = 0.058, p = 0.5, pFDR = 0.92	B = -7.43e-03, p = 0.89, pFDR = 0.96
**Volume of grey matter in Amygdala (left)**	β = 0.073, p = 0.39, pFDR = 0.92	B = 0.056, p = 0.3, pFDR = 0.96
**Volume of grey matter in Amygdala (right)**	β = 0.16, p = 0.054, pFDR = 0.58	B = 0.021, p = 0.7, pFDR = 0.96
**Volume of grey matter in Ventral Striatum (left)**	β = -0.018, p = 0.84, pFDR = 0.99	B = -6.89e-03, p = 0.9, pFDR = 0.96
**Volume of grey matter in Ventral Striatum (right)**	β = 0.027, p = 0.77, pFDR = 0.99	B = -0.031, p = 0.59, pFDR = 0.96
**Volume of grey matter in Frontal Pole (left)**	β = 0.03, p = 0.72, pFDR = 0.99	B = 0.054, p = 0.31, pFDR = 0.93
**Volume of grey matter in Frontal Pole (right)**	β = 0.045, p = 0.6, pFDR = 0.99	B = -9.28e-03, p = 0.86, pFDR = 0.99
**Volume of grey matter in Insular Cortex (left)**	β = 0.033, p = 0.68, pFDR = 0.99	B = 0.035, p = 0.48, pFDR = 0.93
**Volume of grey matter in Insular Cortex (right)**	β = -0.01, p = 0.89, pFDR = 0.99	B = 0.016, p = 0.75, pFDR = 0.99
**Volume of grey matter in Superior Frontal Gyrus (left)**	β = 0.029, p = 0.74, pFDR = 0.99	B = 0.032, p = 0.56, pFDR = 0.99
**Volume of grey matter in Superior Frontal Gyrus (right)**	β = 5.04e-03, p = 0.95, pFDR = 0.99	B = -3.39e-03, p = 0.95, pFDR = 0.99
**Volume of grey matter in Middle Frontal Gyrus (left)**	β = -5.61e-03, p = 0.95, pFDR = 0.99	B = -0.014, p = 0.81, pFDR = 0.99
**Volume of grey matter in Middle Frontal Gyrus (right)**	β = 0.057, p = 0.51, pFDR = 0.97	B = 1.31e-03, p = 0.98, pFDR = 0.99
**Volume of grey matter in Inferior Frontal Gyrus, pars triangularis (left)**	β = 0.069, p = 0.46, pFDR = 0.97	B = -6.34e-03, p = 0.91, pFDR = 0.99
**Volume of grey matter in Inferior Frontal Gyrus, pars triangularis (right)**	β = 0.023, p = 0.81, pFDR = 0.99	B = 0.04, p = 0.5, pFDR = 0.93
**Volume of grey matter in Inferior Frontal Gyrus, pars opercularis (left)**	β = 0.013, p = 0.89, pFDR = 0.99	B = 0.033, p = 0.58, pFDR = 0.99
**Volume of grey matter in Inferior Frontal Gyrus, pars opercularis (right)**	β = 0.19, p = 0.037, pFDR = 0.75	B = -0.086, p = 0.14, pFDR = 0.93
**Volume of grey matter in Precentral Gyrus (left)**	β = 0.057, p = 0.5, pFDR = 0.97	B = -0.041, p = 0.44, pFDR = 0.93
**Volume of grey matter in Precentral Gyrus (right)**	β = 7.94e-03, p = 0.93, pFDR = 0.99	B = -0.017, p = 0.75, pFDR = 0.99
**Volume of grey matter in Temporal Pole (left)**	β = -0.09, p = 0.32, pFDR = 0.97	B = 0.091, p = 0.11, pFDR = 0.93
**Volume of grey matter in Temporal Pole (right)**	β = -0.025, p = 0.78, pFDR = 0.99	B = 0.049, p = 0.39, pFDR = 0.93
**Volume of grey matter in Superior Temporal Gyrus, anterior division (left)**	β = 0.038, p = 0.67, pFDR = 0.99	B = 0.031, p = 0.59, pFDR = 0.99
**Volume of grey matter in Superior Temporal Gyrus, anterior division (right)**	β = -0.15, p = 0.1, pFDR = 0.75	B = 0.06, p = 0.29, pFDR = 0.93
**Volume of grey matter in Superior Temporal Gyrus, posterior division (left)**	β = 0.061, p = 0.49, pFDR = 0.97	B = -0.028, p = 0.62, pFDR = 0.99
**Volume of grey matter in Superior Temporal Gyrus, posterior division (right)**	β = 3.99e-03, p = 0.96, pFDR = 0.99	B = 0.048, p = 0.37, pFDR = 0.93
**Volume of grey matter in Middle Temporal Gyrus, anterior division (left)**	β = -0.076, p = 0.4, pFDR = 0.97	B = 0.034, p = 0.56, pFDR = 0.99
**Volume of grey matter in Middle Temporal Gyrus, anterior division (right)**	β = -0.013, p = 0.89, pFDR = 0.99	B = -0.031, p = 0.6, pFDR = 0.99
**Volume of grey matter in Middle Temporal Gyrus, posterior division (left)**	β = 8.22e-03, p = 0.93, pFDR = 0.99	B = 0.05, p = 0.38, pFDR = 0.93
**Volume of grey matter in Middle Temporal Gyrus, posterior division (right)**	β = -0.068, p = 0.44, pFDR = 0.97	B = 0.023, p = 0.68, pFDR = 0.99
**Volume of grey matter in Middle Temporal Gyrus, temporooccipital part (left)**	β = -0.024, p = 0.8, pFDR = 0.99	B = -3.96e-03, p = 0.95, pFDR = 0.99
**Volume of grey matter in Middle Temporal Gyrus, temporooccipital part (right)**	β = -9.51e-04, p = 0.99, pFDR = 1	B = -5.56e-03, p = 0.92, pFDR = 0.99
**Volume of grey matter in Inferior Temporal Gyrus, anterior division (left)**	β = -0.091, p = 0.32, pFDR = 0.97	B = 0.096, p = 0.095, pFDR = 0.93
**Volume of grey matter in Inferior Temporal Gyrus, anterior division (right)**	β = -0.077, p = 0.4, pFDR = 0.97	B = 0.015, p = 0.8, pFDR = 0.99
**Volume of grey matter in Inferior Temporal Gyrus, posterior division (left)**	β = -0.14, p = 0.11, pFDR = 0.78	B = 0.13, p = 0.023, pFDR = 0.92
**Volume of grey matter in Inferior Temporal Gyrus, posterior division (right)**	β = -0.023, p = 0.8, pFDR = 0.99	B = -9.69e-03, p = 0.86, pFDR = 0.99
**Volume of grey matter in Inferior Temporal Gyrus, temporooccipital part (left)**	β = -2.61e-03, p = 0.98, pFDR = 1	B = -0.022, p = 0.68, pFDR = 0.99
**Volume of grey matter in Inferior Temporal Gyrus, temporooccipital part (right)**	β = 0.019, p = 0.82, pFDR = 0.99	B = 0.014, p = 0.8, pFDR = 0.99
**Volume of grey matter in Postcentral Gyrus (left)**	β = -0.059, p = 0.48, pFDR = 0.97	B = 0.02, p = 0.7, pFDR = 0.99
**Volume of grey matter in Postcentral Gyrus (right)**	β = -0.14, p = 0.092, pFDR = 0.75	B = 0.088, p = 0.1, pFDR = 0.93
**Volume of grey matter in Superior Parietal Lobule (left)**	β = -0.18, p = 0.047, pFDR = 0.75	B = 0.13, p = 0.03, pFDR = 0.92
**Volume of grey matter in Superior Parietal Lobule (right)**	β = -4.96e-03, p = 0.96, pFDR = 0.99	B = 7.43e-04, p = 0.99, pFDR = 0.99
**Volume of grey matter in Supramarginal Gyrus, anterior division (left)**	β = -4.29e-05, p = 1, pFDR = 1	B = -0.077, p = 0.19, pFDR = 0.93
**Volume of grey matter in Supramarginal Gyrus, anterior division (right)**	β = -0.19, p = 0.042, pFDR = 0.75	B = 0.11, p = 0.052, pFDR = 0.92
**Volume of grey matter in Supramarginal Gyrus, posterior division (left)**	β = 0.15, p = 0.084, pFDR = 0.75	B = -0.053, p = 0.35, pFDR = 0.93
**Volume of grey matter in Supramarginal Gyrus, posterior division (right)**	β = 0.015, p = 0.88, pFDR = 0.99	B = 0.029, p = 0.63, pFDR = 0.99
**Volume of grey matter in Angular Gyrus (left)**	β = 0.21, p = 0.022, pFDR = 0.75	B = -0.043, p = 0.46, pFDR = 0.93
**Volume of grey matter in Angular Gyrus (right)**	β = 0.058, p = 0.54, pFDR = 0.99	B = -0.079, p = 0.18, pFDR = 0.93
**Volume of grey matter in Lateral Occipital Cortex, superior division (left)**	β = 0.045, p = 0.61, pFDR = 0.99	B = 0.015, p = 0.78, pFDR = 0.99
**Volume of grey matter in Lateral Occipital Cortex, superior division (right)**	β = 0.16, p = 0.07, pFDR = 0.75	B = -0.062, p = 0.26, pFDR = 0.93
**Volume of grey matter in Lateral Occipital Cortex, inferior division (left)**	β = -0.041, p = 0.64, pFDR = 0.99	B = -0.026, p = 0.64, pFDR = 0.99
**Volume of grey matter in Lateral Occipital Cortex, inferior division (right)**	β = -0.074, p = 0.39, pFDR = 0.97	B = 0.042, p = 0.44, pFDR = 0.93
**Volume of grey matter in Intracalcarine Cortex (left)**	β = -0.17, p = 0.056, pFDR = 0.75	B = 0.021, p = 0.71, pFDR = 0.99
**Volume of grey matter in Intracalcarine Cortex (right)**	β = -0.094, p = 0.29, pFDR = 0.97	B = -0.057, p = 0.31, pFDR = 0.93
**Volume of grey matter in Frontal Medial Cortex (left)**	β = -0.067, p = 0.45, pFDR = 0.97	B = 0.055, p = 0.33, pFDR = 0.93
**Volume of grey matter in Frontal Medial Cortex (right)**	β = -1.64e-03, p = 0.99, pFDR = 1	B = 5.87e-03, p = 0.92, pFDR = 0.99
**Volume of grey matter in Juxtapositional Lobule Cortex (formerly Supplementary Motor Cortex) (left)**	β = -0.074, p = 0.41, pFDR = 0.97	B = 0.051, p = 0.37, pFDR = 0.93
**Volume of grey matter in Juxtapositional Lobule Cortex (formerly Supplementary Motor Cortex) (right)**	β = -0.096, p = 0.28, pFDR = 0.97	B = 0.047, p = 0.41, pFDR = 0.93
**Volume of grey matter in Subcallosal Cortex (left)**	β = -5.84e-03, p = 0.94, pFDR = 0.99	B = 0.01, p = 0.83, pFDR = 0.99
**Volume of grey matter in Subcallosal Cortex (right)**	β = -0.059, p = 0.43, pFDR = 0.97	B = 0.034, p = 0.47, pFDR = 0.93
**Volume of grey matter in Paracingulate Gyrus (left)**	β = -0.017, p = 0.85, pFDR = 0.99	B = -0.028, p = 0.61, pFDR = 0.99
**Volume of grey matter in Paracingulate Gyrus (right)**	β = -0.017, p = 0.84, pFDR = 0.99	B = -0.057, p = 0.29, pFDR = 0.93
**Volume of grey matter in Cingulate Gyrus, anterior division (left)**	β = -0.026, p = 0.77, pFDR = 0.99	B = 0.024, p = 0.67, pFDR = 0.99
**Volume of grey matter in Cingulate Gyrus, anterior division (right)**	β = -0.1, p = 0.26, pFDR = 0.97	B = 0.015, p = 0.79, pFDR = 0.99
**Volume of grey matter in Cingulate Gyrus, posterior division (left)**	β = -0.08, p = 0.3, pFDR = 0.97	B = 3.01e-03, p = 0.95, pFDR = 0.99
**Volume of grey matter in Cingulate Gyrus, posterior division (right)**	β = -0.024, p = 0.76, pFDR = 0.99	B = 0.037, p = 0.46, pFDR = 0.93
**Volume of grey matter in Precuneous Cortex (left)**	β = -0.082, p = 0.33, pFDR = 0.97	B = 0.033, p = 0.54, pFDR = 0.99
**Volume of grey matter in Precuneous Cortex (right)**	β = -0.066, p = 0.43, pFDR = 0.97	B = 0.028, p = 0.6, pFDR = 0.99
**Volume of grey matter in Cuneal Cortex (left)**	β = 0.063, p = 0.46, pFDR = 0.97	B = 4.02e-03, p = 0.94, pFDR = 0.99
**Volume of grey matter in Cuneal Cortex (right)**	β = 0.072, p = 0.41, pFDR = 0.97	B = -0.044, p = 0.42, pFDR = 0.93
**Volume of grey matter in Frontal Orbital Cortex (left)**	β = -0.047, p = 0.57, pFDR = 0.99	B = -5.23e-03, p = 0.92, pFDR = 0.99
**Volume of grey matter in Frontal Orbital Cortex (right)**	β = -0.14, p = 0.095, pFDR = 0.75	B = 0.059, p = 0.26, pFDR = 0.93
**Volume of grey matter in Parahippocampal Gyrus, anterior division (left)**	β = -0.03, p = 0.73, pFDR = 0.99	B = 0.056, p = 0.31, pFDR = 0.93
**Volume of grey matter in Parahippocampal Gyrus, anterior division (right)**	β = 5.48e-03, p = 0.95, pFDR = 0.99	B = -0.014, p = 0.8, pFDR = 0.99
**Volume of grey matter in Parahippocampal Gyrus, posterior division (left)**	β = -0.017, p = 0.85, pFDR = 0.99	B = -0.059, p = 0.3, pFDR = 0.93
**Volume of grey matter in Parahippocampal Gyrus, posterior division (right)**	β = -0.21, p = 0.021, pFDR = 0.75	B = 0.093, p = 0.1, pFDR = 0.93
**Volume of grey matter in Lingual Gyrus (left)**	β = -0.098, p = 0.24, pFDR = 0.97	B = 0.024, p = 0.64, pFDR = 0.99
**Volume of grey matter in Lingual Gyrus (right)**	β = -0.071, p = 0.38, pFDR = 0.97	B = 0.021, p = 0.69, pFDR = 0.99
**Volume of grey matter in Temporal Fusiform Cortex, anterior division (left)**	β = -0.063, p = 0.47, pFDR = 0.97	B = 0.1, p = 0.06, pFDR = 0.93
**Volume of grey matter in Temporal Fusiform Cortex, anterior division (right)**	β = -0.073, p = 0.42, pFDR = 0.97	B = 0.022, p = 0.7, pFDR = 0.99
**Volume of grey matter in Temporal Fusiform Cortex, posterior division (left)**	β = 0.02, p = 0.82, pFDR = 0.99	B = -1.36e-03, p = 0.98, pFDR = 0.99
**Volume of grey matter in Temporal Fusiform Cortex, posterior division (right)**	β = -0.082, p = 0.33, pFDR = 0.97	B = -5.47e-03, p = 0.92, pFDR = 0.99
**Volume of grey matter in Temporal Occipital Fusiform Cortex (left)**	β = 0.011, p = 0.91, pFDR = 0.99	B = -0.088, p = 0.12, pFDR = 0.93
**Volume of grey matter in Temporal Occipital Fusiform Cortex (right)**	β = 0.032, p = 0.72, pFDR = 0.99	B = 2.07e-03, p = 0.97, pFDR = 0.99
**Volume of grey matter in Occipital Fusiform Gyrus (left)**	β = -0.054, p = 0.55, pFDR = 0.99	B = -0.019, p = 0.74, pFDR = 0.99
**Volume of grey matter in Occipital Fusiform Gyrus (right)**	β = -0.071, p = 0.44, pFDR = 0.97	B = 0.016, p = 0.78, pFDR = 0.99
**Volume of grey matter in Frontal Operculum Cortex (left)**	β = -0.017, p = 0.84, pFDR = 0.99	B = -0.064, p = 0.24, pFDR = 0.93
**Volume of grey matter in Frontal Operculum Cortex (right)**	β = 0.15, p = 0.097, pFDR = 0.75	B = -0.045, p = 0.42, pFDR = 0.93
**Volume of grey matter in Central Opercular Cortex (left)**	β = 0.021, p = 0.8, pFDR = 0.99	B = -0.027, p = 0.61, pFDR = 0.99
**Volume of grey matter in Central Opercular Cortex (right)**	β = -0.12, p = 0.16, pFDR = 0.94	B = -8.50e-03, p = 0.87, pFDR = 0.99

**Volume of grey matter in Parietal Operculum Cortex (left)**	β = -0.069, p = 0.44, pFDR = 0.97	B = -0.015, p = 0.79, pFDR = 0.99
**Volume of grey matter in Parietal Operculum Cortex (right)**	β = 0.016, p = 0.85, pFDR = 0.99	B = -0.041, p = 0.46, pFDR = 0.93
**Volume of grey matter in Planum Polare (left)**	β = 0.15, p = 0.093, pFDR = 0.75	B = -0.053, p = 0.33, pFDR = 0.93
**Volume of grey matter in Planum Polare (right)**	β = -0.033, p = 0.68, pFDR = 0.99	B = -0.062, p = 0.23, pFDR = 0.93
**Volume of grey matter in Heschl's Gyrus (includes H1 and H2) (left)**	β = -0.066, p = 0.43, pFDR = 0.97	B = 0.039, p = 0.47, pFDR = 0.93
**Volume of grey matter in Heschl's Gyrus (includes H1 and H2) (right)**	β = -0.15, p = 0.081, pFDR = 0.75	B = 0.044, p = 0.43, pFDR = 0.93
**Volume of grey matter in Planum Temporale (left)**	β = -0.066, p = 0.44, pFDR = 0.97	B = -6.52e-03, p = 0.9, pFDR = 0.99
**Volume of grey matter in Planum Temporale (right)**	β = 0.036, p = 0.67, pFDR = 0.99	B = -6.12e-03, p = 0.91, pFDR = 0.99
**Volume of grey matter in Supracalcarine Cortex (left)**	β = 0.13, p = 0.14, pFDR = 0.91	B = -0.098, p = 0.071, pFDR = 0.93
**Volume of grey matter in Supracalcarine Cortex (right)**	β = 0.15, p = 0.079, pFDR = 0.75	B = -0.11, p = 0.046, pFDR = 0.92
**Volume of grey matter in Occipital Pole (left)**	β = -0.1, p = 0.27, pFDR = 0.97	B = 0.074, p = 0.19, pFDR = 0.93
**Volume of grey matter in Occipital Pole (right)**	β = -0.063, p = 0.46, pFDR = 0.97	B = 0.046, p = 0.39, pFDR = 0.93
**Volume of grey matter in Thalamus (left)**	β = -0.13, p = 0.15, pFDR = 0.91	B = 0.039, p = 0.49, pFDR = 0.93
**Volume of grey matter in Thalamus (right)**	β = -0.12, p = 0.17, pFDR = 0.97	B = 0.041, p = 0.46, pFDR = 0.93
**Volume of grey matter in Caudate (left)**	β = 0.1, p = 0.25, pFDR = 0.97	B = -0.064, p = 0.24, pFDR = 0.93
**Volume of grey matter in Caudate (right)**	β = 0.11, p = 0.18, pFDR = 0.97	B = -0.037, p = 0.49, pFDR = 0.93
**Volume of grey matter in Putamen (left)**	β = 0.03, p = 0.74, pFDR = 0.99	B = 1.51e-03, p = 0.98, pFDR = 0.99
**Volume of grey matter in Putamen (right)**	β = 0.063, p = 0.48, pFDR = 0.97	B = -1.10e-03, p = 0.98, pFDR = 0.99
**Volume of grey matter in Pallidum (left)**	β = 0.12, p = 0.2, pFDR = 0.97	B = -0.065, p = 0.25, pFDR = 0.93
**Volume of grey matter in Pallidum (right)**	β = 0.068, p = 0.46, pFDR = 0.97	B = -0.041, p = 0.48, pFDR = 0.93
**Volume of grey matter in Hippocampus (left)**	β = 0.052, p = 0.52, pFDR = 0.98	B = 0.057, p = 0.27, pFDR = 0.93
**Volume of grey matter in Hippocampus (right)**	β = 0.036, p = 0.67, pFDR = 0.99	B = 0.021, p = 0.69, pFDR = 0.99
**Volume of grey matter in Amygdala (left)**	β = 0.048, p = 0.55, pFDR = 0.99	B = 0.078, p = 0.13, pFDR = 0.93
**Volume of grey matter in Amygdala (right)**	β = 0.13, p = 0.1, pFDR = 0.75	B = 0.042, p = 0.4, pFDR = 0.93
**Volume of grey matter in Ventral Striatum (left)**	β = -0.028, p = 0.73, pFDR = 0.99	B = 9.73e-03, p = 0.85, pFDR = 0.99
**Volume of grey matter in Ventral Striatum (right)**	β = 0.014, p = 0.86, pFDR = 0.99	B = -0.012, p = 0.81, pFDR = 0.99

Table S10

label	acronym
**anterior corona radiata**	ACR
**anterior limb of internal capsule**	ICant
**acoustic radiation**	AR
**anterior thalamic radiation**	ATR
**body of corpus callosum**	CCbody
**cingulum cingulate gyrus**	CCG
**cingulum hippocampus**	CH
**cerebral peduncle**	CP
**corticospinal tract**	CST
**external capsule**	EC
**fornix**	Fornix
**fornix cres+stria terminalis**	FCST
**forceps major**	FMaj
**forceps minor**	FMin
**genu of corpus callosum**	CCgenu
**inferior cerebellar peduncle**	ICP
**inferior fronto-occipital fasciculus**	IFOF
**inferior longitudinal fasciculus**	ILF
**middle cerebellar peduncle**	MCP
**medial lemniscus**	ML
**posterior corona radiata**	PCR
**pontine crossing tract**	PCT
**posterior limb of internal capsule**	ICpos
**parahippocampal part of cingulum**	PrCing
**posterior thalamic radiation**	PTR
**retrolenticular part of internal capsule**	ICretr
**superior cerebellar peduncle**	SCP
**superior corona radiata**	SCR
**superior fronto-occipital fasciculus**	SFOF
**superior longitudinal fasciculus**	SLF
**splenium of corpus callosum**	CCspl
**sagittal stratum**	SS
**superior thalamic radiation**	STR
**tapetum**	tapetum
**uncinate fasciculus**	UF
